# Supplementary figures and images for: Efficacy and safety of vamorolone in Duchenne muscular dystrophy: An 18-month interim analysis of a non-randomized open-label extension study
Source: PLoS Med. 2020 Sep 21;17(9):e1003222. doi: 10.1371/journal.pmed.1003222 (PMC7505441; doi:10.1371/journal.pmed.1003222)

**S1 Fig. Participant-specific dose levels in VBP15-LTE.**


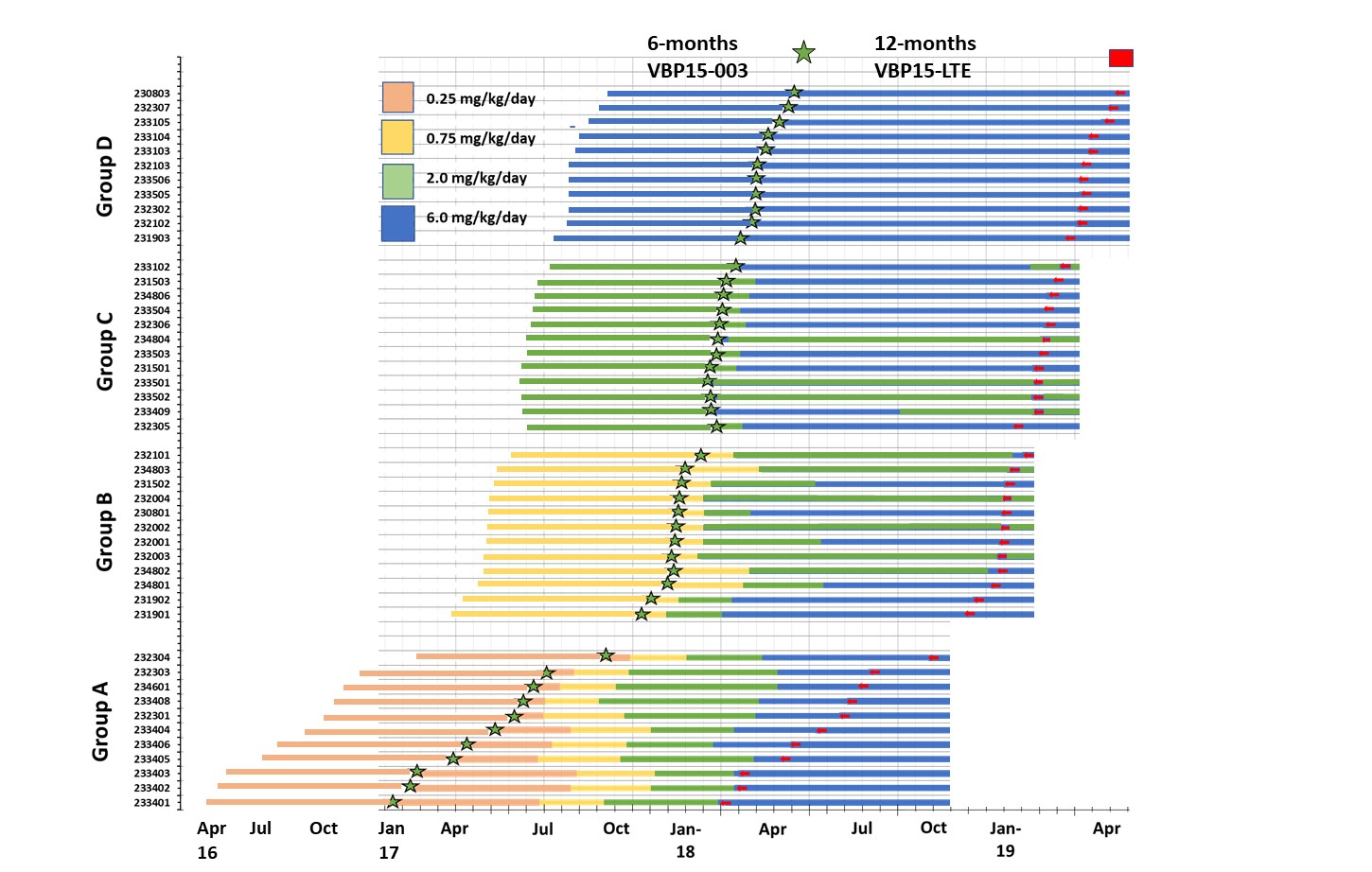

Supplement: S1 Fig — Each horizontal line indicates a participant in VBP15-LTE. x-Axis is date. The green star indicates the transition point of each participant from the time of VBP15-003 completion to VBP15-LTE enrollment. VBP15-003 was a 24-week dose-ranging study, with each group (y-axis) of 12 participants started at a specific dose (doses indicated by colors and legend; 0.25 mg/kg/day, 0.75 mg/kg/day, 2.0 mg/kg/day, 6.0 mg/kg/day). VBP15-LTE is a 2-year long-term extension study, but data presented here are from the midpoint (12 months of treatment in VBP15-LTE); the 12-month interim assessment time point is indicated by the red box in each participant. Dose escalations and dose de-escalations were permitted in VBP15-LTE at the discretion of the treating physician and the participant’s family; dose changes are indicated for each participant by the change in color. (DOCX) [file pmed.1003222.s002.docx]
