# Supplementary material for: Efficacy and safety of vamorolone in Duchenne muscular dystrophy: An 18-month interim analysis of a non-randomized open-label extension study
Source: PLoS Med. 2020 Sep 21;17(9):e1003222. doi: 10.1371/journal.pmed.1003222 (PMC7505441; doi:10.1371/journal.pmed.1003222)
Supplement: S1 iSAP — (PDF) [file pmed.1003222.s003.pdf]

**ReveraGen BioPharma, Inc.**

# INTERIM STATISTICAL ANALYSIS PLAN

| | |
|---|---|
| **Protocol Title:** | Amendment #1 for a 24-month Phase II Open-label, Multicenter Extension Study to Assess the Long-term Safety and Efficacy of Vamorolone in Boys with Duchenne Muscular Dystrophy (DMD) |
| **Protocol Number** | VBP15-LTE-A1 |
| **Phase:** | Phase II |
| **Sponsor:** | ReveraGen BioPharma, Inc.<br>155 Gibbs Street<br>Suite 433<br>Rockville, MD 20850 |
| **Author:** | Summit Analytical, LLC<br>8354 Northfield Blvd., Bldg. G Suite 3700<br>Denver, CO 80238 |
| **ISAP Date:** | 2019-06-21 |
| **Status:** | Final v1.0 |

| SUMMITANALYTICAL | **Statistical Analysis Plan Approval Form** |
|---|---|

| | |
|---|---|
| **Sponsor:** | ReveraGen BioPharma, Inc. |
| **Protocol:** | VBP15-LTE-A1 |
| **Protocol Title:** | Amendment #1 for a 24-month Phase II Open-label, Multicenter Extension Study to Assess the Long-term Safety and Efficacy of Vamorolone in Boys with Duchenne Muscular Dystrophy (DMD) |
| **ISAP Version:** | Final v1.0 |
| **ISAP Date:** | 21June2019 |

The interim statistical analysis plan has been reviewed and approved.

**Sponsor:** *ReveraGen BioPharma, Inc.*
*Eric Hoffman*
*CEO*

[signature] _21 June 2019_______
Signature Date

**Author:** *Summit Analytical LLC*
*Mark Jaros, PhD*
*Senior Vice President*

[signature] 21 JUN 2019
Signature Date

**Reviewer:** *Laurie S. Conklin*
*Director, Clinical Programs*

[signature] June 21, 2019________
Signature Date

[redacted]

# 1. TABLE OF CONTENTS

1. TABLE OF CONTENTS ..... 3
2. LIST OF ABBREVIATIONS AND DEFINITION OF TERMS ..... 6
3. INTRODUCTION ..... 8
3.1. Preface ..... 8
3.2. Purpose of Analyses ..... 8
3.3. Summary of Statistical Analysis Changes to the Protocol ..... 8
4. STUDY OBJECTIVES AND ENDPOINTS ..... 9
4.1. Study Objectives ..... 9
4.1.1. Primary Objective ..... 9
4.1.2. Secondary Objectives ..... 9
4.1.3. Exploratory Objectives ..... 9
4.2. Interim Analysis Endpoints ..... 10
5. STUDY METHODS ..... 13
5.1. Overall Study Design ..... 13
5.2. Inclusion – Exclusion Criteria and General Study Population ..... 17
5.3. Randomization and Blinding ..... 17
6. SAMPLE SIZE ..... 18
7. GENERAL CONSIDERATIONS FOR THE INTERIM ANALYSIS ..... 19
7.1. Analysis Populations ..... 19
7.1.1. Safety Population ..... 19
7.1.2. Full Analysis Set (FAS) ..... 19
7.1.3. Control Population DNHS Study Steroid Naïve Data Set ..... 19
7.1.4. Control Population Corticosteroid-treatment Subjects ..... 20
7.2. Covariates and Subgroups ..... 21
7.2.1. Planned Covariates ..... 21
7.2.2. Planned Subgroups ..... 22
7.3. Management of Analysis Data ..... 22
7.3.1. Data Handling ..... 22
7.3.2. Missing Data ..... 22
7.3.3. Handling of Early Termination Visit Information ..... 24
7.3.4. Pooling of Investigational Sites ..... 24
7.3.5. Coding Conventions for Events and Medications ..... 24
7.3.6. Baseline Visits ..... 24
7.3.7. Analysis Software ..... 24
7.4. Planned Study Analyses ..... 24
7.4.1. Statistical Summaries: Descriptive and Inferential ..... 24
7.5. Multiple Testing Procedures ..... 24
8. MONTH 12 INTERIM ANALYSIS ..... 25
8.1. Subject Summary Grouping ..... 25
8.2. Subject Disposition ..... 25
8.3. Demographics and Baseline Characteristics ..... 25
8.4. TTSTAND, TTRW, TTCLIMB, CQMS, 6MWT, and NSAA ..... 25
8.5. Adverse Events ..... 26

8.6. BMI ........ 27
8.7. Height Percentiles ........ 28
8.8. Blood Pressure ........ 28
8.9. White Cell Count and PD Biomarkers ........ 29

9. REPORTING CONVENTIONS ........ 30

9.1. General Reporting Conventions ........ 30

10. POPULATION SUMMARY CONVENTIONS ........ 32

11 REFERENCES ........ 33

11.1 Calculating BMI Z-Scores ........ 35
11.2 ISAP Amendment Summary of Changes ........ 36

## LIST OF TABLES

Table 1 12-Month Interim Analyses of Disposition, Demographics and Baseline Characteristics, Safety, Efficacy, and PD Biomarkers .......... 10
Table 2 Dose level group .......... 14
Table 3 Schedule of Study Activities .......... 15
Table 4 Study Schema .......... 17

# 2. LIST OF ABBREVIATIONS AND DEFINITION OF TERMS

Only abbreviations and terms relevant to this interim statistical analysis plan (ISAP) are repeated herein. The reader is referred to the protocol for the complete and comprehensive list of abbreviations and definitions of terms for the study.

| | |
|---|---|
| AE | adverse event |
| BL | baseline |
| BMI | body mass index |
| BP | blood pressure |
| CINRG | Cooperative International Neuromuscular Research Group |
| cm | centimeter |
| CQMS | CINRG Quantitative Measurement System |
| CTCAE | Common Terminology Criteria for Adverse Events |
| CTX1 | Telopeptides of type 1 collagen (C-terminal) |
| DHEA | dehydroepiandrosterone |
| DMD | Duchenne muscular dystrophy |
| DSMB | Data and Safety Monitoring Board |
| eCRF | electronic case report form |
| EDC | electronic data capture |
| FDA | Food and Drug Administration |
| GCP | Good Clinical Practice |
| HIPAA | Health Insurance Portability and Accountability Act |
| hr | hour |
| ICF | informed consent form |
| ICH | International Conference on Harmonisation |
| IND | Investigational New Drug |
| IRB | Institutional Review Board |
| ISAP | interim statistical analysis plan |
| LS | least squares |
| m | meter |
| MedDRA | Medical Dictionary for Regulatory Activities |
| mg | milligram |
| min | minute |
| mL | milliliter |
| No., n | number |

| | |
|---|---|
| nmol | nanomole |
| P1NP | serum aminoterminal propeptide of type I collagen |
| SAE | serious adverse event |
| SAP | statistical analysis plan |
| SCR | screening |
| SD | standard deviation |
| SOC | system organ class |
| TEAE | treatment-emergent adverse event |
| TTCLIMB | Time to Climb (Test) |
| TTRW | Time to Run/Walk (Test) |
| TTSTAND | Time to Stand (Test) |
| vs. | versus |
| WBC | white blood cell |
| WHO | World Health Organization |

# 3. INTRODUCTION

## 3.1. Preface

This document presents a statistical analysis plan (SAP) for a partial interim data review for ReveraGen BioPharma, Inc. Protocol VBP15-LTE-A1 (*Amendment #1 for a 24-month Phase II Open-label, Multicenter Long-term Extension Study to Assess the Long-term Safety and Efficacy of Vamorolone in Boys with Duchenne Muscular Dystrophy (DMD))*. This ISAP will provide the details and methods for analysis and reporting of certain subject characteristic, disposition, safety, efficacy, and pharmacodynamic (PD) data.

Reference materials for this statistical plan include the protocol VBP15-LTE (Amendment 1 Dated: 03 October 2018).

This ISAP is for the 12-month study mid-point informal analysis.

## 3.2. Purpose of Analyses

These interim analyses are to partially assess the safety, clinical efficacy, and PD data of the vamorolone extension study for subjects who have completed VBP15-002, VBP15-003 and the first 12 months of VBP15-LTE. This will aid regulatory filings, recruitment into VBP15-004, publications, and will build a stronger case for a claim of improved safety relative to corticosteroids and long-term efficacy.

## 3.3. Summary of Statistical Analysis Changes to the Protocol

Interim analyses described in this plan are in addition to the analyses described in the study protocol as no formal interim analysis is prescribed in the protocol.

# 4. STUDY OBJECTIVES AND ENDPOINTS

The full study objectives and endpoints defined in the protocol include safety, tolerability, clinical efficacy, and PD. Though not all of the protocol listed objectives and pre-specified endpoints are presented in this ISAP, we provide them here as general background.

## 4.1. Study Objectives

### 4.1.1. Primary Objective

1. To evaluate the long-term safety and tolerability of vamorolone, administered orally at daily doses up to 6.0 mg/kg over a 24-month Treatment Period, in young boys with DMD who completed protocol VBP15-003; and

2. To compare the efficacy, as measured by the Time to Stand Test (TTSTAND), of vamorolone administered orally at daily doses up to 6.0 mg/kg over a 24-month Treatment Period vs. untreated DMD historical controls in young boys with DMD.

### 4.1.2. Secondary Objectives

1. To investigate the effects of vamorolone, administered orally at daily doses up to 6.0 mg/kg over a 24-month Treatment Period on serum PD biomarkers of safety (insulin resistance, adrenal axis suppression, and bone turnover);

2. To investigate the effects of vamorolone, administered orally at daily doses up to 6.0 mg/kg over a 24-month Treatment Period, on muscle strength, mobility and functional exercise capacity vs. untreated DMD historical controls as measured by Time to Climb Test (TTClimb), Time to Run/Walk Test (TTRW), North Star Ambulatory Assessment (NSAA), 6-minute Walk Test (6MWT), and Cooperative International Neuromuscular Research Group Quantitative Measurement System (CQMS) in young boys with DMD; and

3. To compare the safety, as assessed by bone age, spine fractures, body mass index (BMI) Z-score, and height Z-score, of vamorolone administered orally at daily doses up to 6.0 mg/kg over a 24-month Treatment Period vs. prednisone- and deflazacort-treated historical control boys with DMD.

### 4.1.3. Exploratory Objectives

1. To investigate the effects of vamorolone administered orally at daily doses up to 6.0 mg/kg over a 24-month Treatment Period on Quality of Life measures (Pediatric Outcomes Data Collection Instrument [PODCI]);

2. To investigate the effects of vamorolone administered orally at daily doses up to 6.0 mg/kg over a 24-month Treatment Period on additional exploratory PD biomarkers; and

3. To determine if established genetic modifiers of DMD (gene polymorphisms associated with disease severity, or response to glucocorticoid treatment) are similarly associated with vamorolone-treated DMD subjects (baseline disease severity, or response to vamorolone treatment).

## 4.2. Interim Analysis Endpoints

Only a subset of the presentations described in the protocol will be presented in the ISAP. Aside from disposition and demographics and baseline characteristics, **Table 1** lists the safety, efficacy, and PD endpoints that will be included in the interim analysis.

**Table 1 12-Month Interim Analyses of Disposition, Demographics and Baseline Characteristics, Safety, Efficacy, and PD Biomarkers**

| Parameters | Details | Study comparators |
|---|---|---|
| **Efficacy Endpoints** | | |
| TTSTAND raw scores (sec) and velocity (rise/sec) | Descriptive summary and test of 18-month change from baseline by ANCOVA | LTE (cohort 3,4) (18 months) v. DNHS steroid-naïve (18 months) |
| TTSTAND raw scores (sec) and velocity (rise/sec) | Descriptive summary only (no inferential statistics) | LTE (cohort 3,4) (18 months) v. CINRG daily prednisone (12 months) |
| TTSTAND - Time to >5 sec and Time to >10 sec | Kaplan-Meier estimates | LTE (cohort 3,4) (18 months) v. DNHS steroid-naïve (18 months) |
| TTRW raw scores (sec) and velocity (meter/sec) | Descriptive summary and test of 18-month change from baseline by ANCOVA | LTE (cohort 3,4) (18 months) v. DNHS steroid-naïve (18 months) |
| TTRW raw scores (sec) and velocity (meter/sec) | Descriptive summary only (no inferential statistics) | LTE (cohort 3,4) (18 months) v. CINRG daily prednisone (12 months) |
| TTCLIMB raw scores (sec) and velocity (task/sec) | Descriptive summary and test of 18-month change from baseline by ANCOVA | LTE (cohort 3,4) (18 months) v. DNHS steroid-naïve (18 months) |
| TTCLIMB raw scores (sec) and velocity (task/sec) | Descriptive summary only (no inferential statistics) | LTE (cohort 3,4) (18 months) v. CINRG daily prednisone (12 months) |
| CINRG QMS hip and elbow extension and flexion | Descriptive summary and test of 18-month change from baseline by ANCOVA | LTE (cohort 3,4) (18 months) v. DNHS steroid-naïve (18 months) |

| | | |
|---|---|---|
| 6MWT | Descriptive summary and test of 18-month change from baseline by ANCOVA | LTE (cohort 3,4) (18 months) v. DNHS steroid-naïve (18 months) |
| NSAA composite score | Descriptive summary and test of 18-month change from baseline by ANCOVA | LTE (cohort 3,4) (18 months) v. DNHS steroid-naïve (18 months) |
| **Safety Endpoints** | | |
| BMI Z-score and raw score | Descriptive summary and test of 18-month change from baseline by ANCOVA | LTE (cohort 3,4) (18 months) v. DNHS steroid-naïve (18 months) |
| BMI Z-score and raw score | Descriptive summary and test of 18-month change from baseline by ANCOVA | LTE (cohort 3,4) (18 months) v. CINRG daily prednisone (12 months) |
| Height percentile | Descriptive summary and test of 18-month change from baseline by ANCOVA | LTE (cohort 3,4) (18 months) v. DNHS steroid-naïve (18 months) |
| Height percentile | Descriptive summary and test of 18-month change from baseline by ANCOVA | LTE (cohort 3,4) (18 months) v. DNHS steroid-treated (18 months) |
| Height percentile | Descriptive summary and test of 18-month change from baseline by ANCOVA | LTE (cohort 3,4) (18 months) v. CINRG daily prednisone (12 months) |
| Adverse events | Descriptive summary of TEAEs and SAEs | LTE (cohort 3,4) (18 months)<br>DNHS steroid-treated (18 months)<br>CINRG daily prednisone (12 months) |
| Blood pressure (SBP, DBP, and mean arterial pressure) | Descriptive summary and cross-sectional comparison by ANCOVA analysis | LTE (cohort 3,4) (18 months) v. DNHS steroid-naive (18 months) |
| Blood pressure (SBP, DBP, and mean arterial pressure) | Descriptive summary and cross-sectional comparison by ANCOVA analysis | LTE (cohort 3,4) (18 months) v. DNHS steroid-treated (18 months) |
| Blood pressure (SBP, DBP, and mean arterial pressure) | Descriptive summary and cross-sectional comparison by ANCOVA analysis | LTE (cohort 3,4) (18 months) v. CINRG prednisone (12 months) |
| Blood pressure (SBP, DBP, and mean arterial pressure) | Descriptive summary and test of 18 month-change from baseline by ANCOVA | LTE (cohort 3,4)<br>CINRG prednisone (12 months)<br>DNHS steroid treated (18 months)<br>DNHS steroid-naïve (18 months) |
| **Biomarker Endpoints** | | |
| Bone biomarker (Osteocalcin, CTX1, P1NP) observed | Descriptive summary and comparison of observed response by dose during assessment by timepoint between cohorts | LTE cohort 2 v. cohort 3 v. cohort 4 (18 months) |

| | | |
|---|---|---|
| First in morning cortisol | Descriptive summary and comparison of observed response by dose during assessment by timepoint between cohorts | LTE cohort 2 v. cohort 3 v. cohort 4 (18 months) |
| Fasting insulin, Fasting glucose | Descriptive summary and comparison of observed response by dose during assessment between cohorts | LTE cohort 2 v. cohort 3 v. cohort 4 (18 months) |
| White blood cell count and differentials:<br>- Absolute and percent neutrophils<br>- Absoluate and percent lymphocyteslu<br>- Total white blood cell count | Descriptive summary and comparison of observed count by dose during assessment by timepoint between cohorts | LTE cohort 2 v. cohort 3 v. cohort 4 (18 months) |

# 5. STUDY METHODS

## 5.1. Overall Study Design

This Phase II long term extension (LTE) study is an open-label, multicenter study to evaluate the long-term safety, tolerability, clinical efficacy, and PD of vamorolone at dose levels up to 6.0 mg/kg administered daily by liquid oral suspension over a Treatment Period of 24 months to young boys with DMD who participated in the VBP15-002 Phase IIa and VBP15-003 Phase IIa extension core studies. Overall study duration is 24- 25 months.

Subjects who completed the Phase IIa extension VBP15-003 Study Week 24 Follow-up assessments were eligible for enrollment into the VBP15-LTE study. The LTE study is comprised of a Pretreatment Baseline Period of up to 24 hours, which begins with the signing of the VBP15-LTE-specific informed consent form (ICF), a 24-month Treatment Period, and an up to 5-week Dose-tapering Period for subjects who elect to transition off vamorolone treatment at the end of the study. Subjects prematurely discontinued from the VBP15-LTE long term extension study will not be replaced. Subjects completing the VBP15-LTE study are given the option of continuing vamorolone treatment under an Expanded Access Protocol, or compassionate use protocol.

For subjects who enroll in the VBP15-LTE long term extension study within 28 days after completion of all VBP15-003 Week 24 final assessments, many of the safety, efficacy, and PD assessments performed at the VBP15-003 Week 24 Final Visit may be used to determine extension study eligibility and/or to provide baseline study data for the extension study and do not need to be repeated at the VBP15-LTE Baseline Day -1 Visit. For these subjects, additional long-term extension study procedures will still be performed at the Baseline Day -1 Visit, within 24 hours prior to administration of the first dose of study drug in the long-term extension study. Subjects who enroll in the VBP15-LTE long term extension study > 28 days after the date of the VBP15-003 Week 24 Final Visit must have all Baseline Day -1 assessments performed, according to the schedule in Table 8.

Subjects received vamorolone at one of four dose levels (0.25, 0.75, 2.0, or 6.0 mg/kg/day) over the course of the 24-week VBP15-003 trial, and then continued on this dose in the current VBP15-LTE, unless dose-escalations were done. Subjects retained the enrollment numbers assigned to them for the Phase IIa extension VBP15-003 core study. If dose-limiting toxicities are observed in this LTE study, dose level(s) can be de-escalated, as needed. VBP15-LTE is carried out with daily dosing.

Site Investigators may opt to dose escalate to a higher dose level during the VBP15-LTE once the subject has been on the initial dose in VBP15-LTE for at least one month, the next higher dose is determined to be safe in the VBP15-002 Phase IIa Study, and no safety issues with that dose have emerged in the VBP15-003 Phase IIa extension study.

Table 2 **Dose level group**

| Planned Dose Level Group | No. Subjects in Dose Level Group | Vamorolone Dose |
|---|---|---|
| 1 | 12 | 0.25 mg/kg/day |
| 2 | 12 | 0.75 mg/kg/day |
| 3 | 12 | 2.0 mg/kg/day |
| 4 | 12 | 6.0 mg/kg/day |

**Table 3 Schedule of Study Activities**

| | Pretreatment Period | | Treatment Period | | | | | | | | | Dose-tapering Period |
|---|---|---|---|---|---|---|---|---|---|---|---|---|
| | Baseline | | | | | | | | | | | |
| | Day | | Month | | | | | | | | | |
| **Study Day or Month/Visit** | **-1[a]** | **1[b]** | **1 (±7d)** | **3 (±7d)** | **6 (±7d)** | **9 (±7d)** | **12 (±7d)** | **15 (±7d)** | **18 (±7d)** | **21 (±7d)** | **24[c] (±7d)** | **24-25[d] (±7d)** |
| Inclusion/Exclusion Criteria | X | | | | | | | | | | | |
| Informed Consent | X[e] | | | | | | | | | | | |
| Enrollment | X | | | | | | | | | | | |
| Interim Medical History | X[f] | | | | | | | | | | | |
| Medication History | X[g] | | | | | | | | | | | |
| Physical Examination | X[h] | | | | | | X | | | | X | |
| Height | X | | | | | | X | | | | X | |
| Weight | X | | | X | X | X | X | X | X | X | X | |
| Vital Signs[i] | X | | X | X | X | X | X | X | X | X | X | X |
| Blood for Clinical Labs[j] | X[h] | | | | X | | X | | X | | X | X |
| Urinalysis[k] | X[h] | | | | X | | X | | X | | X | |
| Blood for Serum PD Biomarker Panel[l] | X[h] | | | | X | | X | | X | | X | X |
| Fasting morning blood for insulin, glucose, cortisol[m] | | | | | | X | | | | X | | |
| Blood for DNA Testing | | | | | | | | | | | X | |
| 12-lead ECG[n] | X[h] | | | | | | X | | | | X | |
| Dispense Study Medication | X | | | X | X | X[o] | X | X | X | X[o] | X | |
| Return Study Medication/ Compliance Monitoring | | | | X | X | X | X | X | X | X | X | X |
| Study Medication Dosing | | X → | | | | | | | | | X | |
| Vamorolone dose tapering[p,q] | | | | | | | | | | | | X |
| Time to Stand Test (TTSTAND) | X[h] | | | | | | X | | | | X | |
| Time to Climb Test (TTCLIMB) | X[h] | | | | | | X | | | | X | |
| Time to Run/Walk Test (TTRW) | X[h] | | | | | | X | | | | X | |
| NSAA[r] | X[h] | | | | | | X | | | | X | |
| Quantitative Muscle Testing (QMT) | X[h] | | | | | | X | | | | X | |
| Six-minute Walk Test (6MWT) | X[h] | | | | | | X | | | | X | |
| Pediatric Outcomes Data Collection Instrument (PODCI) | X[h] | | | | | | X | | | | X | |
| Spine x-ray | | | | | | | | | | | X | |
| Hand x-ray | | | | | | | | | | | X | |
| Dispense Subject Diaries[s] | X | | | X | X | X | X | X | X | X | X | |
| Return Subject Diaries | | | X | X | X | X | X | X | X | X | X | X |
| AE/SAE Recording[t] | X → | | | | | | | | | | X | X |
| Concomitant Medications | | X → | | | | | | | | | X | X |
| Discharge from Study | | | | | | | | | | | X[u] | X[v] |

d = day(s); w = week.

a. Baseline Day -1, within 24 hours prior to administration of the first dose of study drug. The Baseline Visit for Study VBP15-LTE can coincide with the Week 24 Final Visit for Study VBP15-003, or the final visit following dose-tapering for subjects who dose taper, or may occur up to 8 weeks after the date of the final visit following dose tapering.
b. Treatment Day 1 begins at the time of administration of the first dose of VBP15-LTE study medication at home. No scheduled study visit will occur on Day 1.
c. Subjects who prematurely discontinue from the study prior to Month 24 should complete the Month 24 assessments at the time of early discontinuation.
d. All subjects EXCEPT those who elect to continue vamorolone therapy in a further extension study must continue in the Dose-tapering Period and have their vamorolone dose tapered at weekly intervals over a 2-5week period prior to discharge from this study. Subjects participating in the Dose-tapering Period will have one study site visit during this period, at the end of dose tapering (Months 24-25) (see Protocol Section 6.3.4).
e. Informed Consent for this extension study may be obtained at the Study VBP15-003 Week 24 Final Visit or within 8 weeks after the final dose-tapering visit for subjects who dose-taper, after completion of all final VBP15-003 study assessments and prior to any VBP15-LTE extension study-specific procedures. For all subjects, informed consent for VBP15-LTE participation will be given on Study Day -1, within 24 hours of administration of first dose of study medication in VBP15-LTE.
f. Interim Medical History will be collected on Baseline Day -1 for all subjects, and will include any AEs that occurred during the VBP15-003 core study and are ongoing at the time of entry into VBP15-LTE (see Protocol Section 7.2.1).
g. Any changes in medication/therapy including administration of new medication(s), change of dose, or discontinuation of medication after completion of the VBP15-003 core study and prior to administration of the first dose of study medication in VBP15-LTE will be captured as Prior Medications.
h. If the Baseline Day -1 Visit occurs ≤ 28 days after the date of the Week 24 Final Visit in the VBP15-003 core study, these assessments may be used for the VBP15-LTE study Baseline assessments. Clinical laboratory and urinalysis test results from the VBP15-003 Week 24 Final Visit may be used and should be reviewed by the Site Investigator to determine eligibility for Baseline Day -1 enrollment into the VBP15-LTE long-term extension study.
i. Supine blood pressure, oral temperature, respiratory rate, and heart rate.
j. Blood for hematology, chemistry, and lipids.
k. Urinalysis by dipstick and microscopic analysis.
l. Blood collected for pharmacodynamic safety biomarkers and exploratory safety and efficacy biomarkers.
m. Blood will be collected for insulin, glucose, and cortisol determination in the morning after subjects have fasted for ≥ 6 hours, prior to the daily dose of study medication.
n. ECG recorded after subject has rested quietly in a supine position for at least 5 minutes.
o. The dose of study medication on the days of the Month 9 and 21 Visits will be administered after 1) a morning fasting blood draw for insulin, glucose, and cortisol; and 2) breakfast provided by the study site. All other doses will be taken at home.
p. Only for subjects who will participate in the Dose-tapering Period.
q. Subjects who elect to switch to standard of care glucocorticoids, or discontinue vamorolone and not begin glucocorticoid treatment for DMD at the end of the study will have their vamorolone dose tapered at weekly intervals to a dose of 0 mg/kg/day prior to final study assessments (see Protocol Section 6.3.4).
r. North Star Ambulatory Assessment; includes the Time to Stand Test (TTSTAND).
s. Subject diaries used to record any changes to concomitant medications taken and any AEs experienced during the study.
t. All AEs and SAEs must be collected in the source documents and eCRF from the date of the subject's written informed consent until the Month 24 Visit or the subject's participation in the study is completed. Ongoing AEs will be followed to resolution, stabilization, or until such time the Investigator agrees follow-up is not necessary.
u. Subjects who elect to continue vamorolone therapy in a subsequent extension study may be discharged from the study following completion of all final Month 24 procedures.
v. Subjects who participate in the Dose-tapering Period may be discharged from the study following completion of all final Dose-tapering Visit assessments (see Protocol Section 6.3.4).

**Table 4 Study Schema**

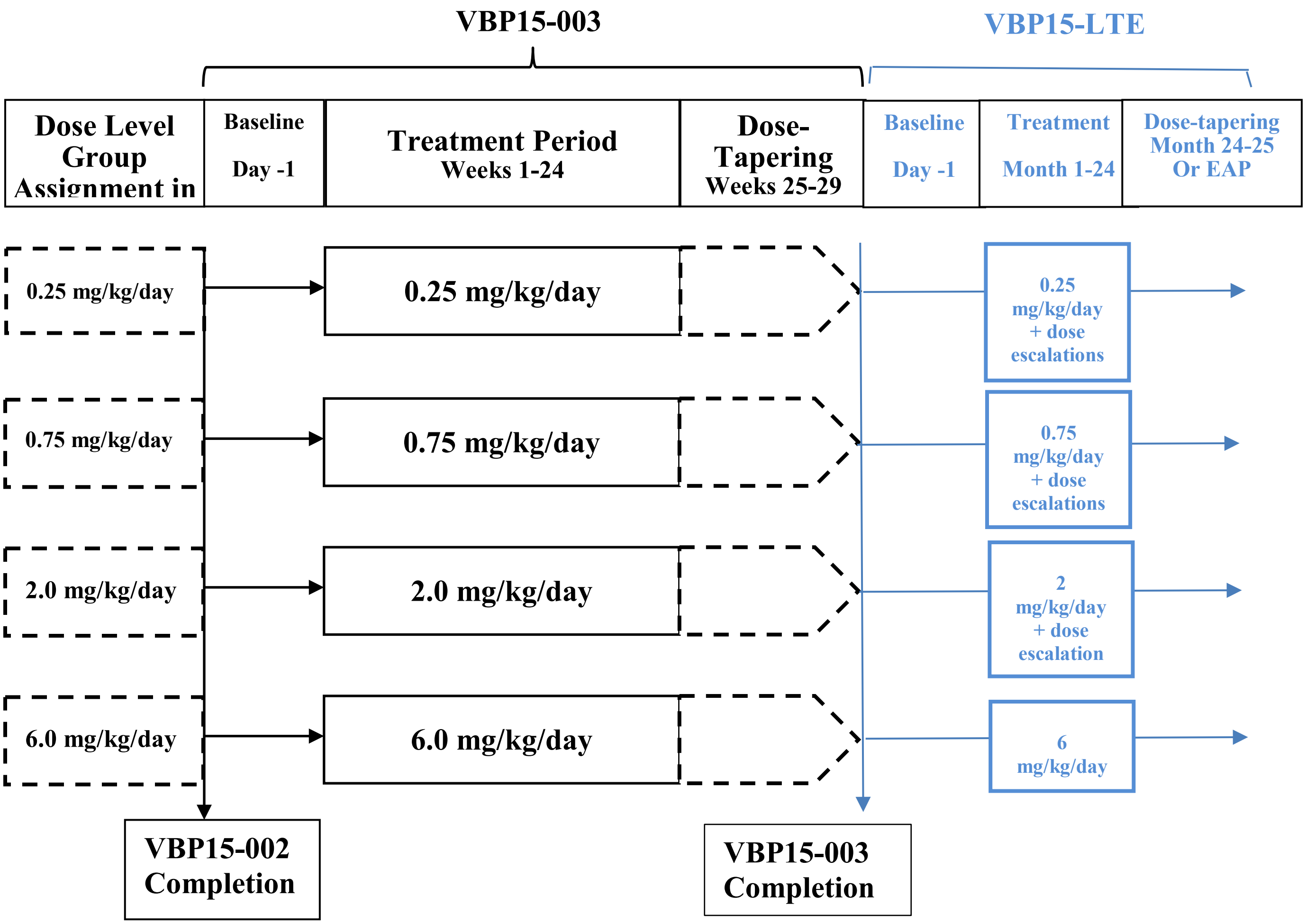


## 5.2. Inclusion – Exclusion Criteria and General Study Population

The inclusion and exclusion criteria defined in the protocol apply to all subjects and are not repeated here in the ISAP.

## 5.3. Randomization and Blinding

This is an open-label study, and no randomization schedule or blinding of study medication is applicable.

# 6. SAMPLE SIZE

Eligible subjects are those who enrolled in the VBP15-003 study and completed the Phase IIa extension VBP15-003 Study Week 24 Follow-up assessments (n=12 per vamorolone dose group). There is no placebo control, instead untreated (steroid naïve) natural history control population data (efficacy and safety comparators) is from the CINRG DNHS study of ~400 DMD boys (steroid- naïve) (McDonald et al. 2019). Corticosteroid-treated historical control data are from a clinical trial (daily prednisone-dosing arm) (Escolar et al. 2011), and the CINRG DNHS (steroid-treated).

# 7. GENERAL CONSIDERATIONS FOR THE INTERIM ANALYSIS

## 7.1. Analysis Populations

There will be four (4) analysis populations defined for this study.

### 7.1.1. Safety Population

All subjects who receive at least one dose of vamorolone study medication in the VBP15-LTE extension study will be included in the Safety Population. The Safety Population is the primary analysis population for safety assessments. This is also the modified Intention to Treat (mITT) population.

### 7.1.2. Full Analysis Set (FAS)

All subjects who receive at least one dose of vamorolone study medication in the VBP15-LTE extension study and have at least one post-baseline assessment will be included in the FAS. The FAS is the primary analysis population for clinical efficacy and PD assessments. The FAS population is the mITT population, with the additional requirement of having at least one post-baseline assessment. Subjects who receive at least one dose of vamorolone but never have post-baseline assessments will be excluded.

### 7.1.3. Control Population DNHS Study Steroid Naïve Data Set

To provide a historical control group of steroid-naïve subjects for comparison to VBP15-LTE (VBP15-002 Baseline vs. VB15-LTE 12 months; 18 months total), the CINRG DNHS subjects were matched for key enrollment criteria, as follows:

1. Subjects with 18 months of data.

   Specifics for programming: The visit window tolerance is 16-20 months.
2. Subjects between age 4 and <7 years old at the observation interval start (visit considered as Baseline for this ISAP).
3. Subjects from all regions.
4. Subjects who were not treated with corticosteroids (prednisone, deflazacort; any dose) at any time during 18-month period.
5. Subjects not co-enrolled in other clinical trials.
6. Subjects able to perform the time to run/walk 10m, time to stand from supine, and time to climb 4 stairs at the observation interval start.
7. Subjects having both height and BMI assessments at both observation interval start and interval end (i.e. at 18 months).

**The following data from the CINRG DNHS steroid naïve subjects will be provided:**

1. Demographic information including age

2. Anthropometric information including standing height, calculated height, weight, and BMI
3. Blood pressure
4. Timed function test assessments (the time to run/walk 10m, time to stand from supine, and time to climb 4 stairs) including measured seconds and velocities.
   a. Velocity values will include zeroes imputed for the first visit for which the timed function test cannot be performed due to disease progression.
5. North Star Ambulatory Assessment total score
6. 6-minute walk test distance traveled
7. CQMS hip extension
8. CQMS hip flexion
9. CQMS elbow extension
10. CQMS elbow flexion

### 7.1.4. Control Population Corticosteroid-treatment Subjects

Corticosteroid-treated DMD comparators will be drawn from two studies: CINRG DNHS steroid-treated subjects (McDonald et al. 2018), and a CINRG prednisone clinical trial (Escolar et al. 2011).

**CINRG DNHS steroid-treated:**

To provide a historical control group for VBP15-LTE (VBP15-002 Baseline vs. VB15-LTE 12 months; 18 months total), the CINRG DNHS subjects were matched for key enrollment criteria of ReveraGen VBP15-002/LTE, as follows:

1. Subjects with 18 months of data.
   Specifics for programming: The visit window tolerance is 16-20 months.
2. Subjects between age 4 and <7 years old at baseline visit.
3. Subjects from all regions.
4. Subjects who initiated corticosteroids (prednisone, deflazacort; any dose) prior to baseline visit, and were maintained on corticosteroids throughout the subsequent 18-month period. (Inclusive of those that start steroids at the visit of the start of the 18-month period).
5. Subjects not co-enrolled in other exon-skipping clinical trials.
6. Subjects able to perform the time to run/walk 10m, time to stand from supine, and time to climb 4 stairs at the observation interval start.
7. Subjects having both height and BMI assessments at both observation interval start and interval end (i.e. at 18 months).

**The following data from the CINRG DNHS steroid-treated subjects will be provided:**

1. Demographic information including age

2. Anthropometric information including standing height, calculated height, weight, and BMI
3. Blood pressure
4. Corticosteroid usage information including current use and total lifetime corticosteroid use
5. Timed function test assessments (the time to run/walk 10m, time to stand from supine, and time to climb 4 stairs) including measured seconds and velocities.
    a. Velocity values will include zeroes imputed for the first visit for which the timed function test cannot be performed due to disease progression.
6. North Star Ambulatory Assessment total score
7. 6-minute walk test distance traveled
8. CQMS hip extension vs. steroid naïve.
9. CQMS hip flexion vs. steroid naïve.
10. CQMS elbow extension vs. steroid naïve.
11. CQMS elbow flexion vs. steroid naïve.

**Prednisone clinical trial (Escolar et al. 2011).**

Subjects treated with daily prednisone and having measures at baseline and study end (12 months) will be included.

**The following data from the CINRG prednisone trial will be provided:**

1. Demographic information including age
2. Anthropometric information including standing height, calculated height, weight, and BMI
3. Timed function test assessments (the time to run/walk 10m, time to stand from supine, and time to climb 4 stairs) including measured seconds and velocities.
    a. Velocity values will include zeroes imputed for the first visit for which the timed function test cannot be performed due to disease progression.
4. CQMS hip extension
5. CQMS hip flexion
6. CQMS elbow extension
7. CQMS elbow flexion

## 7.2. Covariates and Subgroups

### 7.2.1. Planned Covariates

Baseline response and age will be included in the ANCOVA statistical analyses as covariates. For the vamorolone subjects, age is calculated as (date of informed consent – birthdate) /365.25. For the prednisone and DNHS subjects, age is calculated as (date of Baseline visit used in this study – birthdate)/365.25. Note that the Baseline visit for a DNHS

subject is the first visit that the subject meets the comparison eligibility criteria and has a non-missing response for at least one endpoint of interest.

#### 7.2.2. Planned Subgroups

None.

### 7.3. Management of Analysis Data

#### 7.3.1. Data Handling

Unscheduled or repeated results will not be analyzed in the ISAP but will be included in data listings.

#### 7.3.2. Missing Data

Every effort will be made to collect all data up to VBP15-LTE Month 12 for the interim analysis. However, despite best efforts, missing or incomplete data may be reported. All missing or partial data will be presented in the subject data listing, as they are recorded on the eCRF. Data after VBP15-LTE Month 12 visit will not be included in this ISAP.

Subjects lost to follow-up or withdrawn will be included in statistical presentations up to the point of their last evaluation. Unless otherwise specified, in general no imputation of values for missing data will be performed.

##### 7.3.2.1. Handling of Missing Date Values

If the start date/time of an AE is completely missing. The AE will be assumed as TEAE. Otherwise, the following rules may apply.

<u>Partial or Missing Dates/Times</u>

The following conventions will be used to impute missing portions of dates for adverse events. Note that the imputed values outlined here may not always provide the most conservative date. In those circumstances, the imputed value may be replaced by a date that will lead to a more conservative analysis.

A. Start Dates
   1) If the year is unknown, then the date will not be imputed and will be assigned a missing value.
   2) If the month is unknown, then:
      i) If the year matches the first dose date year, then impute the month and day of the first dose date.
      ii) Otherwise, assign 'January.'

3) If the day is unknown, then:
   i) If the month and year match the first dose date month and year, then impute the day of the first dose date.
   ii) Otherwise, assign the first day of the month.

B. Stop Dates
1) If the year is unknown, then the date will not be imputed and will be assigned a missing value.
2) If the month is unknown, then assign 'December.'
3) If the day is unknown, then assign the last day of the month.

For AEs, partial or missing times will be imputed in the following manner:

A. Start Times
1) Day 1:
   i) If hour is missing on the CRF, hour will be imputed as hour of the first dose.
   ii) If minute is missing on the CRF, minute will be imputed as minute of the first dose.
   iii) If hour is missing for both start of the AE and for the time of the first dose on the CRF, hour will be imputed as 23.
   iv) If minute is missing for both start of the AE and for the time of the first dose on the CRF, minute will be imputed as 59.

2) Study days other than Day 1:
   i) If hour is missing on the CRF, hour will be imputed as 00.
   ii) If minute is missing on the CRF, minute will be imputed as 00.

B. Stop Times
1) If hour is missing on the CRF, hour will be imputed as 23.
2) If minute is missing on the CRF, minute will be imputed as 59.

### 7.3.2.2. Imputation Methods

Velocity scores for TTSTAND, TTRW, and TTCLIMB will be imputed as 0 at the first response missing due to disease progression and imputed as 0 for all remaining visits.

All other data will be observed values only, without imputation.

#### 7.3.3. Handling of Early Termination Visit Information

If a subject is terminated early before VBP15-LTE Month 12, the early termination (ET) visit data will be analyzed at the closest scheduled visit. If the closest visit has valid data, the early termination data will be assigned to the next available visit. In instances where the next scheduled visit is too far in the future, from a clinical perspective, analyses may be presented twice, once with the ET observation assigned to the next available visit, and once with the ET observation omitted.

#### 7.3.4. Pooling of Investigational Sites

The data from all study centers will be pooled together for analyses.

#### 7.3.5. Coding Conventions for Events and Medications

All adverse events will be mapped to the Medical Dictionary for Regulatory Activities (MedDRA version 19.0) system for reporting (preferred term and system organ class).

#### 7.3.6. Baseline Visits

For analyses that present demographic and baseline data or change from baseline, Baseline is defined as study VBP15-002 for the LTE subjects. The 3 CINRG cohorts will define Baseline as the first visit the subject meets the matching criteria for this ISAP. baseline.

#### 7.3.7. Analysis Software

Data manipulation, tabulation of descriptive statistics, and graphical representations will be performed primarily using SAS (release 9.4 or higher) for Windows.

### 7.4. Planned Study Analyses

#### 7.4.1. Statistical Summaries: Descriptive and Inferential

All statistical tests will be two-sided and a resultant p-value of less than or equal to 0.05 will be considered statistically significant. All p-values will be rounded to and displayed in four decimals. If a p-value less than 0.0001 occurs, it will be shown in tables as <0.0001.

Descriptive summaries of variables will be provided where appropriate. In general, for continuous variables, the number of non-missing values (n) and the mean, standard deviation, median, minimum, and maximum will be tabulated. For categorical variables, the frequency and percentage will be tabulated.

### 7.5. Multiple Testing Procedures

No adjustments for multiplicity on inferential statistics will be presented in this ISAP.

# 8. MONTH 12 INTERIM ANALYSIS

VBP15-LTE subject data will include up to Month 12 visit providing up to 18 months of on-treatment study data using VBP15-002 Baseline as baseline. CINRG DNHS steroid naïve and steroid treated subject data will include up to Month 18 visit. CINRG prednisone treated subject data will include up to Month 12 visit.

## 8.1. Subject Summary Grouping

VBP15-LTE disposition, demographic and baseline characteristics, strength and functionality tests, height, blood pressure (BP), BMI, and AE data will be presented by grouping dose Cohort 3 and 4 (combined 2.0 and 6.0 mg/kg/day) data.

White cell counts and PD biomarker subject data will be presented by dose subject was on during visit of data collection.

## 8.2. Subject Disposition

The number of subjects enrolled in VBP15-LTE, the number of discontinuations (if any) by VBP15-LTE Month 12, and the reason for discontinuation, will be tabulated. VBP15-LTE Cohort 3 and 4 combined will be presented vs. each of the 3 CINRG cohorts, CINRG DNHS steroid naïve up to Month 18, CINRG DNHS steroid treated up to Month 18, and CINRG prednisone treated up to Month 12.

Subject disposition data will be presented in by-subject listings.

Analysis will be by planned treatment.

## 8.3. Demographics and Baseline Characteristics

Descriptive statistics for age, race, and ethnicity collected at VBP15-002 Baseline study will be tabulated for Cohort 3 and 4 combined using the Safety population vs. baseline data for each of the 3 CINRG cohorts.

Demographic and baseline data will be presented in by-subject listings.

Analysis will be by planned treatment.

## 8.4. TTSTAND, TTRW, TTCLIMB, CQMS, 6MWT, and NSAA

TTSTAND, TTRW, TTCLIMB (raw score and velocity), CQMS, 6MWT, and NSAA composite score observed and change from baseline will be summarized descriptively for VBP-LTE Cohort 3 and 4 (2 and/or 6 mg/kg/day) combined at VBP15-002 Baseline and LTE Month 12 vs. the CINRG DNHS steroid naive cohort at CINRG Baseline and Month 18.

Inferential testing will be presented to compare combined VBP-LTE Cohort 3 and 4 (2 and/or 6 mg/kg/day) to CINRG DNHS steroid naïve on TTSTAND, TTRW, TTCLIMB (raw score and velocity), CQMS, 6MWT, and NSAA composite score mean change from baseline. An analysis of covariance (ANCOVA) model including treatment group, age at baseline, and baseline response will be used to test for a statistical difference in least squares (LS) means across the treatment groups. P-values and 95% confidence intervals for the differences in the LS means will be presented.

TTSTAND, TTRW, and TTCLIMB (raw score and velocity) observed and change from baseline will be summarized descriptively for VBP-LTE Cohort 3 and 4 (2 and/or 6 mg/kg/day) combined at VBP15-002 Baseline and LTE Month 12 vs. DNHS prednisone data at Baseline and Month 12. No inferential statistics will be presented.

Also, observed TTSTAND data will be analyzed using Kaplan-Meier (KM) time-to-event analyses. VBP-LTE Cohort 3 and 4 combined subjects at VBP-LTE Month 18 will be compared to CINRG DNHS steroid naïve subjects at Month 18. Two analyses will be performed; one will include only subjects that have a recorded TTSTAND >5 seconds, and one will include only subjects with a recorded TTSTAND >10 seconds. Estimated time to stand quartiles ($25^{th}$ percentile, median, and $75^{th}$ percentile) along with 95% confidence intervals will be presented along with KM curves.

For TTSTAND, TTCLIMB and TTRW, the following transformation formulas show how to convert raw scores to velocities:

- TTSTAND velocity = 1 / TTSTAND and is expressed as rises/second.
- TTCLIMB velocity = 1 / TTCLIMB and is expressed as tasks/second.
- TTRW velocity = 10 / TTRW and is expressed as meters/second.

TTSTAND, TTRW, and TTCLIMB data will be presented in by-subject listings.

The FAS population will be used for VBP15-LTE subjects and analysis will be by planned treatment.

## 8.5. Adverse Events

Treatment-emergent adverse events (TEAEs) will be defined as any adverse event starting or worsening after VBP15-LTE first dose through the subject's Month 12 visit. TEAEs collected after the Month 12 visit will not be included.

If the onset of an AE is on VBP15-LTE Day 1 and its relationship to time of study drug administration is unknown, then the AE will be counted as treatment-emergent. If the onset of the AE is on Day 1 but is known to have onset prior to the time of the first administration of study drug, the AE will not be considered treatment-emergent.

AEs initiated during VBP15-003 participation which are ongoing at the time of enrollment into VBP15-LTE will be recorded as Medical History in the VBP15-LTE eCRF and will not be presented as TEAEs in the VBP15-LTE study.

The number and percentage of subjects with any TEAEs will be summarized by system organ class (SOC) and preferred term (PT) by combined VBP15-LTE Cohort 3 and 4. At each level of tabulation (e.g., at the preferred term level) subjects will be counted only once if they had more than one such event reported during the AE collection period.

Level of intensity will be assessed using the CTCAE grading.

The following summary tables and subject level listings will be presented for TEAE data:

- Overall summary of TEAEs
- Summary table of TEAEs by SOC and PT
- Summary table of TEAEs by maximum relationship to treatment by SOC and PT
- Summary table of TEAEs by maximum severity by SOC and PT
- Summary table of serious TEAEs by SOC and PT
- Summary table of TEAEs leading to study drug withdrawal by SOC and PT

AE data will be presented in by-subject listings.

The Safety Population will be used for VBP15-LTE subjects and analysis will be by actual treatment.

## 8.6. BMI

BMI absolute and Z-score responses, observed and change from baseline, will be summarized descriptively for VBP-LTE Cohort 3 and 4 (2 and/or 6 mg/kg/day) combined data at VBP15-002 Baseline and LTE Month 12 vs. CINRG prednisone at Baseline and Month 12 and vs. CINRG DNHS steroid naïve data at Baseline and Month 18.

Inferential testing will be presented to compare combined VBP-LTE Cohort 3 and 4 (2 and/or 6 mg/kg/day) to CINRG prednisone and CINRG DNHS steroid naïve cohorts on BMI absolute and Z-score mean change from baseline in separate models. An analysis of covariance (ANCOVA) model including treatment group, age at baseline, and baseline response will be used to test for a statistical difference in least squares (LS) means across the treatment groups. P-values and 95% confidence intervals for the differences in the LS means will be presented.

BMI data will be presented in by-subject listings.

The Safety Population will be used for VBP15-LTE subjects and analysis will be by actual treatment.

See Appendix 11.1 or VBP15-003 protocol Appendix11.1 for a description and example of BMI percentile calculation given BMI score; see Centers for Disease Control and Prevention [CDC] webpage https://www.cdc.gov/growthcharts/percentile_data_files.htm for a detailed discussion on the derivation of the computational algorithm).

### 8.7. Height Percentiles

Height percentile is defined as a function of age and is collected from the Centers for Disease Control Data Table of Stature-for-Age Charts (males ages 2-20 years) https://www.cdc.gov/growthcharts/html_charts/statage.htm#males.

Height percentile observed and change from baseline will be summarized descriptively for VBP-LTE Cohort 3 and 4 (2 and/or 6 mg/kg/day) combined data at VBP15-002 Baseline and LTE Month 12 vs. CINRG prednisone at Baseline and Month 12 and vs. CINRG DNHS steroid treated data at Baseline and Month 18 and vs. CINRG DNHS steroid naïve at Baseline and Month 18..

Inferential testing will be presented to compare combined VBP-LTE Cohort 3 and 4 (2 and/or 6 mg/kg/day) vs CINRG prednisone and vs CINRG DNHS steroid treated and vs. CINRG DNHS steroid naive cohorts on mean change from baseline in height percentile in separate models. An analysis of covariance (ANCOVA) model including treatment group, age at baseline, and baseline response will be used to test for a statistical difference in least squares (LS) means across the treatment groups. P-values and 95% confidence intervals for the differences in the LS means will be presented.

Height percentile data will be presented in by-subject listings.

The Safety Population will be used for VBP15-LTE subjects and analysis will be by actual treatment.

### 8.8. Blood Pressure

Systolic blood pressure (SBP), diastolic blood pressure (DBP) and mean arterial pressure (MAP=DP+1/3 (SP-DP) observed will be summarized descriptively for VBP-LTE Cohort 3 and 4 (2 and/or 6 mg/kg/day) combined at LTE Month 12 vs. CINRG prednisone subjects at Month 12 vs. CINRG DNHS steroid treated subjects at Month 18 vs. CINRG DNHS steroid naive subjects at Month 18.

SBP, DBP, and mean arterial pressure will be presented in by-subject listings.

Descriptive summary, cross-sectional analyses, and test of 18 month-change from baseline by ANCOVA will be performed for all groups.

The Safety Population will be used for VBP15-LTE subjects and analysis will be by actual treatment.

### 8.9. White Cell Count and PD Biomarkers

White blood cell counts, bone biomarkers, first in morning cortisol, and fasting glucose and insulin observed will be summarized descriptively for VBP15-LTE subjects at dose level 0.75, 2.0, and 6.0 mg/kg/day as follows:

- Bone biomarkers (Osteocalcin, CTX1, P1NP) at 6 months and 12 months in VBP15-LTE. Dose at time of measurement: 0.75 vs. 2.0 vs. 6.0 mg/kg/day.

- First in morning cortisol at 9 months. Dose at time of measurements: 0.75 vs. 2.0 vs. 6.0 mg/kg/day. Data will be analyzed as % below threshold of 3.6µg/dL [100 nmol/L] (consistent with adrenal suppression).

- Fasting insulin/glucose at 9 months. Dose at time of measurements: 0.75 vs. 2.0 vs. 6.0 mg/kg/day.

- White cell count differentials (absolute lymphocyte count, absolute neutrophil count, and total white blood cell count) at 12 months. Dose at time of measurements: 0.75 vs. 2.0 vs. 6.0 mg/kg/day.

Note that subjects are presented in the dose group they are on at each visit (i.e., a subject may be summarized within one dose group at one visit and then summarized as part of another dose group at another visit).

White cell counts and PD biomarker data will be presented in by-subject listings.

The Safety Population will be used, and analysis will be by actual treatment.

# 9. REPORTING CONVENTIONS

The following reporting conventions will be adopted for the presentation of study data. These conventions will enhance the review process and help to standardize presentation with common notations

## 9.1. General Reporting Conventions

- All tables will be developed in Landscape Orientation
- Specialized text styles, such as bolding, italics, borders, shading, superscripted and subscripted text will not be used in tables, figures, and data listings unless they add significant value to the table, figure, or data listing.
- Only standard keyboard characters should be used in tables and data listings. Special characters, such as non-printable control characters, printer specific, or font specific characters, will not be used on a table, figure, or data listing. Hexadecimal character representations are allowed (e.g.,μ,α,β).
- All titles will be centered on a page. The ICH numbering convention is to be used for all tables, figures, and data listings.
- All footnotes will be left justified and the bottom of a page. Footnotes must be present on the page where they are first referenced. Footnotes should be used sparingly and must add value to the table, figure, or data listing. If more than four footnote lines are planned then a cover page may be used to display footnotes.
- Missing values for both numeric and character variables will be presented as blanks in a table or data listing. A zero (0) may be used if appropriate to identify when the frequency of a variable is not observed.
- All date values will be presented as YYYY-MM-DD (e.g., 2013-05-17) ISO 8601 format.
- All observed time values will be presented using a 24-hour clock HH:MM:SS format (e.g., 01:35:45 or 11:26). Seconds should only be reported if they were measured as part of the study, also in ISO 8601 format.
- Time durations will be reported in mixed hh mm ss notation (e.g., 5h 32m, or 27h 52m 31s). The use of decimal notation to present (display) time durations should be avoided (e.g. 0.083h = 5m) unless it is necessary to show the computation of time differences in a table, figure, or data listing, in which case both notations may be used to display the time duration.
- All tables will have the Table status (DRAFT, FINAL), and a date/time stamp on the bottom of each output.
- All analysis programs developed for a table display will be self-contained to

facilitate transfer of programs to multiple computing environments and transfer to a regulatory agency (if requested).

# 10. POPULATION SUMMARY CONVENTIONS

- Population(s) represented on the tables or data listings will be clearly identified in the last title of the Table as “Population: <name of population>” and will be identical in name to that identified in the protocol or SAP.
- Consistent terminology will be used to define and identify a population.
- Sub-population(s) or special population(s) descriptions will provide sufficient detail to ensure comprehension of the population (e.g., FAS Females, Per-Protocol Males >60 years of age) used for analysis in a table or figure.
- Population sizes may be presented for each treatment or dosing category as totals in the column header as (N=xxxx), where appropriate.
- Population sizes shown with summary statistics are the samples sizes (n) of subjects with non-missing values.
- All population summaries for categorical variables will include all categories that were planned and for which the subjects may have had a response. Percentages corresponding to null categories (cells) will be suppressed.
- All population summaries for continuous variables will include: N, mean, SD, median, minimum, and maximum. Other summaries (e.g. number missing, quartiles, 5%, 95% intervals, CV or %CV) may be used as appropriate.
- All percentages are rounded and reported to xx.x%. A percentage of 100% will be reported as 100%. For categorical summaries presenting “n (%)”, a count of 0 will be presented as “0”. For continuous results, an estimated % of 0 will be presented as “0%”.
- Population summaries that include p-values will report the p-value to four decimal places with a leading zero (e.g., 0.0001). All p-values reported on default output from statistical software (i.e., SAS® Software version 9.2 or later) may be reported at the default level of precision. P-values <0.0001 should be reported as <0.0001 not 0.0000.

# 11 REFERENCES

Bello L, Gordish-Dressman H, Morgenroth LP, Henricson EK, Duong T, Hoffman EP, Cnaan A, McDonald CM; CINRG Investigators. Prednisone/prednisolone and deflazacort regimens in the CINRG Duchenne Natural History Study. Neurology. 2015 Sep 22;85(12):1048-55. doi: 10.1212/WNL.0000000000001950. Epub 2015 Aug 26. PubMed PMID: 26311750; PubMed Central PMCID: PMC4603595.

Bello L, Morgenroth LP, Gordish-Dressman H, Hoffman EP, McDonald CM, Cirak S; CINRG investigators. DMD genotypes and loss of ambulation in the CINRG Duchenne Natural History Study. Neurology. 2016 Jul 26;87(4):401-9. doi: 10.1212/WNL.0000000000002891. Epub 2016 Jun 24. PubMed PMID: 27343068; PubMed Central PMCID: PMC4977110.

Bello L, Flanigan KM, Weiss RB; United Dystrophinopathy Project, Spitali P,Aartsma-Rus A, Muntoni F, Zaharieva I, Ferlini A, Mercuri E, Tuffery-Giraud S, Claustres M, Straub V, Lochmüller H, Barp A, Vianello S, Pegoraro E, Punetha J, Gordish-Dressman H, Giri M, McDonald CM, Hoffman EP; Cooperative International Neuromuscular Research Group. Association Study of Exon Variants in the NF-κB and TGFβ Pathways Identifies CD40 as a Modifier of Duchenne Muscular Dystrophy. Am J Hum Genet. 2016 Nov 3;99(5):1163-1171. doi: 10.1016/j.ajhg.2016.08.023. Epub 2016 Oct 13. PubMed PMID: 27745838; PubMed Central PMCID: PMC5097949.

Brookmeyer R Crowley J. A confidence interval for the median survival time, Biometrics 1982;38:29-41.

Clopper CJ, Pearson ES The use of confidence or fiducial limits illustrated in the case of the binomial. Biometrika 1934; 26(4):404-13.

Escolar DM, Henricson EK, Pasquali L, Gorni K, Hoffman EP. Collaborative translational research leading to multicenter clinical trials in Duchenne muscular dystrophy: the Cooperative International Neuromuscular Research Group (CINRG). Neuromuscul Disord. 2002 Oct;12 Suppl 1:S147-154.

Escolar DM, Hache LP, Clemens PR, Cnaan A, McDonald CM, Viswanathan V, Kornberg AJ, Bertorini TE, Nevo Y, Lotze T, Pestronk A, Ryan MM, Monasterio E, Day JW, Zimmerman A, Arrieta A, Henricson E, Mayhew J, Florence J, Hu F, Connolly AM. Randomized, blinded trial of weekend vs daily prednisone in Duchenne muscular dystrophy. Neurology. 2011 Aug 2;77(5):444-52.

ICH E9 Expert Working Group. Statistical Principles for Clinical Trials: ICH Harmonized Tripartite Guideline, September 1998

Kalbfleisch JD, Prentise RL. The Statistical Analysis of Failure Time Data. New York: John Wiley & Sons, Inc. 1980.

McDonald CM, Henricson EK, Abresch RT, Duong T, Joyce NC, Hu F, Clemens PR, Hoffman EP, Cnaan A, Gordish-Dressman H; CINRG Investigators. Long-term effects of glucocorticoids on function, quality of life, and survival in subjects with Duchenne muscular dystrophy: a prospective cohort study. Lancet. 2018 Feb 3;391(10119):451-461.

### 11.1 Calculating BMI Z-Scores

The following example for computing BMI Z-scores given age and sex for children aged 2 to 20 years uses the computational algorithm presented on the Centers for Disease Control and Prevention (CDC) webpage "Percentile Data Files with LMS Values". For a detailed discussion on the derivation of the computational algorithm and reference materials, visit the webpage at https://www.cdc.gov/growthcharts/percentile_data_files.htm.

To obtain the Z-score (Z) for a given BMI measurement X, use the following equation:

$Z= [((X/M)^{\wedge L}) - 1] / (LS)$, where $L \neq 0$

or

$Z = \ln(X/M)/S$, where $L=0$

where L, M, and S are the values from the BMIAGE.xls reference table (growth chart 8 linked to on the aforementioned CDC webpage).

For example, for a 24 month old male (coded sex value = 1) who has a BMI of 17.2864, the BMIAGE.xls reference table presents values of L=-2.01118, M=16.57503, and S=0.080592. Plugging those parameter values into the Z formula above results in a Z-score of 0.5.

### 11.2 ISAP Amendment Summary of Changes

| Page Number | Section | Description of Change |
|---|---|---|
| | | |
| | | |
| | | |
