# Supplementary material for: Efficacy and safety of vamorolone in Duchenne muscular dystrophy: An 18-month interim analysis of a non-randomized open-label extension study
Source: PLoS Med. 2020 Sep 21;17(9):e1003222. doi: 10.1371/journal.pmed.1003222 (PMC7505441; doi:10.1371/journal.pmed.1003222)
Supplement: S1 Protocol — Amendment #1 for a 24-month Phase II open-label, multicenter long-term extension study to assess the long-term safety and efficacy of vamorolone in boys with Duchenne muscular dystrophy (DMD). (PDF) [file pmed.1003222.s004.pdf]

Altered from the original to remove "confidential"

CLINICAL STUDY PROTOCOL

Amendment #1

**Document Title:**	**Amendment #1 for a 24-month Phase II Open-label, Multicenter Long-term Extension Study to Assess the Long-term Safety and Efficacy of Vamorolone in Boys with Duchenne Muscular Dystrophy (DMD)**

Protocol Number: VBP15-LTE

Document Number: VBP15-LTE-A1

FDA IND No.: 118,942

Investigational Product: Vamorolone (VBP15)

Sponsor: ReveraGen BioPharma, Inc.
155 Gibbs Street
Suite 433
Rockville, MD. 20850

Study Chair: Paula R. Clemens, M.D.
Phone: (412) 383-7207
Email: clempr@upmc.edu

Medical Monitor: Benjamin D. Schwartz, M.D., Ph.D.
Phone: (314) 220-7067
Email: bdschw@camdengroup.com

Document Date: 03 October 2018

[redacted]

SIGNATURES OF AGREEMENT FOR VBP15-LTE-A1

Amendment #1 for a 24-month Phase II Open-label, Multicenter Extension Study to Assess the Long-term Safety and Efficacy of Vamorolone in Boys with Duchenne Muscular Dystrophy (DMD)

Reviewed and Approved by:

4 October 2018

Eric Hoffman, Ph.D.
Chief Executive Officer
ReveraGen BioPharma, Inc.

Date

03 OCT 2018

Paula R. Clemens, M.D.
Study Chair
University of Pittsburgh School of Medicine

Date

03 October 2018

Benjamin D. Schwartz, M.D., Ph.D.
Consulting Medical Monitor
The Camden Group, LLC

Date

03 October 2018

Laurel J. Mengle-Gaw, Ph.D.
Consulting Clinical Monitor
The Camden Group, LLC

Date

04 OCT 2018

Mark J. Jaros, Ph.D.
Consulting Statistician
Summit Analytical, LLC

Date

INVESTIGATOR PROTOCOL AGREEMENT

Amendment #1 for a 24-month Phase II Open-label, Multicenter Long-term Extension Study to Assess the Long-term Safety and Efficacy of Vamorolone in Boys with Duchenne Muscular Dystrophy (DMD)

**Protocol Number:**	**VBP15-LTE**
**Document Number:**	**VBP15-LTE-A1**
**Sponsor:**	**ReveraGen BioPharma, Inc.**
**Document Date:**	**03 October 2018**

By my signature, I confirm that my staff and I have carefully read and understand this protocol, protocol amendment, amended protocol, or revised protocol and agree to comply with the conduct and terms of the study specified herein and with any other study conduct procedures provided by ReveraGen BioPharma, Inc.

I agree to conduct the study according to this protocol and the obligations and requirements of clinical Investigators and all other requirements listed in 21 CFR part 312 and all applicable local, state, and federal regulations and ICH guidelines. I will not initiate this study without the approval of an Institutional Review Board (IRB) or Independent Ethics Committee (IEC).

I understand that, should the decision be made by ReveraGen BioPharma, Inc. to terminate prematurely or suspend the study at any time for whatever reason, such decision will be communicated to me in writing. Conversely, should I decide to withdraw from execution of the study, I will communicate immediately such decision in writing to ReveraGen BioPharma, Inc.

For protocol amendments and amended protocols, I agree not to implement the amendment without agreement from ReveraGen BioPharma, Inc. and prior submission to and written approval (where required) from the IRB/IEC, except when necessary to eliminate an immediate hazard to the subjects, or for administrative aspects of the study (where permitted by all applicable regulatory requirements).

______________________________________ __________________
Investigator's Signature Date

____
Investigator's Name (Please print)
Address (Please print):

RETAIN THE ORIGINAL SIGNED AGREEMENT AT YOUR SITE AND RETURN AN ELECTRONIC SIGNED COPYTO REVERAGEN BIOPHARMA, INC. OR DESIGNEE

SERIOUS ADVERSE EVENT CONTACT INFORMATION

In the event of a serious adverse event (SAE) (see Section 7.2.6.1), the Investigator will complete the SAE electronic case report form within 24 hours of first awareness of the event. In the unlikely event that the electronic study database is inaccessible and the Investigator is unable to complete the SAE electronic case report form within 24 hours, the SAE Notification Form (pdf) should be completed and emailed or printed/faxed to the PRA safety management team within 24 hours, using the contact information below:

Email CHOSafety@prahs.com
Drug Safety Fax: 1-888-772-6919 or 1-434-951-3482

SAE Questions: Drug Safety Hotline: 1-800-772-2215 or 1-434-951-3489

PROTOCOL AMENDMENT TRACKING

Document	Document Number	Approval Date
Original Protocol	VBP15-LTE	11 January 2017
Amendment #1	VBP15-LTE-A1	03 October 2018

Reasons for Protocol Amendment #1:

1. To update Section 1 Introduction with safety results from Phase II studies in DMD boys (VBP15-002 and VBP15-003);
2. To update Section 1.5 Overall Benefit/Risk;
3. To update Section 13 References;
4. To clarify that dose may be escalated incrementally to 6.0 mg/kg/day;
5. To clarify the circumstances under which study drug dose should be interrupted, de-escalated, or discontinued;
6. To allow dose de-escalation from 6.0 mg/kg/day to 2.0 mg/kg/day to be followed by dose escalation to 4.0 mg/kg/day, balancing efficacy and safety concerns, in the opinion of the Investigator;
7. To prohibit use of Exondys 51, Translarna, and other medications indicated for treatment of DMD during the study;
8. To add details of Data and Safety Monitoring Board (DSMB) responsibilities;
9. To add collection of 8.5 mL of blood at the Month 24 Visit for deoxyribonucleic acid (DNA) testing for established genetic modifiers of DMD;
10. To add spine x-ray for detection of fracture at the Month 24 Visit;
11. To add hand x-ray for assessment of bone age at the Month 24 Visit;
12. To remove the designation of BMI z-score as the primary safety outcome;
13. To update the primary efficacy endpoint;
14. To remove comparison of vamorolone with prednisone-treated historical control data for serum PD biomarkers of safety;
15. To add comparison of vamorolone to prednisone- and deflazacort-treated historical control data for bone age, spine fracture, and height z-score, and comparison to deflazacort-treated historical control data for BMI z-score;
16. To update the statistical methodology and composition of the control populations;
17. To clarify that collection of the Pediatric Outcomes Data Collection Instrument (PODCI) does not need to be repeated at the VBP15-LTE Baseline Visit if the Baseline Visit occurs ≤ 28 days after the date of the VBP15-003 Week 24 Visit;
18. To clarify the PD biomarkers to be evaluated;
19. To remove the designation of methodologies to be used for measurement of exploratory PD biomarkers;
20. To clarify the exploratory endpoint for PODCI assessment;
21. To clarify that vital signs, height, and clinical laboratory test results are included in safety data; and
22. To correct typographical errors.

The Sections changed by amendment are listed in was/is format in **Appendix 14.2**.

STUDY SYNOPSIS

**Protocol Title**	A 24-month Phase II Open-label, Multicenter, Long-Term Extension Study to Assess the Long-term Safety and Efficacy of Vamorolone in Boys with Duchenne Muscular Dystrophy (DMD)
**Protocol Number**	VBP15-LTE
**Name of Sponsor**	ReveraGen BioPharma, Inc.
**Drug Substance**	delta-1,4,9(11)-pregnatriene-17-alpha,21-dihydroxy-16-alpha-methyl-3,20-dione
**Investigational Drug Product**	Vamorolone, 4% suspension for oral dosing
**Phase of Development**	Phase II
**Indication**	Treatment of Duchenne muscular dystrophy (DMD)
**Objectives**	***Primary:***<br>1. To evaluate the long-term safety and tolerability of vamorolone, administered orally at daily doses up to 6.0 mg/kg over a 24-month Treatment Period, in young boys with DMD who completed protocol VBP15-003; and<br>2. To compare the efficacy, as measured by the Time to Stand Test (TTSTAND), of vamorolone administered orally at daily doses up to 6.0 mg/kg over a 24-month Treatment Period vs. untreated DMD historical controls in young boys with DMD.<br>***Secondary:***<br>1. To investigate the effects of vamorolone, administered orally at daily doses up to 6.0 mg/kg over a 24-month Treatment Period on serum pharmacodynamic (PD) biomarkers of safety (insulin resistance, adrenal axis suppression, and bone turnover);<br>2. To investigate the effects of vamorolone, administered orally at daily doses up to 6.0 mg/kg over a 24-month Treatment Period, on muscle strength, mobility and functional exercise capacity vs. untreated DMD historical controls as measured by Time to Run/Walk Test (TTRW), North Star Ambulatory Assessment (NSAA), Time to Climb Test (TTCLIMB), 6-minute Walk Test (6MWT), and Quantitative Muscle Testing (QMT) in young boys with DMD; and<br>3. To compare the safety, as assessed by bone age, spine fractures, BMI z-score, and height z-score, of vamorolone administered orally at daily doses up to 6.0 mg/kg over a 24-month Treatment Period vs. prednisone- and deflazacort-treated historical control boys with DMD.<br>***Exploratory:***<br>1. To investigate the effects of vamorolone administered orally at daily doses up to 6.0 mg/kg over a 24-month Treatment Period on Quality of Life measures (Pediatric Outcomes Data Collection Instrument [PODCI]);<br>2. To investigate the effects of vamorolone administered orally at daily doses up to 6.0 mg/kg over a 24-month Treatment Period on additional exploratory PD biomarkers; and

	3. To determine if established genetic modifiers of DMD (gene polymorphisms associated with disease severity, or response to glucocorticoid treatment) are similarly associated with vamorolone-treated DMD patients (baseline disease severity, or response to vamorolone treatment).
**Study Design**	This long-term extension (LTE) study is an open-label, multicenter study to evaluate the long-term safety, tolerability, clinical efficacy, and PD of vamorolone at dose levels up to 6.0 mg/kg administered daily by liquid oral suspension over a Treatment Period of 24 months to young boys with DMD.<br>Subjects who have completed the Phase IIa extension VBP15-003 Study Week 24 Follow-up assessments prior to enrollment are eligible for enrollment into the VBP15-LTE study.<br>The LTE study is comprised of a Pretreatment Baseline Period of up to 24 hours, which begins with the signing of the VBP15-LTE-specific informed consent form (ICF), a 24-month Treatment Period, and an up to 5-week Dose-tapering Period for subjects who elect to transition off vamorolone treatment at the end of the study.<br>Subjects are enrolled at the time of the Informed Consent Form (ICF) signing. Parents or guardians of eligible subjects will provide written informed consent for VBP15-LTE long-term extension study participation. The assessments collected at the final VBP15-003 Week 24 Visit will serve as the Baseline assessments for the VBP15-LTE study.
**Planned Sample Size**	Up to approximately 50 subjects who complete the VBP15-003 study will be enrolled.
**Population**	**Inclusion Criteria:**<br>1. Subject's parent or legal guardian has provided written informed consent and HIPAA authorization (if applicable) prior to any VBP15-LTE long-term extension study-specific procedures;<br>2. Subject has previously completed study VBP15-003 up to and including the Week 24 Final assessments, prior to enrolling in the VBP15-LTE study at the conclusion of the VBP15-003 Week 24 Visit [Note: if entering the dose-tapering period, subject is enrolling within 8 weeks after the VBP15-003 final visit following dose-tapering]; and<br>3. Subject and parent/guardian are willing and able to comply with scheduled visits, study drug administration plan, and study procedures.<br>**Exclusion Criteria:**<br>1. Subject had a serious or severe adverse event in study VBP15-003 that, in the opinion of the Investigator, was probably or definitely related to vamorolone use and precludes safe use of vamorolone for the subject in this long-term extension study;<br>2. Subject has current or history of major renal or hepatic impairment, diabetes mellitus or immunosuppression;<br>3. Subject has current or history of chronic systemic fungal or viral infections;<br>4. Subject has used mineralocorticoid receptor agents, such as spironolactone, eplerenone, canrenone (canrenoate potassium), prorenone (prorenoate potassium), mexrenone (mexrenoate potassium) within 4 weeks prior to the first dose of study medication;<br>5. Subject has evidence of symptomatic cardiomyopathy. [Note: Asymptomatic cardiac abnormality on investigation would not be exclusionary];

	6. Subject is currently being treated or has received previous treatment with oral glucocorticoids or other immunosuppressive agents [Notes: Past transient use of oral glucocorticoids or other oral immunosuppressive agents for no longer than 3 months cumulative, with last use at least 3 months prior to first dose of study medication, will be considered for eligibility on a case-by-case basis. Inhaled and/or topical glucocorticoids prescribed for an indication other than DMD are permitted but must be administered at stable dose for at least 3 months prior to study drug administration];<br>7. Subject has used idebenone within 4 weeks prior to the first dose of study medication;<br>8. Subject has an allergy or hypersensitivity to the study medication or to any of its constituents;<br>9. Subject has severe behavioral or cognitive problems that preclude participation in the study, in the opinion of the Investigator;<br>10. Subject has previous or ongoing medical condition, medical history, physical findings or laboratory abnormalities that could affect safety, make it unlikely that treatment and follow-up will be correctly completed or impair the assessment of study results, in the opinion of the Investigator<br>11. Subject is currently taking any investigational drug, or has taken any investigational drug other than vamorolone within 3 months prior to the start of study treatment.<br>Note: Subjects may be re-evaluated if ineligible due to a transient condition which would prevent the subject from participating.
**Number of Centers**	The study will be conducted at approximately 15 United States (US) and non-US study sites.
**Study Period**	First subject enrolled: 1Q 2017<br>Last subject last visit: 2Q 2020
**Study Duration**	Up to approximately 4 years total duration
**Individual Subject Study Duration**	Approximately 24-25 months:<br>• Baseline Period: up to 24 hours immediately prior to administration of first dose of study medication in VBP15-LTE long-term extension study<br>• Treatment Period: 24 months<br>• Dose-tapering Period: up to 5 weeks<br>• Subjects may leave the study at their own discretion at any time. Subjects who discontinue vamorolone will follow the Dose-tapering Period procedures.
**Study Drug Formulation, Dosage & Administration**	Vamorolone 4% oral suspension will be administered once daily over a 24-month Treatment Period, at one of the following planned dose levels investigated in VBP15-003: 0.25 mg/kg/day, 0.75 mg/kg/day, 2.0 mg/kg/day, or 6.0 mg/kg/day with a glass of full fat milk (approximately 8 grams fat or equivalent).<br>Subjects will receive vamorolone throughout the 24-month Treatment Period, initially at the same dose level they received at the time they completed the Week 24 Final Visit of the VBP15-003 Phase IIa extension study. Subjects, after they have been on their initial dose for at least 1 month in VBP15-LTE may be escalated to the next higher dose level at the discretion of the Study Chair and Medical Monitor once the next higher dose has been determined to be safe in the VBP15-002 Phase IIa Study, and no safety issues with that dose have

	emerged in the VBP15-003 Phase IIa extension study. (See procedure detailed below on how to escalate to the next dose level).<br>Dosing is to occur at home throughout the 24-month Treatment Period, except at the Month 9 and Month 21 Visits when dosing will occur at the study site to accommodate morning fasted blood collections. Study drug will be administered by mouth using a volumetric syringe. Following administration of the dose of study drug, the syringe will be filled once with water and the water will be administered by mouth using the volumetric syringe. The subject will then drink approximately 50 mL (approximately 2 ounces) of water to ensure the full dose has been ingested.<br>The daily dose of study medication should be taken with a glass of full fat milk (approximately 8 grams of fat) or food with similar fat content of the subject's choice. There are no additional food or drink restrictions before or after dosing.<br>**Procedure to escalate to the next higher dose:** Dose escalation within this study will be considered at the discretion of the Study Director and Medical Monitor. Subjects, after they have been on their initial dose for at least 1 month in VBP15-LTE, may be escalated to the next higher dose level once the next higher dose has been determined to be safe in the VBP15-002 Phase IIa Study, and no safety issues with that dose have emerged in the VBP15-003 Phase IIa extension study. Dose may be escalated in no greater than 3 increments, not to exceed the MTD identified in the VBP15-002 dose finding study and confirmed in the VBP15-003 Phase IIa extension study.
**Study Summary**	This long-term extension study is an open-label, multiple-dose study to evaluate the long-term safety, tolerability, efficacy and PD of vamorolone administered once daily by liquid oral suspension over a Treatment Period of 24 months to young boys with DMD who participated in the VBP15-002 Phase IIa and VBP15-003 Phase IIa extension core studies.<br>Only subjects who have completed the VBP15-003 Phase IIa extension study Week 24 Final Visit assessments will be eligible for participation in this open-label VBP15-LTE study. Participation in this LTE study will be discussed with the subject's parent or guardian prior to the VBP15-003 Week 24 Visit. Standard of care treatment (glucocorticoids) for DMD should be discussed with the subject's parent/guardian prior to the decision to enroll the subject in the LTE study. Standard of care glucocorticoids should be offered to the subject following completion of the VBP15-003 Phase IIa extension study, if the subject's parent or guardian does not wish to enroll the subject in this long-term extension study and/or the Investigator feels it to be in the best interest of the subject. A total of up to approximately 50 subjects will be enrolled into this long-term extension study.<br>The parents or legal guardians of subjects who choose to enroll in this long-term extension study will give written informed consent for the long-term extension study at the VBP15-LTE Baseline Day -1 Visit, which may coincide with the conclusion of the VBP15-003 Week 24 Visit following all assessments in the trial, or may occur up to 8 weeks following the final visit of the Dose-tapering Period for subjects who dose-taper. Subjects are considered to be enrolled in the VBP15-LTE long-term extension study after the parent or guardian has signed the VBP15-LTE-specific ICF at the Baseline Visit. Each subject will retain the study identification number assigned to him at the start of the Phase IIa core study VBP15-002 and used for the Phase IIa extension study VBP15-003.<br>Subjects who participated in the VBP15-003 study will begin dosing in this study on Study Day 1 at the same vamorolone dose level they received at the time of the Week 24 Final Visit in the VBP15-003 Phase IIa extension study.

	Subjects, after they have been on their initial dose for at least 1 month in VBP15-LTE, may be escalated to the next higher dose level at the discretion of the Study Chair and Medical Monitor once the next higher dose has been determined to be safe in the VBP15-002 Phase IIa Study, and no safety issues with that dose have emerged in the VBP15-003 Phase IIa extension study.<br><br>The planned dose levels in the VBP15-003 study are 0.25 mg /kg (Dose Level 1), 0.75 mg/kg (Dose Level 2), 2.0 mg/kg (Dose Level 3), and 6.0 mg/kg (Dose Level 4).<br><br>If dose limiting toxicities are identified in the VBP15-002 or VBP15-003 studies which preclude confirmation of safety of planned dose levels, the dose levels in this long-term extension study will be modified, as needed.<br><br>In the event any clinical observation suggests an intolerability for an individual subject to the study medication, in the opinion of the Investigator, the subject's dose level may be decreased to the next lower dose level and maintained at that lower dose level throughout the duration of the Treatment Period, with the caveat stated below for subjects de-escalating from 6.0 mg/kg/day to 2.0 mg/kg/day. In the event the next lower dose level is also not tolerated and is considered a safety risk to the subject, in the opinion of the Investigator, Study Chair, and Medical Monitor, the subject will be withdrawn from the study. Subjects whose dose is decreased from 6.0 mg/kg/day to 2.0 mg/kg/day may have their dose subsequently increased to 4.0 mg/kg/day if they have been taking the 2.0 mg/kg/day dose for at least one month and, in the opinion of the Investigator, balancing efficacy with safety concerns, they could benefit from an intermediate higher dose.<br><br>Subjects will be assessed for safety and tolerability, clinical efficacy, pharmacodynamic biomarkers, and quality of life at scheduled visits throughout the 24-month Treatment Period. Treatment Period study visits for VBP15-LTE will occur at Month 1, Month 3 and then at 3-month intervals extending through Month 24. Adverse events (AEs), including serious adverse events (SAEs), and concomitant medications will be recorded throughout the study.<br><br>Once-daily Treatment Period study drug dosing will occur at home on all days throughout the 24-month Treatment Period, except at the Month 9 and Month 21 Visits when dosing will occur at the study site to accommodate morning fasted blood collections.<br><br>Subject diaries will be dispensed at Baseline Day -1 and at each 3-monthly study visit to record AEs and any changes to concomitant medications taken during the study.<br><br>All subjects will return to the clinical site for Month 24 assessments. Following completion of the 24-month Treatment Period, subjects may be offered further extended treatment with vamorolone in a separate extension study, or switch to standard of care treatment (e.g., glucocorticoids) for DMD, as deemed appropriate for each subject. Alternatively, subjects may choose to discontinue vamorolone and not begin standard of care glucocorticoid treatment for DMD. Subjects who switch to standard of care glucocorticoids for DMD or who discontinue treatment will participate in a Dose-tapering Period of up to 5 weeks in duration, following the end of the Treatment Period and prior to discharge from the study; subjects who elect to receive further vamorolone treatment will be discharged from the VBP-LTE study after completion of final VBP-LTE study assessments at the Month 24 Visit, prior to enrollment in the separate extension study. Subjects will be discharged from the study following completion of all final Month 24 or Dose-tapering Period assessments, as appropriate.

	In the event that any clinical or laboratory parameters remain abnormal at the time of discharge from the study, the subject will be followed medically as clinically indicated.<br>A subject may decide to discontinue vamorolone treatment at any time. If the subject discontinues vamorolone, then the up-to-5-week dose-tapering schedule should be followed.
**Safety Measures**	• Physical examination<br>• Weight<br>• Height<br>• Body Mass Index (BMI)<br>• Vital signs (supine blood pressure, heart rate, respiratory rate, oral temperature)<br>• Clinical laboratory tests<br>o Hematology and biochemistry<br>o Urinalysis (by dipstick and microscopic analysis)<br>o Lipid profile (triglycerides, total cholesterol, low density lipoprotein [LDL], high density lipoprotein [HDL])<br>• 12-lead electrocardiogram (ECG)<br>• Hand x-ray<br>• Spine x-ray<br>• Clinical signs and symptoms (AEs and SAEs)<br>• Grading of clinical and clinical laboratory AEs will be according to the Common Terminology Criteria for Adverse Events (CTCAE), v4.0.3
**Pharmacodynamic Measures**	• Blood will be drawn for a serum PD biomarker panel at scheduled visits during the Treatment Period and at the end of the Dose-tapering Period, if applicable, to explore the effect of vamorolone on biomarkers of adrenal axis suppression, bone turnover, insulin resistance, and exploratory biomarkers of efficacy and safety.
**Clinical Efficacy Measures**	• Time to Stand Test (TTSTAND)<br>• Time to Climb Test (TTCLIMB)<br>• Time to Run/Walk Test (TTRW)<br>• North Star Ambulatory Assessment (NSAA)<br>• Six-minute Walk Test (6MWT)<br>• Quantitative Muscle Testing (QMT)
**Exploratory Measures**	• Measures of serum PD biomarkers<br>• Quality of life (PODCI)<br>• DNA testing for established genetic modifiers of DMD
**Statistical Methods**	**Analysis Populations:**<br>Four populations will be defined for data analysis: the Safety Population, the Full Analysis Set, the control population CINRG Duchenne Natural History Study, and the control population FOR DMD Study.<br><u>Safety Population</u><br>All subjects who receive at least one dose of vamorolone study medication in the VBP15-LTE extension study will be included in the Safety Population. The Safety Population is the primary analysis population for safety assessments. This is also the modified Intention to Treat (mITT) population.<br><u>Full Analysis Set (FAS)</u><br>All subjects who receive at least one dose of vamorolone study medication in the VBP15-LTE extension study and have at least one post-baseline assessment will be included in the FAS. The FAS is the primary analysis population for clinical efficacy and PD assessments. The FAS population is the mITT

	population, with the additional requirement of having at least one post-baseline assessment. Subjects who receive at least one dose of vamorolone but never have post-baseline assessments will be excluded.<br><br>Control Population from CINRG Duchenne Natural History Study<br>The control population from the CINRG Duchenne Natural History Study will include all subjects who were observed as part of the study in ages ≥ 4 years and <7 years of age at a start of an interval of observation; observed for at least two years with TTSTAND, TTCLIMB, TTRW, NSAA, 6MWT and QMT measured; remained glucocorticoid-naive during the entire observation period; and were able to walk independently without assistive devices, able to complete the TTSTAND; and lacked any history of disease, impairment, or medications that would have made them ineligible to receive the vamorolone intervention as defined by the VBP15-LTE exclusion criteria at the start of the interval.<br><br>Control Population FOR DMD Study<br>The control population from the FOR DMD Study will include all subjects who were 4-7 years old at entry and who were randomized to the daily prednisone or daily deflazacort arm.<br><br>**General Statistical Considerations:**<br><br>All measurements will be analyzed based upon the type of distribution and descriptive statistics presented by treatment group and time point, as appropriate. No interim statistical analyses are planned. Missing values for safety and exploratory outcomes will be treated as missing.<br><br>All statistical tests will be performed at the 0.05 level. Paired t-tests will be utilized for select variables. When available, baseline from the VBP15-002 will be utilized when calculating change from baseline.
	**Efficacy Analyses:**<br><br>The primary efficacy outcome is TTSTAND (velocity). Secondary efficacy outcomes are the NSAA assessment, TTCLIMB, TTRW, QMT, and the 6MWT. TTSTAND, TTCLIMB, and TTRW will be analyzed using raw scores and velocity.<br><br>The primary outcome is TTSTAND (velocity) change from VBP15-002 study baseline to Month 24 and will be compared between vamorolone and historical untreated controls using a restricted maximum likelihood (REML)-based mixed model for repeated measures (MMRM). This model will include fixed effects for treatment, visit, baseline TTSTAND velocity (from the VBP15-002 study), age at study entry, and the treatment-by-visit interaction. The initial model will compare combined vamorolone doses to untreated natural history controls and subsequent secondary models may compare individual and other combinations of dose levels to untreated natural history controls. The secondary outcome measures will be compared using similar models. Full details will be provided in the Statistical Analysis Plan (SAP).<br><br>**Pharmacodynamics Analyses:**<br><br>Serum PD biomarkers of adrenal axis suppression, insulin resistance, and bone turnover will be assessed, as well as exploratory biomarkers of safety and efficacy. Biomarkers will be summarized descriptively over time.

	**Safety Analyses:**<br>All subjects who received at least one dose of vamorolone (Safety Population) will be included in the safety analyses. In general, descriptive statistics for each safety endpoint will be presented by the combined vamorolone dose level, individual dose levels, and other combinations of dose levels.<br>Safety data will include BMI (raw units and z-score), height (raw units and z-score), vital signs, clinical laboratory test results, hand x-ray, spine x-ray, and ECG results.<br>Changes in BMI z-score and height z-score will be analyzed using the same type of statistical models used for efficacy with vamorolone being compared with the daily prednisone and the daily deflazacort arms from the FOR DMD study. Hand x-ray and spine x-ray results will also be compared with the daily prednisone and the daily deflazacort arms from the FOR DMD study.<br>Adverse events will be summarized overall and by dose level by system organ class (SOC) and preferred term (using the Medical Dictionary for Regulatory Activities [MedDRA]); by relationship to study medication; and by intensity (CTCAE grade).

TABLE OF CONTENTS

PROTOCOL AMENDMENT TRACKING....5

STUDY SYNOPSIS 6

LIST OF TABLES 18

LIST OF FIGURES 19

LIST OF ABBREVIATIONS 20

1 INTRODUCTION....24

1.1 BACKGROUND AND UNMET NEED....24

1.2 NONCLINICAL EXPERIENCE....26

1.2.1 Safety Pharmacology 26

1.2.2 Pharmacokinetics and Metabolism 28

1.2.3 Toxicology 32

1.3 CLINICAL EXPERIENCE....40

1.3.1 Phase I Study in Healthy Adult Volunteers 40

1.3.2 Pharmacokinetics in Phase II Study in 4 to 7 years Duchenne Muscular Dystrophy Boys (VBP15-002)....55

1.3.3 Safety in Phase II Studies in 4 to7 years Duchenne Muscular Dystrophy Boys (VBP15-002 and VBP15-003)....57

1.4 RATIONALE FOR STUDY DESIGN....59

1.5 OVERALL BENEFIT/RISK 62

2 STUDY OBJECTIVES AND ENDPOINTS....63

2.1 STUDY OBJECTIVES....63

2.1.1 Primary Objectives....63

2.1.2 Secondary Objectives 63

2.1.3 Exploratory Objectives....64

2.2 STUDY ENDPOINTS 65

2.2.1 Safety Endpoints 65

2.2.2 Clinical Efficacy Endpoints....66

2.2.3 Pharmacodynamic Endpoints....67

2.2.4 Exploratory Endpoints....67

2.2.5 Endpoints for Subject Reported Outcomes 67

3 STUDY DESIGN....67

3.1 OVERALL STUDY DESIGN 67

3.2 STUDY SUMMARY 70

4 SELECTION AND WITHDRAWAL OF STUDY SUBJECTS......................70
4.1 SUBJECT SCREENING, ENROLLMENT, AND IDENTIFICATION LOG......................70
4.2 INCLUSION CRITERIA......................71
4.3 EXCLUSION CRITERIA......................71
4.4 WITHDRAWAL OF SUBJECTS FROM STUDY......................73
4.5 REPLACEMENT OF WITHDRAWN SUBJECTS......................74
4.6 TERMINATION OF STUDY......................74
5 TREATMENT OF STUDY SUBJECTS......................74
5.1 STUDY MEDICATION ADMINISTERED......................74
5.2 IDENTITY OF INVESTIGATIONAL PRODUCT......................75
5.3 DOSAGE SCHEDULE AND ADMINISTRATION OF STUDY MEDICATION......................75
5.4 RATIONALE FOR DOSE SELECTION......................76
5.5 TREATMENT COMPLIANCE......................77
5.6 STUDY DRUG DOSE INTERRUPTION, DE-ESCALATION, OR DISCONTINUATION......................77
5.7 PRIOR AND CONCOMITANT MEDICATIONS AND THERAPIES......................78
5.7.1 Prior Therapy......................78
5.7.2 Concomitant Therapy......................78
5.7.3 Prohibited Therapies......................79
5.7.4 Permitted Therapies......................80
5.8 STUDY MEDICATION MANAGEMENT......................80
5.8.1 Packaging and Labeling of Study Medication......................80
5.8.2 Storage of Study Medication......................81
5.8.3 Study Medication Shipping and Handling......................81
5.8.4 Study Medication Accountability......................82
5.9 PROCEDURES FOR ASSIGNING SUBJECT STUDY NUMBERS......................82
6 STUDY SCHEDULE......................83
6.1 TIME AND EVENTS SCHEDULE......................83
6.2 INFORMED CONSENT/HIPAA AUTHORIZATION......................87
6.3 VISIT SCHEDULE AND PROCEDURES......................88
6.3.1 Baseline Period (Day -1)......................88
6.3.2 Treatment Period Day 1......................90
6.3.3 Treatment Period Months 1-24......................91
6.3.4 Dose Tapering Period (Months 24-25)......................92
6.4 SUBJECT DISCONTINUATION......................94
6.5 STUDY COMPLETION......................94
7 STUDY ASSESSMENTS AND MEASUREMENTS......................94
7.1 DEMOGRAPHIC ASSESSMENTS......................94

7.1.1 Genetic Modifiers of DMD 94
7.2 SAFETY AND TOLERABILITY ASSESSMENTS 95
7.2.1 Interim Medical History 95
7.2.2 Physical Examination, Weight, and Height 95
7.2.3 Vital Signs 96
7.2.4 Clinical Laboratory Tests 96
7.2.5 12-Lead ECG 99
7.2.6 Adverse Events and Serious Adverse Events 100
7.2.7 Bone Age (Hand X-ray) 107
7.2.8 Spine X-ray 107
7.3 CLINICAL EFFICACY AND PHARMACODYNAMIC ASSESSMENTS 108
7.3.1 Time to Stand Test (TTSTAND) 108
7.3.2 Time to Climb Test (TTCLIMB) 108
7.3.3 Time to Run/Walk Test (TTRW) 108
7.3.4 North Star Ambulatory Assessment (NSAA) 109
7.3.5 Six-minute Walk Test (6MWT) 109
7.3.6 Quantitative Muscle Testing (QMT) 110
7.3.7 Pharmacodynamic Biomarker Panel 110
7.3.8 Pediatric Outcomes Data Collection Instrument (PODCI) 111
7.4 TOTAL BLOOD VOLUME REQUIRED 112
8 DATA COLLECTION 112
8.1 SOURCE DOCUMENTS 112
8.2 ELECTRONIC CASE REPORT FORM COMPLETION 113
8.3 DATA PROCESSING 114
8.4 SUBJECT DIARIES 114
9 STATISTICAL METHODS AND PLANNED ANALYSES 114
9.1 STATISTICAL AND ANALYTICAL PLAN (SAP) 115
9.1.1 Deviations from the Statistical Analysis Plan 115
9.2 ANALYSIS POPULATIONS 115
9.2.1 Safety Population 115
9.2.2 Full Analysis Set (FAS) 116
9.2.3 Control Population DNHS Study 116
9.2.4 Control Population FOR DMD Study 116
9.3 MEASURES TAKEN TO AVOID/MINIMIZE BIAS 116
9.4 INTERIM ANALYSIS 116
9.5 MISSING, UNUSED, AND SPURIOUS DATA 117
9.6 STATISTICAL ANALYSIS 117

9.6.1 General Considerations 117
9.6.2 Subject Disposition, Demographics, and Baseline Characteristics 117
9.6.3 Safety Analyses 117
9.6.4 Clinical Efficacy and Pharmacodynamic Analyses 118
9.6.5 Concurrent Medications 119

10 STUDY MANAGEMENT AND ETHICAL AND REGULATORY REQUIREMENTS 119
10.1 REGULATORY APPROVAL AND GOOD CLINICAL PRACTICE 119
10.2 INVESTIGATOR RESPONSIBILITIES 120
10.2.1 Subject Information and Informed Consent 120
10.2.2 Institutional Review Board/Independent Ethics Committee Approval and Other Institutional Requirements 121
10.2.3 Study Documentation 122
10.2.4 Delegation of Investigator Responsibilities 123
10.3 PROTOCOL DEVIATIONS AND VIOLATIONS 123
10.3.1 Protocol Deviation and Violation Definitions 123
10.3.2 Reporting Deviations/Violations 124
10.4 STUDY RECORDS RETENTION AND DIRECT ACCESS TO SOURCE DOCUMENTS124
10.5 DATA AND SAFETY MONITORING BOARD (DSMB) AND OTHER STUDY MONITORING 125
10.5.1 DSMB 125
10.5.2 Study Monitoring 126
10.6 QUALITY ASSURANCE 127
10.7 STUDY TERMINATION AND SITE CLOSURE 128
10.8 SITE TERMINATION 128
10.9 DISCONTINUATION OF STUDY 129

11 DISCLOSURE OF DATA 129
11.1 CONFIDENTIALITY 129
11.2 PUBLICATION 130

12 INVESTIGATOR'S PROTOCOL AGREEMENT 131

13 REFERENCES 132

14 APPENDICES 137
APPENDIX 14.1 VAMOROLONE DOSE CALCULATION WORKSHEET AND BULK DISPENSING GUIDE 138
APPENDIX 14.2 PROTOCOL AMENDMENT #1 COMPLETE LIST OF CHANGES 141

LIST OF TABLES

Table 1 Summary of pharmacokinetic parameters for vamorolone after oral administration of single doses of 0.1, 0.3, 1.0, 3.0, 8.0, and 20.0 mg/kg to healthy subjects under fasted conditions 42

Table 2 Summary of pharmacokinetic parameters for vamorolone after single dose oral administration of 8 mg/kg to healthy subjects under fed and fasted conditions 44

Table 3 Summary of pharmacokinetic parameters for vamorolone during oral administration of 1, 3, 9, and 20 mg/kg doses once daily for 14 days to healthy subjects under fasted conditions 45

Table 4 Serum osteocalcin concentrations (ng/ml) in vamorolone Phase I MAD groups. Within 2 hours prior to dosing (pre-dose); 8 hours postdose day 1 (8 hr); 1 day after 14 days of dosing (14 day); 3 days after last dose (Rec) 53

Table 5 Serum CTX1 concentrations (ng/ml) in vamorolone Phase I MAD groups. Within 2 hours prior to dosing (pre-dose); 8 hours postdose day 1 (8 hr); 1 day after 14 days of dosing (14 day); 3 days after last dose (Rec) 53

Table 6 Pharmacokinetics of 0.25 mg/kg/day vamorolone in 4-7 years DMD boys 56

Table 7 Biomarkers previously defined to be responsive to chronic doses of glucocorticoids in DMD boys 61

Table 8 Schedule of Study Activities 85

Table 9 Vamorolone Dose Tapering 93

Table 10 Hematology, Chemistry, and Lipids Clinical Laboratory Tests 98

Table 11 Urinalysis Clinical Laboratory Tests 98

Table 12 Pharmacodynamic Biomarkers 111

Table 13 Blood Sample Number and Volume 112

LIST OF FIGURES

Figure 1 **Arithmetic mean ± standard error plasma concentrations of vamorolone (VBP15) after oral administration of single doses of 0.1, 0.3, 1, 3, 8, and 20 mg/kg to healthy subjects under fasted conditions - linear plot (top); semi-logarithmic plot (bottom) 41**

Figure 2 **Relationship between individual subject vamorolone AUC($_{inf}$) and dose after oral administration of single doses of 0.1, 0.3, 1, 3, 8, and 20 mg/kg to healthy subjects under fasted conditions 42**

Figure 3 **Arithmetic mean ± standard error plasma concentrations of vamorolone (VBP15) after single dose oral administration of 8 mg/kg to healthy subjects under fed and fasted conditions - linear (top panel) and semi-logarithmic (bottom panel) axes 43**

Figure 4 **Arithmetic mean ± standard error plasma concentrations of vamorolone (VBP15) on Days 1 and 14 during oral administration of 1, 3, 9, and 20 mg/kg doses once daily for 14 days to healthy subjects under fasted conditions (linear axes) 46**

Figure 5 **Morning cortisol measures in the vamorolone Phase I MAD volunteers.* 48**

Figure 6 **Fasting serum glucose during the Phase I MAD period (two weeks daily treatment)..50**

Figure 7 **Comparisons of serum osteocalcin and CTX1 show no differences of placebo compared to 20 mg/kg/day vamorolone. Within 2 hours prior to dosing (pre-dose); 8 hours postdose day 1 (8 hr); 1 day after 14 days of dosing (14 day); 3 days after last dose (Rec).......................................52**

Figure 8 **Pharmacokinetics of 0.25 mg/kg/day vamorolone in 4-7 years DMD boys 56**

LIST OF ABBREVIATIONS

Abbreviation	Definition/Term
ACTH	adrenocorticotropic hormone
ADL	activities of daily living
AE	adverse event
ALP	alkaline phosphatase
ALT	alanine aminotransferase
ANCOVA	analysis of covariance
AST	aspartate aminotransferase
AUC	area under the concentration-time curve
$AUC_{0\text{-}24hr}$	area under the concentration-time curve from time 0 to 24 hours
$AUC_{0\text{-}t}$	area under the concentration-time curve from time 0 to time t
AUC_{inf}	area under the concentration-time curve from time 0 to infinity
AUC_{last}	area under the plasma concentration-time curve from time 0 to the last observed measurable concentration
BMI	body mass index
BUN	blood urea nitrogen
C	Celsius
CD23	cluster designation 23
CFR	Code of Federal Regulations
CINRG	Cooperative International Neuromuscular Research Group
CK	creatine kinase
CL	clearance
ConA	concanavalin A
cm	centimeter
C_{max}	maximum observed plasma concentration
CQMS	CINRG Quantitative Measurement System
CTCAE	Common Terminology Criteria for Adverse Events
CTM	Clinical Trial Material
CTX	carboxy-terminal telopeptide
CYP	cytochrome P450
dL	deciliter
DMD	Duchenne muscular dystrophy
DNA	deoxyribonucleic acid
DSMB	Data and Safety Monitoring Board
ECG	electrocardiogram
eCRF	electronic case report form

Abbreviation	Definition/Term
EDC	electronic data capture
F	Fahrenheit
F, F%	bioavailability, percent bioavailability
FAS	Full Analysis Set
FDA	Food and Drug Administration
GCP	Good Clinical Practice
GGT	gamma glutamyl transferase
GLDH	glutamate dehydrogenase
GLP	Good Laboratory Practice
HbA1c	hemoglobin A1c
HDL	high density lipoprotein
HIPAA	Health Insurance Portability and Accountability Act
hr	hour
ICF	Informed Consent Form
ICH	International Conference on Harmonisation
IEC	Independent Ethics Committee
IGFBP-2	insulin-like growth factor-binding protein 2
IGFBP-5	insulin-like growth factor-binding protein 5
IL-22BP	interleukin-22 binding protein
IND	Investigational New Drug
IRB	Institutional Review Board
K_2-EDTA	dipotassium ethylenediaminetetraacetic acid
kg	kilogram
λ_z	elimination rate constant
L	liter
LLC	Limited Liability Company
LC-MS	Liquid Chromatography – Mass Spectrometry
LDH	lactate dehydrogenase
LDL	low density lipoprotein
m	meter
MAD	multiple ascending dose (study)
mdx	mouse model lacking dystrophin
MedDRA	Medical Dictionary for Regulatory Activities
mg	milligram
min	minute
MIST	Metabolites in Safety Testing

Abbreviation	Definition/Term
mITT	modified Intention to Treat
mL	milliliter
MMP-3	matrix metalloproteinase-3
MMP-12	matrix metalloproteinase-12
MS Band	Microsoft Band
NADPH	nicotinamide adenine dinucleotide phosphate
NCA	noncompartmental
NF-κB	nuclear factor kappa-light-chain-enhancer of activated B cells
ng	nanogram
No., n	number
NOAEL	no observed adverse effect level
NSAA	North Star Ambulatory Assessment
OTC	over-the-counter (non-prescription medication)
%CV	percentage coefficient of variation
P1NP	serum aminoterminal propeptide of type I collagen
PBL	peripheral blood leukocytes
PD	pharmacodynamics
PHI	Protected Health Information
PK	pharmacokinetics
PODCI	Pediatric Outcomes Data Collection Instrument
PR [PQ]	interval from onset of P wave to start of the QRS complex
Q	quarter
QMT	Quantitative Muscle Testing
QRS	in electrocardiography, the complex consisting of Q, R, and S waves, corresponding to depolarization of ventricles [complex]
QSAR	quantitative structure-activity relationship
QT	in cardiology, the interval between the start of the Q wave and end of the T wave
QT_cF	Corrected QT interval using Fredericia's calculation
RBC	Red Blood Cell
RR	in electrocardiography, the interval between successive R waves (peaks of QRS complexes)
6MWT	Six-minute Walk Test
SAD	single ascending dose (study)
SAE	Serious Adverse Event
SAP	statistical analysis plan
SD	standard deviation

Abbreviation	Definition/Term
SOC	system organ class
SOP	standard operating procedures
SSL	secure socket layers
$t_{1/2}$	terminal half-life
TEAE	treatment-emergent adverse event
T_{max}	time to maximum observed plasma concentration
TTCLIMB	Time to Climb (Test)
TTSTAND	Time to Stand (Test)
TTRW	Time to Run/Walk (Test)
US	United States
vs.	versus
V_{ss}	volume of distribution at steady state
V_z	terminal phase volume of distribution
WBC	White Blood Cell
WHO	World Health Organization

1 INTRODUCTION

1.1 Background and Unmet Need

Duchenne muscular dystrophy (DMD) is a rapidly progressive form of muscular dystrophy that occurs primarily in males and manifests prior to the age of six years. Duchenne muscular dystrophy affects approximately 1 in 3,600 to 9,300 male births worldwide.[1] Duchenne muscular dystrophy is caused by mutations in the dystrophin gene which codes for a protein that provides structural stability to the dystroglycan complex on muscle cell membranes.[2] The lack of dystrophin reduces plasma membrane stability. Membrane destabilization results in altered mechanical properties and aberrant signaling, which contribute to membrane fragility, necrosis, inflammation, and progressive muscle wasting.[3]

In addition to the significant contribution of membrane destabilization and mechanical injury in DMD, aberrant intracellular signaling cascades that regulate inflammatory and immune processes also contribute to DMD pathophysiology. Up-regulated inflammatory gene expression and activated immune cell infiltrates, at least partially mediated by nuclear factor kappa-light-chain-enhancer of activated B cells (NF-κB) activation, are evident during early disease stages and play a significant role in muscle wasting.[3] NF-κB has been shown to regulate the expression of numerous inflammatory genes in immune cells and muscle fibers,[4,5,6,7] and the infiltration and activation of these cells can trigger muscle fiber death.[8,9]

Although significant advances have been made in understanding the etiology of DMD, a cure has not been found, and current treatment options are all medications used "off-label" to alleviate the symptoms of DMD. Despite scientific advances, only glucocorticoids, such as prednisone, have consistently demonstrated efficacy in clinical trials.[10] Further, many disease modifying technologies that are currently in development focus on subsets of dystrophin mutations and therefore do not address the unmet need in all persons with DMD. However, it is likely that glucocorticoids will need to be co-administered with many of these compounds for maximum effect and glucocorticoids have extensive side effect profiles, often limiting long-term administration. The current

goal of DMD research is to find a mutation-independent treatment that matches or exceeds the efficacy of glucocorticoids with a significantly lower side effect profile.

Vamorolone is a first-in-class delta-9, 11 chemical compound belonging to the structural class of synthetic steroidal drugs, which includes the glucocorticoids prednisone, methylprednisone, deflazacort, and dexamethasone.[11] The chemical structure of vamorolone has optimized four subactivities of traditional glucocorticoid drugs, namely transactivation, transrepression, physiochemical membrane properties, and mineralocorticoid receptor antagonism.[11] By reducing transactivation subproperties, retaining transrepression, imparting membrane stabilizing properties, and inhibiting the mineralocorticoid receptor pathway, vamorolone has favorable efficacy and side effect profiles relative to classic glucocorticoids in nonclinical models and is anticipated to be an attractive candidate for the treatment of DMD in pediatric patients.

In vitro, nonclinical, and clinical (Phase I) data to date suggest that vamorolone may offer a much needed alternative to the current glucocorticoids which are standard of care for DMD,[12] with administration beginning around the age of 5 years in most developed countries, or even earlier in some cases.

The significant effects of glucocorticoids on growth and development, however, prevent their routine administration in infancy or ‘toddler’ years, despite evidence that the earlier the administration, the better the overall functional outcome.[13] The cumulative side effects of glucocorticoids, including excess weight, delayed puberty, fragile skin, loss of bone mineral density, bruising, and Cushingoid appearance continue to negatively impact on the quality of life of the individual, leading to significant variations in clinical practice.[14] Glucocorticoids also contribute to further muscle damage with long-term administration. Vamorolone has shown few if any of the side effects of traditional glucocorticoids in mouse models of DMD.[11,15,16]

This study is targeted to explore whether vamorolone will show at least equal efficacy to glucocorticoids with a more favorable side effect profile, thereby improving the quality of life for DMD patients. This profile would enable use of vamorolone in DMD boys who are at a younger age than when glucocorticoid treatment is currently initiated. In

addition, vamorolone could be prescribed in later stage non-ambulant young men with DMD and for a longer period of time, or in very young infants or neonates with DMD, where the risk:benefit balance of glucocorticoids is often less favorable.

Efficacy may also be improved over classic glucocorticoids in the longer term. In addition to the anti-inflammatory properties of vamorolone as a result of NF-κB pathway inhibition, vamorolone may also improve efficacy over conventional glucocorticoids due to the lack of interference in the AKT1/FOXO pathway, a key feature of glucocorticoid therapy which leads in the long term to muscle wasting and atrophy.[17] Further, vamorolone has been recently demonstrated to improve asynchronous remodeling, believed to be a component of progressive muscle weakness and wasting in DMD.[18] Vamorolone is an antagonist to the mineralocorticoid receptor, whereas glucocorticoids are typically agonists. An antagonist for the mineralocorticoid receptor, epleronone, was recently shown to significantly improve DMD heart function.[19] Finally, vamorolone imparts physical stability to myofiber plasma membranes, whereas prednisone destabilizes membranes. This property addresses the primary defect of membrane instability in dystrophin deficient myofibers in DMD.[15]

Potentially, the administration of vamorolone to a DMD patient may begin soon after birth to slow the dystrophic process of muscle, retaining regenerative capacity and substantially improving patient quality of life.

1.2 Nonclinical Experience

The safety pharmacology, pharmacokinetics (PK) and metabolism, and toxicology of vamorolone have been evaluated in multiple nonclinical studies *in vitro* and in mice, rats, beagle dogs, and cynomolgus monkeys *in vivo*.

1.2.1 Safety Pharmacology

Stunted growth is a significant side effect of chronic glucocorticoid use in children.[20,21] Chronic treatment with glucocorticoids negatively affects bone growth and development and can cause osteoporosis.[22,23]

The effect of vamorolone as compared to prednisolone on bone growth and development was evaluated in the *mdx* mouse model of DMD that lacks dystrophin due to a premature chain-terminating mutation in the mouse homologue of the dystrophin gene. In the pre-symptomatic *mdx* study, tibia length was measured to determine if vamorolone inhibited bone growth. Prednisolone significantly decreased tibia length whereas vamorolone did not affect tibia length at any concentration tested. Micro-computed tomography was performed on femurs to examine bone density and structure. Comparison of vehicle, prednisolone, and the highest vamorolone dose showed prednisolone to significantly reduce trabecular thickness compared to vehicle, while vamorolone did not.[15]

In normal, male CD-1® mice, these effects were reproduced. Unlike CD-1 mice treated with prednisolone, CD-1 mice receiving vamorolone did not experience tibia length shortening.[16] However, at the highest vamorolone dose tested, mice did have significantly reduced body length, though to a lesser extent as compared to prednisolone.

Duchenne muscular dystrophy is associated with cardiomyopathy that can become life threatening, and increased fibrosis with prednisone treatment in heart muscle of the *mdx* mouse has been reported.[24] Histologically, clear fibrosis was evident in 50% of young (8-week) prednisolone-treated mouse hearts compared to no incidence of fibrosis identified in the other groups (wild type; *mdx* vehicle, and vamorolone -treated).

Pharmacologically, glucocorticoids show immunosuppressive and immunotoxic properties that limit therapeutic windows and long-term use. Vamorolone (5, 15, 30 mg/kg/day) was benchmarked against prednisolone (5 mg/kg/day) to determine if similar properties were observed.[15] Untreated *mdx* mice showed increased numbers of peripheral blood leukocytes (PBL) and enlarged spleens resulting from ongoing muscle damage compared to wild type mice. Vamorolone treatment reduced spleen mass and PBL counts in a dose-dependent manner. This finding is attributed to a reduction in muscle damage by vamorolone that decreases spleen size to levels resembling those in wild type mice. Prednisolone reduced these measures below wild type, suggesting immunosuppressive and/or immunotoxic properties. Further, prednisolone significantly decreased viable splenocytes per gram of tissue ($p<0.005$), whereas this decrease was not

observed for any vamorolone dose tested (ReveraGen Report No. MDX-RBP-VBP15-02).[15]

To further query the potential immune modulation, the effects of vamorolone and prednisolone on counts of splenic B and T-lymphocytes isolated from treated *mdx* mice were examined. CD4+ T-cell activation was assayed by stimulation of splenocytes with the T-cell mitogen, concanavalin A (ConA). Splenocytes obtained from prednisolone-treated mice displayed a significant reduction of the percentage of splenic activated CD4+CD25+ T-cells upon ConA stimulation while splenocytes derived from vamorolone-treated mice did not (ReveraGen Report No. MDX-RBP-VBP15-02).

Taken together, these findings suggest that while prednisolone treatment leads to a reduction in T-cell number and activation status, vamorolone modulates inflamed *mdx* immune systems towards a wild type state without compromising T-cell activation status.

1.2.2 Pharmacokinetics and Metabolism

1.2.2.1 Single Dose

Vamorolone PK profiles were determined in male CD-1 mice, Sprague Dawley rats and beagle dogs after a single intravenous injection of 10 mg/kg and after a single oral dose of 50 mg/kg in mice and rats and 30 mg/kg in dogs.

Pharmacokinetic results for vamorolone following a single intravenous administration of 10 mg/kg in Crl:CD1(ICR) mice demonstrated a clearance (CL) of 18.8 mL/min/kg. The terminal half-life ($t_{½}$) was 0.35 hours. Volume of distribution at steady state (V_{ss}) was 0.76 L/kg. Following oral administration of 50 mg/kg in mice, the maximum observed plasma concentration (C_{max}) of 6787 ng/mL was observed at 2 hours (time to maximum observed plasma concentration [T_{max}]) after drug administration, and percent bioavailability (F%) was 74.5%. Following oral administration of 15 mg/kg via cherry syrup, the C_{max} of 1527 ng/mL was observed at 2 hours after drug administration and bioavailability was 47.7% (ReveraGen Report No. PH-DPMK-VBP-10-004).

Pharmacokinetic results for vamorolone following a single intravenous administration of 50 mg/kg in Sprague Dawley rats indicated a CL of 20.2 mL/min/kg. The $t_{½}$ was

0.58 hours. V_{ss} was 0.77 L/kg, which was similar to that observed in mice. After oral administration of 50 mg/kg in rats, a C_{max} of 2543 ng/mL was observed at 4 hours after dose administration, and bioavailability was 47.8% (ReveraGen Report No. PH-DPMK-VBP-10-007).

In beagle dogs, vamorolone had a CL of 24.7 mL/min/kg. The $t_{½}$ was 5.42 hours and V_{ss} was 1.93 L/kg. After oral administration of 30 mg/kg in dogs, a C_{max} of 814 ng/mL was observed at 6 hours after dose administration and bioavailability was 53.2% (ReveraGen Report No. 48504-10-464).

Vamorolone clearance was therefore comparable in all 3 species studied (19-25 mL/min/kg). Bioavailability ranged from approximately 50% in mouse (cherry syrup), rat, and dog to 75% in the mouse (30% Labrafil) (ReveraGen Report Nos. PH-DPMK-VBP-10-004, PH-DPMK-VBP-10-007, 48504-10-464).

1.2.2.2 Multiple Dose

Crl:CD1(ICR) mice were administered vamorolone or vehicle once daily for 28 consecutive days. Vamorolone exposure (as assessed by the C_{max} and area under the concentration-time curve [AUC]) increased with increasing dose on Study Days 1 and 28. Repeated dosing of vamorolone over a 28-day duration was associated with decreases in mean vamorolone AUC_{last} values in the 30 and 100 mg/kg dose groups compared to Day 1, indicating possible enzyme induction. On Study Day 28, mean AUC_{last} values were 1.81-fold and 5.02-fold lower compared to Study Day 1 for the 30 and 100 mg/kg dose groups, respectively. The observed difference in exposure relative to Day 1 increased with the increase in administered dose of vamorolone (ReveraGen Report No. 1998-009).

Beagle dogs were either administered vamorolone or vehicle once daily for 28 consecutive days. Vamorolone exposure in dogs (as assessed by C_{max} and AUC_{last}) generally increased with increasing dose on Study Days 1 and 28. For the 2 and 10 mg/kg dose groups, exposure on Day 28 was generally higher than on Day 1, indicating possible inhibition of metabolism of vamorolone at these dose levels. On Day 28, mean AUC_{last} values were 2.35-fold and 2.43-fold (males) and 3.03-fold and 3.23-fold

(females) higher compared to Study Day 1 for the 2 and 10 mg/kg/day dose groups, respectively. For the 50 mg/kg dose group, exposure on Day 28 was similar to that on Day 1. At the 50 mg/kg dose, AUC_{last} values in males were 1.71-fold lower whereas females were 1.22 higher on Day 28 compared to Day 1 (ReveraGen Report No. 031302).

Beagle dogs were administered vehicle or vamorolone at doses of 2 mg/kg/day, 10 mg/kg/day, or 50 mg/kg/day for 39 weeks. Systemic exposure ($AUC_{0\text{-}24hr}$) to vamorolone appeared to be independent of sex. Mean $AUC_{0\text{-}24hr}$ and C_{max} values for vamorolone increased with increasing dose in an approximately dose proportional manner on Days 1 and 270. Mean systemic exposure ($AUC_{0\text{-}24hr}$) to vamorolone appeared to increase following repeated administration of vamorolone. Due to the alterations in the feeding regimen, changes in systemic exposure following repeated administration should be viewed with caution due the influence of feeding on exposure. For the 2 and 10 mg/kg/day dose groups, exposure on Day 270 was generally higher than on Day 1, indicating possible inhibition of metabolism of vamorolone at these dose levels. On Day 270, mean $AUC_{0\text{-}24hr}$ values were 2.34-fold and 2.98-fold higher compared to Study Day 1 for the 2 and 10 mg/kg/day dose groups, respectively. For the 50 mg/kg dose group, exposure on Day 270 was 2.07-fold higher compared to that on Day 1 (ReveraGen Report No. 1998-014).

Non-naïve cynomolgus monkeys were administered vamorolone (300 and 600 mg/kg/day) or vehicle once daily for 7 consecutive days. Vamorolone exposure (as assessed by C_{max} and AUC_{last}) generally increased with increasing dose on Study Days 1 and 7 with the exception of male monkeys on Day 7, which showed no clear increase in exposure between the 300 and 600 mg/kg/day dose levels. Repeated dosing over the 7-day study duration was associated with decreases in mean plasma vamorolone AUC_{last} values for female and male monkeys indicating possible metabolic induction. On Day 7, mean AUC_{last} values were 1.60-fold, 2.19-fold, and 2.02-fold lower in females and 1.20-fold, 2.09-fold, and 2.88-fold lower in males compared to Study Day 1 for the 100, 300 and 600 mg/kg/day dose groups, respectively (ReveraGen Report Nos. 1998-001, SW11-0418).

1.2.2.3 Distribution

In the plasma protein binding studies, percent bound was similar in human and mouse cells in culture (88.06% and 86.71%, respectively). In the blood partition experiment done *ex vivo*, the blood to plasma ratio was similar between human and mouse (0.87 and 0.68, respectively), but the red blood cell to plasma ratio for the mouse (0.33) was less than half that of the human (0.74). Human *in vivo* data are presented in Section **1.3** (VBP15-001). In the blood/brain concentration mouse experiment *in vivo*, the plasma concentrations of vamorolone were higher than brain concentrations with the AUC and C_{max} approximately 2-fold higher in plasma than in brain (ReveraGen Report Nos. ADME-NCG-PPB-NC135, ADME-VBP-PPB-V002, ADME-NCG-BP-NC134, NCATS 2013-38).

1.2.2.4 Metabolism

The *in vitro* and *in vivo* data demonstrate that vamorolone can be metabolized via multiple metabolic pathways, including glucuronidation, hydroxylation, and reduction. Glucuronidation appeared to be the major metabolic pathway in human cells *in vitro*. All metabolites observed in human *in vitro* were observed in monkey *in vitro*. Most human metabolites identified *in vitro* were also found in mouse and dog. Thus, there is no unique human metabolite identified for vamorolone.

The metabolic stability of vamorolone was assessed in non-Good Laboratory Practice (GLP) studies. Based on the data generated, vamorolone was highly stable for up to 60 minutes in human, monkey, dog, and mouse liver microsomes in the presence or absence of nicotinamide adenine dinucleotide phosphate (NADPH) and stable for up to 60 minutes in rat liver in the absence of NADPH. Moderate metabolism was apparent in rat liver microsomes in the presence of NADPH stimulation (35% remaining), suggesting that rat was a high metabolizer of vamorolone relative to other species (mouse, dog, human) (ReveraGen Report Nos. NIH-R2526, and ADME-VBP-LM-V003).

Vamorolone did not significantly inhibit any of the cytochrome P450 (CYP) enzyme isoforms tested (CYP 1A2, 2B6, 2C8, 2C9, 2C19, 2D6, and 3A4). Vamorolone moderately induced CYP3A4 (24% to 42%), indicating that vamorolone is a potential

inducer of CYP3A4 (ReveraGen Report Nos. ADME-VBP-Inhibition-V005, ADME-VBP-Induction-V006, ADME-VBP-Induction-V009).

1.2.2.5 Excretion

Vamorolone showed high plasma clearance in rats but, consistent with the extensive metabolism in hepatocytes from this species, the biliary and urinary excretion of the parent compound was low with an average of <0.05% of the dose recovered in bile and approximately 0.1% in urine. Overall, vamorolone showed high plasma clearance and extremely low biliary and urinary excretion (ReveraGen Report No. NCATS 2013-44).

1.2.3 Toxicology

1.2.3.1 Single Dose

Crl:CD1(ICR) mice were administered vamorolone once via oral gavage at 50, 125, 250, and 500 mg/kg and observed for abnormalities. All animals survived to their scheduled termination, and there were no significant abnormalities observed. There was a slight decrease in body weight attributed to vamorolone in both males and females at doses above 125 mg/kg. A dose dependent decrease in food consumption related to vamorolone was also observed in males and females. No other abnormalities were observed in experimental mice (ReveraGen Report No. 1998-002).

Beagle dogs received single 60, 180, 360, and 750 mg/kg doses of vamorolone using an escalation study design with a 4-day washout period between doses. All animals survived dose escalation. Clinical signs attributed to vamorolone (750 mg/kg) included red discoloration of the ears and face. This effect occurred within a few hours of dosing and was transient. The highest dose also resulted in increased white blood cell count (increased neutrophils and monocytes [female only] and decreased lymphocytes and eosinophils [male and female]). At the 360 and 750 mg/kg dose levels, slight elevations in albumin were observed. A mild elevation in cholesterol at the 750 mg/kg (and possibly 360 mg/kg) dose level was also observed (ReveraGen Report No. 13788.01.01).

In cynomolgus monkeys, single oral doses of up to 500 mg/kg were well tolerated with no significant abnormalities observed (ReveraGen Report No. 1998-001).

1.2.3.2 Multiple Dose

Vamorolone or vehicle was administered to Crl:CD1(ICR) mice once daily for 28 consecutive days at doses of 10, 30 and 100 mg/kg/day. All animals survived to their scheduled necropsy with the exception of a female mouse (100 mg/kg/day dose group) that was found dead on Day 16. The cause of death was considered incidental and attributed to a dosing injury based on the amount of red fluid in the thoracic cavity.

No effects attributable to vamorolone were observed in food consumption, ophthalmic examination, or urinalysis during the study. Dose-dependent decrease in body weight gain was observed at all doses; however, weight was fully regained during the recovery period. Adrenal gland weights were variable between groups and generally decreased, but without a dose response relationship, and correlated microscopically with minimal to moderate vacuolar degeneration and cortical atrophy. After the 2-week recovery period there was evidence of vacuolar degeneration of the adrenal gland. Liver weights were significantly increased at the 100 mg/kg/day dose level. Hepatocellular hypertrophy, increased vacuolation, and necrosis (single cell) were seen in a few male mice at 30 mg/kg/day. There was evidence of lipid and glycogen accumulation. Serum alanine aminotransferase and aspartate aminotransferase levels were higher with associated microscopic hypertrophy/vacuolation/necrosis at 100 mg/kg/day. Spleen weights decreased in a dose-dependent manner and correlated with a decreased number of lymphocytes in spleen. Thymus weights decreased in a dose dependent manner and were associated microscopically with lymphoid atrophy. Mice had dose-dependent reductions in serum lymphocytes which were significant in the 100 mg/kg dose group. After the recovery period, all parameters returned to normal (untreated) except for thymus weights, which were increased.

Based on the liver-related findings in this study, the no observed adverse effect level (NOAEL) for vamorolone in mice is 30 mg/kg/day (ReveraGen Report No. 1998-009).

Vamorolone or vehicle was administered to beagle dogs once daily for 28 consecutive days at doses of 2, 10 and 50 mg/kg/day. All animals survived to their scheduled termination and no effect of vamorolone was noted on gross visual inspection, body

weight, body temperature, food consumption, ophthalmology, electrocardiography or urinalysis parameters at necropsy. A dose-dependent decrease in the expected normal body weight gain was observed at all doses but weights generally increased to a normal level during the recovery period.

Adrenal gland weights decreased with vamorolone treatment, which correlated with mild or moderate diffuse bilateral atrophy of the adrenal cortex, mild multifocal bilateral vacuolation of the adrenal cortex, increased white blood cell and neutrophil counts, and decreased eosinophil counts. Liver weights increased in the 50 mg/kg/day dose group, which correlated with diffuse hypertrophy and vacuolation and increased levels of alkaline phosphatase and gamma glutamyltransferase. Spleen weights decreased, which correlated with lymphoid depletion. Thymus weights decreased, which corresponded to diffuse lymphoid depletion. With the exception of diffuse depletion of lymphocytes in thymus in the 50 mg/kg group, all abnormal parameters returned to normal during the recovery period.

The NOAEL was considered by the study director to be 10 mg/kg/day. Although reversible, the liver changes were considered adverse at 50 mg/kg/day because the severity score was moderate and the changes were diffuse in nature in all animals treated at the high dose. This determination is in contrast to the conclusion drawn by the study pathologist, who considered the NOAEL to be 50 mg/kg/day due to reversibility following cessation of dosing (ReveraGen Report No. 31302).

Vamorolone or vehicle was administered to beagle dogs once daily for 39 weeks at doses of 2, 10 and 50 mg/kg/day. Six dogs of each sex received each dose or placebo, and two of the six dogs of each sex at each dose or placebo were followed for an additional 4 weeks to evaluate reversibility, progression, or delayed appearance of any observed changes. One male dog that received 50 mg/kg/day was euthanized in extremis on Day 273 due to paraphimosis (an extended penis). All other animals survived to their scheduled termination.

Detailed clinical observations considered test article-related at 50 mg/kg/day, and reversible, included decreased activity (considered adverse), struggling during dosing,

feces soft, limb function impaired, interdigital cysts, and unkempt appearance (considered adverse). Test article-related, dose-dependent increases in body weight gains correlating with increases in food consumption were observed relative to controls in males at all dose levels and in females at 10 and 50 mg/kg/day. Test article-related, reversible increases in average mean food consumption, relative to controls, over the course of the 39-week dose phase were observed in both sexes at 10 and 50 mg/kg/day.

No test article-related ophthalmological effects were noted. No test-article-related changes were noted in respiratory rates or rectal temperatures. There may have been a mild dose-related reversible increase in the heart rate at the terminal post-dose interval that was significantly different from vehicle in both sexes following the 50 mg/kg/day dose. Semen analysis/evaluation for test article affects could not be conducted as there were not enough viable samples collected.

Test article-related effects on clinical pathology endpoints with microscopic correlates included the following:

- A hepatocellular and hepatobiliary effect in males at 10 mg/kg/day and both sexes at 50 mg/kg/day, which included increased alkaline phosphatase, gamma glutamyltransferase, alanine aminotransferase, and aspartate aminotransferase activity. These changes correlated with microscopic changes in the liver, bile duct, and gall bladder. This spectrum of changes was considered adverse in both sexes at 50 mg/kg/day.
- There was also evidence of an inflammatory response in both sexes at 50 mg/kg/day, which included increased total leukocyte, neutrophil, and monocyte counts, and increased fibrinogen and/or globulin concentrations. The inflammatory response was likely secondary to inflammation in the liver associated with hepatocellular necrosis. Platelet counts were also increased in both sexes at 50 mg/kg/day and may have been secondary to the inflammatory response.

Following a 4-week recovery period, all noted clinical pathological changes resolved, with the exceptions of increased alanine aminotransferase activity in both sexes at 50 mg/kg/day, and increased globulin in males at 50 mg/kg/day.

Reversible, test article-related macroscopic findings included mildly to moderately enlarged livers in males and females at 50 mg/kg/day, which correlated microscopically with panlobular hepatocellular hypertrophy and/or hepatocellular vacuolation; hemorrhage in the gall bladder of one 50 mg/kg/day female, that was associated with moderate acute inflammation and mild vascular necrosis, and considered to be adverse; red focus/foci within the pylorus of the stomach of one 50 mg/kg/day female and one male at 10 mg/kg/day, which correlated microscopically with mild acute inflammation in the female.

Test article-related organ weight changes at the terminal necropsy included decreases in adrenal gland weights in both sexes at ≥ 2 mg/kg/day (microscopic correlate of bilateral cortical atrophy); increases in liver weights in both sexes at ≥ 10 mg/kg/day (microscopic correlates of panlobular hepatocellular hypertrophy and/or hepatocellular vacuolation); increases in kidney weights in females at ≥ 10 mg/kg/day and males at 50 mg/kg/day (microscopic correlate of bilateral tubular vacuolation); decreases in prostate gland weights in males at 50 mg/kg/day (microscopic correlate of decreased secretory product). These organ weight changes were all reversible, except for the decreases in the prostate gland. Microscopic evaluation revealed the following test article-related changes: adrenal glands (atrophy of the zona fasciculata and zona reticularis and hypertrophy/hyperplasia of the zona glomerulosa in both sexes at ≥ 10 mg/kg/day and atrophy was considered adverse); esophagus and pylorus of the stomach (erosion/ulceration in a few animals of both sexes at 50 mg/kg/day); gallbladder (hypertrophy/hyperplasia of the mucosal epithelium in both sexes at ≥ 10 mg/kg/day and cytoplasmic vacuolation of the mucosal epithelium in males at ≥ 10 mg/kg/day and females at ≥ 2 mg/kg/day); liver (hepatocellular vacuolation in males at ≥ 10 mg/kg/day and females at ≥ 2 mg/kg/day, panlobular hypertrophy in males at 50 mg/kg/day and females at ≥ 10 mg/kg/day, and inflammation/necrosis in both sexes at 50 mg/kg/day and considered adverse, bile duct hyperplasia in both sexes at 50 mg/kg/day, bile duct

hypertrophy in males at 50 mg/kg/day and females at ≥ 10 mg/kg/day, and cytoplasmic vacuolation of the bile duct epithelium in both sexes at ≥ 10 mg/kg/day); kidneys (bilateral tubular vacuolation in males at 50 mg/kg/day and females at ≥ 10 mg/kg/day and an increased incidence of bilateral basophilic tubules in males and females at 50 mg/kg/day); lymphoid depletion in both sexes at 50 mg/kg/day in mandibular and mesenteric lymph nodes, thymus and spleen (with extramedullary hematopoiesis in 50 mg/kg/day females); bone marrow in the sternum (increased adipocytes in males at ≥ 2 mg/kg/day and females at 50 mg/kg/day); testes (spermatocyte/spermatid degeneration in males at 50 mg/kg/day); epididymides (oligospermia/germ cell debris in males at 50 mg/kg/day); ovaries (absent corpora lutea in females at ≥ 2 mg/kg/day and considered adverse); the mammary gland and other tissues in the female reproductive tract (uterus, cervix, and vagina) of these animals were consistent with animals that have not ovulated; vacuolation in the epithelium of the mammary gland duct in females at 50 mg/kg/day; parotid salivary gland (cytoplasmic alteration in both sexes at ≥ 10 mg/kg/day); biceps femoris (atrophy of the skeletal muscle in both sexes at 50 mg/kg/day); skin (atrophy and alopecia/hypotrichosis in males at 50 mg/kg/day and females at ≥ 10 mg/kg/day); prostate gland (decreased secretory product in males at 50 mg/kg/day); thyroid glands (bilateral increased colloid in males at ≥ 10 mg/kg/day).

Many of the findings were felt by the Study Director to be consistent with the pharmacology of the test article including cortical atrophy of the adrenal glands (affecting the zona fasciculata and reticularis), generalized lymphoid depletion in lymphoid tissues (thymus, spleen, and lymph nodes), increased adipocytes in the bone marrow, atrophy of the skeletal muscle, alopecia/hypotrichosis and atrophy of the skin (thinning of the dermal collagen and atrophy of hair follicles and adnexa), an absence of corpora lutea in the ovary (likely indicative of delayed puberty), decreased secretory product in the prostate gland, and multiple changes in the liver. The liver had panlobular hypertrophy and vacuolation of hepatocytes consistent with glycogen accumulation. Due to the magnitude of hypertrophy and vacuolation, there were (likely secondary) foci of hepatocellular necrosis and inflammation.

Test article-related microscopic findings at the recovery necropsy were present in the adrenal glands, liver, gallbladder, kidneys, stomach (pylorus), female reproductive tract (ovaries), male reproductive tract (testes, epididymides, prostate gland), mesenteric lymph node, skeletal muscle (biceps femoris), and parotid salivary gland.

The No Observed Adverse Effect Level was 2 mg/kg/day for males; a No Observed Adverse Effect Level was not observed for females (ReveraGen Report No. 1998-014).

Non-naive cynomolgus monkeys were administered vamorolone or vehicle once daily for 7 consecutive days at doses of 100, 300, and 600 mg/kg. All animals survived until the end of the study period. There were effects on clinical observations, food consumption, and urinalysis attributable to vamorolone that are described below.

There was a dose proportional decrease in body weight gain observed in males and females at each dose (up to 11% and 9% respectively) related to vamorolone. A cessation of the body weight loss in treatment was observed during the recovery phase but no recovery of body weight lost during the 7 days of dosing was observed.

At termination there were nonsignificant increases in red cell mass and decreases in lymphocytes (up to 56%) in the 600 mg/kg/day dose group. However, most individual animals, including controls, had decreases in lymphocytes (up to 81%) at termination relative to their respective pretest. They had resolved by the recovery interval in both sexes.

In both sexes receiving ≥ 300 mg/kg/day, there was increased urea nitrogen (up to 141%), creatinine (up to 58%), total protein (up to 15%), albumin (up to 11%), globulin (up to 25%), and/or potassium (up to 39%) with concurrent decreases in sodium (up to 10%) and chloride (up to 10%) relative to controls. At the recovery interval, the majority of these effects had resolved (ReveraGen Report No. 1998-001).

1.2.3.3 Genotoxicity

The mutagenic and genotoxic potential of vamorolone was assessed in several assays. A non-GLP Ames screen was negative for bacterial mutations (ReveraGen Report No. BIO-VBP-001-AMES). In a GLP Ames test, no background lawn toxicity was observed;

however, a reduction in revertant counts was observed (ReveraGen Report No. AD79DT.502ICH.BTL). Vamorolone was negative for inducing chromosomal aberrations in cultured mouse lymphocytes without and with metabolic activation (ReveraGen Report No. AD79DT.704.BTL).

Femoral bone marrow was microscopically evaluated for the presence of polychromatic erythrocytes (PCEs) containing micronuclei. No significant reductions in the PCEs/EC (total erythrocytes) ratio were observed in the vamorolone groups compared to the vehicle control group. Although statistically significant increases in the incidence of micronucleated PCEs in the vamorolone treated groups were observed, no dose response was observed with respect to other groups and the values of micronuclei for the individual animals were within the historical range. Therefore, the statistically significant increase was considered as biologically insignificant (ReveraGen Report No. AD76BK.123012ICH.BTL).

A study was performed to evaluate the potential mutagenicity of two theoretical epoxide impurities related to the drug substance vamorolone (formerly VBP15), which is a steroid-like structure containing a delta 9,11 double bond. The delta 9,11 epoxide structures evaluated were VBP15-B-3, which is structurally similar to vamorolone except for the epoxide moiety, and VBP15-B-2, which has a 21-acetate substitution (vamorolone and VBP15-B-3 contain a 21-hydroxy moiety). Two validated and complementary *in silico* prediction methodologies were used for assessing mutagenic potential. The statistics-based quantitative structure-activity relationship (QSAR) program MultiCASE CASE Ultra was used, employing four different modules (GT1_A7B, GT1_AT_ECOLI, PHARM_ECOLI, and PHARM_SAL) designed to cover a wide range of molecular substructures collected from both proprietary and public compounds. In addition, the expert rule-based SAR program Derek Nexus was used to determine if the theoretical impurities contained structural alerts associated with known genotoxicants. CASE Ultra predicted both VBP15-B-2 and VBP15-B-3 as negative for mutagenicity [ReveraGen Report "In Silico Mutagenicity Evaluation of Delta 9,11 Epoxide Structures of VBP15: VBP15-B-2 (21-Acetate) and VBP15-B-3 (21-Hydroxy)"].

Taken together, these data indicate vamorolone has not generated a mutagenic signal based on these simulations.

1.3 Clinical Experience

1.3.1 Phase I Study in Healthy Adult Volunteers

Clinical experience is limited to a single Phase I clinical trial of vamorolone in healthy adult volunteers (VBP15-001). This study evaluated the safety, tolerability, and PK of vamorolone in a Phase I randomized, placebo-controlled, double-blind, single ascending dose (SAD) and multiple ascending dose (MAD) study. In the SAD portion of the study, Cohorts 1 through 5 and Cohort 7 were comprised of eight subjects each; six subjects in each cohort received a single oral dose of vamorolone (0.1 mg/kg, 0.3 mg/kg, 1.0 mg/kg, 3.0 mg/kg, 8.0 mg/kg, and 20 mg/kg, respectively) and two subjects in each cohort received placebo under fasted conditions. In Cohort 6, six subjects received a single oral dose of 8.0 mg/kg vamorolone within 30 minutes of beginning a high fat/high calorie meal. The MAD portion of the study had four cohorts (6 drug, 2 placebo in each) receiving 14 daily doses of vamorolone (1.0, 3.0, 9.0 and 20.0 mg/kg/day). The clinical conduct for all seven SAD cohorts and all four MAD cohorts has been completed.

The primary objectives of the Phase I study were to evaluate the safety and tolerability of single and multiple oral doses of vamorolone, and to evaluate the PK of single doses and multiple doses of vamorolone. A secondary objective was to evaluate the effect of food on the absorption and PK of vamorolone. Other objectives were to obtain samples from subjects on Day 1 (pre-dose) and Day 14 of the MAD cohorts for use in Metabolites in Safety Testing (MIST) assessments, and to test back-up PK samples from a subset of MAD subjects for pharmacodynamic (PD) biomarkers.

1.3.1.1 SAD Cohorts

1.3.1.1.1 SAD Cohorts – Pharmacokinetics Fasted

Vamorolone PK data shows strong adherence to dose linearity and dose proportionality, with relatively little subject-subject variation (**Figure 1, Table 1**, **Figure 2**). The half-life

was about 2 hours for doses 0.1-1.0 mg/kg. Doses at 3.0, 8.0 and 20.0 mg/kg showed an extended tail, increasing half-life to 2.5, 3.8 and 3.8 hours, respectively (**Figure 1**).

Figure 1 Arithmetic mean ± standard error plasma concentrations of vamorolone (VBP15) after oral administration of single doses of 0.1, 0.3, 1, 3, 8, and 20 mg/kg to healthy subjects under fasted conditions - linear plot (top); semi-logarithmic plot (bottom)

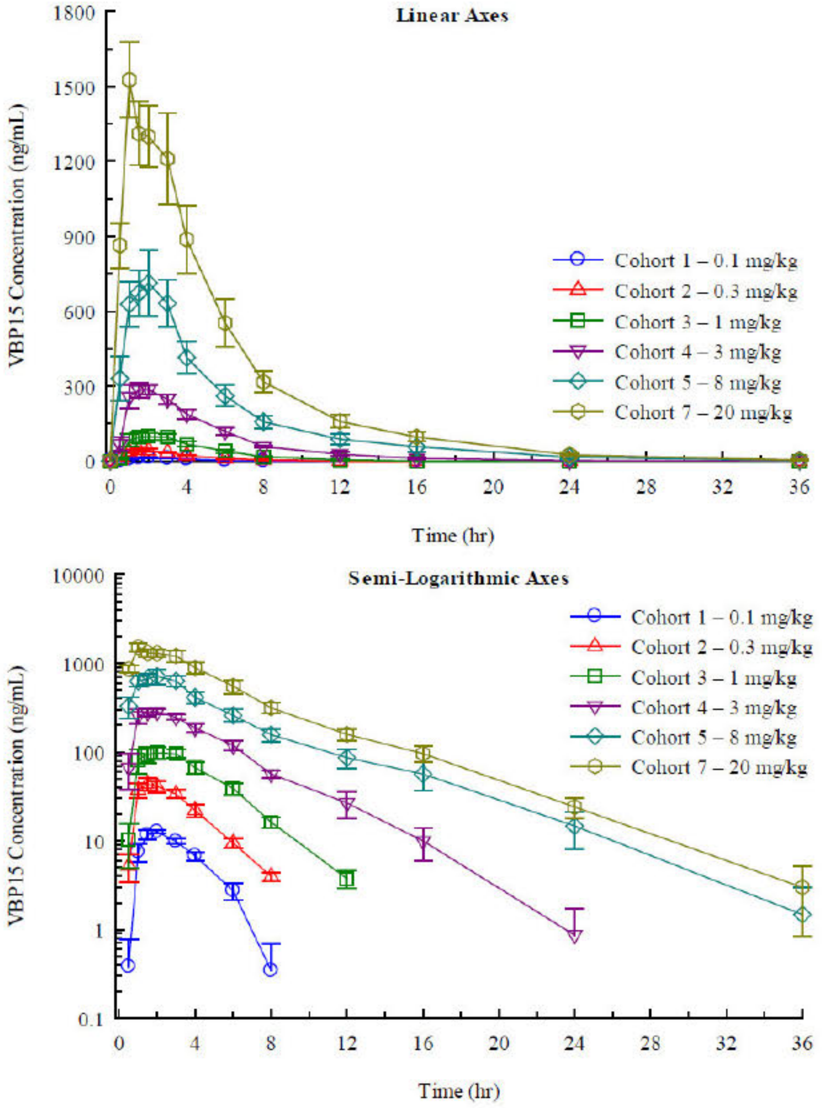

Table 1 Summary of pharmacokinetic parameters for vamorolone after oral administration of single doses of 0.1, 0.3, 1.0, 3.0, 8.0, and 20.0 mg/kg to healthy subjects under fasted conditions

	Dose					
Parameter*	0.1 mg/kg	0.3 mg/kg	1 mg/kg	3 mg/kg	8 mg/kg	20 mg/kg
Cmax (ng/mL)	13.1 (12.8) (6)	50.8 (16.5) (6)	122 (32.8) (6)	305 (24.4) (6)	718 (42.5) (6)	1,648 (16.7) (6)
Tmax (hr)	1.50 (6) [1.50 – 2.03]	1.50 (6) [1.00 – 3.00]	1.75 (6) [1.00 – 3.00]	1.75 (6) [1.00 – 2.00]	1.78 (6) [1.00 – 2.00]	1.50 (6) [1.00 – 3.03]
AUC(0-t) (hr×ng/mL)	41.9 (16.8) (6)	161 (15.9) (6)	486 (19.7) (6)	1,578 (20.7) (6)	3,997 (55.0) (6)	8,545 (29.5) (6)
AUC(inf) (hr×ng/mL)	49.5 (12.5) (6)	170 (16.5) (6)	500 (19.2) (6)	1,600 (20.3) (6)	4,137 (62.1) (5)	8,277 (37.2) (4)
λz (1/hr)	0.4064 (12.4) (6)	0.4376 (16.5) (6)	0.3829 (18.0) (6)	0.2792 (18.5) (6)	0.1823 (52.3) (5)	0.1827 (11.9) (4)
t½ (hr)	1.71 (12.4) (6)	1.58 (16.5) (6)	1.81 (18.0) (6)	2.48 (18.5) (6)	3.80 (52.3) (5)	3.79 (11.9) (4)
CL/F (L/hr/kg)	2.02 (12.5) (6)	1.76 (16.5) (6)	2.00 (19.2) (6)	1.88 (20.3) (6)	1.93 (62.1) (5)	2.42 (37.2) (4)
Vz/F (L/kg)	4.97 (6.06) (6)	4.03 (19.5) (6)	5.22 (19.1) (6)	6.72 (30.5) (6)	10.6 (57.8) (5)	13.2 (32.4) (4)

C_{max} = maximum observed plasma concentration; T_{max} = time to maximum observed plasma concentration; $AUC_{(0\text{-}t)}$ = area under concentration-time curve from time 0 to time t; $AUC_{(inf)}$ = area under concentration-time curve from time 0 to infinity; λ_z = elimination rate constant; $t_{½}$ = terminal half-life; CL/F = apparent total clearance from plasma; V_z/F = apparent volume of distribution during terminal phase.

Figure 2 Relationship between individual subject vamorolone AUC(inf) and dose after oral administration of single doses of 0.1, 0.3, 1, 3, 8, and 20 mg/kg to healthy subjects under fasted conditions

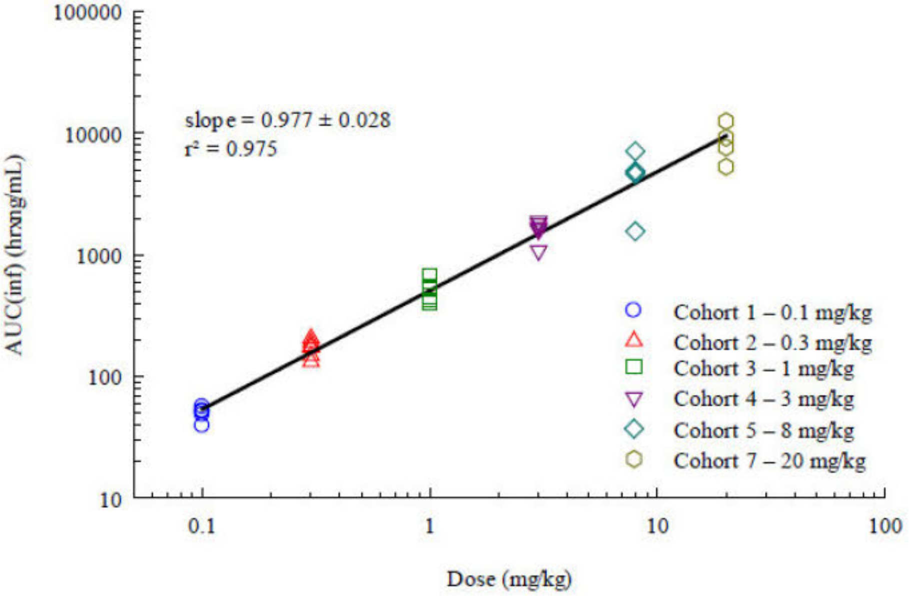

1.3.1.1.2 SAD Cohorts – Pharmacokinetics Fed

For the food effect group, a high fat meal (45 grams fat) was given to a cohort of Phase I SAD volunteers with the 8.0 mg/kg dose of vamorolone. These data were then compared to the fasted 8.0 mg/kg cohort data. This showed that absorption was increased by 2.5-fold by the high fat meal, consistent with the lipophilic character of vamorolone (steroidal compound) (**Figure 3**, **Table 2**).

Figure 3 Arithmetic mean ± standard error plasma concentrations of vamorolone (VBP15) after single dose oral administration of 8 mg/kg to healthy subjects under fed and fasted conditions - linear (top panel) and semi-logarithmic (bottom panel) axes

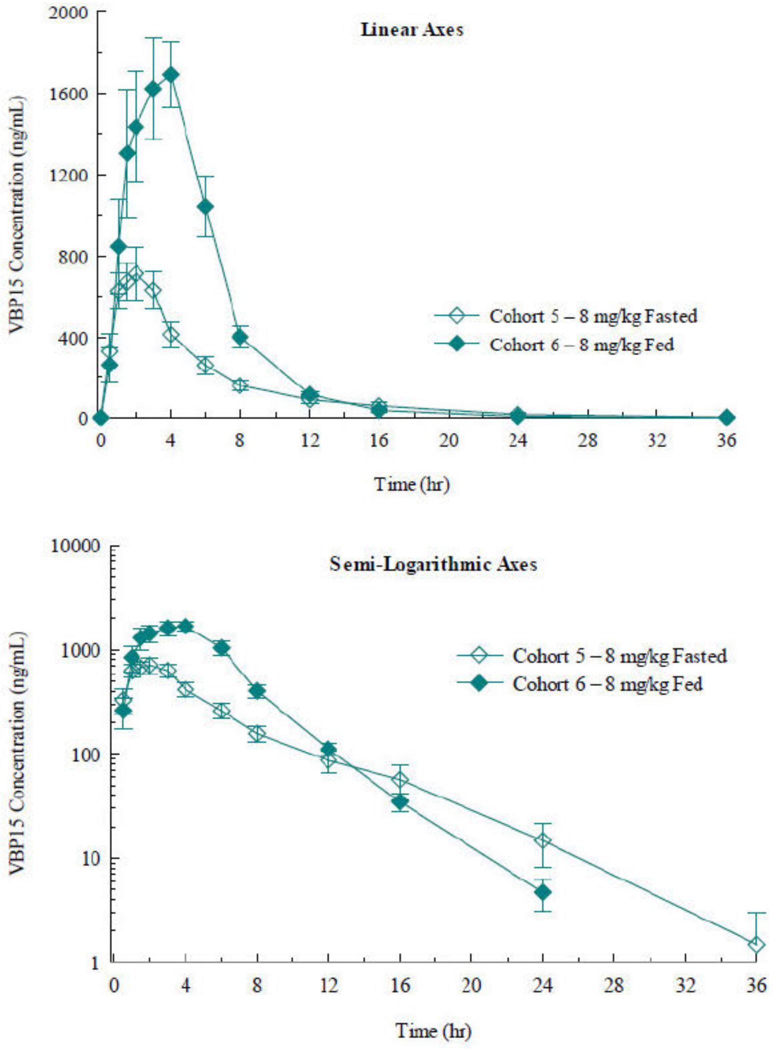

Table 2 Summary of pharmacokinetic parameters for vamorolone after single dose oral administration of 8 mg/kg to healthy subjects under fed and fasted conditions

Parameter*	8 mg/kg		Ratio†
	Fasted	Fed	
Cmax (ng/mL)	718 (42.5) (6)	1,817 (31.4) (6)	2.53
Tmax (hr)	1.78 (6) [1.00 – 2.00]	4.00 (6) [2.00 – 6.00]	
AUC(0-t) (hr×ng/mL)	3,997 (55.0) (6)	10,139 (25.1) (6)	2.54
AUC(inf) (hr×ng/mL)	4,137 (62.1) (5)	10,170 (24.9) (6)	2.46
λz (1/hr)	0.1823 (52.3) (5)	0.2950 (18.9) (6)	
t½ (hr)	3.80 (52.3) (5)	2.35 (18.9) (6)	
CL/F (L/hr/kg)	1.93 (62.1) (5)	0.79 (24.9) (6)	
Vz/F (L/kg)	10.6 (57.8) (5)	2.67 (23.4) (6)	

*Geometric mean (%CV) (N) except T_{max} for which the median (N) is reported.

†Ratio of the geometric means.

C_{max} = maximum observed plasma concentration; T_{max} = time to maximum observed plasma concentration; $AUC_{(0\text{-}t)}$ = area under concentration-time curve from time 0 to time t; $AUC_{(inf)}$ = area under concentration-time curve from time 0 to infinity; λ_z = elimination rate constant; $t_{½}$ = terminal half-life; CL/F = apparent total clearance from plasma; V_z/F = apparent volume of distribution during terminal phase.

1.3.1.1.3 SAD Cohorts – Adverse Events

One subject in the SAD 8.0 mg/kg showed a delayed mild elevation of liver enzymes. These elevations were not felt to be drug-related due to timing of the elevations. Unblinding revealed that the subject had received placebo. There were no other adverse events (AEs) seen in any dose group.

1.3.1.2 MAD Cohorts

The Phase I MAD treatment plan was discussed in light of the initial PK data. The relatively short half-life of vamorolone (2-4 hours), coupled with the planned daily dose schedule, would be expected to give PK data on each single dose, not cumulative dose, as the dosing interval was > 5 × $t_{½}$. Thus, the MAD component would be a study of individual daily doses, rather than dose-related accumulation and pharmacodistribution related to cumulative drug exposure. In other words, a typical goal of a MAD study is to determine steady state drug levels after multiple doses; yet with the short half-life of vamorolone, useful information would not be expected to be gained with the current daily

dosing schedule. Safety and tolerability are additional goals of the MAD study, and these remain important endpoints independent of the PK studies.

1.3.1.2.1 MAD Cohorts – Pharmacokinetics Fasted

The original design for the Phase I MAD was modified to remove the two lowest doses (0.1, 0.3 mg/kg/day), and to begin dosing at 1.0 mg/kg/day. The clinical conduct of all four cohorts has been completed (1.0 mg/kg/day, 3.0 mg/kg/day, 9.0 mg/kg/day, 20.0 mg/kg/day) for the MAD study (**Table 3**).

Table 3 Summary of pharmacokinetic parameters for vamorolone during oral administration of 1, 3, 9, and 20 mg/kg doses once daily for 14 days to healthy subjects under fasted conditions

	**Vamorolone Dose**			
	**1 mg/kg**	**3 mg/kg**	**9 mg/kg**	**20 mg/kg**
Day 1<br>C_{max} (ng/mL)	153 (15.9)	281 (36.9)	1,082 (23.3)	2,416 (51.1)
T_{max} (hr)	3.04<br>[1.50 – 4.00]	2.01<br>[1.00 – 3.00]	1.75<br>[1.00 – 6.00]	1.00<br>[0.50 – 3.00]
$AUC_{(0\text{-}t)}$ (hr×ng/mL)	686 (22.4)	1,471 (23.6)	5,709 (29.9)	10,182 (28.1)
$AUC_{(0\text{-}24)}$ (hr×ng/mL)	686 (22.4)	1,471 (23.6)	5,709 (29.9)	10,182 (28.1)
$AUC_{(inf)}$ (hr×ng/mL)	695 (22.1)	1,487 (23.7)	5,745 (29.5)	10,190 (27.0)
λ_z (1/hr)	0.3848 (10.9)	0.2918 (18.1)	0.2317 (22.6)	0.1747 (44.3)
$t_{½}$ (hr)	1.80 (10.9)	2.38 (18.1)	2.99 (22.6)	3.97 (44.3)
CL/F (L/hr/kg)	1.44 (22.1)	2.02 (23.7)	1.57 (29.5)	1.96 (27.0)
V_z/F (L/kg)	3.74 (16.9)	6.91 (34.8)	6.76 (46.9)	11.2 (77.6)
Day 14<br>C_{max} (ng/mL)	203 (30.1)	276 (35.6)	935 (48.3)	2,491 (27.9)
T_{max} (hr)	2.96<br>[1.50 – 3.00]	2.50<br>[1.00 – 4.00]	1.25<br>[0.55 – 3.00]	2.00<br>[1.00 – 2.00]
$AUC_{(0\text{-}24)}$ (hr×ng/mL)	794 (22.3)	1,494 (18.6)	4,366 (20.2)	9,309 (38.8)
λ_z (1/hr)	0.3993 (20.4)	0.3273 (25.2)	0.1629 (63.5)	0.1879 (31.6)
$t_{½}$ (hr)	1.74 (20.4)	2.12 (25.2)	4.25 (63.5)	3.69 (31.6)
CL/F (L/hr/kg)	1.26 (22.3)	2.01 (18.6)	2.06 (20.2)	2.15 (38.8)
V_z/F (L/kg)	3.15 (20.6)	6.14 (39.7)	12.7 (79.9)	11.4 (49.1)

C_{max} = maximum observed plasma concentration; T_{max} = time to maximum observed plasma concentration; $AUC_{(0\text{-}t)}$ = area under concentration-time curve from time 0 to time t; $AUC_{(0\text{-}24)}$ = area under concentration-time curve from time 0 to 24 hours; $AUC_{(inf)}$ = area under concentration-time curve from time 0 to infinity; λ_z = elimination rate constant; $t_{½}$ = terminal half-life; CL/F = apparent total clearance from plasma; V_z/F = apparent volume of distribution during terminal phase.

Taking into account the small numbers and different subjects, the geometric mean values for C_{max}, $AUC_{(0-t)}$, and $AUC_{(inf)}$ are not different for the SAD and MAD cohorts. Within the MAD, there is good agreement between Days 1 and 14 at all dose groups. There is no accumulation — the geometric mean C_{max} and $AUC_{(0-24)}$ on Days 1 and 14 are not different, consistent with the $t_{½}$ (~2 hour) and dosing interval (24 hours) (**Figure 4**; **Table 3**).

Figure 4 Arithmetic mean ± standard error plasma concentrations of vamorolone (VBP15) on Days 1 and 14 during oral administration of 1, 3, 9, and 20 mg/kg doses once daily for 14 days to healthy subjects under fasted conditions (linear axes)

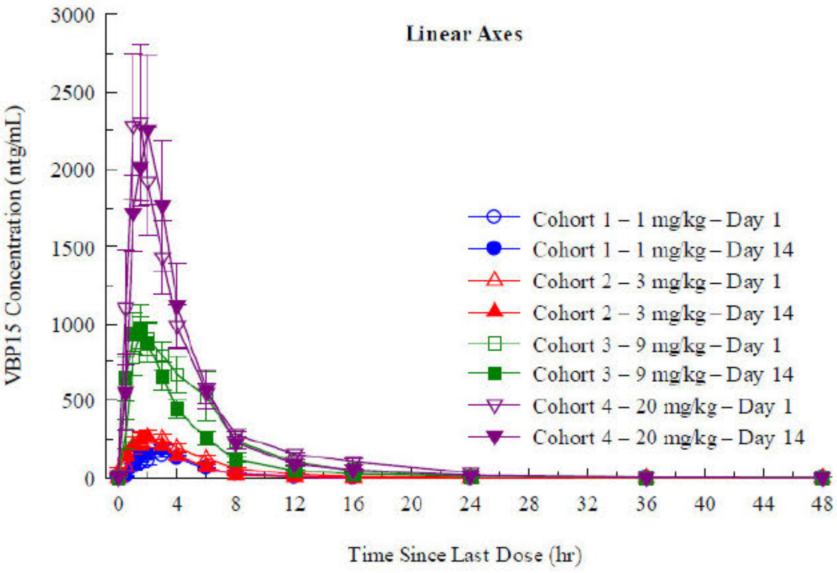

1.3.1.2.2 MAD Cohorts – Adverse Events

Three subjects have discontinued dosing; one subject (1.0 mg/kg/day group) withdrew consent on Day 10 so that he could receive care for an exacerbation of a previously existing dental condition. Dosing was discontinued in a second subject on Day 9 (on placebo in the 1.0 mg/kg/day cohort after completing 8 days of dosing) due to an increase in alanine aminotransferase (ALT). The subject's baseline value was 40 (normal ≤ 50); on Days 7, 8, and 9, it was 70, 86, and 106, respectively. Following cessation of dosing, the ALT has slowly declined and is continuing to be followed. Aspartate

aminotransferase (AST) was within normal limits with the exception of a value of 47 on Day 9 (normal ≤ 45), which was again normal the following day. Alkaline phosphatase, bilirubin, and coagulation parameters have remained within normal limits and the subject has remained asymptomatic. A third subject who was on active drug in the 20 mg/kg dose group was discontinued after 9 days of dosing due to an increase in ALT. The subject's baseline value was 43 (normal ≤ 50) at screening; on Days 7, 8, and 9, it was 56, 60, and 73, respectively. Following cessation of dosing, the ALT increased to 96 and 104 on Days 10 and 12; on Day 17, it had declined to 85. The subject continues to be followed and has remained asymptomatic. Aspartate aminotransferase was within normal limits with the exception of a value of 47 on Day 12 (normal ≤ 45); total bilirubin was 0.7 at screening (normal 0.2-1.2); it was increased at 1.4, 1.9, and 1.4 on Days 1, 2, and 3, respectively, and within normal limits for the remainder of dosing and follow-up. No subject in the 3.0 or 9.0 mg/kg/day cohorts had to discontinue dosing for an AE.

1.3.1.2.3 Pharmacodynamic Safety Biomarkers

Vamorolone has shown improved safety profiles relative to prednisone in nonclinical testing, both *in vitro* and *in vivo*.[15,17] Safety concerns with glucocorticoids include suppression of the adrenal axis and insulin resistance. Pharmacodynamic biomarker assays of suppression of the adrenal axis (serum cortisol) and insulin resistance (serum glucose) were measured in the Phase I MAD studies of vamorolone.

Suppression of the adrenal axis. Prednisone directly impinges on cortisol regulatory pathways (adrenal axis) both acutely and chronically. Acute suppression of adrenal function is seen within hours of doses of a single 0.1 mg/kg/day (approximate) dose of prednisone, as evidenced by reductions in adrenocorticotropic hormone (ACTH) levels in normal volunteers.[25] More chronic suppression of the adrenal axis, characterized as severe, is typically diagnosed when morning cortisol is < 100 nmol/L (< 3.6 microgram/dL) when drawn > 24 hrs after the last dose of pharmacological steroids.

Morning serum cortisol levels were measured in the vamorolone Phase I MAD cohorts, at baseline (prior to drug administration), 24 hours after the first dose (Day 1), and 24 hours after the 14-day dose (Day 15) (**Figure 5**). Active substance volunteers at four MAD

dose levels are shown (1.0 mg/kg/day; 3.0 mg/kg/day; 9.0 mg/kg/day; 20.0 mg/kg/day); all subjects were treated for 14 days with daily dosing. The red hatched line on each graph shows a typical threshold for adrenal axis suppression (< 100 nmol/L, or < 3.6 µg/dL). P values shown are for paired T test, indicating significance of the consistency of longitudinal changes of subjects relative to their own individual baseline values. Acute adrenal axis suppression is measured at 24 hours (after first dose), whereas chronic adrenal axis suppression is measured after 14 days of daily dosing (24 hours after last dose).

Figure 5 Morning cortisol measures in the vamorolone Phase I MAD volunteers.*

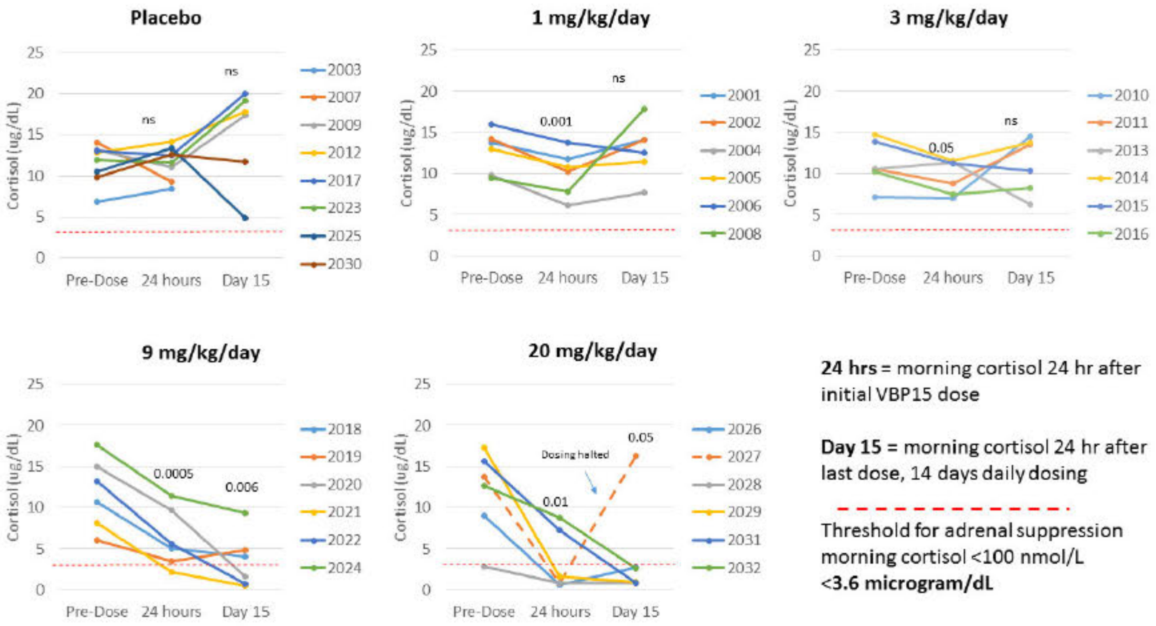

* Placebo subjects from each of the four MAD cohorts are graphed together.

Vamorolone showed little evidence of either acute (24 hour data), or chronic (Day 15 data) suppression of the adrenal axis at doses of either 1.0 mg/kg/day or 3.0 mg/kg/day. The data suggest that vamorolone induces variable, mild, acute and chronic suppression of the adrenal axis at 9.0 mg/kg/day, and stronger evidence of both acute and chronic adrenal axis suppression at 20.0 mg/kg/day. Prednisone typically shows both acute and chronic adrenal axis suppression approximately at 0.1 mg/kg/day,[25] suggesting that vamorolone has an improved safety window regarding adrenal axis suppression.

Vamorolone thus shows approximately a 100-fold improvement in safety window compared to prednisone on a mg/kg comparative basis. These data are consistent with *in vitro* and *ex vivo* nonclinical mouse data comparing VBP15/vamorolone to prednisone for adrenal suppression.[15]

Insulin resistance. Prednisone induces the safety signal of insulin resistance, where glucose is not efficiently taken up from the blood by target tissues, such as muscle and liver, leading to hyperglycemia.[25] Insulin resistance may be an important safety signal for dystrophic muscle, where the dysfunctional myofibers have been shown to have inadequate energy stores,[18,26] and insulin resistance likely limits availability of glycogen substrates for glycolysis. The hyperglycemia, in turn, leads to chronic increases in insulin levels (hyperinsulinemia).

Levels of fasting glucose and insulin are reasonably sensitive and reliable measures of insulin resistance in non-diabetic individuals. Glucose is acutely (single dose) and chronically (multiple doses) elevated after treatment with pharmacological glucocorticoids. Glucose is elevated 24 hours after a single administration of glucocorticoids (2.0 mg/kg).[27,28]

In the Phase I MAD of vamorolone, fasting serum glucose was measured at 10 time points during the 2-week study; each sample was taken 24 hours after the previous dose of vamorolone (**Figure 6**).

Figure 6 Fasting serum glucose during the Phase I MAD period (two weeks daily treatment)

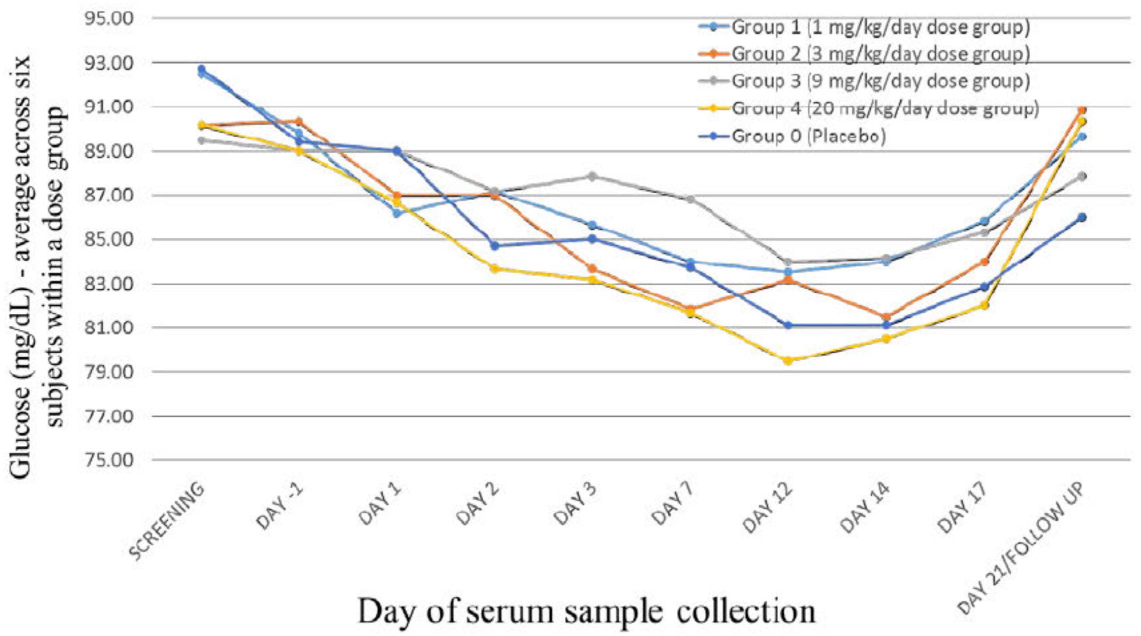

Glucose levels for all vamorolone dose groups were similar to those of the placebo group. There was no evidence of elevations of glucose levels at any time point or any dose of vamorolone, suggesting that the side effect of insulin resistance was not seen with vamorolone. These data are consistent with a nonclinical study in a dystrophin-deficient mouse model, where chronic treatment of prednisolone (5 mg/kg/day) versus vamorolone (15 mg/kg/day; 30 mg/kg/day) showed development of insulin resistance with prednisolone, but not vamorolone.[29]

Bone remodeling. Glucocorticoid side effects impacting bone cause significant morbidity. Extensively studied in older adults where glucocorticoids are often given for arthritis and other age-related chronic inflammatory states, adjusted relative rate of non-vertebral fractures shows a dose-dependent increase to 2.5 times non-treated adults, and this is directly related to glucocorticoid-induced osteopenia.[30] The bone effects in glucocorticoid-treated patients with DMD can be even greater because the standard of care for boys with DMD is chronic treatment with double the highest glucocorticoid dose typically used in older adults.[31] Fractures due to osteoporosis are common in patients chronically treated with glucocorticoids, both in older adult populations (e.g. arthritis)

and in the pediatric population (e.g. Duchenne muscular dystrophy; DMD). Bone safety is a significant cause of clinical morbidity, including vertebral fractures that can lead to chronic back pain and spinal deformity, and long bone fractures that have been linked to permanent, premature loss of ambulation.[32]

Because the initiation of glucocorticoid treatment is recommended in early childhood for patients with DMD, the adverse effects of glucocorticoids are amplified through effects on bone cellular metabolism, bone growth and puberty. Osteoporosis, manifesting as low-trauma vertebral or long bone fractures, is frequent in DMD.[33] This is not surprising given the potent osteotoxicity of glucocorticoid therapy combined with the progressive myopathy, both of which are key risk factors for reduced bone strength. Studies have reported that 20 to 60% of boys with DMD have low-trauma extremity fractures (usually of the distal femur or tibia/fibula) while up to 30% develop symptomatic vertebral fractures during the pediatric years.[34,35,36] The number of symptomatic vertebral fractures that have been reported to date suggests the true vertebral fracture prevalence is likely higher, since vertebral fractures are frequently asymptomatic when identified in children with other glucocorticoid-treated illnesses through a monitoring program that includes a lateral spine radiography[37]. Untreated, vertebral fracture lead to chronic back pain and spine deformity, while leg fractures can cause premature, permanent loss of ambulation.[25] Death due to fat embolism syndrome and subsequent respiratory distress after long bone fractures in both ambulant and non-ambulant patients has also been reported in DMD.[38,39] A recent study found that osteoporosis treatment with bisphosphonates may improve survival in DMD, emphasizing the urgency for effective osteoporosis diagnosis, monitoring and prevention strategies.[40]

Bone turnover markers are often used to monitor effects of drugs and other interventions on bone health. Serum osteocalcin is considered a biomarker for bone formation, where prednisone decreases serum levels, and this is correlated with bone health outcomes. Serum CTX1 (C-terminal telo peptide of Collagen 1) is a marker of bone reabsorption, prednisone causes increases in serum levels, and these are also correlated with later bone health outcomes. Osteocalcin and CTX1 serum levels were measured in the Phase I MAD groups at the following four time points: Within 2 hours prior to dosing (pre-dose);

8 hours postdose day 1 (8 hr); 1 day after 14 days of dosing (14 day); 3 days after last dose (Rec) (Table 4, Table 5, Figure 7). The results showed no significant changes of bone turnover markers through the highest vamorolone dose tested compared to placebo (20 mg/kg/day vamorolone). These results are in contrast to prednisone, where two independent studies showed significant changes in these same biomarkers at all doses tested, to 0.25 mg/kg/day.[25,41]

Figure 7 Comparisons of serum osteocalcin and CTX1 show no differences of placebo compared to 20 mg/kg/day vamorolone. Within 2 hours prior to dosing (pre-dose); 8 hours postdose day 1 (8 hr); 1 day after 14 days of dosing (14 day); 3 days after last dose (Rec)

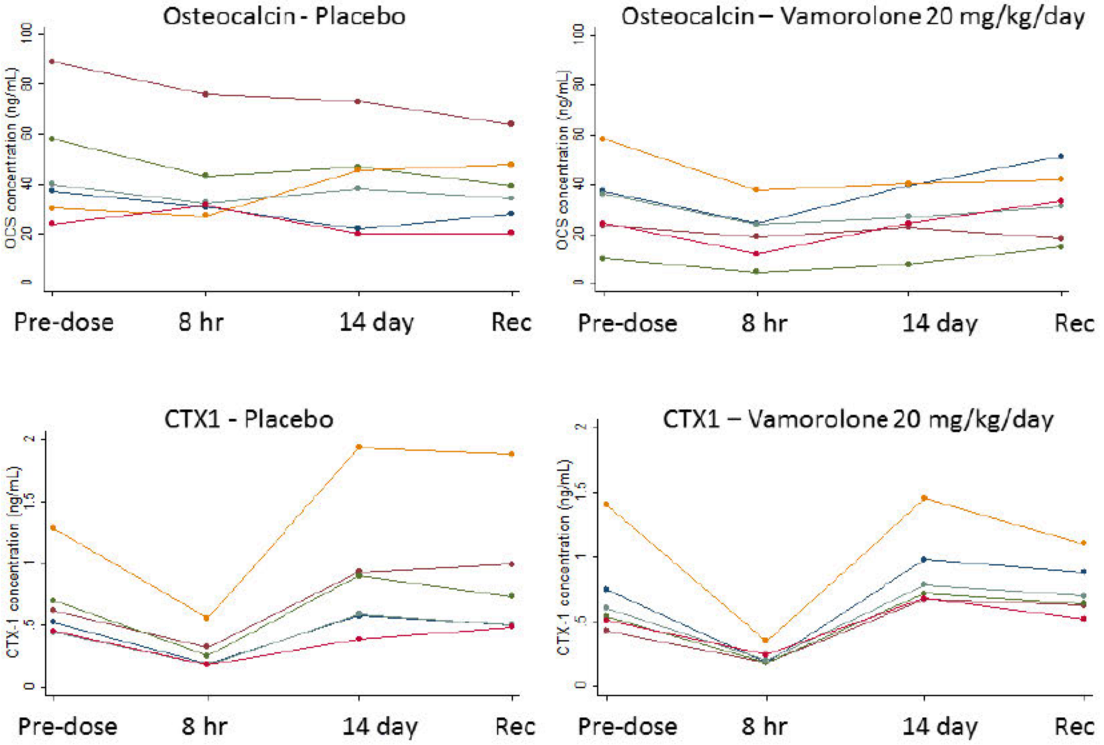

Table 4 Serum osteocalcin concentrations (ng/ml) in vamorolone Phase I MAD groups. Within 2 hours prior to dosing (pre-dose); 8 hours postdose day 1 (8 hr); 1 day after 14 days of dosing (14 day); 3 days after last dose (Rec)

Dose vamorolone	Osteocalcin (ng/ml): Time Point			
	Predose	8 hr	14 days	Rec
Placebo	46.3 ± 9.7	40.1 ± 7.4	40.8 ± 7.9	38.8 ± 6.2
1.0 mg/kg	33.2 ± 11.5	27.4 ± 9.3	28.8 ± 9.3	25.9 ± 8.0
3.0 mg/kg	13.7 ± 11.6	13.6 ± 11.5	16.1 ± 12.5	13.9 ± 9.5
9.0 mg/kg	32.9 ± 8.6	20.4 ± 4.7	26.7 ± 5.2	33.5 ± 7.2
20.0 mg/kg	31.5 ± 6.7	20.2 ± 4.6	26.9 ± 4.9	31.8 ± 5.6

Table 5 Serum CTX1 concentrations (ng/ml) in vamorolone Phase I MAD groups. Within 2 hours prior to dosing (pre-dose); 8 hours postdose day 1 (8 hr); 1 day after 14 days of dosing (14 day); 3 days after last dose (Rec)

Dose vamorolone	CTX1 (ng/ml): Time Point			
	Predose	8 hr	14 days	Rec
Placebo	0.66 ± 0.12	0.28 ± 0.05	0.88 ± 0.22	0.84 ± 0.22
1.0 mg/kg	0.50 ± 0.11	0.22 ± 0.02	0.68 ± 0.16	0.55 ± 0.09
3.0 mg/kg	0.31 ± 0.05	0.18 ± 0.00	0.48 ± 0.10	0.46 ± 0.12
9.0 mg/kg	0.59 ± 0.11	0.19 ± 0.006	0.77 ± 0.13	0.60 ± 0.10
20.0 mg/kg	0.70 ± 0.14	0.22 ± 0.02	0.87 ± 0.12	0.66 ± 0.12

1.3.1.3 Summary of Phase I data

In summary:

- Vamorolone PK data show strong adherence to dose linearity and dose proportionality, with relatively little subject-subject variation (both SAD and MAD).
- The half-life was about 2 hours for doses 0.1-1.0 mg/kg. Doses at 3.0, 8.0, and 20.0 mg/kg showed an extended tail, increasing half-life to 2.5, 3.8, and 3.8 hours, respectively. The PK for the MAD cohorts was very similar to the SAD cohorts, showing little if any drug accumulation, consistent with the short half-life and daily dosing schedule.

- For the food effect group, a high fat meal was given to a cohort of Phase I SAD volunteers with the 8.0 mg/kg dose of vamorolone. These data were then compared to the fasted 8.0 mg/kg cohort data. The comparison showed that absorption was increased by 2.5-fold by the high fat meal, consistent with the lipophilic character of vamorolone (steroidal compound).
- For the MAD cohorts, there were no AEs precluding further escalations in dosing. The anticipated therapeutic dose in DMD boys is estimated between 1.0-8.0 mg/kg/day, and this is the dose range proposed in the later Phase II first-in-patient studies.
- Regarding the primary target organ, liver, one subject on placebo in the 1.0 mg/kg/day cohort and one subject on vamorolone in the 20 mg/kg/day cohort showed mild elevations of liver enzymes, and drug dosing was halted. No subjects in the 3.0 or 9.0 mg/kg/day MAD cohorts showed elevations of liver enzymes.
- Safety PD biomarker studies showed that vamorolone had an improved safety window for adrenal axis suppression (100-fold increase in therapeutic window), no evidence of insulin resistance, no changes in bone turnover markers (osteocalcin, CTX1), compared to prednisone studies reported in the literature.[25,41]

The results show that the half-life of vamorolone is similar to glucocorticoids, such as prednisone. Despite the short half-life, prednisone is typically given once per day for most indications (including DMD), and once daily dosing is also proposed for vamorolone. Previous studies of increasing the frequency of drug administration of glucocorticoids have shown that this has increased side effect profiles without a significant gain in efficacy. Thus, the mechanism of action of glucocorticoids may be related to the short-term (pulsed) daily drug exposure. As vamorolone is thought to share a similar anti-inflammatory mechanism of action as glucocorticoids (NF-κB inhibition), it is felt that the daily pulsed exposure in the Phase I MAD is most relevant to the planned clinical trials in DMD including the Phase II MAD and Phase II efficacy/safety studies, where daily dosing schedules will be followed. The option of increasing the dosing regimen in the Phase I MAD to better study drug accumulation and steady state PD was

considered, but this was felt to be unrelated to planned Phase II trials, and drug mechanism of action.

1.3.2 Pharmacokinetics in Phase II Study in 4 to 7 years Duchenne Muscular Dystrophy Boys (VBP15-002)

<u>Preliminary Assessment of Vamorolone (VBP15) Pharmacokinetics in Boys with DMD:</u>

Pharmacokinetics has been studied in six boys with DMD received 0.25 mg/kg oral doses of VBP15 once daily for 14 days. The drug was administered with milk. Six blood samples were collected at 0, 1, 2, 4, 6, and 8 hours and plasma was analyzed for VBP15. The time-course of plasma drug concentrations were assessed using noncompartmental (NCA) methods using the WinNonlin software (Certera). Selected exposure parameters from this limited sampling schedule were compared with those from healthy adult men from Study VBP15-001, who received a range of single and multiple oral doses of VBP15.

The graphs displayed in **Figure 8** depict the plasma concentration versus time profiles for all subjects on Days 1 and 14. The sampling schedule reasonably captured the absorption (upcurve) and disposition (downcurve) profiles with peak concentrations occurring at 2 to 4 hours after dosing. The $\mathbf{C}_{max}$ averaged 18.9 (CV% = 46) on Day 1 and 37.9 (42%) ng/mL on Day 14. The Area Under the Curve (**AUC**) with extrapolation averaged 87.2 (37%) on Day 1 and 147.9 (27%) on Day 14. As the zero-time concentrations of VBP15 were undetectable before dosing on Day 14 and the half-life of the drug was relatively short (5.9 hr on Day 1 and 3.55 hr on Day 14), the increase in concentrations from Day 1 to Day 14 was not likely due to accumulation. Increased bioavailability is more likely. As the Day 14 exposures are most relevant, a comparison of these plasma concentrations with data from adult men will be made.

Figure 8 Pharmacokinetics of 0.25 mg/kg/day vamorolone in 4-7 years DMD boys

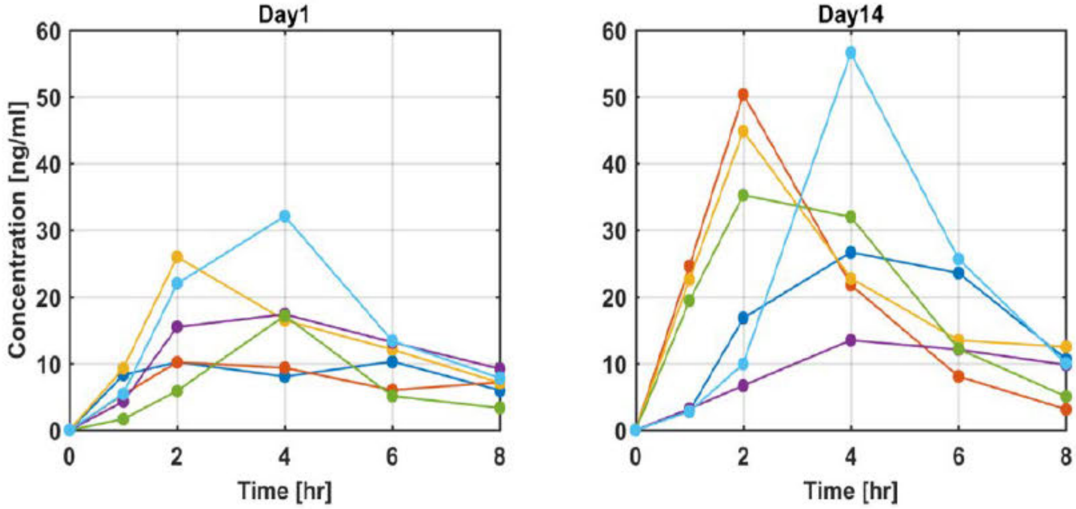

A listing of the NCA pharmacokinetic parameters for the data from Day 14 is provided for the 6 subjects in the table. It should be noted that the clearance values (CL) are apparent since the bioavailability of the drug in these subjects is uncertain.

Table 6 Pharmacokinetics of 0.25 mg/kg/day vamorolone in 4-7 years DMD boys

Subject	Cmax [ng/mL]	Tmax [hr]	AUC [hr.ng/ml]	t1/2 [hr]	CL [L.hr/kg]	CL [L/hr]
233401	26.7	4	137.59	2.65	1.400	36.26
233402	50.4	2	156.17	1.46	1.536	39.01
233403	44.9	2	171.75	5.71	0.910	24.20
233405	13.5	4	74.17	8.39	1.297	26.71
233406	35.3	2	161.62	1.55	1.445	33.24
233407	56.6	4	185.88	1.53	1.202	22.72

➢ Mean data statistics

Dose [mg/kg]	Cmax [ng/mL] (CV%)	Tmax [hr] (CV%)	AUC [hr.ng/ml] (CV%)	t1/2 [hr] (CV%)	CL [L/hr/kg] (CV%)
0.25	37.9 (42.3)	3 (36.5)	147.86 (26.7)	3.55 (81.1)	1.298 (17.2)

The PK of VBP15 did not change from Day 1 to Day 14 in healthy men. The following PK metrics were observed in 6 healthy men who received a single dose of 0.3 mg/kg of the drug under fasting conditions:

C_{max} = 50.8 (16.5%); **T_{max}** = 1.5 hr; **AUC** = 170 (16.5%) ng.hr/mL; **$t_{1/2}$** = 1.60 (17.8%) hours; **CL/F** = 1.76 (15.5%) L/hr/kg.

It can be observed that most of these metrics are quite comparable between adult men and boys with DMD considering that the dose was about 20% lower in the latter group. While normal children typically metabolize drugs somewhat faster than adults (clearance usually relates to body weight as **CL** = a.BW**0.75 where "a" is a specific constant for each drug), the DMD boys may have somewhat low clearances. The drug exhibits markedly increased absorption when ingested with food in adults. Thus dosing of the drug with milk in the DMD boys may produce increased bioavailability as well and produce a higher **AUC** and lower apparent **CL/F**. It can be noted that the mean half-life (**$t_{1/2}$**) appears much shorter in adults (1.6 hr) than in DMD boys (3.55 hr). It is likely that the longer mean **$t_{1/2}$** is due to prolonged absorption of the drug in 3 subjects as 3 of the DMD boys had a more reasonable **$t_{1/2}$** of about 1.5 hours. It appears that the variability in the PK metrics is slighter greater in the DMD boys than in adults. However, these parameters are expected to be more accurate in the adults owing to collection of more time samples (n = 13) versus 6 in the DMD boys.

Conclusion:

While the increased exposures of the 0.25 mg/kg doses VBP15 taken with milk on Day 14 compared to Day 1 are unexpected, the PK metrics observed on Day 14 in DMD boys are remarkably consistent with those from adult men who received single fasting doses of 0.3 mg/kg. As VBP15 exhibits linear and consistent pharmacokinetics in adult men, increasing the dose in DMD boys should also produce predictably consistent increases in exposures.

1.3.3 Safety in Phase II Studies in 4 to7 years Duchenne Muscular Dystrophy Boys (VBP15-002 and VBP15-003)

Adverse events: There were no serious adverse events (SAEs) reported over the 14-day treatment in the Phase I clinical trial in healthy adult volunteers, nor in the four cohorts (0.25 mg/kg, 0.75 mg/kg, 2.0 mg/kg, and 6.0 mg/kg) of the Phase IIa study (VBP15-002; 14-day treatment) in boys ages 4 to <7 years with DMD. There has been a total of

4 SAEs in the Phase IIa VBP15-003 study and one SAE to date in the VBP15-LTE extension study: two SAEs of pneumonia in two different subjects (both subjects receiving vamorolone 0.75 mg/kg/day), one SAE of bilateral testicular torsion and one SAE of hypoxia in the same subject receiving 6.0 mg/kg/day, and one SAE of influenza associated dehydration in a subject receiving 6.0 mg/kg/day. Each of these SAEs was considered unrelated to study drug, and none of them resulted in discontinuation from the study. In the VBP15-003 study, there were a total of 218 TEAEs among 42 of the 48 subjects (87.5%). In VBP15-003 study, the TEAEs with the highest incidence were viral upper respiratory tract infection (41.7%); pyrexia (35.4%); cough (18.8%); vomiting (14.6%); and diarrhea (10.4%).

Body Mass Index (BMI): Body Mass Index was measured throughout the VBP15-003 study. The mean change from baseline to Week 24 for BMI was 0.03, 0.20, 0.23, and 1.15 for the 0.25, 0.75, 2.0, and 6.0 mg/kg/day dose level groups, respectively. Body Mass Index increases generally reflect an increase in weight. Body Mass Index z-score was monitored in the VBP15-003 study. The mean change from baseline to Week 24 for BMI z-score for the 6.0 mg/kg/day group showed a statistically significant increase compared to the mean change from baseline to Week 24 for the 0.25 mg/kg/day and 0.75 mg/kg/day dose level groups. In contrast, the mean change from baseline to Week 24 for the 2.0 mg/kg/day dose level group in BMI z-score was minimal and comparisons with the other vamorolone dose level groups lacked statistical significance. The mean increase from baseline to Week 24 for BMI z-score was similar for the 6.0 mg/kg/day group and a daily prednisone-treated historical control group.

Potential liver toxicity: In the Phase I clinical trial in adult volunteers, vamorolone showed mild elevations of liver enzymes in one subject receiving 20.0 mg/kg in the fasted state, and dosing was halted. All DMD subjects have elevated serum ALT and AST enzymes because of the muscle condition. For that reason, two enzymes, glutamate dehydrogenase (GLDH) and gamma glutamyl transferase (GTT), that are preferentially expressed in liver were evaluated in the VBP15-003 study. None of the mean changes in GLDH from baseline to any of the VBP15-003 on-treatment assessment time points were statistically significant for any dose level group. Although the mean changes from

baseline to Week 8, Week 16, and Week 24 in GLDH levels did not show a dose response, shift analysis of GLDH levels did suggest a possible dose-related shift to higher GLDH levels at 2.0 mg/kg/day and 6.0 mg/kg/day at Weeks 16 and 24. Mean GGT levels and individual subject values at each VBP15-003 assessment time point across the four dose level groups remained at or below the normal range. On the basis of these mean GLDH and GGT data, vamorolone at dose levels up to 6.0 mg/kg/day does not appear to induce liver toxicity over a 24-week treatment period.

Adrenal suppression: In the VBP15-003 study, after 24 weeks of treatment, 0 of 8 tested participants (0.25 mg/kg/day), 1 of 12 (8.3%) tested participants (0.75 mg/kg/day), 5 of 12 (41.7%) tested participants (2.0 mg/kg/day), and 8 of 9 (88.9%) tested participants (6.0 mg/kg/day) had a depressed morning cortisol (<3.6 μg/dL [100 nM]) consistent with chronic adrenal suppression.[29]

Insulin resistance: In the VBP15-003 study, mean changes from baseline for fasting insulin showed dose-and time-related changes for all dose level groups at Week 12 and Week 24. Statistical significance was observed for mean increase from baseline for the 6.0 mg/kg/day dose level group at Week 12 and Week 24.

Bone Turnover: In the VBP15-003 study, pharmacodynamic biomarker testing for bone turnover markers suggested that vamorolone does not have the detrimental bone effects observed with prednisone and deflazacort.

1.4 Rationale for Study Design

Vamorolone is under development for the treatment of DMD. The first-in-human clinical assessment of vamorolone is ongoing in the SAD/MAD study (VBP15-001) and is providing an initial clinical assessment and assessment of the PK characteristics of vamorolone following administration of an oral suspension of vamorolone in healthy adult subjects. While there are likely to be some differences between adults and children, and between healthy and DMD subjects, the data from VBP15-001 have established the 2-4 hour half-life of vamorolone, food effect, dose proportionality and variability among subjects.

A Phase IIa Multiple Ascending Dose study (VBP15-002) will determine the safe and tolerable dose(s) of vamorolone to enable future studies with chronic administration in DMD subjects, aged 4-7 years. The narrow age window for this study is driven by several factors: 1) the average age of diagnosis is still approximately 4.8 years due to lack of screening programs; 2) the current standards of clinical care in Western countries, where glucocorticoid therapy is typically initiated between 5 and 7 years of age for DMD patients; and 3) the observation that peak function for DMD patients occurs at around 7 years of age (after which time most affected males with DMD will begin to decline), all of which result in a narrow age window to identify steroid-naive males with DMD.

Key safety parameters in conjunction with PK data are being evaluated during the course of the Phase IIa study to assess vamorolone safety and tolerability. Exploratory clinical efficacy parameters and PD biomarkers of tissue breakdown and repair are also being evaluated.

The vamorolone development program has made advances in biomarker discovery and development in DMD, with a subset of these studies recently published.[42] A panel of biomarkers in DMD patient sera that show response to chronic (~4 month) treatment with glucocorticoids has been identified. These chronic prednisone PD biomarkers were discovered through study of serum samples of DMD patients enrolled in the Cooperative International Neuromuscular Research Group (CINRG) Duchenne Natural History Study (DNHS), and data have been reported for the VBP15-002 study.[43] SOMAscan aptamer panels testing 1,200 serum proteins were used to discover a candidate set of prednisone-responsive biomarkers, with a subset of these validating in a longitudinal sample set (individual DMD patients pre/post steroid treatment). These PD biomarkers were assigned to a safety panel or efficacy panel based on comparison to normal controls and information concerning the function of each protein (**Table 7**). All safety biomarkers were validated in a separate cohort of pediatric inflammatory disease patients (longitudinal pre/post steroids). The same SOMAscan assay was used on the chronic samples from the vamorolone Phase IIa trial (entry, 2 weeks treatment, 2 weeks washout), with initial data analyses limited to those validated biomarkers shown (**Table 7**).

Table 7 Biomarkers previously defined to be responsive to chronic doses of glucocorticoids in DMD boys

Chronic safety	Assay method	Chronic efficacy	Assay method
MMP-3	SOMAscan	CD23	SOMAscan
Leptin	SOMAscan	MDC	SOMAscan
Insulin	SOMAscan	IL-22BP	SOMAscan
IGFBP-5	SOMAscan	Lymphotoxin a1/b2	SOMAscan
Angiotensinogen	SOMAscan	IGFBP-2	SOMAscan
Afamin	SOMAscan	Integrin a1b1; CD49a	SOMAscan
17-hydroxyprogesterone	LC-MS	MMP-12	SOMAscan
Corticosterone	LC-MS		
Testosterone	LC-MS		
11-deoxycortisol	LC-MS		

Adrenal axis suppression is a well-documented safety signal with chronic use of glucocorticoids. As noted in Section 1.3.3, in the VBP15-003 study, after 24 weeks of treatment, 0 of 8 tested participants (0.25 mg/kg/day), 1 of 12 (8.3%) tested participants (0.75 mg/kg/day), 5 of 12 (41.7%) tested participants (2.0 mg/kg/day), and 8 of 9 (88.9%) tested participants (6.0 mg/kg/day) had a depressed morning cortisol (<3.6 μg/dL [100 nM]) consistent with chronic adrenal suppression. To further evaluate the effects of vamorolone on chronic adrenal axis suppression, four steroid hormones (17-hydroxyprogesterone, corticosterone, testosterone, 11-deoxycortisol) are being analyzed by LC-MS in the VBP15-LTE study (Table 7). All four steroid hormones showed significant reductions after glucocorticoid treatment in DMD boys in the cross-sectional and longitudinal CINRG DNHS participants.

The current long-term extension study (VBP15-LTE) is designed to provide long-term safety, efficacy, and PD data for the vamorolone dose(s) that are recognized as safe and well-tolerated in the Phase IIa (VBP15-002) and Phase IIa extension (VBP15-003) core studies. Enrollment in this VBP15-LTE long term extension study will be offered to subjects who have completed VBP15-003. VBP15-LTE extends the treatment duration and increases the potential for direct benefit of study treatment to these pediatric DMD subjects.

Subjects enrolling into the VBP15-LTE study will remain on the same dose that they were on at the time they completed the Week 24 Final Visit of the VBP15-003 Phase IIa extension study (i.e.. one of four doses: 0.25 mg/kg/day, 0.75 mg/kg/day, 2.0 mg/kg/day, or 6.0 mg/kg/day). Subjects, after they have been on their initial dose for at least 1 month in VBP15-LTE may be escalated to the next higher dose level at the discretion of the Study Chair and Medical Monitor once the next higher dose has been determined to be safe in the VBP15-002 Phase IIa Study, and no safety issues with that dose have emerged in the VBP15-003 Phase IIa extension study.

Comparisons of efficacy and safety parameters with historical natural history (untreated)[44,45,46,47] and prednisone- and deflazacort-treated control groups[48] will be performed as outcomes of this VBP15-LTE study.

This trial will be conducted in compliance with this protocol, Good Clinical Practice (GCP), applicable Food and Drug Administration (FDA) requirements, and the recently issued FDA guidance on developing drugs for treatment for Duchenne muscular dystrophy and related dystrophinopathies.[49]

It is obligatory that the Investigator become familiar with all sections of the Vamorolone Investigator's Brochure.[29]

1.5 Overall Benefit/Risk

The current study is a long-term safety and efficacy study in young boys with DMD. It is anticipated that the adverse effect profile of the investigational product will be more favorable than standard of care glucocorticoids in the long term (see Section 1.3.3). The adverse effects of vamorolone over long term treatment are not currently known.

Instructions for detecting adrenal crisis and the circumstances in which stress dose steroids should be provided are included in the informed consent form (ICF), and Investigators should monitor clinical study participants closely to identify elevations in liver-specific enzymes.

Potential health benefits: Subjects may or may not receive direct health benefit from participating in the study. Subjects will receive vamorolone at one of four planned dose

levels (0.25 mg/kg/day, 0.75 mg/kg/day, 2.0 mg/kg/day, or 6.0 mg/kg/day) over the course of the 24-week VBP15-003 trial, and then continue on this dose in the current VBP15-LTE study for up to 2 years. As noted above, the Medical Monitor, Study Director, and Site Investigator may opt to dose escalate to a higher dose level during the VBP15-LTE once the subject has been on the initial dose in VBP15-LTE for at least one month, the next higher dose is determined to be safe in the VBP15-002 Phase IIa Study, and no safety issues with that dose have emerged in the VBP15-003 Phase IIa extension study. In the VBP15-003 study, clinical efficacy was assessed by Timed Function Tests. Improvement in Time to Stand, Time to Climb, Time to Run/Walk 10 Meters, and 6-Minute Walk Test were seen predominantly for the 2.0 and 6.0 mg/kg/day dose level groups, with many of the improvements showing statistical significance compared to an untreated Duchenne Natural History Group. In view of the initial clinical evidence of safety, the improvements seen in assessments of efficacy, and the monitorable nature of key potential adverse effects, , data support an acceptable benefit/risk profile for vamorolone.

2 STUDY OBJECTIVES AND ENDPOINTS

2.1 Study Objectives

2.1.1 Primary Objectives

The primary objectives of this study are:

1. To evaluate the long-term safety and tolerability of vamorolone, administered orally at daily doses up to 6.0 mg/kg over a 24-month Treatment Period, in young boys with DMD who completed protocol VBP15-003; and
2. To compare the efficacy, as measured by the Time to Stand Test (TTSTAND), of vamorolone administered orally at daily doses up to 6.0 mg/kg over a 24-month Treatment Period vs. untreated DMD historical controls in young boys with DMD.

2.1.2 Secondary Objectives

The secondary objectives of this study are:

1. To investigate the effects of vamorolone, administered orally at daily doses up to 6.0 mg/kg over a 24-month Treatment Period on serum pharmacodynamic (PD) biomarkers of safety (insulin resistance, adrenal axis suppression, and bone turnover);
2. To investigate the effects of vamorolone, administered orally at daily doses up to 6.0 mg/kg over a 24-month Treatment Period, on muscle strength, mobility and functional exercise capacity vs. untreated DMD historical controls as measured by Time to Run/Walk Test (TTRW), North Star Ambulatory Assessment (NSAA), Time to Climb Test (TTCLIMB), 6-minute Walk Test (6MWT), and Quantitative Muscle Testing (QMT) in young boys with DMD; and
3. To compare the safety, as assessed by bone age, spine fractures, BMI z-score, and height z-score, of vamorolone administered orally at daily doses up to 6.0 mg/kg over a 24-month Treatment Period vs. prednisone- and deflazacort-treated historical control boys with DMD.

2.1.3 Exploratory Objectives

The exploratory objectives of this study are:

1. To investigate the effects of vamorolone administered orally at daily doses up to 6.0 mg/kg over a 24-month Treatment Period on Quality of Life measures (Pediatric Outcomes Data Collection Instrument [PODCI]);
2. To investigate the effects of vamorolone administered orally at daily doses up to 6.0 mg/kg over a 24-month Treatment Period on additional exploratory PD biomarkers; and
3. To determine if established genetic modifiers of DMD (gene polymorphisms associated with disease severity, or response to glucocorticoid treatment) are similarly associated with vamorolone-treated DMD patients (baseline disease severity, or response to vamorolone treatment).

2.2 Study Endpoints

2.2.1 Safety Endpoints

1. BMI z-score: Comparison with prednisone- and deflazacort-treated historical control groups for change from Baseline to Month 12 and Month 24;
2. BMI z-score: Change from Baseline to each of the scheduled on-treatment and post-treatment assessment time points;
3. Height z-score: Comparison with prednisone- and deflazacort-treated historical control groups for change from Baseline to Month 12 and Month 24;
4. Treatment-emergent adverse events (TEAEs) and serious adverse events (SAEs) by system organ class (SOC): Overall by treatment, by treatment and relationship, and by treatment and intensity (see Section **7.2.6**);
5. Vital signs [blood pressure, heart rate, respiratory rate, oral body temperature]: Change from Baseline to each of the scheduled on-treatment and post-treatment assessment time points;
6. Body weight: Change from Baseline to each of the scheduled on-treatment and post-treatment assessment time points;
7. Clinical laboratory values (hematology and biochemistry): Change from Baseline to each of the scheduled on-treatment and post-treatment assessment time points;
8. Lipid profile (triglycerides, total cholesterol, low density lipoprotein [LDL], high density lipoprotein [HDL]): Change from Baseline to each of the scheduled on-treatment and post-treatment assessment time points;
9. Urinalysis by dipstick and microscopic analysis: Change from Baseline to each of the scheduled on-treatment and post-treatment assessment time points;
10. 12-lead electrocardiogram (ECG): Change from Baseline to each of the scheduled on-treatment and post-treatment assessment time points;
11. Hand x-ray: bone age at Month 24;
12. Spine x-ray: Spine fractures at Month 24.

Data for the following additional safety outcomes will be listed only:

1. Physical examination findings at Pretreatment, Month 12, and Month 24.

2.2.2 Clinical Efficacy Endpoints

2.2.2.1 Primary Clinical Efficacy Endpoint

1. Time to Stand Test (TTSTAND) velocity (rise/second): Comparison with a historical natural history (untreated) control group for change from Baseline to Month 24.

2.2.2.2 Secondary Efficacy Endpoints

1. Time to Stand Test (TTSTAND) velocity (rise/second): Comparison with a historical natural history (untreated) control group for change from Baseline to Month 12;
2. Time to Stand Test (TTSTAND): Change from Baseline to each of the scheduled on-treatment and post-treatment assessment time points;
3. Time to Climb (4 Steps) Test (TTCLIMB): Change from Baseline to each of the scheduled on-treatment and post-treatment assessment time points;
4. North Star Ambulatory Assessment (NSAA): Change in timed assessments and total score from Baseline to each of the scheduled on-treatment and post-treatment assessment time points;
5. Total distance traveled, in meters, in completing the Six-minute Walk Test (6MWT): Change from Baseline to each of the scheduled on-treatment and post-treatment assessment time points;
6. Time to Run/Walk Test (TTRW): Change from Baseline to each of the scheduled on-treatment and post-treatment assessment time points; and
7. Quantitative Muscle Testing (QMT): Change from Baseline to each of the scheduled on-treatment and post-treatment assessment time points.

2.2.3 Pharmacodynamic Endpoints

Concentrations of serum PD biomarkers of adrenal suppression, insulin resistance, and bone turnover.

2.2.4 Exploratory Endpoints

1. Pediatric Outcomes Data Collection Instrument (PODCI): Change from baseline to each of the scheduled on-treatment and post-treatment assessment time points;
2. Levels of additional exploratory PD biomarkers; and
3. DNA testing for established genetic modifiers of DMD.

2.2.5 Endpoints for Subject Reported Outcomes

Safety endpoints based on subject reports of AEs are listed in Section **2.2.1**. Additionally, subjects' parents/legal guardians will be asked to complete the Pediatric Outcomes Data Collection Instrument (see Section **7.3.8)**. No other subject-reported outcomes are planned.

3 STUDY DESIGN

3.1 Overall Study Design

This Phase II long term extension study is an open-label, multiple-dose study to evaluate the long-term safety, tolerability, efficacy and PD of vamorolone administered once daily by liquid oral suspension over a Treatment Period of 24 months to young boys with DMD.

Only subjects who have completed the VBP15-003 Phase IIa extension study Week 24 Final Visit will be eligible for participation in the VBP15-LTE open-label extension study. Participation in this extension study will be discussed with the subject's parent or guardian prior to the subject's final visit of VBP15-003. A total of up to approximately 50 subjects will be enrolled into this long term extension study.

The parents or legal guardians of subjects who choose to enroll in this long term extension study will give written informed consent for the long-term extension study at the VBP15-LTE Baseline Day -1 Visit. The Baseline Day -1 Visit, including signing of

the VBP15-LTE-specific ICF, may occur at the conclusion of the VBP15-003 final visit; alternatively, the Baseline Day -1 Visit and VBP15-003-specific ICF signing may occur up to 8 weeks after the final VBP15-003 visit for subjects who dose taper, at the convenience of the subject's parent or legal guardian and discretion of the Investigator. Subjects are considered to be enrolled in the VBP15-LTE long term extension study after the parent or guardian has signed the VBP15-LTE-specific ICF. Each subject will retain the study identification number assigned to him at the start of the Phase IIa study VBP15-002 and retained in the Phase IIa extension study VBP15-003.

For subjects who enroll in the VBP15-LTE long term extension study within 28 days after completion of all VBP15-003 Week 24 final assessments, many of the safety, efficacy, and PD assessments performed at the VBP15-003 Week 24 Final Visit may be used to determine extension study eligibility and/or to provide baseline study data for the extension study and do not need to be repeated at the VBP15-LTE Baseline Day -1 Visit (see **Table 8**). For these subjects, additional long term extension study procedures will still be performed at the Baseline Day -1 Visit, within 24 hours prior to administration of the first dose of study drug in the long term extension study. Subjects who enroll in the VBP15-LTE long term extension study > 28 days after the date of the VBP15-003 Week 24 Final Visit must have all Baseline Day -1 assessments performed, according to the schedule in **Table 8**.

Subjects in the VBP-LTE will begin dosing at the same vamorolone dose level they received at the time they completed the Week 24 Final Visit in the VBP15-003 Phase IIa extension core study. Subjects, once they have been on their initial dose in the VBP15-LTE study for at least one month, may have their dose escalated at the discretion of the Study Chair and Medical Monitor once that dose has been determined to be safe in the VBP15-002 Phase IIa Study, and no safety issues have emerged at that dose in the VBP15-003 Phase IIa extension study. Subjects may continue to have their dose of study medication escalated incrementally at no shorter than one-month intervals to the maximum dose tested and assessed as safe in the VBP15-002 and VBP15-003 studies, 6.0 mg/kg/day. Subjects will continue to receive vamorolone at one of the assigned dose

levels for the duration of the 24-month Treatment Period, unless ongoing safety data indicate the dose level should be de-escalated.

If dose limiting toxicities are identified in the VBP15-002 Phase IIa study or VBP15-003 Phase IIa extension study which preclude confirmation of safety of all four planned dose levels, the dose levels in this extension study will be modified, as needed.

In the event any clinical observation suggests an intolerability for an individual subject to the study medication, in the opinion of the Investigator, the subject's dose level may be decreased to the next lower dose level and maintained at that lower dose level throughout the duration of the Treatment Period, with the caveat stated below for subjects de-escalating from 6.0 mg/kg/day to 2.0 mg/kg/day. In the event the next lower dose level is also not tolerated and is considered a safety risk to the subject, in the opinion of the Investigator, Study Chair, and Medical Monitor, the subject should be withdrawn from the study. Subjects whose dose is decreased from 6.0 mg/kg/day to 2.0 mg/kg/day may have their dose subsequently increased to 4.0 mg/kg/day if they have been taking the 2.0 mg/kg/day dose for at least one month and, in the opinion of the Investigator, balancing efficacy with safety concerns, they could benefit from an intermediate higher dose. Details of dose interruption, de-escalation, and discontinuation are presented in Section **5.6**.

Subjects will be assessed for safety and tolerability, clinical efficacy, and PD at scheduled visits throughout the 24-month Treatment Period (see **Table 8** for a schedule of study assessments). Treatment Period study visits will occur at Month 1, Month 3, and every 3 months thereafter through Month 24 (**Table 8**). Adverse events, including SAEs, and concomitant medications will be recorded throughout the study.

Once daily Treatment Period study drug dosing will occur from Day 1 until the Month 24 Visit (Section **5.3**).

Subject diaries will be dispensed at Baseline Day -1 and at each subsequent 3-month scheduled study visit to record AEs and changes to concomitant medications taken during the study.

All subjects will return to the clinical site for Month 24 assessments. Subjects who switch to standard of care glucocorticoids for DMD or who discontinue treatment will participate in a Dose-tapering Period of 2-5 weeks in duration, following the end of the Treatment Period and prior to discharge from the study (see Section **6.3.4**). Subjects will be discharged from the study following completion of all final Month 24 or Dose-tapering Period assessments, as appropriate.

In the event that any clinical or laboratory parameters remain abnormal at the time of discharge from the study, the subject will be followed medically as clinically indicated. Any subject who discontinues the study prior to the Month 24 Visit should return to the study unit for final Month 24 safety and efficacy assessments at the time of early discontinuation.

3.2 Study Summary

This Phase II long term extension study is an open-label, multicenter study to evaluate the long-term safety, tolerability, clinical efficacy, and PD of vamorolone at dose levels up to 6.0 mg/kg administered daily by liquid oral suspension over a Treatment Period of 24 months to young boys with DMD who participated in the VBP15-002 Phase IIa and VBP15-003 Phase IIa extension core studies.

The study is comprised of a Pretreatment Baseline Period of up to 24 hours in duration, which begins at the signing of the VBP-LTE study-specific informed consent, a 24-month Treatment Period, and a 2- to 5-week Dose-tapering Period for subjects who elect to transition off vamorolone treatment at the end of the study.

4 SELECTION AND WITHDRAWAL OF STUDY SUBJECTS

4.1 Subject Screening, Enrollment, and Identification Log

All subjects who complete the Week 24 Final Visit of the VBP15-003 Phase IIa core extension study will be eligible for enrollment in this VBP15-LTE long term extension study, provided they meet all VBP15-LTE study entry criteria. Limited data will be collected for subjects who are not interested in participating in the study, including date of birth and reason for exclusion from the study. Subjects are considered to be enrolled

in the VBP15-LTE long term extension study after the parent or guardian has signed the VBP15-LTE-specific ICF at the Baseline Day -1 Visit. The Baseline Day -1 Visit, including signing of the ICF, may occur at the conclusion of the final visit of the VBP15-003; alternatively, the Baseline Day -1 Visit and VBP15-LTE-specific ICF signing may occur up to 8 weeks after the VBP15-003 final visit following dose-tapering, at the convenience of the subject's parent or legal guardian and discretion of the Investigator.

Subject enrollment and identification logs will be maintained for all subjects enrolled in the study. These logs will be reviewed during routine monitoring calls and/or visits.

4.2 Inclusion Criteria

To qualify for enrollment in this extension study, the subject must satisfy the following inclusion criteria:

1. Subject's parent or legal guardian has provided written informed consent and HIPAA authorization (if applicable) prior to any VBP15-LTE long-term extension study-specific procedures;
2. Subject has previously completed study VBP15-003 up to and including the Week 24 Final assessments prior to enrolling in the VBP15-LTE study at the conclusion of the VBP15-003 Week 24 Visit [Note: if entering the dose-tapering period, subject is enrolling within 8 weeks after the VBP15-003 final visit following dose-tapering]; and
3. Subject and parent/guardian are willing and able to comply with scheduled visits, study drug administration plan, and study procedures.

4.3 Exclusion Criteria

A subject will be excluded from enrollment in this extension study if he meets any of the following exclusion criteria:

1. Subject had a serious or severe adverse event in study VBP15-003, that, in the opinion of the Investigator, was probably or definitely related to vamorolone use and precludes safe use of vamorolone for the subject in this long-term extension study;

2. Subject has current or history of major renal or hepatic impairment, diabetes mellitus or immunosuppression;
3. Subject has current or history of chronic systemic fungal or viral infections;
4. Subject has used mineralocorticoid receptor agents, such as spironolactone, eplerenone, canrenone (canrenoate potassium), prorenone (prorenoate potassium), mexrenone (mexrenoate potassium) within 4 weeks prior to the first dose of study medication;
5. Subject has evidence of symptomatic cardiomyopathy. [Note: Asymptomatic cardiac abnormality on investigation would not be exclusionary];
6. Subject is currently being treated or has received previous treatment with oral glucocorticoids or other immunosuppressive agents. [Note**s**: Past transient use of oral glucocorticoids or other oral immunosuppressive agents for no longer than 3 months cumulative, with last use at least 3 months prior to first dose of study medication, will be considered for eligibility on a case-by-case basis. Inhaled and/or topical glucocorticoids prescribed for an indication other than DMD are permitted but must be administered at stable dose for at least 3 months prior to study drug administration];
7. Subject has used idebenone within 4 weeks prior to the first dose of study medication;
8. Subject has an allergy or hypersensitivity to the study medication or to any of its constituents;
9. Subject has severe behavioral or cognitive problems that preclude participation in the study, in the opinion of the Investigator;
10. Subject has previous or ongoing medical condition, medical history, physical findings or laboratory abnormalities that could affect safety, make it unlikely that treatment and follow-up will be correctly completed or impair the assessment of study results, in the opinion of the Investigator; or

11. Subject is currently taking any investigational drug, or has taken any investigational drug other than vamorolone within 3 months prior to the start of study treatment.

Note: Subjects may be re-evaluated if ineligible due to a transient condition which would prevent the subject from participating.

4.4 Withdrawal of Subjects from Study

A subject may withdraw from the study, or may be withdrawn by his parent or guardian at any time without the need to justify the decision.

The Investigator has the right to terminate participation of a subject in the study for any of the following reasons:

- The subject's parent/legal guardian is uncooperative/noncompliant and does not adhere to study responsibilities, including failure to appear at study visits;
- Difficulty in obtaining blood samples from the subject for safety monitoring;
- The subject experiences an unmanageable or non-tolerable AE/SAE which is considered to be possibly, probably, or definitely related to study drug, in the opinion of the Investigator;
- The Sponsor terminates the study;
- Any other reason relating to subject safety or integrity of the study data.

In the event a subject is withdrawn from the study, the Sponsor or designee (e.g., Coordinating Center) will be informed within one business day. If there is a medical reason for withdrawal, the subject will remain under the supervision of the Investigator until resolution of the event.

All subjects who withdraw from the study prior to the Month 24 Visit should return to the study site for Month 24 assessments at the time of early withdrawal, assuming the subject has not withdrawn consent. In the event a subject withdraws informed consent, no further study procedures should be performed and no additional data

should be collected. Any data collected up to the point of withdrawal of informed consent may be used by the Sponsor.

4.5 Replacement of Withdrawn Subjects

Only subjects who have completed the VBP15-003 Phase IIa extension study are eligible to enroll in this long-term extension study. Subjects prematurely discontinued from the VBP15-LTE long term extension study will not be replaced.

4.6 Termination of Study

This study may be prematurely terminated if, in the opinion of the Sponsor, there is sufficient reasonable cause. An example of a circumstance that may warrant termination is determination of unexpected, significant, or unacceptable risks to participants.

If the study is prematurely terminated or suspended, the Sponsor will promptly inform the site Investigators and the regulatory authority(ies) of the termination or suspension and the reason(s) for the termination or suspension. The Institutional Review Board(s) (IRB[s])/Independent Ethics Committee(s) (IEC[s]) will also be informed promptly by the Investigator/institution or the Sponsor and provided the reason(s) for the termination or suspension.

Subject enrollment at a given site may be terminated by the Sponsor. Possible reasons for termination of the study at a given site include, but are not limited to:

1. Unsatisfactory enrollment with respect to quantity or quality
2. Inaccurate or incomplete data collection
3. Falsification of records
4. Failure to adhere to the protocol.

5 TREATMENT OF STUDY SUBJECTS

5.1 Study Medication Administered

Vamorolone will be administered to all subjects as an oral liquid suspension.

Planned vamorolone dose levels: 0.25 mg/kg, 0.75 mg/kg/day, 2.0 mg/kg, and 6.0 mg/kg.

Vamorolone will be administered to all subjects once daily for 24 months, from Study Day 1 until the Month 24 Visit. At the end of the 24-month Treatment Period, a subset of subjects may receive additional vamorolone treatment in a dose-tapering manner during a 2- to 5-week Dose-tapering Period, prior to discharge from the study (see Section **6.3.4**).

5.2 Identity of Investigational Product

ReveraGen BioPharma, Inc. will supply the following investigational study medication:

Name:	Vamorolone
Active Substance:	VBP15
Strength:	4% by weight
Dosage Form:	Oral suspension
Manufacturer:	Velesco Pharma

5.3 Dosage Schedule and Administration of Study Medication

Subjects who have completed the Phase IIa extension VBP15-003 study and who meet all other eligibility criteria (see Sections **4.2** and **4.3**) will be enrolled into this long term extension study on Baseline Day -1, following signing of the VBP15-LTE extension-study-specific ICF. Subjects will retain the enrollment numbers assigned to them for the Phase IIa extension VBP15-003 core study.

Subjects will receive vamorolone 0.25 mg/kg, 0.75 mg/kg/day, 2.0 mg/kg, or 6.0 mg/kg, administered orally once daily with a glass of full fat milk (approximately 8 grams of fat) or food with similar fat content of the subject's choice from Study Day 1 to Month 24. A subset of subjects may also receive vamorolone for an additional 1-4 weeks according to a dose-tapering protocol following the end of the Treatment Period (see Section **6.3.4**).

The site pharmacist or designated site study staff will dispense study medication in 100 mL bottles to each subject enrolled in the study (see Section **5.8.1** and **Appendix 14.1**). All subjects will receive vamorolone 4% oral suspension.

Study medication sufficient for 3 months of daily dosing (plus overage) will be dispensed by trained study staff on Study Day -1 and at each scheduled subsequent 3-month visit thereafter. Each subject's dose (in mg and mL) will be calculated based on the weight of

the subject (in kg) recorded at the dispensing visit (see **Appendix 14.1** for a dose calculation worksheet).

Enrolled subjects will receive all doses of vamorolone under the supervision of parent or legal guardian or trained study staff. Dosing is to occur at home throughout the 24-month Treatment Period, except at the Month 9 and Month 21 Visits when dosing will occur at the study site to accommodate morning fasted blood collections. Study drug will be administered by mouth using a volumetric syringe supplied by the site. Following administration of the dose of study drug, the syringe will be filled once with water and the water will be administered by mouth using the volumetric syringe. The subject will then drink approximately 50 mL of water to ensure the full dose has been ingested. Subjects should receive each dose of study medication at approximately the same time of day.

The daily dose of study medication should be taken with a full glass of whole milk (approximately 8 grams of fat), or equivalent high fat food portion.

The dispensed study medication bottle(s) will be returned to the study site at each subsequent scheduled visit.

5.4 Rationale for Dose Selection

Dose levels were chosen for this study to ensure the safety of subjects enrolled in the study, to allow demonstration of efficacy and PD effects, and to ensure adequate safety in future clinical studies and in the patient population after FDA regulatory approval. All dosing will be done in the morning with a glass of whole milk (approximately 8 grams of fat) or similar fat equivalent meal.

Each subject will enroll in this long-term extension study to receive the same dose that he received at the time the subject completed the Week 24 Visit of the VBP15-003 core study. The planned dose levels in this study are 0.25 mg/kg, 0.75 mg/kg, 2.0 mg/kg, and 6.0 mg/kg. If dose-limiting toxicities are observed in the Phase IIa or Phase IIa extension core studies at doses being used in this LTE study, dose level(s) will be de-escalated, as appropriate.

Based on the safety data presented in Section **1.3.3**, the doses initially selected for this study are appropriate for evaluation in a long-term extension study.

5.5 Treatment Compliance

Subject compliance with the dosing schedule will be assessed by site maintenance of accurate study drug dispensing and return records. The Investigator is responsible for ensuring that dosing is administered in compliance with the protocol. The Investigator or designee will instruct the subject's parent or guardian with regard to proper dosing of study medication, and will reinforce the importance of taking all study medication per protocol instructions. The volume of unused study medication remaining in each bottle returned will be documented in the source documents and on the appropriate electronic case report form (eCRF).

5.6 Study Drug Dose Interruption, De-escalation, or Discontinuation

Administration of study drug to individual subjects should be interrupted, and the case discussed with the Study Chair and Medical Monitor within 24 hours, in the event any clinical observation suggests an intolerability to the study medication and a safety risk to an individual subject, in the opinion of the Investigator. In such an event, or in situations where the intolerability is not considered an immediate safety risk to the subject, the subject's dose level may be decreased to the next lower dose level and maintained at that lower dose level throughout the duration of the study, with the caveat stated below for subjects de-escalating from 6.0 mg/kg/day to 2.0 mg/kg/day. In the event the next lower dose level is also not tolerated and is considered a safety risk to the subject, in the opinion of the Investigator, Study Chair and Medical Monitor, the subject should be withdrawn from the study.

Subjects whose dose is decreased from 6.0 mg/kg/day to 2.0 kg/kg/day may have their dose subsequently increased to 4.0 mg/kg/day if they have been taking the 2.0 mg/kg/day dose for at least one month and, in the opinion of the Investigator, balancing efficacy with safety concerns, they could benefit from an intermediate higher dose.

In the event that dose-limiting toxicities are observed in the VBP15-002 Phase IIa or VBP15-003 Phase IIa extension study that require de-escalation of dose in the long-term

extension study VBP15-LTE, subjects will be de-escalated as dictated by safety results in the VBP15-003 study.

5.7 Prior and Concomitant Medications and Therapies

5.7.1 Prior Therapy

Any changes in non-study medication/therapy, including administration of new medication(s), change of dose, or discontinuation of medication, that occur after completion of the VBP15-003 core study Week 24 final assessments, or the final assessments following dose-tapering for subjects who taper the dose, and prior to administration of the first dose of study medication in the extension study will be captured as prior medications (Medication History) in the VBP15-LTE extension study eCRF.

5.7.2 Concomitant Therapy

Any medications that are started after administration of the first dose of study medication in the VBP-15 LTE extension study will be recorded as concomitant medications on the appropriate eCRF. Subject diaries will be provided to subjects to record any concomitant medication changes during the study (see Section **8.4**).

All medications (prescription and over-the-counter [OTC]) taken during the VBP15-LTE study must be recorded in the source documents and in the eCRF, including the name of the medication, dosage and regimen, reason for therapy, and treatment start and stop dates. Furthermore, each change in concomitant medication (e.g., new treatment, discontinuation of treatment, or change in dosage/regimen) during the study must be documented in the same manner. Details of any non-pharmacological therapies (e.g., devices, procedures), including name, reason for therapy, and dates of therapy, will also be recorded. Site personnel will review the information with the subject and/or his parent or guardian, if applicable, for completeness and accuracy at each study visit.

5.7.3 Prohibited Therapies

Subjects must discontinue use of the following medications prior to participation in the study, as indicated, and refrain from using these medications throughout the duration of the study:

- Mineralocorticoid receptor agents, such as spironolactone, eplerenone, canrenone (canrenoate potassium), prorenone (prorenoate potassium), mexrenone (mexrenoate potassium): use must be discontinued at least 4 weeks prior to the first dose of study medication;
- Oral glucocorticoids or other oral immunosuppressive agents: Subjects who have received more than 3 months cumulative treatment with oral immunosuppressive agents or last treatment within 3 months prior to first dose of study medication are ineligible for study entry. [Note: Inhaled and/or topical glucocorticoids prescribed for an indication other than DMD are permitted but must be administered at stable dose for at least 3 months prior to study drug administration];
- Idebenone: use must be discontinued at least 4 weeks prior to first dose of study medication;
- Live attenuated vaccines (use must be avoided for the duration of participation in the study);
- Medications indicated for the treatment of DMD, including Exondys 51 and Translarna (use must be avoided for the duration of participation in the study);
- Any investigational medications other than vamorolone (use must be discontinued at least 3 months prior to the first dose of study medication).

The Investigator should contact the Study Chair concerning individual medications or therapies not listed that may be of concern.

5.7.4 Permitted Therapies

Every effort should be made NOT to take any prescription or OTC medications during the study. Concomitant medications should be maintained on the same dose and regimen throughout the study whenever possible. However, all other medications other than those specifically prohibited above may be taken during the study, if clinically indicated, provided they are recorded in the source documents and in the eCRF.

5.8 Study Medication Management

5.8.1 Packaging and Labeling of Study Medication

The site pharmacist or designated study staff will receive clinical trial material (CTM) when all regulatory requirements have been completed by the site. Additional CTM will be available upon request. ReveraGen BioPharma, Inc. or designee will provide CTM in bulk quantities sufficient to satisfy the protocol requirement. Clinical trial material will be shipped in bulk to the study site's registered pharmacist or designated study staff in suitably labeled study cartons. Cartons will contain study medication packaged in sterile 100 mL glass bottles; each bottle will contain 4 grams of vamorolone as a 4% suspension in sterile water. Bulk drug supplies will be labeled with the name of Sponsor, protocol number, lot number, expiration or retest date, and other appropriate study information. Bottle labels will include the following statement: "Caution: New Drug – Limited by Federal Law to Investigational Use" or comparable statement, as required by ex-US regulatory authorities.

100 mL bottles of 4% vamorolone oral suspension will be dispensed to the subject's parent or legal guardian at the Baseline Day -1 Visit and at each scheduled dispensing visit (see **Appendix 14.1** for instructions on calculating the number of vamorolone bottles to be dispensed at Baseline Day -1 and each subsequent scheduled 3-month visit); dispensed bottle(s) of study medication will be returned at each subsequent scheduled 3-month visit, prior to dispensing sufficient bottle(s) to provide daily dosing until the next subsequent scheduled 3-month visit. Each vamorolone study medication bottle may be used for a single subject only. The volume per dose to be administered to each subject depends on the subject weight calculated at the dispensing visit; see **Appendix 14.1** for

complete instructions on calculating dose volume. Clinical supplies dispensed by the study site staff and ready for administration to subjects will be labeled with the dispense date, protocol number, vamorolone dose level, and volume to be administered per dose.

5.8.2 Storage of Study Medication

All CTM for use in the trial must be stored in a locked container/cabinet free from environmental extremes, under the responsibility of the institutional pharmacist or Principal Investigator. Bulk study medication should be stored at refrigerated temperature (2°C – 8°C; 36°F – 46°F). Excursions to ambient temperature are allowed. Access to study medication stored at the study site must be limited to authorized clinic personnel.

5.8.3 Study Medication Shipping and Handling

Clinical trial material will be shipped to the study sites only after receipt of required documents in accordance with applicable regulatory requirements and Sponsor procedures.

Clinical trial material will only be dispensed once a subject has (1) a signed ICF and HIPAA authorization, if applicable, on file, and has been enrolled in the VBP15-LTE long-term extension study; (2) met all eligibility criteria for entry into the VBP15-LTE long-term extension study, (3) completed all Pretreatment requirements.

It is essential to this study that all CTM be accounted for during the study period. All unused study medication will be retained at the study site for reconciliation and collection by the Sponsor's study monitors (or designees) during routine monitoring visits. Study site personnel should not dispose of any CTM. Final disposition of all unused CTM will be coordinated by the Sponsor's study monitors (or designees) at the end of the study (see Section **5.8.4**).

Clinical trial material must be dispensed and administered according to the procedures described in this protocol. Only subjects enrolled in the study may receive study medication, in accordance with all applicable regulatory requirements. Only authorized study personnel may supply CTM. Authorized study personnel refers to the Investigator

(or designee), in accordance with all applicable regulatory requirements and the Site Signature Log/Delegation of Authority. Only authorized study personnel or the subject's parent or legal guardian may administer CTM.

5.8.4 Study Medication Accountability

The Investigator is responsible for the control of drugs under investigation. Adequate records of the receipt (e.g., Drug Receipt Record) and disposition (e.g., Drug Dispensing Log) of the study drug must be maintained. The Drug Dispensing Log must be kept current and should contain the following information:

- The identification of the subject to whom the study drug was dispensed
- The date(s) and quantity of the study drug dispensed to the subject
- The date(s) and quantity of the study drug returned by the subject.

All records and drug supplies must be available for inspection by the Study Monitor at every monitoring visit. Unused medication will be returned to ReveraGen or its designee at the end of the study. The completed Drug Dispensing Log and drug return record(s) will be returned to ReveraGen or its designee. The Investigator's copy of the drug return record(s) must accurately document the return of all study drug supplies to ReveraGen or its designee.

5.9 Procedures for Assigning Subject Study Numbers

This is an open-label study. Subjects enrolled in this long-term extension study will retain the enrollment numbers assigned to them at the start of the VBP15-002 core study. All data for all subjects whose parent or guardian signs the ICF for the VBP15-LTE extension study will be identified using the unique subject identification number. Subjects are considered to be enrolled in this extension study when the parent or guardian signs the VBP15-LTE extension study-specific ICF at the Baseline Day -1 Visit. The Site Investigator will keep a record relating the names of the subjects to their enrollment numbers (Subject Identification Log) to permit efficient verification of data subject files, when required. This record will also include the dates of subject enrollment and completion/termination.

6 STUDY SCHEDULE

6.1 Time and Events Schedule

The study procedures to be conducted for each subject are divided into the following study periods:

Pretreatment Baseline Period: The 24-hour period immediately prior to administration of the first dose of study medication (Study Day -1), which includes signing of the VBP15-LTE extension study-specific ICF and HIPAA authorization, if applicable, and enrollment into the extension study. The Baseline Day -1 Visit will generally coincide with the final Week 24 Visit in the VBP15-003 core study. Alternatively, subjects who participate in the Dose-tapering Period may return to the study site up to 8 weeks after completion of the VBP15-003 final assessments following the Dose-tapering Period for signing of the ICF and HIPAA authorization, if applicable, and completion of Baseline assessments at Baseline Day -1.

Treatment Period: The 24-month interval starting with administration of the first dose of study medication on Study Day 1and continuing through the time of the Month 24 Visit and administration of the final dose of Treatment Period study medication.

Dose-tapering Period: The 2- to 5-week interval following the end of the 24-month Treatment Period during which subjects who elect to either switch to standard of care glucocorticoids for DMD, or discontinue vamorolone and not begin standard of care glucocorticoid treatment, at the end of the study will have their vamorolone dose tapered to 0 mg/kg/day. The length of time required for this tapering will depend upon the dose received during the Treatment Period (see Section **6.3.4**). Subjects whose dose is tapered during the Dose-tapering Period will be discharged from the study following completion of all final Dose-tapering Period assessments.

The procedures to be completed during each study period are presented in the Schedule of Study Assessments in **Table 8** and in the sections that follow. Detailed descriptions of the assessments and the definitions of study endpoints are provided in Section **7** and Section **2.2**. Any deviation from study procedures should be noted in the source

documents and in the eCRF, and significant deviations should be reported immediately to the Sponsor.

Overall, approximately 24-25 months are allocated for each subject to complete the study, including a 1-day Pretreatment Baseline Period and a 24-month Treatment Period for all subjects. For subjects who elect to either switch to standard of care glucocorticoids for DMD, or discontinue vamorolone and not begin glucocorticoid treatment at the end of the study, an additional 2-5 weeks are allocated for vamorolone dose tapering and final study assessments.

Table 8 Schedule of Study Activities

	Pretreatment Period		Treatment Period									Dose-tapering Period
	Baseline											
	Day		Month									
**Study Day or Month/Visit**	**-1[a]**	**1[b]**	**1 (±7d)**	**3 (±7d)**	**6 (±7d)**	**9 (±7d)**	**12 (±7d)**	**15 (±7d)**	**18 (±7d)**	**21 (±7d)**	**24[c] (±7d)**	**24-25[d] (±7d)**
Inclusion/Exclusion Criteria	X											
Informed Consent	X[e]											
Enrollment	X											
Interim Medical History	X[f]											
Medication History	X[g]											
Physical Examination	X[h]						X				X	
Height	X						X				X	
Weight	X			X	X	X	X	X	X	X	X	
Vital Signs[i]	X		X	X	X	X	X	X	X	X	X	X
Blood for Clinical Labs[j]	X[h]				X		X		X		X	X
Urinalysis[k]	X[h]				X		X		X		X	
Blood for Serum PD Biomarker Panel[l]	X[h]				X		X		X		X	X
Fasting morning blood for insulin, glucose, cortisol[m]						X				X		
Blood for DNA Testing											X	
12-lead ECG[n]	X[h]						X				X	
Dispense Study Medication	X			X	X	X[o]	X	X	X	X[o]	X	
Return Study Medication/ Compliance Monitoring				X	X	X	X	X	X	X	X	X
Study Medication Dosing		X	→	→	→	→	→	→	→	→	X	
Vamorolone dose tapering[p,q]												X
Time to Stand Test (TTSTAND)	X[h]						X				X	
Time to Climb Test (TTCLIMB)	X[h]						X				X	
Time to Run/Walk Test (TTRW)	X[h]						X				X	
NSAA[r]	X[h]						X				X	
Quantitative Muscle Testing (QMT)	X[h]						X				X	
Six-minute Walk Test (6MWT)	X[h]						X				X	
Pediatric Outcomes Data Collection Instrument (PODCI)	X[h]						X				X	
Spine x-ray											X	
Hand x-ray											X	
Dispense Subject Diaries[s]	X			X	X	X	X	X	X	X	X	
Return Subject Diaries			X	X	X	X	X	X	X	X	X	X
AE/SAE Recording[t]	X	→	→	→	→	→	→	→	→	→	X	X
Concomitant Medications		X	→	→	→	→	→	→	→	→	X	X
Discharge from Study											X[u]	X[v]

d = day(s); w = week.

a. Baseline Day -1, within 24 hours prior to administration of the first dose of study drug. The Baseline Visit for Study VBP15-LTE can coincide with the Week 24 Final Visit for Study VBP15-003, or the final visit following dose-tapering for subjects who dose taper, or may occur up to 8 weeks after the date of the final visit following dose tapering.
b. Treatment Day 1 begins at the time of administration of the first dose of VBP15-LTE study medication at home. No scheduled study visit will occur on Day 1.
c. Subjects who prematurely discontinue from the study prior to Month 24 should complete the Month 24 assessments at the time of early discontinuation.
d. All subjects EXCEPT those who elect to continue vamorolone therapy in a further extension study must continue in the Dose-tapering Period and have their vamorolone dose tapered at weekly intervals over a 2-5 week period prior to discharge from this study. Subjects participating in the Dose-tapering Period will have one study site visit during this period, at the end of dose tapering (Months 24-25) (see **6.3.4**).
e. Informed Consent for this extension study may be obtained at the Study VBP15-003 Week 24 Final Visit or within 8 weeks after the final dose-tapering visit for subjects who dose-taper, after completion of all final VBP15-003 study assessments and prior to any VBP15-LTE extension study-specific procedures. For all subjects, informed consent for VBP15-LTE participation will be given on Study Day -1, within 24 hours of administration of first dose of study medication in VBP15-LTE.
f. Interim Medical History will be collected on Baseline Day -1 for all subjects, and will include any AEs that occurred during the VBP15-003 core study and are ongoing at the time of entry into VBP15-LTE (see **7.2.1**).
g. Any changes in medication/therapy including administration of new medication(s), change of dose, or discontinuation of medication after completion of the VBP15-003 core study and prior to administration of the first dose of study medication in VBP15-LTE will be captured as Prior Medications.
h. If the Baseline Day -1 Visit occurs ≤ 28 days after the date of the Week 24 Final Visit in the VBP15-003 core study, these assessments may be used for the VBP15-LTE study Baseline assessments. Clinical laboratory and urinalysis test results from the VBP15-003 Week 24 Final Visit may be used and should be reviewed by the Site Investigator to determine eligibility for Baseline Day -1 enrollment into the VBP15-LTE long-term extension study.
i. Supine blood pressure, oral temperature, respiratory rate, and heart rate.
j. Blood for hematology, chemistry, and lipids.
k. Urinalysis by dipstick and microscopic analysis.
l. Blood collected for pharmacodynamic safety biomarkers and exploratory safety and efficacy biomarkers.
m. Blood will be collected for insulin, glucose, and cortisol determination in the morning after subjects have fasted for ≥ 6 hours, prior to the daily dose of study medication.
n. ECG recorded after subject has rested quietly in a supine position for at least 5 minutes.
o. The dose of study medication on the days of the Month 9 and 21 Visits will be administered after 1) a morning fasting blood draw for insulin, glucose, and cortisol; and 2) breakfast provided by the study site. All other doses will be taken at home.
p. Only for subjects who will participate in the Dose-tapering Period.
q. Subjects who elect to switch to standard of care glucocorticoids, or discontinue vamorolone and not begin glucocorticoid treatment for DMD at the end of the study will have their vamorolone dose tapered at weekly intervals to a dose of 0 mg/kg/day prior to final study assessments (see **6.3.4**).
r. North Star Ambulatory Assessment; includes the Time to Stand Test (TTSTAND).
s. Subject diaries used to record any changes to concomitant medications taken and any AEs experienced during the study.
t. All AEs and SAEs must be collected in the source documents and eCRF from the date of the subject's written informed consent until the Month 24 Visit or the subject's participation in the study is completed. Ongoing AEs will be followed to resolution, stabilization, or until such time the Investigator agrees follow-up is not necessary.
u. Subjects who elect to continue vamorolone therapy in a subsequent extension study may be discharged from the study following completion of all final Month 24 procedures.
v. Subjects who participate in the Dose-tapering Period may be discharged from the study following completion of all final Dose-tapering Visit assessments (see **6.3.4**).

6.2 Informed Consent/HIPAA Authorization

The parents or guardians of subjects who choose to enroll in this long-term extension study will give written informed consent for this study at the Baseline Day -1 Visit. If the Baseline Day -1 Visit coincides with the VBP15-003 Week 24 Final Visit, the parents or guardians of subjects may give written informed consent for this study following completion of all VBP15-003 assessments at the final visit. For subjects who participate in the VBP15-003 Dose -tapering Period, informed consent for the VBP15-LTE study may be given within 8 weeks after completion of the final VBP15-003 Dose-tapering Visit. The Investigator (or designated staff) will obtain the written informed consent and HIPAA authorization, if applicable, from the subject's parent or guardian prior to any extension study-specific procedures. Each subject's parent or guardian will receive an explanation of the nature and purposes of the study from the Investigator or designee. The Investigator or designee will ensure the study is appropriate for the subject. Reasons for exclusion will be documented for subjects found ineligible during the Pretreatment Period. The subject's parent or guardian will be asked if s/he understands that the study is for research purposes only and that it may not provide any therapeutic benefit to the subject. Each subject's parent or guardian will be asked if s/he understands that the subject is free to withdraw from the study at any time without prejudice. If relevant, the Investigator or designee will review the elements of the HIPAA and Protected Health Information (PHI) with each subject's parent or guardian and each subject's parent or guardian will be asked if s/he understands HIPAA authorization and PHI. Each subject's parent or guardian will be required to sign a study ICF (and HIPAA authorization, if applicable) before any procedures are performed for the long-term extension study. The written assent of children will be obtained per individual site guidelines.

The Investigator or designee will obtain written informed consent from each subject's parent or guardian prior to subject's participation in the long-term extension study on Informed Consent Forms approved by the appropriate IRB/IEC at each site. Consent must be obtained in accordance with the principles outlined in the current version of the Declaration of Helsinki. Informed Consent Forms must be dated and signed by the Investigator or designee and the subject's legal representative and the original signed

consent form must be kept by the Investigator in the study subject's file. "Legal representative" means an individual whom a judicial or other body authorized under applicable law to consent on behalf of a prospective study subject to the subject's participation in the procedure(s) involved in the research. The Study Monitor will ensure that the ICF has been signed by the subject's legal representative. The study subject's legal representative will receive a copy of the signed consent form.

6.3 Visit Schedule and Procedures

During the study, there will be a total of up to 11 study site visits: Pretreatment Baseline Visit (Day -1); Treatment Period Months 1, 3, 6, 9, 12, 15, 18, 21, and 24; and a final Dose-tapering Period Visit for subjects whose vamorolone dose is tapered at the end of the Treatment Period. Each subject will receive the investigational product for a period of 24 months. Subjects will initiate dosing in the VBP15-LTE study at the same dose level at which they were dosing at the time of the Week 24 Final Visit in the VBP15-003 Phase IIa core extension study. The dose level may be increased at the discretion of the Study Chair and Medical Monitor once the subject has received the initial dose in the VBP15-LTE study for at least one month, the next dosing level has been determined to be safe in the VBP15-002 Phase IIa core study, and no safety issues at that dose have emerged in the VBP15-003 core study. Subjects who participate in the Dose-tapering Period will receive additional vamorolone at a decreasing dose over 1 to 4 weeks during a 2- to 5-week Dose-tapering Period, and will return to the study site for final study assessments at the end of the Dose-tapering Period. See Section 7 for a detailed description of the safety, clinical efficacy, and PD assessments to be performed in this study.

6.3.1 Baseline Period (Day -1)

For all subjects, the Baseline Day -1 Visit will occur after completion of all VBP15-003 final procedures and within 24 hours prior to administration of the first dose of study medication in the VBP15-LTE long term extension study.

Parents or guardians of subjects who have completed all VBP15-003 study assessments will sign the ICF and HIPAA authorization, if applicable, for the VBP15-LTE long term

extension study at the Baseline Day -1 Visit. If either the VBP15-003 Week 24 Final Visit or the final visit following dose-tapering for subjects who dose-taper, will coincide with Baseline Visit for VBP15-LTE, the VBP15-LTE-specific ICF must be signed after completion of all VBP15-003 assessments at the relevant final visit and prior to the performance of any VBP15-LTE extension study-specific procedures (Section **6.2**).

Subjects whose VBP15-LTE Baseline Day -1 Visit occurs ≤ 28 days after the VBP15-003 Week 24 Final Visit, may use the physical examination, clinical laboratory tests, PD biomarker blood draw, 12-lead ECG, and clinical efficacy assessments performed at the VBP15-003 Week 24 Final Visit as their Baseline Day -1 Visit assessments.

Subjects will retain the subject identification number assigned for the Phase IIa VBP-002 and Phase IIa extension VBP15-003 core studies.

Any new medications taken from the time of completion of the VBP15-003 study to administration of the first dose of study medication in the VBP15-LTE study, or any changes to medications taken during the VBP15-003 core study, will be recorded as Prior Medications on the appropriate eCRF. See Section **7** for detailed descriptions and instructions for completion of each safety, efficacy, and PD assessment.

The following procedures will be performed at the Baseline Day -1 Visit (see **Table 8**):

- Review of Inclusion and Exclusion Criteria (see **4.2** and **4.3**)
- Written informed consent and HIPAA authorization, if applicable, for the VBP15-LTE extension study (see **6.2**)
- Enrollment (see **5.9**)
- Recording of Interim Medical History (see **7.2.1**)
- Recording of Medication History (see **5.7.1**)
- Recording of AEs and SAEs beginning at the time written informed consent is obtained (see **7.2.6**)
- Complete physical examination (not to be repeated if Baseline Day -1 is ≤ 28 days after the VBP15-003 Week 24 Final Visit assessment date) (see **7.2.2**).
- Measurement of height and weight (see **7.2.2**)

- Recording of vital signs (supine blood pressure, heart rate, oral temperature, respiratory rate) (see 7.2.3)
- Clinical laboratory evaluation including hematology, clinical chemistry, lipids, and urinalysis tests (not to be repeated if Baseline Day -1 is ≤ 28 days after the VBP15-003 Week 24 Final Visit assessment date) (see 7.2.4)
- Blood samples for PD biomarkers. Blood will also be collected and stored for exploratory PD biomarkers. (Not to be repeated if Baseline Day -1 is ≤ 28 days after the VBP15-003 Week 24 Final Visit assessment date.) (see 7.3.7)
- 12-lead ECG (not to be repeated if Baseline Day -1 is ≤ 28 days after the VBP15-003 Week 24 Final Visit assessment date) (see 7.2.5)
- Time to Run/Walk Test (TTRW) (not to be repeated if Baseline Day -1 is ≤ 28 days after the VBP15-003 Week 24 Final Visit assessment date) (see 7.3.3)
- Time to Stand Test (TTSTAND) (not to be repeated if Baseline Day -1 is ≤ 28 days after the VBP15-003 Week 24 Final Visit assessment date) (see 7.3)
- Time to Climb Test (TTCLIMB) (not to be repeated if Baseline Day -1 is ≤ 28 days after the VBP15-003 Week 24 Final Visit assessment date) (see 7.3.2)
- North Star Ambulatory Assessment (NSAA) (not to be repeated if Baseline Day -1 is ≤ 28 days after the VBP15-003 Week 24 Final Visit assessment date) (see 7.3.4)
- Six-minute Walk Test (6MWT) (not to be repeated if Baseline Day -1 is ≤ 28 days after the VBP15-003 Week 24 Final Visit assessment date) (see 7.3.5)
- Quantitative Muscle Testing (QMT) (not to be repeated if Baseline Day -1 is ≤ 28 days after the VBP15-003 Week 24 Final Visit assessment date) (see 7.3.6)
- Pediatric Outcomes Data Collection Instrument (PODCI) completed by parent/legal guardian (see 7.3.8).
- Dispensing of study medication and subject diaries (see 5.3 and 8.4)

6.3.2 Treatment Period Day 1

Treatment Period Day 1 begins with administration of the first dose of study medication. There is no scheduled clinic visit on Day 1.

Subjects will take the first dose of study medication at home on the morning of the day after the Day -1 Visit, on Study Day 1 with a full glass of whole milk (approximately 8 grams of fat) or a serving of an equivalent fat amount of a high fat food, under the supervision of a parent or guardian (**5.3**).

6.3.3 Treatment Period Months 1-24

Subjects will return to the study site for safety, efficacy, and PD assessments beginning at Month 1 and continuing through Month 24, according to the schedule of visits in **Table 8**.

Subjects will continue to receive daily oral administration of vamorolone throughout the 24-month Treatment Period, taken following ingestion of a full glass of whole milk (approximately 8 grams of fat) or a serving of an equivalent fat amount of high fat food.

Dosing is to occur at home throughout the 24-month Treatment Period, except at the Month 9 and Month 21 Visits when dosing will occur at the study site to accommodate morning fasted blood collections. Subjects must have fasted ≥ 6 hours prior to arrival at the study site for Month 9 and Month 21 procedures and assessments. The pre-dose blood draws for PD (insulin, glucose, and cortisol only) determination must be collected after subjects have fasted for ≥ 6 hours. Breakfast (a glass of whole milk [approximately 8 grams of fat] or an equivalent high fat food portion and 1 cup of cereal) will be served at the study site after the blood draw for insulin, glucose, and cortisol, and within approximately 30 minutes prior to administration of the dose of study medication. All other Month 9 and Month 21 assessments should be performed after administration of the dose of study medication.

Clinical efficacy assessments (TTSTAND, TTRW, TTCLIMB, NSAA, 6MWT, and QMT) and the Pediatric Outcomes Data Collection Instrument (PODCI) will be conducted at 12 and 24 months. Weight will be recorded every visit from 3-24 months and height will be measured at 12 and 24 months. Vital signs will be recorded at each study visit. A physical examination will be performed at Months 12 and 24. A 12-lead ECG will be recorded at Months 12 and 24. Blood and urine samples for clinical laboratory tests, and blood for PD biomarkers will be collected at 6, 12, 18, and 24 months (see **Table 8**). Blood will be collected at Month 24 for DNA testing for

established genetic modifiers of DMD. Spine x-ray and hand x-ray will be performed at Month 24. Adverse events, including SAEs, and concomitant medications will be assessed at each study visit and recorded throughout the study.

Study medication will be dispensed and returned at 3-month intervals for all subjects. Subjects will receive subject diaries at Baseline Day -1 visit and each scheduled 3-month visit and return the diaries at each subsequent visit; diaries returned at the Month 1 Visit will be reviewed by study staff for accuracy and returned to the subjects for continued use through the time of the Month 3 Visit. Diaries will be reviewed with the subject's parent or guardian by the study staff to assess AEs and changes to concomitant medications/therapies.

Subjects who will not participate in the Dose-tapering Period (see Section **6.3.4**) will be discharged from the study following completion of all Month 24 assessments. Subjects who do participate in the Dose-tapering Period will be dispensed vamorolone (and standard of care glucocorticoid at the discretion of the Investigator for subjects who elect to transition to standard of care glucocorticoid treatment) at the Month 24 Visit, as well as instructions for tapering the dose of vamorolone during the Dose-tapering Period.

6.3.4 Dose Tapering Period (Months 24-25)

Subjects who elect to transition to standard of care glucocorticoid treatment for DMD, or discontinue vamorolone and not begin standard of care glucocorticoid treatment for DMD at the end of the study will have their vamorolone dose tapered prior to discharge from the study. Subjects who discontinue study medication prior to completion of Month 24 assessments will also participate in the Dose-tapering Period if possible and if, in the opinion of the Investigator, it is safe to do so. Dose tapering will be performed in a stepwise manner, according to the subject's vamorolone dose at completion of the 24-month Treatment Period, as outlined in **Table 9**. Subjects who elect to transition to a standard of care glucocorticoid will have their vamorolone dose tapered as in **Table 9**, and will begin treatment with a standard of care glucocorticoid on the day dose tapering begins. These subjects will continue to receive standard of care glucocorticoid treatment,

with drug and dose at the discretion of the Investigator, throughout the duration of the Dose-tapering Period and after discharge from the study.

The subject's weight recorded at the Month 24 Visit will be used to calculate dose and dose volume for all dose de-escalations during the Dose-tapering Period (see **Appendix 14.1**).

Table 9 Vamorolone Dose Tapering

Treatment Period Vamorolone Dose Level at Month 24 Final Visit	Dose Level for First Week Following the Month 24 Final Visit	Dose Level for Second Week Following the Month 24 Final Visit	Dose Level for Third Week Following the Month 24 Final Visit	Dose Level for Fourth Week Following the Month 24 Final Visit	Dose Level for Fifth Week Following the Month 24 Final Visit
0.25 mg/kg/day	0.125 mg/kg/day	0 mg/kg/day			
0.75 mg/kg/day	0.375 mg/kg/day	0.125 mg/kg/day	0 mg/kg/day		
2.0 mg/kg/day	1 mg/kg/day	0.5 mg/kg/day	0.25 mg/kg/day	0 mg/kg/day	
4.0 mg/kg/day	2 mg/kg/day	1 mg/kg/day	0.5 mg/kg/day	0.25 mg/kg/day	0 mg/kg/day
6.0 mg/kg/day	3 mg/kg/day	1.5 mg/kg/day	0.75 mg/kg/day	0.25 mg/kg/day	0 mg/kg/day

All subjects participating in the Dose-tapering Period will return to the study site for final study assessments approximately one week after the final dose de-escalation (i.e., approximately one week after the subject has received his last dose of vamorolone). The duration of the Dose-tapering Period will therefore vary depending upon the vamorolone dose received by the subject at completion of the Month 24 Visit of the Treatment Period, from a total duration of two weeks (for subjects receiving 0.25 mg/kg/day vamorolone; final study visit two weeks following the Month 24 Final Visit) to five weeks (for subjects receiving vamorolone 4.0 mg/kg/day or 6.0 mg/kg/day; final study visit five weeks following the Month 24 Final Visit).

At the final study visit following the Dose-tapering Period, subjects participating in the Dose-tapering Period will have vital signs recorded, and blood collected for clinical laboratory tests and PD biomarker panel. Study medication will be returned for compliance monitoring. Adverse events, including SAEs, and concomitant medications will be assessed. Subject diaries will be returned and reviewed with site staff.

Subjects participating in the Dose-tapering Period will be discharged from the study following completion of all final Dose-tapering Period assessments.

6.4 Subject Discontinuation

In the event that a subject withdraws early from the study prior to the Month 24 Final Visit, the reason for discontinuation must be fully documented in the source documents and the eCRF. For subjects who withdraw early, final Month 24 assessments should be performed at the time of early discontinuation (see Section **6.3.3**). Site personnel will document all assessments, including any AEs, in the source documents and eCRF. Subjects who discontinue study medication should follow vamorolone dose tapering procedures described in Section **6.3.4** and detailed in the Manual of Operations.

6.5 Study Completion

A completed subject is defined as a subject who has completed the 24-month Treatment Period and Dose-tapering Period, if applicable, and has not prematurely withdrawn from the study for any reason.

7 STUDY ASSESSMENTS AND MEASUREMENTS

7.1 Demographic Assessments

Demographic information (birth date, race, and ethnicity) collected during the VBP15-002 study will be used for this study; collection of demographic data will not be repeated during this study.

7.1.1 Genetic Modifiers of DMD

Approximately 8.5 mL of blood will be collected at the Month 24 Visit for DNA testing to determine if established genetic modifiers of DMD (gene polymorphisms associated with disease severity or response to glucocorticoid treatment) are similarly associated with vamorolone-treated DMD patients (baseline disease severity or response to vamorolone treatment).

DNA testing will be performed by a certified central laboratory.

The procedures for the collection, handling, and shipping of blood samples for DNA testing will be specified in the Laboratory Manual(s) provided to the clinical center. Results will be compared to historical control data for glucocorticoid responders and non-responders.

7.2 Safety and Tolerability Assessments

7.2.1 Interim Medical History

At the Baseline Day -1 Visit, interim medical history will be recorded for each subject, including medical history migrated from the VBP15-003 core study and any medical history that occurred in the period between completion of VBP15-003 and enrollment into the VBP15-LTE long term extension study. Migrated medical history will include any condition that was recorded as medical history for the VBP15-003 Phase IIa extension core study. Medical history occurring between completion of VBP15-003 and VBP15-LTE enrollment will include surgical history and concurrent diseases, including any changes from the core study. All AEs that are recorded during the VBP15-003 core study and are ongoing at the time of enrollment into the VBP15-LTE study will be recorded as medical history in the VBP15-LTE study.

Interim medical history will be recorded in the source documents and in the eCRF.

7.2.2 Physical Examination, Weight, and Height

A complete physical examination will be performed at the Baseline Day -1, Month 12, and Month 24 Visits, and will include examination of the following: head, eyes, ears, nose, and throat, neck *(including an examination of the thyroid),* heart, lungs, abdomen *(including an examination of the liver and spleen),* lymph nodes, extremities, nervous system, and skin. If the Baseline Day -1 Visit occurs ≤ 28 days after the date of the VBP15-003 Phase IIa extension core study Week 24 Final Visit, the Baseline Day -1 physical examination does not need to be repeated (results from the VBP15-003 Week 24 Final Visit may be used as the Baseline assessment).

Additional unscheduled symptom-directed physical examinations may be conducted at any time at the Investigator's discretion.

Height (in cm) will be recorded at Baseline Day -1, and Months 12 and 24. Weight (in kg) will be recorded at Baseline Day -1, Months 3, 6, 9, 12, 15, 18, 21, and 24. Weight recorded at each scheduled visit will be used to calculate the vamorolone dose volume for each subsequent period's dosing (see **Appendix 14.1**).

Results will be recorded in the source documents and on the appropriate eCRF.

7.2.3 Vital Signs

Vital signs (supine blood pressure, heart rate, respiration rate, and oral temperature) will be recorded at Baseline Day -1, and each scheduled visit thereafter through Month 24, and at the final study visit following dose-tapering for subjects who dose-taper. Vital signs should be recorded after the subject has been resting for at least 5 minutes.

Results will be recorded in the source documents and on the appropriate eCRF.

If vital signs assessment is performed at the same study visit as blood sampling and ECG recording, the following sequence should be followed: 1) ECG recording; 2) vital signs assessment; and 3) blood sampling.

7.2.4 Clinical Laboratory Tests

Each subject will have blood drawn and urine collected for the hematology, chemistry, lipids, and urinalysis clinical laboratory tests listed in **Table 10** and **Table 11**, below, at the Baseline Day -1, and Month 6, 12, 18, and 24 Visits. Subjects whose dose is tapered will also have clinical laboratory tests performed at the final visit following the Dose-tapering Period. If the Baseline Day -1 Visit occurs ≤ 28 days after the date of the VBP15-003 Phase IIa extension core study Week 24 Final Visit, clinical laboratory test results from that visit may be used as baseline measurements for the VBP15-LTE study.

For all subjects, the clinical laboratory test results obtained at the VBP15-003 Week 24 Final Visit will be used to assess extension study eligibility.

All blood and urine samples will be sent to the designated central laboratory for testing.

For the hematology, chemistry, and lipids laboratory tests, blood will be collected by direct venipuncture of peripheral veins. Approximately 4.5 mL of blood will be obtained for clinical laboratory tests at each scheduled visit. A total of approximately 18-27 mL of

blood will be collected over the course of this study for clinical laboratory evaluation (see Section **7.4** for details of blood volumes to be collected).

If blood sampling is performed at the same study visit as vital signs assessment and ECG recording, the following sequence should be followed: 1) ECG recording; 2) vital signs assessment; and 3) blood sampling.

Any abnormal hematology, chemistry, lipid, or urinalysis test result deemed clinically significant by the Investigator or medically qualified sub-investigator may be repeated, including test results obtained on the final study day.

Any treatment-emergent abnormal laboratory test result that is clinically significant, i.e., meeting one or more of the following conditions, should be recorded as a single diagnosis on the AE section of the eCRF:

- Accompanied by clinical symptoms
- Requiring a change in concomitant therapy (e.g., addition of, interruption of, discontinuation of, or any other change in a concomitant medication, therapy, or treatment)
- Is otherwise considered clinically significant by the Investigator

Any clinically significant test abnormality as defined above should be recorded as an AE (unless it was considered spurious), and repeat analysis performed until resolution or until the Investigator or medically qualified sub-investigator determines that resolution of the abnormality is not expected.

Table 10 Hematology, Chemistry, and Lipids Clinical Laboratory Tests

**Hematology**		
	Red Blood Cells (RBC)	Numerical platelet count (estimate not acceptable)
	Hemoglobin	White Blood Cells (WBC) with differential (percent)
	Hematocrit	
**Chemistry**		
	Sodium	Total Bilirubin[a]
	Potassium	Uric Acid
	Chloride	Glucose
	Calcium	Alkaline phosphatase (ALP)
	Inorganic Phosphorus	Gamma Glutamyl Transferase (GGT)
	Blood Urea Nitrogen (BUN)	Aspartate aminotransferase (AST)
	Creatinine	Alanine aminotransferase (ALT)
	Total Protein	Creatine kinase (CK)
	Albumin	Lipase
	Bicarbonate	Amylase
	Lactate Dehydrogenase (LDH)	
**Lipids**		
	Triglycerides	Low Density Lipoprotein (LDL)
	Total cholesterol	High density Lipoprotein (HDL)
	a. If outside normal range, direct bilirubin will be measured and reported.	

Urine will be collected for routine analysis, by dipstick and microscopic analysis, for the tests described in Table 11. Urine will be analyzed by dipstick and microscopic analysis by the central laboratory at all assessment time points.

Table 11 Urinalysis Clinical Laboratory Tests

**Urinalysis (including microscopic examination)**	
**Dipstick[a]**	**Microscopic Analysis**
Protein	WBC/hpf
Glucose	RBC/hpf
Ketones	Casts
pH	Bacteria
Leukocyte esterase	
Blood	

[a]A midstream clean-catch urine specimen will be collected for dipstick analysis.

Clinical laboratory tests will be performed by a central laboratory; results will be reported to the study site and transferred electronically into the clinical study database.

The procedures for the collection, handling, and shipping of laboratory samples will be specified in the Laboratory Manual(s) provided to the clinical center.

7.2.4.1 Follow-up of Abnormal Laboratory Test Results

In the event of a medically significant, unexplained, or abnormal clinical laboratory test value, the test(s) may be repeated, evaluated by the Investigator for sustainability and reproducibility to determine if the abnormality represents an AE, and followed-up until the results have returned to the normal range, stabilized, and/or an adequate explanation for the abnormality is found. If a clear explanation is established, it should be recorded in the source documents and eCRF. The clinical laboratory will clearly mark all laboratory test values that are outside the normal range and the Investigator will indicate which of these deviations are clinically significant. These clinically significant deviating laboratory results will then be further described as AEs, and the relationship to the treatment, in the Investigator's opinion, will be indicated (see Section **7.2.6**).

7.2.5 12-Lead ECG

12-lead ECGs will be collected at the Baseline Day -1, Month 12 and Month 24 Visits. If the Baseline Day -1 Visit occurs ≤ 28 days after the date of the VBP15-003 Phase IIa extension core study Week 24 Final Visit, the Baseline Day -1 12-lead ECG does not need to be repeated (results from the VBP15-003 Week 24 Final Visit may be used). All ECG recordings must be performed using a standard high-quality, high-fidelity machine equipped with computer-based interval measurements. Digital ECG recording is recommended. Automated ECG intervals (QRS duration, PR [PQ] interval, RR interval [interbeat interval], QT interval, QTc, and heart rate) will be captured or calculated.

12-lead ECGs will be obtained over a 3- to 5-minute period after the subject has been resting quietly in a supine position for at least 5 minutes.

If blood sampling, vital signs assessment, and ECG recordings are scheduled at the same study visits, the following sequence should be followed: 1) ECG recording; 2) vital signs assessment; and 3) blood sampling.

ECG results will be read locally. Results must be interpreted and recorded on the appropriate eCRF.

7.2.6 Adverse Events and Serious Adverse Events

The condition of the subjects will be monitored throughout the duration of the study by the clinical site study team and by recording of AEs in subject diaries. An AE is any untoward medical occurrence in a subject which does not necessarily have to have a causal relationship with the intervention. An AE can therefore be any unfavorable and unintended sign (including an abnormal laboratory finding, for example), symptom, or disease temporally associated with the use of an investigational product, whether or not considered related to the product. Pre-existing conditions that worsen during a study are to be reported as AEs.

Adverse events will be recorded from the date of informed consent and through the time of the subject's last study visit (study completion or early discontinuation). Serious adverse events will be recorded from the date of informed consent, throughout the clinical trial, and for up to 30 days after the final administration of study drug. Subjects who enroll in the VBP15-LTE long term extension study will have any AEs which initiated during VBP15-003 participation and which are ongoing at the time of enrollment into VBP15-LTE recorded as Medical History in the VBP15-LTE eCRF. In addition, subjects (and their parent or legal guardian) will be questioned by study staff at each study visit for any new signs or symptoms or changes in existing signs or symptoms.

All AEs and SAEs that are spontaneously reported, identified during questioning, or are apparent from a participant's physical appearance will be recorded in the source documents and in the subject's eCRF. The date of onset will be recorded. Any laboratory abnormality that is outside the normal range and is considered an AE (see Section **7.2.4**) should be recorded as an AE on the appropriate eCRF. The details recorded shall include the nature, date of onset, final outcome and its date, intensity

assessment (Common Terminology Criteria for Adverse Events [CTCAE] grade), and a determination of the relationship of the event to administration of the study drug (i.e., causality). All AEs will be graded by CTCAE, Version 4.03. Details of any medications given to the subject to abate the AE should be recorded in the appropriate eCRF.

Intensity

All clinical AEs encountered during the clinical study will be recorded in the eCRF. Intensity of AEs will be graded using the most current version of the Common Terminology Criteria for Adverse Events (CTCAE), version 4.03, 5-point scale, and reported in detail as indicated on the eCRF. A description of the intensity scales can be found below:

Mild (Grade 1): Asymptomatic or mild symptoms: clinical or diagnostic observations only; intervention not indicated.
Moderate (Grade 2): Minimal, local, or noninvasive intervention indicated; limiting age-appropriate instrumental activities of daily living (ADL).
Severe (Grade 3): Severe or medically significant but not immediately life-threatening: hospitalization or prolongation of hospitalization indicated; disabling; limiting self-care ADL; incapacitating with inability to work or perform normal daily activity.
Life-Threatening (Grade 4): Urgent intervention indicated.
Death (Grade 5): Death related to AE.

Relationship

Relationship to study drug will be graded on a 5-point scale (definite, probable, possible, remote, or unrelated). A description of the relationship scale can be found below:

Definite: This category applies to an AE that meets at least criteria 1, 2, and 4 of the "Probable" category.

Probable: This category applies to those AEs that are considered, with a high degree of certainty, to be related to the study drug. An AE may be considered probable, if (must include first 3):

1. It follows a reasonable temporal sequence from administration of the study drug.
2. It cannot be reasonably explained by the known characteristics of the subject's clinical state, environmental or toxic factors, or other modes of therapy administered to the subject.
3. It disappears or decreases after dosing is complete. (There are important exceptions when an AE does not disappear upon discontinuation of study drug, yet drug relatedness clearly exists, e.g., [1] bone marrow depression and [2] tardive dyskinesia.)
4. It follows a known pattern of response to the suspected study drug.

Possible: This category applies to those AEs for which the connection with study drug administration appears unlikely but cannot be ruled out with certainty. An AE may be considered possibly related to study drug if or when (must include first 2):

1. It follows a reasonable temporal sequence from administration of the study drug.
2. It may have been produced by the subject's clinical state, environmental or toxic factors, or other modes of therapy administered to the subject.
3. It follows a known pattern of response to the suspected study drug.

Remote: In general, this category is applicable to an AE that meets the following criteria (must include the first 2):

1. It does not follow a reasonable temporal sequence from administration of the study drug.
2. It may readily have been produced by the subject's clinical state, environmental or toxic factors, or other modes of therapy administered to the subject.
3. It does not follow a known pattern of response to the suspected study drug.

Unrelated: This category is applicable to those AEs which are judged to be clearly and incontrovertibly due only to extraneous causes (disease, environment, etc.) and

do not meet the criteria for study drug relationship listed under remote, possible, or probable.

Clinical Laboratory Test Abnormalities

Clinical laboratory test results will be recorded in the designated eCRF. The intensity of abnormal clinical laboratory test results that are AEs will also be graded using the most current version of the CTCAE, version 4.03, 5-point scale and reported in detail as indicated in the eCRF. A description of the intensity scale can be found above.

Any treatment-emergent abnormal clinical laboratory test result that is clinically significant, i.e., meeting one or more of the following conditions, should be recorded as a single diagnosis on the AE section of the eCRF:

- Accompanied by clinical symptoms
- Requiring a change in concomitant therapy (e.g., addition of, interruption of, discontinuation of, or any other change in a concomitant medication, therapy, or treatment)
- Is otherwise considered clinically significant by the Investigator

This applies to any protocol and non-protocol-specified safety laboratory result from tests performed after the first dose of study drug, which falls outside the laboratory reference range and meets the clinical significance criteria per Investigator standard operating procedures (SOPs).

This does not apply to any abnormal laboratory result that falls outside the laboratory reference range, but does not meet the clinical significance criteria (which will be analyzed and reported as laboratory abnormalities); those that are considered AEs of the type explicitly exempted by the protocol; or those that are the result of an AE which has already been reported.

Please Note: any clinical laboratory abnormality fulfilling the criteria for an SAE should be reported as such, in addition to being recorded as an AE in the eCRF.

Follow-Up of Adverse Events

Adverse events will be followed until they have returned to baseline status, stabilized, or the Investigator, Study Chair, Medical Monitor and Sponsor agree that follow-up is no longer needed. If a clear explanation of cause is established, it should be recorded in the source documents and eCRF. In the event of unexplained abnormal laboratory test values, the tests may be repeated as soon as possible and followed up until they have returned to the normal range or baseline value and/or an adequate explanation of the abnormality is found. In case of ongoing AEs at the time of database closure, the data obtained at the time of database closure will be used in the statistical analysis. The follow-up of AEs will be documented in the source documents and will be described in the final report only if considered relevant by the Investigator, the Study Chair, the Medical Monitor and/or the Sponsor.

In addition, the Medical Monitor may request additional blood tests, diagnostic imaging studies, or specialist physician consultations in order to further evaluate any AE or test abnormality considered to be clinically significant by the Study Sponsor.

Dosing Error

For the purposes of this study, a dosing error is defined as a dose exceeding or less than the scheduled dose of 0.25 mg/kg, 0.75 mg/kg, 2.0 mg/kg, or 6.0 mg/kg specified for each dose level group. Such occurrences should be reported and recorded in the dosing page of the eCRF and as follows:

- Use of study medication in doses in excess of that specified in the protocol should not be recorded as an AE unless there are associated signs or symptoms.
- A dosing error with associated non-serious AEs should be recorded as AEs on the relevant AE forms in the eCRF.
- A dosing error with an associated SAE should be recorded as an SAE.
- Details of all dosing errors, including actual dose administered, should be documented in the source documents.

7.2.6.1 Serious Adverse Events

For treatment-eligible subjects, SAEs will be collected and reported during the study from the time informed consent is obtained through 30 days after the final dose of study medication, according to the protocol and applicable regulations.

All SAEs, including those that continue beyond the normal AE collection period (i.e., are ongoing at the subject's last study visit), will be followed until resolution or until stabilized without sequelae. Serious adverse events that begin after the subject's participation in the study is complete but that the Investigator considers to be related to study drug will be reported to the Sponsor within 24 hours or discovery by the Investigator.

During the SAE collection period, the Investigator or clinical site personnel should notify the PRA safety management team of all SAEs, regardless of relationship to the investigational drug, within 24 hours of clinical staff becoming aware of the event; notification to the PRA safety management team will trigger alerts to the Coordinating Center, Study Chair, the Sponsor, and the Medical Monitor. The Investigator will provide the initial notification by completing the SAE eCRF in the electronic data capture (EDC), which must include the Investigator's assessment of the relationship of the event to investigational drug. In the unlikely event that the electronic study database is inaccessible and the Investigator is unable to complete the SAE eCRF within 24 hours, the SAE Notification Form (pdf) should be completed and emailed or printed/faxed to the PRA safety management team.

In addition, notification is sent by the Investigator to the IRB and the subject's Primary Care Physician.

Follow-up information, or new information regarding an ongoing SAE, must be provided promptly to the PRA safety management team within 24 hours of knowledge of the new or follow-up information, which will forward the information to the Coordinating Center, Study Chair, the Sponsor, and the Medical Monitor.

The Data and Safety Monitoring Board (DSMB) will review SAEs at regular intervals during the study.

All SAE reports should be completed within the EDC.

An AE or suspected adverse reaction is considered serious if, in the view of either the Investigator or Sponsor, it results in any of the following outcomes:

- Is fatal (results in the outcome of death)
- Is life-threatening
- Requires in-patient hospitalization or prolongation of existing hospitalization
- Results in persistent or significant incapacity or substantial disruption of the ability to conduct normal life functions
- Is a congenital anomaly or birth defect
- Is an important medical event that may jeopardize the subject and may require medical or surgical intervention to prevent one of the outcomes listed above.

The term “sudden death” should only be used when the cause is of a cardiac origin as per standard definition.

The terms death and sudden death are clearly distinct and must not be used interchangeably.

Any AE or clinically significant abnormal laboratory test value, as determined by the Investigator, that is serious and which occurs during the course of the study (as defined above) must be reported to the PRA safety management team, who will notify the Coordinating Center, Study Chair, the Sponsor, and the Medical Monitor within 24 hours of the Investigator becoming aware of the event. Additional information that becomes available for an SAE after the initial report is submitted will be reported to the PRA safety management team, who will notify the Coordinating Center**,** Study Chair, the Sponsor, and the Medical Monitor within 24 hours of the Investigator becoming aware of the new information.

Related SAEs MUST be collected and reported regardless of the time elapsed from the last administration of study drug, even if the study has been closed. Unrelated SAEs must be collected and reported during the study from the time of informed consent through 30 days after the final dose of study medication.

If, at any time during the study, a subject experiences an SAE, appropriate care should be instituted.

In the event of an SAE, the Investigator will complete the SAE eCRF within 24 hours of first awareness of the event. In the unlikely event that the electronic study database is inaccessible and the Investigator is unable to complete the SAE eCRF within 24 hours, the SAE Notification Form (pdf) should be completed and emailed or printed/faxed to the PRA safety management team within 24 hours, using the contact information below:

Email CHOSafety@prahs.com
Drug Safety Fax: 1-888-772-6919 or 1-434-951-3482

SAE Questions: Drug Safety Hotline: 1-800-772-2215 or 1-434-951-3489

Serious Adverse Events will be recorded from the time the subject's written informed consent is obtained. Serious adverse events that occur within 30 days of study drug dosing must continue to be recorded and reported to the Study Sponsor or its designee. Should there be an SAE that occurs that suggests an increased risk to the participants, the following steps will be considered, depending on the number and severity of the SAE(s): modification of the protocol, investigation of the relationship of the SAE(s) to study drug, suspension of the study, and/or discontinuation of the study.

7.2.7 Bone Age (Hand X-ray)

Bone age will be assessed by hand x-ray at the Month 24 Visit. Hand radiography will be performed as described in the Manual of Operations. Hand radiographs will be analyzed centrally by a certified radiologist through the imaging coordinating center in Ottawa, Canada.

7.2.8 Spine X-ray

Data on bone health will also be collected by lateral spine x-ray (T4-L5) at the Month 24 Visit. Lateral spine x-rays will be analyzed centrally by two certified pediatric radiologists at Children's Hospital of Ottawa in Ottawa, Canada, who are blinded to the results of one another; a third radiologist will resolve any discrepancies arising from the first two readings. Quantification of any vertebral fractures detected will be performed.

Fractures will be recorded as AEs.

7.3 Clinical Efficacy and Pharmacodynamic Assessments

7.3.1 Time to Stand Test (TTSTAND)

The Time to Stand Test (TTSTAND) will be administered to subjects at the Baseline Day -1, Month 12 and Month 24 Visits. If the Baseline Day -1 Visit occurs ≤ 28 days after the date of the VBP15-003 Phase IIa extension core study Week 24 Final Visit, the Baseline Day -1 TTSTAND does not need to be repeated (results from the VBP15-003 Week 24 Final Visit may be used).

The TTSTAND measures the speed (rise/second; velocity) with which the child can stand to an erect position from supine (floor), and is administered as part of the NSAA (see Section **7.3.4**). Complete instructions for administering and scoring the TTSTAND are given in the study manual to be supplied to the sites prior to study start.

Results will be recorded in the source documents and in the eCRF.

7.3.2 Time to Climb Test (TTCLIMB)

The Time to Climb Test (TTCLIMB) will be administered to subjects at the Baseline Day -1, Month 12 and Month 24 Visits. If the Baseline Day -1 Visit occurs ≤ 28 days after the date of the VBP15-003 Phase IIa extension core study Week 24 Final Visit, the Baseline Day -1 TTCLIMB does not need to be repeated (results from the VBP15-003 Week 24 Final Visit may be used).

The TTCLIMB measures the time (in seconds) required for the subject to climb 4 standard stairs, beginning and ending in a standing position with arms at the sides.[50] Complete instructions for administering the TTCLIMB are given in the study manual to be supplied to the sites prior to study start.

Results will be recorded in the source documents and in the eCRF.

7.3.3 Time to Run/Walk Test (TTRW)

The TTRW will be administered to subjects at the Baseline Day -1, Month 12 and Month 24 Visits. If the Baseline Day -1 Visit occurs ≤ 28 days after the date of the

VBP15-003 Phase IIa extension core study Week 24 Final Visit, the Baseline Day -1 TTRW does not need to be repeated (results from the VBP15-003 Week 24 Final Visit may be used).

The Time to Run/Walk Test (TTRW) measures the time (in seconds) that it takes a subject to run or walk 10 meters and is administered as part of the NSAA (see Section 7.3.4). Complete instructions for administering and scoring the TTRW are given in the study manual to be supplied to sites prior to study start.

Results will be recorded in the source documents and in the eCRF.

7.3.4 North Star Ambulatory Assessment (NSAA)

The North Star Ambulatory Assessment (NSAA) is a clinical assessment scale specifically designed to measure functional ability in ambulant boys with DMD.[51] The NSAA consists of 17 scored items and 2 timed tests, including the TTRW and the TTSTAND (see Section 7.3). The NSAA will be conducted at the Baseline Day -1, Month 12 and Month 24 Visits. If the Baseline Day -1 Visit occurs ≤ 28 days after the date of the VBP15-003 core study Week 24 Final Visit, the Baseline Day -1 NSAA does not need to be repeated (results from the VBP15-003 Week 24 Final Visit may be used).

Subjects should be barefoot and wear comfortable clothing. Complete instructions for administering and scoring the NSAA are given in the study manual to be supplied to the sites prior to study start.

The NSAA should be administered BEFORE the 6MWT at study visits where both tests are administered.

Results will be recorded in the source documents and in the eCRF.

7.3.5 Six-minute Walk Test (6MWT)

Functional exercise capacity and mobility will be assessed in all subjects by means of the Six-minute Walk Test (6MWT) at the Baseline Day -1, Month 12 and Month 24 Visits. If the Baseline Day -1 Visit occurs ≤ 28 days after the date of the VBP15-003 Phase IIa extension core study Week 24 Final Visit t, the Baseline Day -1 6MWT does not need to be repeated (results from the VBP15-003 Week 24 Final Visit may be used).

This evaluation is a modified version of the 6MWT, adapted for use in DMD patients.[52]

The total distance traveled, in meters, should be recorded along with the validity of the test as assessed by the test administrator in the source documents and in the eCRF. If a subject cannot complete 6 minutes of walking, the total meters and the time until discontinuation of the test should be recorded. Complete instructions for administering the 6MWT are given in the study manual to be supplied to the sites prior to study start.

The 6MWT should be administered AFTER the NSAA at study visits where both tests are administered.

Results will be recorded in the source documents and in the eCRF.

7.3.6 Quantitative Muscle Testing (QMT)

Quantitative Muscle Testing (QMT) will be administered to subjects at the Baseline Day -1, Month 12, and Month 24 Visits using the CINRG Quantitative Measurement System (CQMS).[53] If the Baseline Day -1 Visit occurs ≤ 28 days after the date of the VBP15-003 Phase IIa extension core study Week 24 Final Visit, the Baseline Day -1 QMT does not need to be repeated (results from the VBP15-003 Week 24 Final Visit may be used).

The QMT measures unilateral strength of four muscle groups: elbow flexion/extension, and knee flexion/extension.[53] Complete instructions for administering the QMT are given in the study manual to be supplied to the sites prior to study start.

Results will be collected using the CQMS3 software and transferred into the eCRF.

7.3.7 Pharmacodynamic Biomarker Panel

Blood samples will be collected to explore the effect of vamorolone on biomarkers of muscle cellular pathology, biomarkers associated with chronic glucocorticoid treatment, and biomarkers associated with insulin resistance, adrenal suppression, and bone turnover, as listed in **Table 12**. Blood samples will be collected at the Baseline Day -1 and Month 6, 12, 18, and 24 Visits, and at the final Dose-tapering Period Visit for subjects whose vamorolone dose is tapered at the end of the study. If the Baseline Day -1 Visit occurs ≤ 28 days after the date of the VBP15-003 Phase IIa extension core study

Week 24 Final Visit, the Baseline Day -1 pharmacodynamic biomarker blood draw does not need to be repeated (results from the VBP15-003 Week 24 Final Visit may be used). Blood samples for PD biomarkers will be drawn after completion of functional tests at the Baseline, and Months 12 and 24 Visits.

Blood will also be collected at each of these time points and stored for exploratory efficacy and safety pharmacodynamic biomarkers.

Morning, fasted blood samples will be collected at the Month 9 and 21 Visits for measurement of fasting glucose, insulin, and cortisol after the subject has fasted for ≥ 6 hours and prior to administration of the daily dose of study medication.

A total of 78-110 mL of blood will be collected for PD biomarkers over the course of the 24-month study (see Section 7.4).

Table 12 Pharmacodynamic Biomarkers

**Adrenal Suppression**
Morning Cortisol
ACTH
17- hydroxyprogesterone
Testosterone
Corticosterone
11- deoxycortisol
**Insulin Resistance**
Fasting Glucose
Fasting Insulin
HbA1c
**Bone Turnover**
Osteocalcin
CTX1
P1NP
**Exploratory Biomarkers**
Other PD biomarkers

7.3.8 Pediatric Outcomes Data Collection Instrument (PODCI)

Quality of life will be assessed by completion of the Pediatric Outcomes Data Collection Instrument (PODCI). The subject parent/legal guardians will be asked to complete this instrument at the Baseline Day -1, Month 12, and Month 24 Visits.

Results will be recorded in the eCRF.

7.4 Total Blood Volume Required

The number and volume of blood samples and total volume of blood to be collected from each subject throughout the duration of the 24-month study are summarized in **Table 13**. A total of approximately 104.5-145.5 mL of blood will be collected from each subject over the course of the up to 24-month study.

Table 13 Blood Sample Number and Volume

Test	Total mL of Blood								
	Day -1[a]	Month 6	Month 9	Month 12	Month 18	Month 21	Month 24	End of Dose-tapering Period[b]	Total Volume
Clinical Safety Labs[c]	4.5	4.5		4.5	4.5		4.5	4.5	18-27
PD Biomarker Panel	15[d]	17[e]		17[e]	17[e]		17[e]	17[e]	68-100
PD Insulin/Glucose/ Cortisol			5[f]			5[f]			10
DNA Testing							8.5		8.5
Total Volume by Visit (mL)	19.5	21.5	5	21.5	21.5	5	30	21.5	104.5-145.5
**Total Volume: approximately 104.5-145.5 mL**									

[a] Day -1 blood draws for clinical labs and PD biomarkers will not be repeated for subjects whose Day -1 Visit is ≤ 28 days following VBP15-003 Week 24 Final Visit and who had blood drawn for clinical labs and PD biomarkers at that Visit.
[b] Only subjects who participate in the Dose-tapering Period will have bloods drawn.
[c] Hematology, Chemistry, Lipids.
[d] cortisol, P1NP, osteocalcin, 17- hydroxyprogesterone, testosterone, corticosterone, 11-deoxycortisol, CTX, ACTH,and exploratory PD biomarkers.
[e] cortisol, P1NP, osteocalcin, 17- hydroxyprogesterone, testosterone, corticosterone, 11-deoxycortisol, CTX, ACTH, HbA1c, and exploratory PD biomarkers.
[f] Subjects must have fasted ≥ 6 hours prior to blood draws. Blood will be drawn prior to administration of the dose of study medication.

8 DATA COLLECTION

8.1 Source Documents

Source documents are defined as original documents, data, and records. These documents may include hospital records, clinical and office charts, laboratory data/information, subjects' diaries or evaluation checklists, pharmacy dispensing and other records, recorded data from automated instruments, microfilm or magnetic media,

and/or x-rays. Data collected during this study must be recorded on the appropriate source documents.

A subject enrollment log is to be completed at each study site. Data recorded on the enrollment log are to include a subject identifier, the date of enrollment, and the reason the subject was not entered (if applicable). All subjects initially considered for enrollment are to be recorded in this log.

The investigator(s)/institution(s) will permit study-related monitoring, audits, IRB/IEC review, and regulatory inspection(s), providing direct access to source data documents.

8.2 Electronic Case Report Form Completion

Subject data will be collected in this study using an EDC system. The EDC and database system will be OpenClinica by Akaza Research, LLC. OpenClinica is a web-based (https://www.openclinica.com) data entry system utilizing a high security environment. The underlying storage facility will be PostgreSQL, whose structure permits the linking of subject information across all tables in relational databases. OpenClinica uses secure socket layers (SSL) and in its Enterprise version used in this study is 21 Code of Federal Regulations (CFR) Part 11 compliant. Once an eCRF is created in the database, a data dictionary exists and the data team creates compatible paper source documentation.

The Coordinating Center will design an electronic database in OpenClinica for this study. Access rights to the EDC system for the study site team members will need to be requested. Every user of the system will be made aware of the fact that user name and password should never be shared and their electronic signature constitutes the legally binding equivalent of a hand-written signature. Only trained personnel certified by the Coordinating Center will receive a user name and password.

All data will be directly entered or collected on a source document and then entered into OpenClinica or transferred electronically to the study database (e.g., clinical laboratory results, quantitative muscle testing).

The Coordinating Center data management team will monitor the eCRFs for completeness and acceptability throughout the course of the study. ReveraGen personnel

(or their representatives) will be allowed access to all source documents in order to verify eCRF entries.

8.3 Data Processing

A clinical study database will be constructed from the eCRFs and any data merged electronically, and the data will be validated both manually and electronically. Clarification of data will be requested from the study site as required. The database will be quality assured in accordance to the data management plan and will be available for statistical analysis according to the methods outlined in Section **9.6** and the Statistical Analysis Plan (SAP).

8.4 Subject Diaries

The parent or legal guardian of each subject will be given a subject diary at the Baseline Day -1 Visit in which to record any new concomitant medications and any changes to existing concomitant medications taken during the study, as well as any AEs experienced by the subject during the study. Parents/legal guardians will be instructed in how to record information in the diary and will be instructed to bring the diary with them to each study visit for review by study staff for completeness and accuracy. A new diary will be dispensed at each scheduled trimonthly visit for use through the time of the next scheduled trimonthly visit; subject diaries dispensed at the Baseline Day -1 Visit will be returned at the Month 1 Visit for review by study staff and returned to the subjects for continued use through the time of the Month 3 Visit. Collection of final diaries will occur at the Month 24 Visit, or at the end of the Dose-tapering Period for subjects whose vamorolone dose is tapered at the end of the study. The information recorded in the diary will be considered source documentation, and any information recorded in the diary should be transcribed by study staff to the appropriate eCRF.

9 STATISTICAL METHODS AND PLANNED ANALYSES

Eligible subjects are those who enrolled in the VBP15-003 Phase IIa extension study and have completed up to and including the Week 24 final study assessments; up to approximately 50 subjects will be enrolled into VBP15-LTE.

This is an open-label extension study with no placebo control. Historical control data are available for the same age range (4-10 years), at largely the same study sites, with the same outcome measures. Untreated natural history control population data is from the ongoing CINRG DNHS study of ~400 DMD boys.[44,45,46,47] Prednisone-treated and deflazacort-treated historical control data are from a clinical trial of daily dosing of prednisone and deflazacort.[48] The vamorolone VBP15-LTE Phase IIa extension trial is carried out with daily dosing.

9.1 Statistical and Analytical Plan (SAP)

The sections below summarize the intended statistical methods and analyses for the VBP15-LTE extension study. A more detailed SAP will be written and finalized prior to finalization of the clinical study database. The SAP will give a detailed description of the summaries and analyses that will be performed and will clearly describe when these analyses will take place. Any changes to the planned methods and analyses will be described and justified in the SAP and/or in the final clinical study report, as appropriate.

9.1.1 Deviations from the Statistical Analysis Plan

An SAP will be written and finalized prior to any lock of the study database. The SAP will give a detailed description of the summaries and analyses that will be performed and will clearly describe when these analyses will take place. Any deviation(s) from the original SAP will be described and justified in the clinical study report.

9.2 Analysis Populations

Four populations will be defined for data analysis.

9.2.1 Safety Population

All subjects who receive at least one dose of vamorolone study medication in the VBP15-LTE extension study will be included in the Safety Population. The Safety Population is the primary analysis population for safety assessments. This is also the modified Intention to Treat (mITT) population.

9.2.2 Full Analysis Set (FAS)

All subjects who receive at least one dose of vamorolone study medication in the VBP15-LTE extension study and have at least one post-baseline assessment will be included in the FAS. The FAS is the primary analysis population for efficacy and PD assessments. The FAS is the mITT population, with the additional requirement of having at least one post-baseline assessment. Subjects who receive at least one dose of vamorolone but never have post-baseline assessments will be excluded.

9.2.3 Control Population DNHS Study

The control population from the CINRG Duchenne Natural History Study will include all subjects who were observed as part of the study in ages ≥ 4 years and <7 years of age at a start of an interval of observation; observed for at least two years with TTSTAND, TTCLIMB, TTRW, NSAA, 6MWT and QMT measured; remained glucocorticoid-naive during the entire observation period; and were able to walk independently without assistive devices, able to complete the TTSTAND; and lacked any history of disease, impairment, or medications that would have made them ineligible to receive the vamorolone intervention as defined by the VBP15-LTE exclusion criteria at the start of the interval.

9.2.4 Control Population FOR DMD Study

The control population from the FOR DMD study will include all subjects who were 4-7 years old at entry and who were randomized to the daily prednisone or daily deflazacort arm.[48]

9.3 Measures Taken to Avoid/Minimize Bias

Not applicable.

9.4 Interim Analysis

No interim statistical analyses are planned.

9.5 Missing, Unused, and Spurious Data

The possibility of bias from missing data of subjects who prematurely discontinue will be addressed in the clinical study report. Missing values for safety and exploratory outcomes will be treated as missing; while those for efficacy measurements will be imputed using methodology as described in the SAP.

9.6 Statistical Analysis

9.6.1 General Considerations

Statistical analyses will be performed using SAS®.

All measurements will be analyzed based upon the type of distribution, and descriptive statistics will be presented by treatment group and assessment time point, as appropriate. Descriptive statistics for continuous variables (number [N], mean, median, standard deviation [SD], minimum, and maximum), descriptive statistics for categorical variables (N and percentage), and individual subject profiles will be presented, as appropriate.

All statistical tests will be performed at the 0.05 level. Paired t-tests will be utilized for select variables. When available, baseline from the VBP15-002 will be utilized when calculating change from baseline.

9.6.2 Subject Disposition, Demographics, and Baseline Characteristics

Subject disposition will be summarized by analysis population. The number of subjects enrolled, the number in each population, and the reason for discontinuation from the study will be summarized.

Subject demographics (e.g., age, race, and ethnicity) and baseline characteristics (e.g., height, weight, and months/years since DMD diagnosis) will be summarized descriptively by analysis population.

9.6.3 Safety Analyses

All subjects who received at least one dose of vamorolone (Safety Population) will be included in the safety analyses. In general, descriptive statistics for each safety endpoint

will be presented by combined vamorolone dose level, individual dose levels, and other combinations of dose levels.

All safety data will be listed.

Safety data will include BMI (raw units and z-score), height (raw units and z-score), vital signs, clinical laboratory test results, spine x-ray results (spine fractures), hand x-ray results (bone age), and ECG results.

Changes in BMI z-score and height z-score will be analyzed using the same type of statistical models used for efficacy with vamorolone being compared with the daily prednisone and the daily deflazacort arms from the FOR DMD study. Hand x-ray and spine x-ray results will also be compared with the daily prednisone and the daily deflazacort arms from the FOR DMD study.

Adverse events will be summarized overall and by dose level by system organ class (SOC) and preferred term (using the Medical Dictionary for Regulatory Activities [MedDRA]); by relationship to study medication; and by intensity (CTCAE grade).

Physical examination results will be listed only.

9.6.4 Clinical Efficacy and Pharmacodynamic Analyses

The evaluations of clinical efficacy and PD will be performed using the FAS Population.

The primary efficacy outcome is TTSTAND (velocity). Secondary efficacy outcomes are the NSAA assessment, TTCLIMB, TTRW, QMT, and the 6MWT. TTSTAND, TTCLIMB, and TTRW will be analyzed using raw scores and velocity.

The primary outcome is TTSTAND (velocity) change from VBP15-002 study baseline to Month 24 and will be compared between vamorolone and historical untreated controls using a restricted maximum likelihood (REML)-based mixed model for repeated measures (MMRM). This model will include fixed effects for treatment, visit, baseline TTSTAND velocity (from the VBP15-002 study), age at study entry, and the treatment-by-visit interaction. The initial model will compare combined vamorolone doses to untreated natural history controls and subsequent secondary models may compare individual and other combinations of dose levels to untreated natural history controls.

The secondary outcome measures will be compared using similar models. Full details will be provided in the Statistical Analysis Plan (SAP).

Serum PD biomarkers of adrenal axis suppression, insulin resistance, and bone turnover will be assessed, as well as exploratory biomarkers of safety and efficacy. Biomarkers will be summarized descriptively over time.

Individual clinical efficacy and PD data will be listed.

9.6.5 Concurrent Medications

A summary of all concomitant medications taken during the course of the study will be presented in tabular form by therapeutic drug class and generic drug name using the World Health Organization (WHO) Drug classification (Version 4.3). All concomitant medications will be detailed in the subject data listings.

10 STUDY MANAGEMENT AND ETHICAL AND REGULATORY REQUIREMENTS

10.1 Regulatory Approval and Good Clinical Practice

This study will be conducted in accordance with the principles of the 18th World Medical Assembly (Helsinki, June 1964), and amendments of the 29th (Tokyo, 1975), 35th (Venice, 1983), 41st (Hong Kong, 1989), 48th (Somerset West, 1996), 52nd (Edinburgh, 2000), 53rd (Washington, 2002), 55th (Tokyo, 2004), 59th (Seoul, 2008), and 64th (Fortaleza, 2013) World Medical Assemblies.

Further, the trial will be conducted in accordance with:

- International Conference on Harmonisation (ICH) E6 Guideline for Good Clinical Practice (GCP)
- The United States (US) FDA Code of Federal Regulations, Title 21 CFR Part 312 – Investigational New Drug Application, Part 50 – Protection of Human Patients with particular focus in SubPart D, and/or Part 56 – Institutional Review Boards
- US Health Insurance Portability and Accountability Act of 1996 (HIPAA).

10.2 Investigator Responsibilities

10.2.1 Subject Information and Informed Consent

Before being admitted to the VBP15-LTE extension study, a parent/guardian must consent in writing for the subject to participate. Written or verbal assent will also be obtained from each subject as required per regulations. An approved ICF will be given to each parent/guardian which will contain all US federally required elements, all ICH-required elements, and HIPAA authorization information, if applicable, in language that is understandable to the parent/guardian. The consent should note that the Investigator is receiving compensation for the expenses of conducting the study.

The process of obtaining the informed consent will be in compliance with all federal regulations, ICH requirements, and local laws.

The Investigator or designee will review the study with the parent/guardian of each subject. The review will include the nature, scope, procedures, and possible consequences of the subject’s participation in the study. The consent, assent, and review must be in a form understandable to the parent/guardian of the subject. The Investigator or designee and the parent/guardian of the subject must both sign and date the ICF after review and before the subject can participate in the study. The parent/guardian of the subject will receive a copy of the signed and dated form, and the original will be retained in the site study files. The Investigator or designee must emphasize to the parent/guardian of the subject that study participation is entirely voluntary and that consent regarding study participation may be withdrawn at any time without penalty or loss of benefits to which the subject is otherwise entitled.

If the ICF is amended during the study, the Investigator must follow all applicable regulatory requirements pertaining to all new subjects and repeat the consent process with the amended ICF for any ongoing subjects.

10.2.2 Institutional Review Board/Independent Ethics Committee Approval and Other Institutional Requirements

Before the start of the study, the study protocol, ICF, and any other appropriate documents will be submitted to the IRB/IEC for review and approval. Per institutional requirements, the study protocol and any other appropriate documents will be submitted to scientific committees for approval.

The Investigator will forward to the Sponsor, or designee (Coordinating Center), a copy of the IRB/IEC's approval of this protocol, amendments, ICF and any changes to the ICF, based on the FDA regulations set forth in Part 56 of Title 21 of the CFR. The Investigator will also keep documentation of study approval by internal scientific committees per institutional requirements.

Study medication can only be supplied to the Investigator after documentation of all ethical and legal requirements for starting the study has been received by the Sponsor or designee (Coordinating Center). This documentation must also include an IRB/IEC membership list that contains members' occupations and qualifications. If the IRB/IEC will not disclose the names of the committee members, it should be asked to issue a statement confirming that the composition of the committee is in accordance with GCP. The IRB Assurance Number may be accepted as a substitute for the IRB membership list.

The Investigator will keep the IRB/IEC informed regarding the progress of the study, per institutional requirements. No changes will be made in the study without IRB/IEC approval, except when required to eliminate apparent immediate hazards to the subjects.

While the study is ongoing and at study completion/discontinuation, the Investigator must submit to the IRB/IEC the following information in accordance with US Federal regulatory requirements:

1. Information on serious or unexpected AEs, showing due diligence in providing this information as soon as possible
2. Periodic reports on the progress of the study
3. Final Study Summary upon study completion or closure.

Notification of the end of the trial will be sent to the IRB/IEC within 30 days after completion of the study close-out visit. In case the study is ended prematurely, the IRB/IEC will be notified within 15 days, including the reasons for the premature termination. The end of the trial is defined as the date of final analysis of the study data according to the SAP.

10.2.3 Study Documentation

10.2.3.1 Before the Start of the Study

The following study documentation will be in place at the study site prior to the first administration of study drug:

- Fully signed protocol and protocol-supporting manuals
- Investigator's Brochure[29]
- Protocol Acceptance form signed by the Investigator
- IRB/IEC-approved copy of the ICF
- Curriculum vitae of the Investigator and all sub-investigators listed on the FDA Form 1572
- A letter of IRB/IEC approval for protocol
- A list of members of the IRB/IEC and their affiliations
- A copy of the Investigator-signed FDA 1572 form
- An Investigator-signed financial disclosure form.

10.2.3.2 During the Study

The following documentation should be added to the site study file during study conduct:

- Any paper source forms completed and subsequently entered into the study database. An explanation should be given for all missing data and any protocol deviations documented in the site study file
- Any changes to the documentation identified above in Section **10.2.3.1**
- Shipping documents relating to shipment of medication (drug accountability) and bioanalytical samples

- Copies of relevant correspondence such as letters, emails, meeting notes, and telephone calls.

10.2.3.3 After the Study

After completion or premature termination of the trial, all of the documents identified should be in the file, together with the following:

- Study drug accountability documents
- Audit certificates (if applicable)
- Investigator delegation of responsibilities log
- Site signature log
- Subject enrollment log
- Substantive correspondence with the Sponsor and IRB/IEC
- Notification of the end of the trial to the IRB/IEC.

10.2.4 Delegation of Investigator Responsibilities

The Investigator must (a) ensure that any individual to whom a task is delegated is qualified by education, training, and experience (and licensure, if relevant) to perform the task; and (b) provide adequate supervision. The Investigator should maintain a list of sub-investigators and other appropriately qualified persons to whom he or she has delegated significant study-related duties.

10.3 Protocol Deviations and Violations

10.3.1 Protocol Deviation and Violation Definitions

10.3.1.1 Protocol Deviation

A protocol deviation is any change, divergence, or departure from the study design or procedures of a research protocol that is under the Investigator's control and that has not been approved by the IRB/IEC.

Changes or alterations in the conduct of the trial which do not have a major impact on the subject's rights, safety or well-being, or the completeness, accuracy and reliability of the study data are considered minor protocol deviations.

10.3.1.2 Protocol Violation

A protocol violation is a deviation from the IRB/IEC-approved protocol that may affect the subject's rights, safety, or well-being and/or the completeness, accuracy and reliability of the study data. This includes examples such as inappropriate consent, errors in drug dosing, or lack of reporting of safety data.

10.3.2 Reporting Deviations/Violations

Upon discovery of a major protocol deviation or violation, the Investigator is responsible for reporting protocol deviations or violations to the IRB/IEC and Sponsor or designee (Coordinating Center) within 24 hours of discovery.

All other deviations must be reported in writing within 7 days of the event or its discovery.

10.4 Study Records Retention and Direct Access to Source Documents

Federal regulations require that, following completion of a clinical study, a copy of all records of that study be maintained by the Investigator for at least the shorter of the following two time periods:

- Two years after FDA approval of the drug for the indication for which it was investigated, or
- In situations where no application is to be submitted or an application is not approved for such indication, 2 years after the Investigational New Drug (IND) Application is discontinued and the FDA is notified.

The Investigator must maintain a copy of all data collected for each subject treated (including eCRFs and source data). In order to assure the accuracy of data collected in the eCRF, it is mandatory that representatives of the Sponsor, or designee, as well as representatives of the FDA or other health authorities have direct access to original source documents (e.g., subject records, subject charts, and laboratory reports). During the review of these documents, the anonymity of the subject will be respected with strict adherence to professional standards of confidentiality.

The Sponsor reserves the right to terminate the study for refusal of the Investigator to supply source documentation of work performed in this clinical study.

The following includes, but is not limited to, the records that must be retained by the Investigator:

1. Signed informed consent documents for all subjects
2. Subject enrollment log
3. Record of all relevant communications between the Investigator and the IRB/IEC
4. Composition of the IRB/IEC
5. Record of all relevant communications between the Investigator and the Sponsor (or designee)
6. List of sub-investigators and other appropriately qualified persons to whom the Investigator has delegated significant study-related duties, together with their roles in the study and their signatures
7. Drug accountability records (see Section **5.8.4**)
8. Record of any body fluids or tissue samples retained
9. All other source documents (subject records, hospital records, laboratory records, etc.)
10. All other documents as listed in Section 8 of the ICH consolidated guideline on GCP (Essential Documents for the Conduct of a Clinical Trial).

10.5 Data and Safety Monitoring Board (DSMB) and Other Study Monitoring

10.5.1 DSMB

An unblinded Data and Safety Monitoring Board (DSMB), operating autonomously from the Sponsor and the site investigators, will be responsible for providing independent recommendations to the Sponsor about risk-benefit of the study and for any modification affecting safety or data integrity required during the course of the study. The DSMB members must not be actively involved in study design, conduct or daily management of this study and must not have financial, proprietary, professional, or other interests that may affect impartial, independent decision-making.

Specialists may be invited to participate as non-voting members at any time if additional expertise is desired. The DSMB will formally interact with the Sponsor through the sharing of DSMB meeting minutes.

The DSMB will be responsible for:

- Examining accumulating safety and other relevant data at pre-specified points during the course of the study in order to make recommendations concerning continuation, termination, or modification of the study;
- Examining accumulating clinical efficacy data and comparing to corticosteroids and an untreated historical control group in order to make recommendations concerning continuation, termination, or modification of the study;
- Reviewing protocol violations;
- Providing expert advice to the Sponsor on an ad hoc basis regarding matters such as safety concerns or diagnostic evaluations in individual subjects;
- Based on the results of its deliberations, the DSMB can recommend continuation of the study unchanged, study interruption, study termination, modification of the studies, or alteration in the DSMB monitoring plan.

10.5.2 Study Monitoring

In accordance with applicable regulations, GCP, and the procedures of the Sponsor or its designees, the Study Monitor will periodically contact the site and conduct on-site visits. The extent, nature, and frequency of on-site visits will be based on enrollment rate and data quality at the site. Through frequent communications (e.g., letter, e-mail, and telephone), the Study Monitor will ensure that the investigation is conducted according to protocol and regulatory requirements.

During these contacts, the monitoring activities will include:

1. Checking and assessing the progress of the study
2. Reviewing study data collected to date for completeness and accuracy
3. Reviewing compliance with protocol assessments

4. Conducting source document verification by reviewing eCRF database data against source documents when available (e.g., medical records, subject diaries, ICF [and assent, if applicable], laboratory result reports, raw data collection forms)
5. Identifying any issues and addressing resolutions.

These activities will be done in order to verify that the:

1. Data are authentic, accurate, and complete
2. Safety and rights of the subjects are being protected
3. Study is conducted in accordance with the currently approved protocol (and any amendments), GCP, and all applicable regulatory requirements.

The Investigator will allow the Study Monitor direct access to all relevant documents, and allocate his/her time and the time of his/her staff to the Study Monitor to discuss findings and any relevant issues.

In addition to contacts during the study, the Study Monitor will contact the site prior to the start of the study to discuss the protocol and data collection procedures with site personnel.

At study closure, Study Monitors will conduct all activities as indicated in Section **10.7**.

10.6 Quality Assurance

At its discretion, the Sponsor or its designee may conduct a quality assurance audit of this study. Auditing procedures of the Sponsor and/or its designee will be followed in order to comply with GCP guidelines and ensure acceptability of the study data for registration purposes. If such an audit occurs, the Investigator will give the auditor direct access to all relevant documents, and will allocate his/her time and the time of his/her staff to the auditor as may be required to discuss findings and any relevant issues.

In addition, regulatory authorities and/or the IRB/IEC may conduct an inspection of this study. If such an inspection occurs, the Investigator will allow the inspector direct access to all source documents, eCRFs, and other study documentation for source data check

and/or on-site audit inspection. The Investigator must allocate his/her time and the time of his/her staff to the inspector to discuss findings of any relevant issues.

An explanation will be given for all missing, unused, and spurious data in the relevant section of the study report.

10.7 Study Termination and Site Closure

Upon completion of the study, the following activities, when applicable, must be conducted by the Study Monitor in conjunction with the Investigator, as appropriate:

1. Provision of all study data to the Sponsor
2. Data clarifications and/or resolutions
3. Accounting, reconciliation, and final disposition of used and unused study medication
4. Review of site study records for completeness.

In addition, the Sponsor reserves the right to temporarily suspend or prematurely terminate this study for any reason.

If the study is suspended or terminated for safety reason(s), the Sponsor will promptly inform the Investigator, and will also inform the regulatory authorities of the suspension or termination of the study and the reason(s) for the action. The Investigator is responsible for promptly informing the IRB/IEC, and providing the reason(s) for the suspension or termination of the study.

If the study is prematurely terminated, all study data must be returned to the Sponsor. In addition, the site must conduct final disposition of all unused study medications in accordance with the Sponsor procedures for the study.

10.8 Site Termination

The Sponsor may at any time, at its sole discretion, terminate the study site for various reasons, including, without limitation, the following:

1. Failure of the Investigator to enroll subjects into the study at a reasonable rate
2. Failure of the Investigator to comply with applicable laws and/or pertinent FDA regulations

3. Submission of knowingly false information from the research facility to the Sponsor, Study Monitor, FDA, or other regulatory authorities
4. Insufficient adherence to protocol requirements.

If participation of a study site is terminated, the Sponsor and Study Chair will issue a written notice to the Investigator. The written notice will contain the reasons for taking such action. If the study site is terminated for noncompliance, appropriate regulatory authorities will also be notified by the Sponsor.

Study termination and follow-up will be performed in compliance with the conditions set forth in 21 CFR 312.50 and 21 CFR 312.56.

10.9 Discontinuation of Study

The Sponsor reserves the right to discontinue the study for any reason at any time. In addition, the study may be stopped at any time if, in the opinion of the Sponsor and Medical Monitor, safety data suggest that the medical safety of subjects is being or may become compromised.

11 DISCLOSURE OF DATA

11.1 Confidentiality

The rights and privacy of participants in this study will be protected at all times. All personal details of subjects will be treated as confidential by the Investigator and handling of personal data will be in compliance with HIPAA. All applicable data protection laws in the relevant countries will be adhered to at all times.

Subject names will remain confidential and will not be included in the database. Only enrollment number and birth date will be recorded on the eCRF. If the subject's name appears on any other document collected (e.g., hospital discharge summary), the name must be obliterated before the document is transmitted to the Sponsor or its designee. All study findings will be stored in electronic databases. The subjects' parents or guardians will give explicit permission for representatives of the Sponsor, regulatory authorities, and the IRB/IEC to inspect the subjects' medical records to verify the information collected. The subjects' parents or guardians will be informed that all personal

information made available for inspection will be handled in the strictest confidence and in accordance with all state, local, and federal data protection/privacy laws, including, without limitation, the HIPAA, as applicable.

The parents or guardians of all participants in the United States will provide written authorization to disclose private health information either as a part of the written ICF or as a separate authorization form. The authorization will contain all required elements specified by 21 CFR 50, and will contain a waiver of subject access to study-related private health information until the conclusion of the clinical study. The authorization will remain valid and in full force and effect until the first to occur of (1) the expiration of 2 years after the study medication is approved for the indication being studied, or (2) the expiration of 2 years after the research program is discontinued. Individual subject medical information obtained during this study is confidential, and its disclosure to third parties (other than those mentioned in this section) is strictly prohibited. In addition, medical information obtained during this study may be provided to the subject’s personal physician or to other appropriate medical personnel when required in connection with the subject’s continued health and welfare.

The study Investigator will maintain a subject identification log (enrollment numbers and corresponding subject names) to enable records to be identified.

Study data will be shared with the study ‘Use of Microsoft Bands as an Outcome Measure in Boys with DMD - Parallel study to Clinical Study Protocol VBP15-003’ if the subject’s parent/legal guardian consents for his participation in both studies.

11.2 Publication

ReveraGen BioPharma, Inc. retains the ownership of all data and results collected during this study. Therefore, the Sponsor reserves the right to use the data from this present study, either in the form of eCRFs (or copies of these), or in the form of a report, with or without comments and analysis in order to submit them to the US FDA or the Health Authorities of any country.

Furthermore, in the event that the clinical research leads to patentable results, the Investigator (or entity acting on his/her behalf according to local requirements) shall

refrain from filing patent application(s). Patent applications will be filed by ReveraGen BioPharma, Inc. or another entity delegated by ReveraGen BioPharma, Inc.

All information concerning the product as well as any information such as clinical indications for the drug, its formula, methods of manufacture and other scientific data relating to it, that have been provided by the Sponsor or designee, and are unpublished, are confidential and must remain the sole property of the Sponsor. The Investigator will agree to use the information only for the purposes of carrying out this study and for no other purpose unless prior written permission from the Sponsor is obtained. The Sponsor has full ownership of the eCRFs completed as part of the study.

By signing the study protocol, the Investigator agrees that the results of the study may be used for the purposes of national and international registration, publication, and information for medical and pharmaceutical professionals by the Sponsor. If necessary, the authorities will be notified of the Investigator's name, address, qualifications, and extent of involvement.

The Sponsor or designee will prepare a final report on the study. The Investigator may not publish or present any information on this study without the express written approval of the Sponsor. Additionally, the Sponsor, may, for any reason, withhold approval for publication or presentation.

12 INVESTIGATOR’S PROTOCOL AGREEMENT

The Investigator's Protocol Agreement at the front of this document must be signed by the study site Principal Investigator. The original *or a copy* must be kept on file by the Sponsor and the Investigator must retain the original *or a copy*. The completed Protocol Agreement signifies review and acceptance of the protocol by the Principal Investigator prior to initiation of the study.

13 REFERENCES

1. Mah JK, Korngut L, Dykeman J, Day L, Pringsheim T, Jette N. A systemic review and meta-analysis on the epidemiology of Duchenne and Becker muscular dystrophy. *Neuromuscul Disord*. 2014; 24(6): 482-91.
2. Hoffman EP, Brown RH and Kunkel LM. Dystrophin: the protein product of the Duchenne muscular dystrophy locus. *Cell*. 1987; 51: 919-28.
3. Evans NP, Misyak SA, Robertson JL, Bassaganya-Riera J, Grange RW. Immune mediated mechanisms potentially regulate the disease time course of Duchenne muscular dystrophy and provide targets for therapeutic intervention. *PM R*. 2009; 1(8): 755-68.
4. Kumar A and Boriek AM. Mechanical stress activates the nuclear factor-kappa B pathway in skeletal muscle fibers: a possible role in Duchenne muscular dystrophy. *FASEB J*. 2003; 17(3): 386-96.
5. Acharyya S, Villalta SA, Bakkar N, et al. Interplay of IKK/NF-kappa B signaling in macrophages and myofibers promotes muscle cell degeneration in Duchenne muscular dystrophy. *J Clin Invest*. 2007; 117(4): 889-901.
6. Dogra C, Changotra H, Wergedal JE, Kumar A. Regulation of phosphatidylinositol-3-kinase (PI3K)/Akt and nuclear factor-kappa B signaling pathways in dystrophin-deficient skeletal muscle in response to mechanical stretch. *J Cell Physiol*. 2006; 208(3): 575-85.
7. Pahl HL. Activators and target genes of Rel/NF-kappa B transcription factors. *Oncogene*. 1999; 18(49): 6853-66.
8. Spencer MJ, Montecino-Rodriguez E, Dorshkind K, Tidball JG. Helper (CD4(+)) and cytotoxic (CD8(+)) T cells promote the pathology of dystrophin-deficient muscle. *Clin Immunol*. 2001; 98(2): 235-43.
9. Spencer MJ and Tidball JG. Do immune cells promote the pathology of dystrophin-deficient myopathies? *Neuromuscul Disord*. 2001; 11(6-7): 556-64.
10. Malik V, Rodino-Klapac L and Mendell JR. Emerging drug for Duchenne muscular dystrophy. *Expert Opin Emerg Drugs*. 2012; 17(2): 261-77.
11. Reeves EK, Hoffman EP, Nagaraju K, Damsker JM, McCall JM. VBP15: preclinical characterization of a novel anti-inflammatory delta 9,11 steroid. *Bioorg Med Chem*. 2013; 21(8): 2241-9.

12. Bushby K, Finkel R, Birnkrant DJ, et al. Diagnosis and management of Duchenne muscular dystrophy, part 1: diagnosis, and pharmacological and psychosocial management. *Lancet Neurol*. 2010; 9(1): 77-93.

13. Pane M, Mazzone ES, Sivo S, et al. Long term natural history data in ambulant Boys with Duchenne muscular dystrophy: 36-month changes. *PlosOne.* 2014; 10: 1371/journal.pone.0108205.

14. Manzur AY, Kuntzer T, Pike M, Swan AV. Glucocorticoid corticosteroids for Duchenne muscular dystrophy. *Cochrane Database Syst Rev.* 2008; (1): CD003725.

15. Heier CR, Damsker JM, Yu Q, et al. VBP15, a novel anti-inflammatory and membrane-stabilizer improves muscular dystrophy without side effects. *EMBO Mol Med*. 2013; 5(10): 1569-85.

16. Damsker JM, Dillingham BC, Rose MC, et al. VBP15, a glucocorticoid analogue, is effective at reducing allergic lung inflammation in mice. *Plos One*. 2013; 8(5): e63871.

17. Dillingham BC, Knoblach SM, Many GM, et al. VBP15, a novel anti-inflammatory, is effective at reducing the severity of murine experimental autoimmune encephalomyelitis. *Cell Mol Neurobiol.* 2015; 35(3): 377-87.

18. Dadgar S, Wang Z, Johnston H, et al. Asynchronous remodeling is a driver of failed regeneration in Duchenne muscular dystrophy. *J Cell Biol*. 2014; 207(1): 139-58.

19. Raman SV, Hor KN, Mazur W, Halnon NJ, Kissel JT, He X, Tran T, Smart S, McCarthy B, Taylor MD, Jefferies JL, Rafael-Fortney JA, Lowe J, Roble SL, Cripe LH. Eplerenone for early cardiomyopathy in Duchenne muscular dystrophy: a randomised, double-blind, placebo-controlled trial. *Lancet Neurol.* 2015; 14(2): 153-61.

20. Avioli LV. Glucocorticoid effects on statural growth. *Br J Rheumatol*. 1993; 32 Suppl 2: 27-30.

21. Wolthers OD and Pedersen S. Short term linear growth in asthmatic children during treatment with prednisolone. *BMJ*. 1990; 301(6744): 145-8.

22. Bircan Z, Soran M, Yildirim I, et al. The effect of alternate-day low dose prednisolone on bone age in children with steroid dependent nephrotic syndrome. *Int Urol Nephrol*. 1997; 29(3): 357-61.

23. Manolagas SC, Weinstein RS. New developments in the pathogenesis and treatment of steroid-induced osteoporosis. *J Bone Miner Res*. 1999; 14(7): 1061-6.

24. Sali A, Guerron AD, Gordish-Dressman H, et al. Glucocorticoid-treated mice are an inappropriate positive control for long-term preclinical studies in the mdx mouse. *Plos One*. 2012; 7(4): e34204.8

25. Kauh E, Mixson L, Malice M-P, Mesens S, Ramael S, Burke J, Reynders T, Van Dyck K, Beals C, Rosenberg E, and Ruddy M. Prednisone affects inflammation, glucose tolerance, and bone turnover within hours of treatment in healthy individuals. *European J Endocrinol.* 2012; 166: 459–67.

26. Wehling-Henricks M, Oltmann M, Rinaldi C, Myung KH, and Tidball JG. Loss of positive allosteric interactions between neuronal nitric oxide synthase and phosphofructokinase contributes to defects in glycolysis and increased fatigability in muscular dystrophy. *Hum Mol Genet*. 2009; 18(18): 3439-51.

27. Younes M, Neffati F, Touzi M, Hassen-Zrour S, Fendri Y, Bejia I, Ben Amor A, Bergaoui N, and Najjar MF. Systemic effects of epidural and intraarticular glucocorticoid injections in diabetic and non-diabetic patients. *Joint Bone Spine*. 2007; 74(5): 472-6.

28. Moon HJ, Choi KH, Lee SI, Lee OJ, Shin JW, and Kim TW. Changes in blood glucose and cortisol levels after epidural or shoulder intra-articular glucocorticoid injections in diabetic or nondiabetic patients. *Am J Phys Med Rehabil*. 2014; 93(5): 372-8.

29. Investigator’s Brochure, Version 8, Vamorolone (17α,21-dihydroxy-16α-methyl-pregna-1,4,9(11)-triene-3,20-dione) 1.33% and 4% Oral Suspension, ReveraGen BioPharma, Inc., September 24, 2018.

30. van Staa TP, Leufkens HG, Abenhaim L, Zhang B, Cooper C. Oral corticosteroids and fracture risk: relationship to daily and cumulative doses. *Rheumatology (Oxford)*. 2000 Dec; 39(12): 1383-9.

31. Hoffman EP, Reeves E, Damsker J, Nagaraju K, McCall JM, Connor EM, Bushby K. Novel approaches to corticosteroid treatment in Duchenne muscular dystrophy. *Phys Med Rehabil Clin N Am.* 2012; 23(4): 821-8.

32. Ma J, McMillan HJ, Karagüzel G, Goodin C, Wasson J, Matzinger MA, Scharke M, Desclouds P, Cram D, Page M, Konji VN, Lentle B and Ward LM. The time to and determinants of first fractures in boys with Duchenne Muscular Dystrophy. Epub 2016 *Osteoporosis International*. November, 2016. DOI: http://dx.doi.org/10.1007/s0019.

33. Larson CM and Henderson RC. Bone mineral density and fractures in boys with Duchenne muscular dystrophy. *J Pediatr Orthop*. 2000; 20: 71-4.

34. McDonald DG, Kinali M, Gallagher AC, et al. Fracture prevalence in Duchenne muscular dystrophy. *Developmental Medicine and Child Neurolog.* 2002; 44: 695-8.

35. King WM, Ruttencutter R, Nagaraja HN, et al. Orthopedic outcomes of long-term daily corticosteroid treatment in Duchenne muscular dystrophy. *Neurology,* 2007; 68: 1607-13.

36. Cummings EA, Ma J, Fernandez CV, et al. Incident vertebral fractures in children with leukemia during the four years following diagnosis. *J Clin Endocrinol Metab,* 2015; 100: 3408-17.

37. Rodd C, Lang B, Ramsay T, et al. Incident vertebral fractures among children with rheumatic disorders 12 months after glucocorticoid initiation: a national observational study. *Arthritis care & research.* 2012; 64: 122-31.

38. Medeiros MO, Behrend C, King W, Sanders J, Kissel J, Ciafaloni E. Fat embolism syndrome in patients with Duchenne muscular dystrophy. *Neurology.* 2013; 80: 1350-2.

39. McAdam LC, Rastogi A, Macleod K, Douglas Biggar W. Fat Embolism Syndrome following minor trauma in Duchenne muscular dystrophy. *Neuromuscul Disord*. 2012; 22: 1035-9.

40. Gordon KE, Dooley JM, Sheppard KM, MacSween J, Esser MJ. Impact of bisphosphonates on survival for patients with Duchenne muscular dystrophy. *Pediatrics.* 2011; 127: e353-8.

41. Fleishaker DL, Mukherjee A, Whaley FS, Daniel S, Zeiher BG. Safety and pharmacodynamic dose response of short-term prednisone in healthy adult subjects: a dose ranging, randomized, placebo-controlled, crossover study. BMC Musculoskelet Disord. 2016 Jul 16;17:293.

42. Hathout Y, Brody E, Clemens PR, et al. Large-scale serum protein biomarker discovery in Duchenne muscular dystrophy. *Proc Natl Acad Sci USA.* 2015; 112(23): 7153-8.

43. Conklin LS, Damsker JM, Hoffman EP, et al. Phase IIa trial in Duchenne muscular dystrophy shows vamorolone is a first-in-class dissociative steroidal anti-inflammatory drug. *Pharmacol. Res*. 2018 Sep 13; 136:140-50.

44. Bello L, Kesari A, Gordish-Dressman H, et al. Genetic modifiers of ambulation in the Cooperative International Neuromuscular Research Group Duchenne natural history study. *Ann Neurol*. 2015; 77(4):684-696.

45. Spurney C, Shimizu R, Morgenroth LP, et al. Cooperative International Neuromuscular Research Group Duchenne Natural History Study demonstrates insufficient diagnosis and treatment of cardiomyopathy in Duchenne muscular dystrophy. *Muscle Nerve*. 2014; Aug;50(2):250-256.

46. McDonald CM, Henricson EK, Abresch RT, et al. The Cooperative International Neuromuscular Research Group Duchenne natural history study – a longitudinal investigation in the era of glucocorticoid therapy: design of protocol and the methods used. *Muscle Nerve.* 2013; 48(1):32-54.

47. Henricson EK, Abresch RT, Cnaan A, et al. The Cooperative International Neuromuscular Research Group Duchenne natural history study: glucocorticoid treatment preserves clinically meaningful function milestones and reduces rate of disease progression as measured by manual muscle testing and other commonly used clinical trial outcome measures. *Muscle Nerve.* 2013; 48(1):55-67.

48. Guglieri M, Bushby K, McDermott MP, et al. Developing standardized corticosteroid treatment for Duchenne muscular dystrophy. *Contempt. Clin. Trials*. 2017 Jul; 58:34-39.

49. FDA Draft Guidance for Industry: Duchenne muscular dystrophy and related dystrophinopathies: developing drugs for treatment. June 2015.

50. Brooke MH, Griggs RC, Mendell JR, et al. Clinical trial in Duchenne dystrophy. I. The design of the protocol. *Muscle & Nerve.* 1981; 4(3): 186-97.

51. Mazzone ES, Messina S, Vasco G, et al. Reliability of the North Star Ambulatory Assessment in a multicentric setting. *Neuromuscul Disord*. 2009; 19(7): 458-61.

52. McDonald CM, Henricson EK, Han JJ, et al. The 6-minute Walk Test as a new outcome measure in Duchenne muscular dystrophy. *Muscle Nerve.* 2010; 41(4): 500-10.

53. Mayhew JE, Florence JM, Mayhew TP, et al. Reliable surrogate outcome measures in multicenter clinical trials of Duchenne muscular dystrophy. *Muscle Nerve.* 2007; 35(1): 36-42.

14 APPENDICES

Appendix 14.1 Vamorolone Dose Calculation Worksheet and Bulk Dispensing Guide

Vamorolone is administered once daily as a 4% oral suspension. The volume per dose is determined by the subject's dosing level and body weight (in kg) at each study medication dispensing visit, as shown by Equation 1:

Equation 1

$$\frac{\textbf{Subject Weight (kg) x Dose Level (mg/kg)}}{\textbf{40 mg/mL}} = \textbf{Subject Dose (in mL)}$$

Subject weight (in kg) should be rounded to the nearest whole integer for the calculation of volume per dose.

Calculated dose volume will be rounded to the nearest **0.01 mL (1 mL syringe),** 0.1 mL **(3 mL syringe),** or 0.2 mL **(5 mL syringe)**, depending upon the total dose volume and calibration of the volumetric syringe to be used for administration of dose, as shown in the examples below:

Example 1: Dose volume calculation for a subject receiving **0.25** mg/kg/day with a body weight of 27 kg:

$$\frac{\textit{Subject Weight (27 kg) x Dose Level (0.25 mg/kg)}}{\textit{40 mg/mL}} = \textit{0.169 mL}$$

The subject will receive a daily dose volume of **0.169** mL per dose throughout the period until the next scheduled visit. Dose will be administered using a **1** mL volumetric syringe rounded to the nearest 0.**0**1 mL, or **0.17** mL daily.

Example 2: Dose volume calculation for a subject receiving **6.0** mg/kg/day with a body weight of 23 kg:

$$\frac{\textit{Subject Weight (23 kg) x Dose Level (6.0 mg/kg)}}{\textit{40 mg/mL}} = \textit{3.45 mL}$$

The subject will receive a daily dose volume of **3.45** mL per dose throughout the period until the next scheduled visit. Dose will be administered using a 5 mL volumetric syringe rounded to the nearest 0.2 mL, or **3.4** mL daily.

Vamorolone 4% Suspension Dispensing Guide

Each subject enrolled in the study will be dispensed 100 ml bottle(s) of vamorolone 4% suspension at the Baseline Day -1 Visit, sufficient for dosing through the time of the next

scheduled trimonthly visit. Additional supplies will be dispensed at each scheduled trimonthly visit, through Month 24. Subjects whose vamorolone dose is tapered during the Dose-tapering Period will be dispensed additional vamorolone 4% suspension at the Month 24 Visit, sufficient for dose tapering to 0 mg/kg/day (see Section **6.3.4**).

The number of bottles to be dispensed at each dispensing visit for the following 3-month dosing period until the next scheduled trimonthly visit is calculated by multiplying the subject's daily dose (in mL/day) (as calculated by Equation 1) by 100 days (93 days in the dosing period + 7 days overage) and dividing that number by 100 mL, the number of mL per bottle, as shown in Equation 2, below:

Equation 2

$$\frac{\textbf{Daily Dose (mL/day) x 100 Days}}{\textbf{100 mL}} = \textbf{\#Bottles to be Dispensed for 3 months dosing}$$

<u>**Example 3:** Calculation of number of bottles to be dispensed for 3-month dosing for a subject taking a daily dose of **0.17** mL (from Example 1 above):</u>

$$\frac{\textbf{Daily Dose (0.17 mL/day) x 100 Days}}{\textbf{100 mL}} = \textbf{0.17 Bottles}$$

The number of bottles dispensed is rounded to the nearest whole integer, or 1 bottle to be dispensed.

<u>**Example 4:** Calculation of number of bottles to be dispensed for 3-month dosing for a subject taking a daily dose of **3.4** mL (from Example 2 above):</u>

$$\frac{\textbf{Daily Dose (3.45 mL/day) x 100 Days}}{\textbf{100 mL}} = \textbf{3.45 Bottles}$$

The number of bottles dispensed is rounded to the nearest whole integer, or 4 bottles to be dispensed.

Each bulk bottle should be used for one subject only. Each bottle dispensed to the subject and ready for administration to subjects will be labeled with subject number, dispense date, protocol number, dose level, and volume to be administered per dose.

Any unused or partially used drug product should be returned at each scheduled trimonthly visit through Month 24 and at the end of the Dose-tapering Period, if

applicable, and retained at the clinical study site for investigational drug accountability monitoring.

Appendix 14.2 Protocol Amendment #1 Complete List of Changes

The following changes have been incorporated into the protocol under this protocol amendment, as summarized in Protocol Amendment Tracking, Reasons for Protocol Amendment #1. Protocol sections that have been changed are itemized below with the original and revised text. Changes that were strictly editorial (e.g. punctuation, correction of spelling errors, grammar) are not included.

Section Changed:

Synopsis, Study Period

Original Text:

**Study Period**	First subject enrolled: 1Q 2017<br>Last subject last visit: 1Q 2021

Revised Text:

**Study Period**	First subject enrolled: 1Q 2017<br>Last subject last visit: 2Q 2020

Section Changed:

Synopsis, Study Summary
Paragraph 7

Original Text:

In the event any clinical observation suggests an intolerability for an individual subject to the study medication, in the opinion of the Investigator, the subject's dose level may be decreased to the next lower dose level (e.g., a subject taking 6.0 mg/kg/day may be decreased to 2.0 mg/kg/day) and maintained at that lower dose level throughout the duration of the Treatment Period. In the event the next lower dose level is also not tolerated and is considered a safety risk to the subject, in the opinion of the Investigator, Study Chair, and Medical Monitor, the subject will be withdrawn from the study.

Revised Text:

In the event any clinical observation suggests an intolerability for an individual subject to the study medication, in the opinion of the Investigator, the subject's dose level may be decreased to the next lower dose level and maintained at that lower dose level throughout the duration of the Treatment Period, with the caveat stated below for subjects de-escalating from 6.0 mg/kg/day to 2.0 mg/kg/day. In the event the next lower dose level is also not tolerated and is considered a safety risk to the subject, in the opinion of the Investigator, Study Chair, and Medical Monitor, the subject will be withdrawn from the study. Subjects whose dose is decreased from 6.0 mg/kg/day to 2.0 mg/kg/day may have their dose subsequently increased

to 4.0 mg/kg/day if they have been taking the 2.0 mg/kg/day dose for at least one month and, in the opinion of the Investigator, balancing efficacy with safety concerns, they could benefit from an intermediate higher dose.

Section Changed:

Synopsis, Safety Measures
Bullets #3 and #7

Original Text:

- Weight
- Body Mass Index (BMI)

- 12-lead electrocardiogram (ECG)
- Clinical signs and symptoms (AEs and SAEs)

Revised Text:

- Weight
- Height
- Body Mass Index (BMI)

- 12-lead electrocardiogram (ECG)
- Hand x-ray
- Spine x-ray
- Clinical signs and symptoms (AEs and SAEs)

Section Changed:

Synopsis, Exploratory Measures

Original Text:

- Measures of serum proteins (SomaSCAN aptamer panel; proteomics profiling; steroid hormones; other biomarkers)
- Quality of life (PODCI)

Revised Text:

- Measures of serum PD biomarkers
- Quality of life (PODCI)
- DNA testing for established genetic modifiers of DMD

Section Changed:

Synopsis, Statistical Methods

Original Text:

Analysis Populations:

All analyses will be based on the actual treatment each subject received. Four populations will be defined for data analysis: the Safety Population, the Full Analysis Set, the control population CINRG Duchenne Natural History Study, and the control population CINRG Prednisone study.

Safety Population

All subjects who receive at least one dose of vamorolone study medication in the VBP15-LTE extension study will be included in the Safety Population. The Safety Population is the primary analysis population for safety assessments. This is also the modified Intention to Treat (mITT) population.

Full Analysis Set (FAS)

All subjects who receive at least one dose of vamorolone study medication in the extension study and have at least one post-baseline assessment will be included in the FAS. The FAS is the primary analysis population for clinical efficacy and PD assessments. The FAS population is the mITT population, with the additional requirement of having at least one post-baseline assessment. Subjects who receive at least one dose of vamorolone but never have post-baseline assessments will be excluded.

Control Populations from CINRG Duchenne Natural History Study

The control population from the CINRG Duchenne Natural History Study will include all subjects who were observed as part of the study in ages ≥ 4 years and <7 years of age at a start of an interval of observation; observed for at least one year with at least two visits in a time interval of no more than 15 months with TTSTAND, TTCLIMB, TTRW, NSAA, 6MWT and QMT measured; remained glucocorticoid-naive during the entire observation period; and were able to walk independently without assistive devices, able to complete the TTSTAND; and lacked any history of disease, impairment, or medications that would have made them ineligible to receive the vamorolone intervention as defined by the Phase IIa extension study VBP15-LTE exclusion criteria at the start of the interval.

Control Population CINRG Prednisone Study

The control population from the CINRG Prednisone study will include all subjects who were younger than 7 years old at entry and who were randomized to the daily prednisone arm.

General Statistical Considerations:

All measurements will be analyzed based upon the type of distribution and descriptive statistics presented by treatment group and time point, as appropriate. No interim statistical analyses are planned. Missing values for safety and exploratory outcomes will be treated as missing.

Baseline measurement is defined as the last non-missing value prior to the first dose of study drug in the extension study.

Efficacy Analyses:

The primary efficacy outcome is TTSTAND. Secondary efficacy outcomes are the NSAA assessment, TTCLIMB, TTRW, QMT, and the 6MWT**.** The primary outcome will be compared between vamorolone and historical untreated controls using a mixed-effects linear model with treatment group. Age at study entry will be included as a covariate. The initial model will compare combined vamorolone doses to untreated natural history controls and a subsequent secondary model will compare each dose to untreated natural history controls. This will allow the testing of whether vamorolone in general and vamorolone at individual doses have significant effects on the slope of the velocity. Additional hypotheses of efficacy will include similar linear modelling with the secondary outcomes. They will also include comparison groups including prednisone-treated or untreated natural history controls.

Pharmacodynamics Analyses:

Serum PD biomarkers of adrenal axis suppression, insulin resistance, and bone turnover will be assessed, as well as exploratory biomarkers of safety and efficacy. Longitudinal analysis of PD biomarkers will be studied as a function of treatment, with comparison to prednisone-treated and untreated historical controls, as appropriate.

Safety Analyses:

All evaluations of clinical safety will be listed with descriptive statistics by treatment group presented. The primary safety variable will be BMI z-score and will be assessed using the same type of statistical models used for efficacy. Additional secondary safety data will include height z-score, blood pressure, and ECG results. As no change in blood pressure or cardiac results is expected, these data will be analyzed using an analysis of covariance (ANCOVA) approach which includes the baseline value for each measurement as a covariate. Changes in height z-scores, for which an observed change is expected, will be modelled using a linear mixed effects model. Continuous, quantitative laboratory values will be analyzed similar to BMI z-scores. Categorized laboratory values and presence or absence of AEs will be compared using an exact chi-square test. Adverse events will be summarized overall and by dose level, system organ class (SOC) and preferred term (using the Medical Dictionary for Regulatory Activities [MedDRA]); by dose level and relationship to study medication; and by dose level and intensity (CTCAE grade). Additional hypotheses of safety will include similar linear modelling with the secondary and exploratory outcomes.

Revised Text:

Analysis Populations:

Four populations will be defined for data analysis: the Safety Population, the Full Analysis Set, the control population CINRG Duchenne Natural History Study, and the control population FOR DMD Study.

<u>Safety Population</u>

All subjects who receive at least one dose of vamorolone study medication in the VBP15-LTE extension study will be included in the Safety Population. The Safety Population is the primary analysis population for safety assessments. This is also the modified Intention to Treat (mITT) population.

<u>Full Analysis Set (FAS)</u>

All subjects who receive at least one dose of vamorolone study medication in the VBP15-LTE extension study and have at least one post-baseline assessment will be included in the FAS. The FAS is the primary analysis population for clinical efficacy and PD assessments. The FAS population is the mITT population, with the additional requirement of having at least one post-baseline assessment. Subjects who receive at least one dose of vamorolone but never have post-baseline assessments will be excluded.

<u>Control Population from CINRG Duchenne Natural History Study</u>

The control population from the CINRG Duchenne Natural History Study will include all subjects who were observed as part of the study in ages ≥ 4 years and <7 years of age at a start of an interval of observation; observed for at least two years with TTSTAND, TTCLIMB, TTRW, NSAA, 6MWT and QMT measured; remained glucocorticoid-naive during the entire observation period; and were able to walk independently without assistive devices, able to complete the TTSTAND; and lacked any history of disease, impairment, or medications that would have made them ineligible to receive the vamorolone intervention as defined by the VBP15-LTE exclusion criteria at the start of the interval.

<u>**C**ontrol Population FOR DMD Study</u>

The control population from the FOR DMD Study will include all subjects who were 4-7 years old at entry and who were randomized to the daily prednisone or daily deflazacort arm.

General Statistical Considerations:

All measurements will be analyzed based upon the type of distribution and descriptive statistics presented by treatment group and time point, as appropriate. No interim statistical analyses are planned. Missing values for safety and exploratory outcomes will be treated as missing.

All statistical tests will be performed at the 0.05 level. Paired t-tests will be utilized for select variables. When available, baseline from the VBP15-002 will be utilized when calculating change from baseline.

Efficacy Analyses:

The primary efficacy outcome is TTSTAND (velocity). Secondary efficacy outcomes are the NSAA assessment, TTCLIMB, TTRW, QMT, and the 6MWT**.** TTSTAND, TTCLIMB, and TTRW will be analyzed using raw scores and velocity.

The primary outcome is TTSTAND (velocity) change from VBP15-002 study baseline to Month 24 and will be compared between vamorolone and historical untreated controls using a restricted maximum likelihood (REML)-based mixed model for repeated measures (MMRM). This model will include fixed effects for treatment, visit, baseline TTSTAND velocity (from the VBP15-002 study), age at study entry, and the treatment-by-visit interaction. The initial model will compare combined vamorolone doses to untreated natural history controls and subsequent secondary models may compare individual and other combinations of dose levels to untreated natural history controls. The secondary outcome measures will be compared using similar models. Full details will be provided in the Statistical Analysis Plan (SAP).

Pharmacodynamics Analyses:

Serum PD biomarkers of adrenal axis suppression, insulin resistance, and bone turnover will be assessed, as well as exploratory biomarkers of safety and efficacy. Biomarkers will be summarized descriptively over time.

Safety Analyses:

All subjects who received at least one dose of vamorolone (Safety Population) will be included in the safety analyses. In general, descriptive statistics for each safety endpoint will be presented by the combined vamorolone dose level, individual dose levels, and other combinations of dose levels.

Safety data will include BMI (raw units and z-score), height (raw units and z-score), vital signs, clinical laboratory test results, hand x-ray, spine x-ray, and ECG results.

Changes in BMI z-score and height z-score will be analyzed using the same type of statistical models used for efficacy with vamorolone being compared with the daily prednisone and the daily deflazacort arms from the FOR DMD study. Hand x-ray and spine x-ray results will also be compared with the daily prednisone and the daily deflazacort arms from the FOR DMD study.

Adverse events will be summarized overall and by dose level by system organ class (SOC) and preferred term (using the Medical Dictionary for Regulatory Activities [MedDRA]); by relationship to study medication; and by intensity (CTCAE grade).

Section Changed:

List of Abbreviations

Original Text:

DMD	Duchenne muscular dystrophy
DSMB	Data and Safety Monitoring Board
ECG	electrocardiogram

Revised Text:

DMD	Duchenne muscular dystrophy
DNA	deoxyribonucleic acid
DSMB	Data and Safety Monitoring Board
ECG	electrocardiogram

Original Text:

GGT	gamma glutamyl transferase
GLP	Good Laboratory Practice

Revised Text:

GGT	gamma glutamyl transferase
GLDH	glutamate dehydrogenase
GLP	Good Laboratory Practice

Section Changed:

1.3.2 Phase II Study in 4 to 7 years Duchenne Muscular Dystrophy Boys

Original Text:

1.3.2 Phase II Study in 4 to 7 years Duchenne Muscular Dystrophy Boys

Revised Text:

1.3.2 Pharmacokinetics in Phase II Study in 4 to 7 years Duchenne Muscular Dystrophy Boys (VBP15-002)

Section Changed:

1.3 Clinical Experience
[Text inserted following Section 1.3.2]

Original Text:

[none]

Revised Text:

1.3.3 Safety in Phase II Studies in 4 to7 years Duchenne Muscular Dystrophy Boys (VBP15-002 and VBP15-003)

Adverse events: There were no serious adverse events (SAEs) reported over the 14-day treatment in the Phase I clinical trial in healthy adult volunteers, nor in the four cohorts (0.25 mg/kg, 0.75 mg/kg, 2.0 mg/kg, and 6.0 mg/kg) of the Phase IIa study (VBP15-002; 14-day treatment) in boys ages 4 to <7 years with DMD. There has been a total of 4 SAEs in the Phase IIa VBP15-003 study and one SAE to date in the VBP15-LTE extension study: two SAEs of pneumonia in two different subjects (both subjects

receiving vamorolone 0.75 mg/kg/day), one SAE of bilateral testicular torsion and one SAE of hypoxia in the same subject receiving 6.0 mg/kg/day, and one SAE of influenza associated dehydration in a subject receiving 6.0 mg/kg/day. Each of these SAEs was considered unrelated to study drug, and none of them resulted in discontinuation from the study. In the VBP15-003 study, there were a total of 218 TEAEs among 42 of the 48 subjects (87.5%). In VBP15-003 study, the TEAEs with the highest incidence were viral upper respiratory tract infection (41.7%); pyrexia (35.4%); cough (18.8%); vomiting (14.6%); and diarrhea (10.4%).

Body Mass Index (BMI): Body Mass Index was measured throughout the VBP15-003 study. The mean change from baseline to Week 24 for BMI was 0.03, 0.20, 0.23, and 1.15 for the 0.25, 0.75, 2.0, and 6.0 mg/kg/day dose level groups, respectively. Body Mass Index increases generally reflect an increase in weight. Body Mass Index z-score was monitored in the VBP15-003 study. The mean change from baseline to Week 24 for BMI z-score for the 6.0 mg/kg/day group showed a statistically significant increase compared to the mean change from baseline to Week 24 for the 0.25 mg/kg/day and 0.75 mg/kg/day dose level groups. In contrast, the mean change from baseline to Week 24 for the 2.0 mg/kg/day dose level group in BMI z-score was minimal and comparisons with the other vamorolone dose level groups lacked statistical significance. The mean increase from baseline to Week 24 for BMI z-score was similar for the 6.0 mg/kg/day group and a daily prednisone-treated historical control group.

Potential liver toxicity: In the Phase I clinical trial in adult volunteers, vamorolone showed mild elevations of liver enzymes in one subject receiving 20.0 mg/kg in the fasted state, and dosing was halted. All DMD subjects have elevated serum ALT and AST enzymes because of the muscle condition. For that reason, two enzymes, glutamate dehydrogenase (GLDH) and gamma glutamyl transferase (GTT), that are preferentially expressed in liver were evaluated in the VBP15-003 study. None of the mean changes in GLDH from baseline to any of the VBP15-003 on-treatment assessment time points were statistically significant for any dose level group. Although the mean changes from baseline to Week 8, Week 16, and Week 24 in GLDH levels did not show a dose response, shift analysis of GLDH levels did suggest a possible dose-related shift to higher

GLDH levels at 2.0 mg/kg/day and 6.0 mg/kg/day at Weeks 16 and 24. Mean GGT levels and individual subject values at each VBP15-003 assessment time point across the four dose level groups remained at or below the normal range. On the basis of these mean GLDH and GGT data, vamorolone at dose levels up to 6.0 mg/kg/day does not appear to induce liver toxicity over a 24-week treatment period.

Adrenal suppression: In the VBP15-003 study, after 24 weeks of treatment, 0 of 8 tested participants (0.25 mg/kg/day), 1 of 12 (8.3%) tested participants (0.75 mg/kg/day), 5 of 12 (41.7%) tested participants (2.0 mg/kg/day), and 8 of 9 (88.9%) tested participants (6.0 mg/kg/day) had a depressed morning cortisol (<3.6 µg/dL [100 nM]) consistent with chronic adrenal suppression.[29]

Insulin resistance: In the VBP15-003 study, mean changes from baseline for fasting insulin showed dose-and time-related changes for all dose level groups at Week 12 and Week 24. Statistical significance was observed for mean increase from baseline for the 6.0 mg/kg/day dose level group at Week 12 and Week 24.

Bone Turnover: In the VBP15-003 study, pharmacodynamic biomarker testing for bone turnover markers suggested that vamorolone does not have the detrimental bone effects observed with prednisone and deflazacort.

Section Changed:

1.4 Rationale for Study Design
Paragraphs 4, 5, and 8

Original Text:

The vamorolone development program has made advances in biomarker discovery and development in DMD, with a subset of these studies recently published.[42] A panel of biomarkers in DMD patient sera that show response to chronic (~4 month) treatment with glucocorticoids has been identified. These chronic prednisone PD biomarkers were discovered through study of serum samples of DMD patients enrolled in the Cooperative International Neuromuscular Research Group (CINRG) Duchenne Natural History Study (DNHS).

Revised Text:

The vamorolone development program has made advances in biomarker discovery and development in DMD, with a subset of these studies recently published.[42] A panel of biomarkers in DMD patient sera that show response to chronic (~4 month) treatment with glucocorticoids has been identified. These chronic prednisone PD biomarkers were discovered through study of serum samples of DMD patients enrolled in the Cooperative International Neuromuscular Research Group (CINRG) Duchenne Natural History Study (DNHS), and data have been reported for the VBP15-002 study.[43]

Original Text:

Adrenal axis suppression is a well-documented safety signal with chronic use of glucocorticoids. As noted above, nonclinical data suggest that vamorolone may not cause adrenal axis suppression. To test whether vamorolone treatment causes chronic adrenal axis suppression, four steroid hormones will be tested by LC-MS in the vamorolone-treated DMD boys (17-hydroxyprogesterone, cortiscosterone, testosterone, 11-deoxycortisol) (**Table 7**). All four steroid hormones showed significant reductions after glucocorticoid treatment in DMD boys in the cross-sectional and longitudinal CINRG DNHS participants.

Revised Text:

Adrenal axis suppression is a well-documented safety signal with chronic use of glucocorticoids. As noted in Section **1.3.3**, in the VBP15-003 study, after 24 weeks of treatment, 0 of 8 tested participants (0.25 mg/kg/day), 1 of 12 (8.3%) tested participants (0.75 mg/kg/day), 5 of 12 (41.7%) tested participants (2.0 mg/kg/day), and 8 of 9 (88.9%)

tested participants (6.0 mg/kg/day) had a depressed morning cortisol (<3.6 µg/dL [100 nM]) consistent with chronic adrenal suppression. To further evaluate the effects of vamorolone on chronic adrenal axis suppression, four steroid hormones (17-hydroxyprogesterone, corticosterone, testosterone, 11-deoxycortisol) are being analyzed by LC-MS in the VBP15-LTE study (**Table 7**). All four steroid hormones showed significant reductions after glucocorticoid treatment in DMD boys in the cross-sectional and longitudinal CINRG DNHS participants.

Original Text:

Comparisons of efficacy and safety parameters with historical natural history (untreated)[43,44,45,46] and prednisone-treated control groups[47] will be performed as primary outcomes of this VBP15-LTE study.

Revised Text:

Comparisons of efficacy and safety parameters with historical natural history (untreated)[44, 45, 46, 47] and prednisone- and deflazacort-treated control groups[48] will be performed as outcomes of this VBP15-LTE study.

<u>Section Changed:</u>

<u>1.5 Overall Benefit/Risk</u>

Original Text:

The current study is a long-term safety and efficacy study in young boys with DMD. It is anticipated that the adverse effect profile of the investigational product will be more favorable than standard of care glucocorticoids in the long term. The adverse effects of vamorolone over long term treatment are not currently known. In the Phase I SAD/MAD study in healthy adults, no serious adverse events were observed in any cohort through 2 weeks of treatment. Cohorts tested in the Phase I MAD study were 1.0, 3.0, 9.0 and 20.0 mg/kg/day for 14 days. In the Phase I MAD clinical trial in adult volunteers, vamorolone showed suppression of the adrenal axis at higher doses (9.0 mg/kg/day and 20.0 mg/kg/day in the fasted state).[29] Instructions for detecting adrenal crisis and the circumstances in which stress dose steroids should be provided will be included in the informed consent form (ICF). One human subject in the Phase I studies in the 20.0

mg/kg/day MAD dose group showed mild elevations of liver enzymes, and dosing was halted. Investigators should monitor clinical study participants closely to identify elevations in liver-specific enzymes.

Subjects may or may not receive direct health benefit from participating in the study. Subjects will receive vamorolone at one of four planned dose levels (0.25 mg/kg/day, 0.75 mg/kg/day, 2.0 mg/kg/day, or 6.0 mg/kg/day) over the course of the 24-week VBP15-003 trial, and then continue on this dose in the current VBP15-LTE study for up to 2 years. As noted above, the Medical Monitor, Study Director, and Site Investigator may opt to dose escalate to a higher dose level during the VBP15-LTE once the subject has been on the initial dose in VBP15-LTE for at least 1 month, the next higher dose is determined to be safe in the VBP15-002 Phase IIa Study, and no safety issues with that dose have emerged in the VBP15-003 Phase IIa extension study. While it is anticipated from nonclinical studies that these dose levels may be efficacious in the treatment of DMD, there are no clinical efficacy data yet available to validate this hypothesis. In view of the initial clinical evidence of safety and the monitorable nature of key nonclinical toxicological findings, data support an acceptable risk profile for vamorolone.

Revised Text:

The current study is a long-term safety and efficacy study in young boys with DMD. It is anticipated that the adverse effect profile of the investigational product will be more favorable than standard of care glucocorticoids in the long term (see Section **1.3.3**). The adverse effects of vamorolone over long term treatment are not currently known.

Instructions for detecting adrenal crisis and the circumstances in which stress dose steroids should be provided are included in the informed consent form (ICF), and Investigators should monitor clinical study participants closely to identify elevations in liver-specific enzymes.

Potential health benefits: Subjects may or may not receive direct health benefit from participating in the study. Subjects will receive vamorolone at one of four planned dose levels (0.25 mg/kg/day, 0.75 mg/kg/day, 2.0 mg/kg/day, or 6.0 mg/kg/day) over the course of the 24-week VBP15-003 trial, and then continue on this dose in the current

VBP15-LTE study for up to 2 years. As noted above, the Medical Monitor, Study Director, and Site Investigator may opt to dose escalate to a higher dose level during the VBP15-LTE once the subject has been on the initial dose in VBP15-LTE for at least one month, the next higher dose is determined to be safe in the VBP15-002 Phase IIa Study, and no safety issues with that dose have emerged in the VBP15-003 Phase IIa extension study. In the VBP15-003 study, clinical efficacy was assessed by Timed Function Tests. Improvement in Time to Stand, Time to Climb, Time to Run/Walk 10 Meters, and 6-Minute Walk Test were seen predominantly for the 2.0 and 6.0 mg/kg/day dose level groups, with many of the improvements showing statistical significance compared to an untreated Duchenne Natural History Group. In view of the initial clinical evidence of safety, the improvements seen in assessments of efficacy, and the monitorable nature of key potential adverse effects, data support an acceptable benefit/risk profile for vamorolone.

Sections Changed:

Synopsis: Objectives
2.1 Study Objectives

Original Text:

2.1.1 Primary Objectives

The primary objectives of this study are:

1. To evaluate the long-term safety and tolerability of vamorolone, administered orally at daily doses up to 6.0 mg/kg over a 24-month Treatment Period, in young boys with DMD who completed protocol VBP15-003;
2. To compare the efficacy, as measured by the Time to Stand Test (TTSTAND), of vamorolone administered orally at daily doses up to 6.0 mg/kg over a 24-month Treatment Period vs. untreated DMD historical controls in young boys with DMD; and
3. To compare the safety, as measured by body mass index (BMI) z-score, of vamorolone administered orally at daily doses up to 6.0 mg/kg over a 24-month

Treatment Period vs. prednisone-treated historical controls in young boys with DMD.

2.1.2 Secondary Objectives

The secondary objectives of this study are:

1. To investigate the effects of vamorolone, administered orally at daily doses up to 6.0 mg/kg over a 24-month Treatment Period vs. prednisone-treated historical controls, on serum pharmacodynamic (PD) biomarkers of safety (insulin resistance, adrenal axis suppression, and bone turnover); and
2. To investigate the effects of vamorolone, administered orally at daily doses up to 6.0 mg/kg over a 24-month Treatment Period, on muscle strength, mobility and functional exercise capacity vs. untreated DMD historical controls as measured by Time to Run/Walk Test (TTRW), North Star Ambulatory Assessment (NSAA), Time to Climb Test (TTCLIMB), 6-minute Walk Test (6MWT), and Quantitative Muscle Testing (QMT) in young boys ages with DMD**.**

2.1.3 Exploratory Objectives

The exploratory objectives of this study are:

1. To investigate the effects of vamorolone administered orally at daily doses up to 6.0 mg/kg over a 24-month Treatment Period on Quality of Life measures (Pediatric Outcomes Data Collection Instrument [PODCI]); and
2. To investigate the effects of vamorolone administered orally at daily doses up to 6.0 mg/kg over a 24-month Treatment Period on an extended panel of exploratory PD biomarkers using somaSCAN aptamer arrays, steroid hormone profiling, and other biomarker methods.

Revised Text:

2.1.1 Primary Objectives

The primary objectives of this study are:

1. To evaluate the long-term safety and tolerability of vamorolone, administered orally at daily doses up to 6.0 mg/kg over a 24-month Treatment Period, in young boys with DMD who completed protocol VBP15-003; and

2. To compare the efficacy, as measured by the Time to Stand Test (TTSTAND), of vamorolone administered orally at daily doses up to 6.0 mg/kg over a 24-month Treatment Period vs. untreated DMD historical controls in young boys with DMD.

2.1.2 Secondary Objectives

The secondary objectives of this study are:

1. To investigate the effects of vamorolone, administered orally at daily doses up to 6.0 mg/kg over a 24-month Treatment Period on serum pharmacodynamic (PD) biomarkers of safety (insulin resistance, adrenal axis suppression, and bone turnover);
2. To investigate the effects of vamorolone, administered orally at daily doses up to 6.0 mg/kg over a 24-month Treatment Period, on muscle strength, mobility and functional exercise capacity vs. untreated DMD historical controls as measured by Time to Run/Walk Test (TTRW), North Star Ambulatory Assessment (NSAA), Time to Climb Test (TTCLIMB), 6-minute Walk Test (6MWT), and Quantitative Muscle Testing (QMT) in young boys with DMD; and
3. To compare the safety, as assessed by bone age, spine fractures, BMI z-score, and height z-score, of vamorolone, administered orally at daily doses up to 6.0 mg/kg over a 24-month Treatment Period vs. prednisone- and deflazacort-treated historical control boys with DMD.

2.1.3 Exploratory Objectives

The exploratory objectives of this study are:

1. To investigate the effects of vamorolone administered orally at daily doses up to 6.0 mg/kg over a 24-month Treatment Period on Quality of Life measures (Pediatric Outcomes Data Collection Instrument [PODCI]);
2. To investigate the effects of vamorolone administered orally at daily doses up to 6.0 mg/kg over a 24-month Treatment Period on additional exploratory PD biomarkers; and

3. To determine if established genetic modifiers of DMD (gene polymorphisms associated with disease severity, or response to glucocorticoid treatment) are similarly associated with vamorolone-treated DMD patients (baseline disease severity, or response to vamorolone treatment).

Section Changed:

2.2 Study Endpoints, Safety Endpoints

Original Text:

2.2.1 Safety Endpoints

2.2.1.1 Primary Safety Endpoint

1. BMI z-score: Comparison with a prednisone-treated historical control group for change from Baseline to Month 12.

2.2.1.2 Additional Safety Endpoints

1. BMI z-score: Change from Baseline to each of the scheduled on-treatment and post-treatment assessment time points;
2. Treatment-emergent adverse events (TEAEs) and serious adverse events (SAEs) by system organ class (SOC): Overall by treatment, by treatment and relationship, and by treatment and intensity (see **7.2.6**);
3. Vital signs [blood pressure, heart rate, respiratory rate, oral body temperature]: Change from Baseline to each of the scheduled on-treatment and post-treatment assessment time points;
4. Body weight: Change from Baseline to each of the scheduled on-treatment and post-treatment assessment time points;
5. Clinical laboratory values (hematology and biochemistry): Change from Baseline to each of the scheduled on-treatment and post-treatment assessment time points;
6. Lipid profile (triglycerides, total cholesterol, low density lipoprotein [LDL], high density lipoprotein [HDL]): Change from Baseline to each of the scheduled on-treatment and post-treatment assessment time points;

7. Urinalysis by dipstick and microscopic analysis: Change from Baseline to each of the scheduled on-treatment and post-treatment assessment time points;

8. 12-lead electrocardiogram (ECG): Change from Baseline to each of the scheduled on-treatment and post-treatment assessment time points;

Data for the following additional safety outcomes will be listed only:

1. Physical examination findings at Pretreatment, Month 12, and Month 24.

Revised Text:

2.2.1 Safety Endpoints

1. BMI z-score: Comparison with prednisone- and deflazacort-treated historical control groups for change from Baseline to Month 12 and Month 24;

2. BMI z-score: Change from Baseline to each of the scheduled on-treatment and post-treatment assessment time points;

3. Height z-score: Comparison with prednisone- and deflazacort-treated historical control groups for change from Baseline to Month 12 and Month 24;

4. Treatment-emergent adverse events (TEAEs) and serious adverse events (SAEs) by system organ class (SOC): Overall by treatment, by treatment and relationship, and by treatment and intensity (see Section **7.2.6**);

5. Vital signs [blood pressure, heart rate, respiratory rate, oral body temperature]: Change from Baseline to each of the scheduled on-treatment and post-treatment assessment time points;

6. Body weight: Change from Baseline to each of the scheduled on-treatment and post-treatment assessment time points;

7. Clinical laboratory values (hematology and biochemistry): Change from Baseline to each of the scheduled on-treatment and post-treatment assessment time points;

8. Lipid profile (triglycerides, total cholesterol, low density lipoprotein [LDL], high density lipoprotein [HDL]): Change from Baseline to each of the scheduled on-treatment and post-treatment assessment time points;

9. Urinalysis by dipstick and microscopic analysis: Change from Baseline to each of the scheduled on-treatment and post-treatment assessment time points;
10. 12-lead electrocardiogram (ECG): Change from Baseline to each of the scheduled on-treatment and post-treatment assessment time points;
11. Hand x-ray: bone age at Month 24;
12. Spine x-ray: Spine fractures at Month 24.

Data for the following additional safety outcomes will be listed only:

1. Physical examination findings at Pretreatment, Month 12, and Month 24.

Section Changed:

2.2.2 Clinical Efficacy Endpoints

Original Text:

2.2.2.1 Primary Clinical Efficacy Endpoint

1. Time to Stand Test (TTSTAND) velocity (rise/second): Comparison with a historical natural history (untreated) control group for change from Baseline to Month 12.

2.2.2.2 Secondary Efficacy Endpoints

1. Time to Stand Test (TTSTAND) velocity (rise/second): Change from Baseline to each of the scheduled on-treatment and post-treatment assessment time points;

Revised Text:

2.2.2.1 Primary Clinical Efficacy Endpoint

1. Time to Stand Test (TTSTAND) velocity (rise/second): Comparison with a historical natural history (untreated) control group for change from Baseline to Month 24.

2.2.2.2 Secondary Efficacy Endpoints

1. Time to Stand Test (TTSTAND) velocity (rise/second): Comparison with a historical natural history (untreated) control group for change from Baseline to Month 12;
2. Time to Stand Test (TTSTAND): Change from Baseline to each of the scheduled on-treatment and post-treatment assessment time points;

Section Changed:

2.2.2.3 Exploratory Efficacy Endpoint

Original Text:

2.2.2.3 Exploratory Efficacy Endpoint

1. To investigate the effects of vamorolone administered orally at daily doses up to 6.0 mg/kg over a 24-month Treatment Period on Quality of Life measures (PODCI).

Revised Text:

[text deleted]

Section Changed:

2.2.3 Pharmacodynamic Endpoints

Original Text:

Concentrations of serum and/or salivary PD biomarkers of adrenal suppression (morning salivary cortisol), insulin resistance (fasting insulin and glucose), and bone turnover (osteocalcin, serum carboxy-terminal telopeptide of type I collagen [CTX1]).

Revised Text:

Concentrations of serum PD biomarkers of adrenal suppression, insulin resistance, and bone turnover.

Section Changed:

2.2.4 Exploratory Endpoints

Original Text:

2.2.4 Exploratory Endpoints

1. Levels of an extended panel of PD efficacy and safety biomarkers using somaSCAN aptamer arrays, steroid hormone profiling, and other biomarker methods.

Revised Text:

2.2.4 Exploratory Endpoints

1. Pediatric Outcomes Data Collection Instrument (PODCI): Change from baseline to each of the scheduled on-treatment and post-treatment assessment time points;
2. Levels of additional exploratory PD biomarkers; and
3. DNA testing for established genetic modifiers of DMD.

Section Changed:

3.1 Overall Study Design
Paragraphs 5 and 7

Original Text:

Subjects in the VBP-LTE will begin dosing at the same vamorolone dose level they received at the time they completed the Week 24 Final Visit in the VBP15-003 Phase IIa extension core study. Subjects, once they have been on their initial dose in the VBP15-LTE study for at least one month, may have their dose escalated at the discretion of the Study Chair and Medical Monitor once that dose has been determined to be safe in the VBP15-002 Phase IIa Study, and no safety issues have emerged at that dose in the VBP15-003 Phase IIa extension study. Subjects will continue to receive vamorolone at one of the assigned dose levels for the duration of the 24-month Treatment Period, unless ongoing safety data indicate the dose level should be de-escalated.

Revised Text:

Subjects in the VBP-LTE will begin dosing at the same vamorolone dose level they received at the time they completed the Week 24 Final Visit in the VBP15-003 Phase IIa extension core study. Subjects, once they have been on their initial dose in the VBP15-LTE study for at least one month, may have their dose escalated at the discretion of the Study Chair and Medical Monitor once that dose has been determined to be safe in the VBP15-002 Phase IIa Study, and no safety issues have emerged at that dose in the

VBP15-003 Phase IIa extension study. Subjects may continue to have their dose of study medication escalated incrementally at no shorter than one-month intervals to the maximum dose tested and assessed as safe in the VBP15-002 and VBP15-003 studies, 6.0 mg/kg/day. Subjects will continue to receive vamorolone at one of the assigned dose levels for the duration of the 24-month Treatment Period, unless ongoing safety data indicate the dose level should be de-escalated.

Original Text:

In the event any clinical observation suggests an intolerability for an individual subject to the study medication, in the opinion of the Investigator, the subject's dose level may be decreased to the next lower dose level (e.g., a subject taking 6.0 mg/kg/day decreased to 2.0 mg/kg/day) and maintained at that lower dose level throughout the duration of the Treatment Period. In the event the next lower dose level is also not tolerated and is considered a safety risk to the subject, in the opinion of the Investigator, Study Chair, and Medical Monitor, the subject should be withdrawn from the study. Details of dose interruption, de-escalation, and discontinuation are presented in **5.6**.

Revised Text:

In the event any clinical observation suggests an intolerability for an individual subject to the study medication, in the opinion of the Investigator, the subject's dose level may be decreased to the next lower dose level and maintained at that lower dose level throughout the duration of the Treatment Period, with the caveat stated below for subjects de-escalating from 6.0 mg/kg/day to 2.0 mg/kg/day. In the event the next lower dose level is also not tolerated and is considered a safety risk to the subject, in the opinion of the Investigator, Study Chair, and Medical Monitor, the subject should be withdrawn from the study. Subjects whose dose is decreased from 6.0 mg/kg/day to 2.0 mg/kg/day may have their dose subsequently increased to 4.0 mg/kg/day if they have been taking the 2.0 mg/kg/day dose for at least one month and, in the opinion of the Investigator, balancing efficacy with safety concerns, they could benefit from an intermediate higher dose. Details of dose interruption, de-escalation, and discontinuation are presented in Section **5.6**.

Section Changed:

5.4 Rationale for Dose Selection
Paragraph 3

Original Text:

Based on the Phase I PD biomarker safety data presented in **1.3**, safety signals reflective of insulin resistance, bone turnover, immune suppression, or adrenal suppression are not anticipated at any of the planned long-term extension dose levels.

Revised Text:

Based on the safety data presented in Section **1.3.3**, the doses initially selected for this study are appropriate for evaluation in a long-term extension study.

Section Changed:

5.6 Study Drug Dose Interruption, De-escalation, or Discontinuation
Paragraph 1

Original Text:

Administration of study drug to individual subjects should be interrupted, and the case discussed with the Study Chair and Medical Monitor within 24 hours, in the event any clinical observation suggests an intolerability of an individual subject to the study medication, in the opinion of the Investigator. In such an event, the subject's dose level may be decreased to the next lower dose level (e.g., a subject taking 6.0 mg/kg/day decreased to 2.0 mg/kg/day) and maintained at that lower dose level throughout the duration of the study. In the event the next lower dose level is also not tolerated and is considered a safety risk to the subject, in the opinion of the Investigator, Study Chair and Medical Monitor, the subject should be withdrawn from the study.

Revised Text:

Administration of study drug to individual subjects should be interrupted, and the case discussed with the Study Chair and Medical Monitor within 24 hours, in the event any clinical observation suggests an intolerability to the study medication and a safety risk to an individual subject, in the opinion of the Investigator. In such an event, or in situations where the intolerability is not considered an immediate safety risk to the subject, the subject's dose level may be decreased to the next lower dose level and maintained at that

lower dose level throughout the duration of the study, with the caveat stated below for subjects de-escalating from 6.0 mg/kg/day to 2.0 mg/kg/day. In the event the next lower dose level is also not tolerated and is considered a safety risk to the subject, in the opinion of the Investigator, Study Chair and Medical Monitor, the subject should be withdrawn from the study.

Subjects whose dose is decreased from 6.0 mg/kg/day to 2.0 kg/kg/day may have their dose subsequently increased to 4.0 mg/kg/day if they have been taking the 2.0 mg/kg/day dose for at least one month and, in the opinion of the Investigator, balancing efficacy with safety concerns, they could benefit from an intermediate higher dose.

Section Changed:

5.7.3 Prohibited Therapies
Paragraph 1, Bullet #5

Original Text:

- Live attenuated vaccines (use must be avoided for the duration of participation in the study);
- Any investigational medications other than vamorolone (use must be discontinued at least 3 months prior to the first dose of study medication).

Revised Text:

- Live attenuated vaccines (use must be avoided for the duration of participation in the study);
- Medications indicated for the treatment of DMD, including Exondys 51 and Translarna (use must be avoided for the duration of participation in the study);
- Any investigational medications other than vamorolone (use must be discontinued at least 3 months prior to the first dose of study medication).

Section Changed:

6.1 Time and Events Schedule
Table 8

Original Text:

Table 8 Schedule of Study Activities

	Pretreatment Period		Treatment Period									Dose-tapering Period
	Baseline											
	Day		Month									
**Study Day or Week/Visit**	-1[a]	1[b]	1 (±7d)	3 (±7d)	6 (±7d)	9 (±7d)	12 (±7d)	15 (±7d)	18 (±7d)	21 (±7d)	24[c] (±7d)	24-25[d] (±7d)
Inclusion/Exclusion Criteria	X											
Informed Consent	X[e]											
Enrollment	X											
Interim Medical History	X[f]											
Medication History	X[g]											
Physical Examination	X[h]						X				X	
Height	X						X				X	
Weight	X			X	X	X	X	X	X	X	X	
Vital Signs[i]	X		X	X	X	X	X	X	X	X	X	X
Blood for Clinical Labs[j]	X[h]				X		X		X		X	X
Urinalysis[k]	X[h]				X		X		X		X	
Blood for Serum PD Biomarker Panel[l]	X[h]				X		X		X		X	X
Fasting morning blood for insulin, glucose, cortisol[m]						X				X		
12-lead ECG[n]	X[h]						X				X	
Dispense Study Medication	X			X	X	X[o]	X	X	X	X[o]	X	
Return Study Medication/ Compliance Monitoring				X	X	X	X	X	X	X	X	X
Study Medication Dosing		X	→	→	→	→	→	→	→	→	→ X	
Vamorolone dose tapering[p,q]												X
Time to Stand Test (TTSTAND)	X[h]						X				X	
Time to Climb Test (TTCLIMB)	X[h]						X				X	
Time to Run/Walk Test (TTRW)	X[h]						X				X	
NSAA[r]	X[h]						X				X	
Quantitative Muscle Testing (QMT)	X[h]						X				X	
Six-minute Walk Test (6MWT)	X[h]						X				X	
Pediatric Outcomes (PODCI) Data Collection Instrument	X						X				X	
Dispense Subject Diaries[s]	X			X	X	X	X	X	X	X	X	
Return Subject Diaries			X	X	X	X	X	X	X	X	X	X
AE/SAE Recording[t]	X →	→	→	→	→	→	→	→	→	→	→ X	X
Concomitant Medications		X	→	→	→	→	→	→	→	→	→ X	X
Discharge from Study											X[u]	X[v]

Revised Text:

Table 8 Schedule of Study Activities

	Pretreatment Period		Treatment Period									Dose-tapering Period
	Baseline											
	Day		Month									
**Study Day or Month/Visit**	**-1[a]**	**1[b]**	**1 (±7d)**	**3 (±7d)**	**6 (±7d)**	**9 (±7d)**	**12 (±7d)**	**15 (±7d)**	**18 (±7d)**	**21 (±7d)**	**24[c] (±7d)**	**24-25[d] (±7d)**
Inclusion/Exclusion Criteria	X											
Informed Consent	X[e]											
Enrollment	X											
Interim Medical History	X[f]											
Medication History	X[g]											
Physical Examination	X[h]						X				X	
Height	X						X				X	
Weight	X			X	X	X	X	X	X	X	X	
Vital Signs[i]	X		X	X	X	X	X	X	X	X	X	X
Blood for Clinical Labs[j]	X[h]				X		X		X		X	X
Urinalysis[k]	X[h]				X		X		X		X	
Blood for Serum PD Biomarker Panel[l]	X[h]				X		X		X		X	X
Fasting morning blood for insulin, glucose, cortisol[m]						X				X		
Blood for DNA Testing											X	
12-lead ECG[n]	X[h]						X				X	
Dispense Study Medication	X			X	X	X[o]	X	X	X	X[o]	X	
Return Study Medication/ Compliance Monitoring				X	X	X	X	X	X	X	X	X
Study Medication Dosing		X	→	→	→	→	→	→	→	→	X	
Vamorolone dose tapering[p,q]												X
Time to Stand Test (TTSTAND)	X[h]						X				X	
Time to Climb Test (TTCLIMB)	X[h]						X				X	
Time to Run/Walk Test (TTRW)	X[h]						X				X	
NSAA[r]	X[h]						X				X	
Quantitative Muscle Testing (QMT)	X[h]						X				X	
Six-minute Walk Test (6MWT)	X[h]						X				X	
Pediatric Outcomes Data Collection Instrument (PODCI)	X[h]						X				X	
Spine x-ray											X	
Hand x-ray											X	
Dispense Subject Diaries[s]	X			X	X	X	X	X	X	X	X	
Return Subject Diaries			X	X	X	X	X	X	X	X	X	X
AE/SAE Recording[t]	X →	→	→	→	→	→	→	→	→	→	X	X
Concomitant Medications		X	→	→	→	→	→	→	→	→	X	X
Discharge from Study											X[u]	X[v]

Table 8, Footnote l

Original Text:

l. Blood collected for pharmacodynamic safety biomarkers (osteocalcin, CTX1), and exploratory safety and efficacy biomarkers (somaSCAN, steroid hormones, others).

Revised Text:

l. Blood collected for pharmacodynamic safety biomarkers and exploratory safety and efficacy biomarkers.

Section Changed:

6.3.1 Baseline Period (Day -1)
Paragraph 6, Bullet #11

Original Text:

- Blood samples for PD biomarkers including osteocalcin, and CTX1. Blood will also be collected and stored for exploratory somaSCAN aptamer panel, steroid hormone profiling, and other studies. (Not to be repeated if Baseline Day -1 is ≤ 28 days after the VBP15-003 Week 24 Final Visit assessment date.) (see **7.3.7**)

Revised Text:

- Blood samples for PD biomarkers. Blood will also be collected and stored for exploratory PD biomarkers. (Not to be repeated if Baseline Day -1 is ≤ 28 days after the VBP15-003 Week 24 Final Visit assessment date.) (see **7.3.7**)

Section Changed:

6.3.3 Treatment Period Months 1-24
Paragraph 4

Original Text:

Clinical efficacy assessments (TTSTAND, TTRW, TTCLIMB, NSAA, 6MWT, and QMT) and the Pediatric Outcomes Data Collection Instrument (PODCI) will be conducted at 12 and 24 months. Weight will be recorded every visit from 3-24 months and height will be measured at 12 and 24 months. Vital signs will be recorded at each study visit. A physical examination will be performed at Months 12 and 24. A 12-lead ECG will be recorded at Months 12 and 24. Blood and urine samples for clinical laboratory tests, and blood for the serum PD biomarker panel including somaSCAN,

steroid hormone profiling, and others will be collected at 6, 12, 18, and 24 months (see **Table 8**). Adverse events, including SAEs, and concomitant medications will be assessed at each study visit and recorded throughout the study.

Revised Text:

Clinical efficacy assessments (TTSTAND, TTRW, TTCLIMB, NSAA, 6MWT, and QMT) and the Pediatric Outcomes Data Collection Instrument (PODCI) will be conducted at 12 and 24 months. Weight will be recorded every visit from 3-24 months and height will be measured at 12 and 24 months. Vital signs will be recorded at each study visit. A physical examination will be performed at Months 12 and 24. A 12-lead ECG will be recorded at Months 12 and 24. Blood and urine samples for clinical laboratory tests, and blood for PD biomarkers will be collected at 6, 12, 18, and 24 months (see **Table 8**). Blood will be collected at Month 24 for DNA testing for established genetic modifiers of DMD. Spine x-ray and hand x-ray will be performed at Month 24. Adverse events, including SAEs, and concomitant medications will be assessed at each study visit and recorded throughout the study.

Section Changed:

6.3.4 Dose-Tapering Period Months 24-25
Paragraphs 2 and 3

Original Text:

The subject's weight recorded at the Month 24 Visit will be used to calculate dose and dose volume for all dose de-escalations during the Dose-tapering Period (see **Appendix 14.1**).

Table 9 Vamorolone Dose Tapering

Treatment Period Vamorolone Dose Level at Month 24 Final Visit	Dose Level for First Week Following the Month 24 Final Visit	Dose Level for Second Week Following the Month 24 Final Visit	Dose Level for Third Week Following the Month 24 Final Visit	Dose Level for Fourth Week Following the Month 24 Final Visit	Dose Level for Fifth Week Following the Month 24 Final Visit
0.25 mg/kg/day	0.125 mg/kg/day	0 mg/kg/day			
0.75 mg/kg/day	0.375 mg/kg/day	0.125 mg/kg/day	0 mg/kg/day		
2.0 mg/kg/day	1 mg/kg/day	0.5 mg/kg/day	0.25 mg/kg/day	0 mg/kg/day	
6.0 mg/kg/day	3 mg/kg/day	1.5 mg/kg/day	0.75 mg/kg/day	0.25 mg/kg/day	0 mg/kg/day

All subjects participating in the Dose-tapering Period will return to the study site for final study assessments approximately one week after the final dose de-escalation (i.e., approximately one week after the subject has received his last dose of vamorolone). The duration of the Dose-tapering Period will therefore vary depending upon the vamorolone dose received by the subject at completion of the Month 24 Visit of the Treatment Period, from a total duration of two weeks (for subjects receiving 0.25 mg/kg/day vamorolone; final study visit two weeks following the Month 24 Final Visit) to five weeks (for subjects receiving vamorolone 6.0 mg/kg/day; final study visit five weeks following the Month 24 Final Visit).

Revised Text:

The subject's weight recorded at the Month 24 Visit will be used to calculate dose and dose volume for all dose de-escalations during the Dose-tapering Period (see Appendix 14.1).

Table 9 Vamorolone Dose Tapering

Treatment Period Vamorolone Dose Level at Month 24 Final Visit	Dose Level for First Week Following the Month 24 Final Visit	Dose Level for Second Week Following the Month 24 Final Visit	Dose Level for Third Week Following the Month 24 Final Visit	Dose Level for Fourth Week Following the Month 24 Final Visit	Dose Level for Fifth Week Following the Month 24 Final Visit
0.25 mg/kg/day	0.125 mg/kg/day	0 mg/kg/day			
0.75 mg/kg/day	0.375 mg/kg/day	0.125 mg/kg/day	0 mg/kg/day		
2.0 mg/kg/day	1 mg/kg/day	0.5 mg/kg/day	0.25 mg/kg/day	0 mg/kg/day	
4.0 mg/kg/day	2 mg/kg/day	1 mg/kg/day	0.5 mg/kg/day	0.25 mg/kg/day	0 mg/kg/day
6.0 mg/kg/day	3 mg/kg/day	1.5 mg/kg/day	0.75 mg/kg/day	0.25 mg/kg/day	0 mg/kg/day

All subjects participating in the Dose-tapering Period will return to the study site for final study assessments approximately one week after the final dose de-escalation (i.e., approximately one week after the subject has received his last dose of vamorolone). The duration of the Dose-tapering Period will therefore vary depending upon the vamorolone dose received by the subject at completion of the Month 24 Visit of the Treatment Period, from a total duration of two weeks (for subjects receiving 0.25 mg/kg/day vamorolone; final study visit two weeks following the Month 24 Final Visit) to five weeks (for

subjects receiving vamorolone 4.0 mg/kg/day or 6.0 mg/kg/day; final study visit five weeks following the Month 24 Final Visit).

Section Changed:

7.1 Demographic Assessments

Original Text:

Demographic information (birth date, race, and ethnicity) collected during the VBP15-002 study will be used for this study; collection of demographic data will not be repeated during this study.

Revised Text:

Demographic information (birth date, race, and ethnicity) collected during the VBP15-002 study will be used for this study; collection of demographic data will not be repeated during this study.

7.1.1 Genetic Modifiers of DMD

Approximately 8.5 mL of blood will be collected at the Month 24 Visit for DNA testing to determine if established genetic modifiers of DMD (gene polymorphisms associated with disease severity or response to glucocorticoid treatment) are similarly associated with vamorolone-treated DMD patients (baseline disease severity or response to vamorolone treatment).

DNA testing will be performed by a certified central laboratory.

The procedures for the collection, handling, and shipping of blood samples for DNA testing will be specified in the Laboratory Manual(s) provided to the clinical center. Results will be compared to historical control data for glucocorticoid responders and non-responders.

Section Changed:

7.2 Safety and Tolerability Assessments
Text inserted following Section 7.2.6

Original Text:

[none]

Revised Text:

7.2.7 Bone Age (Hand X-ray)

Bone age will be assessed by hand x-ray at the Month 24 Visit. Hand radiography will be performed as described in the Manual of Operations. Hand radiographs will be analyzed centrally by a certified radiologist through the imaging coordinating center in Ottawa, Canada.

7.2.8 Spine X-ray

Data on bone health will also be collected by lateral spine x-ray (T4-L5) at the Month 24 Visit. Lateral spine x-rays will be analyzed centrally by two certified pediatric radiologists at Children's Hospital of Ottawa in Ottawa, Canada, who are blinded to the results of one another; a third radiologist will resolve any discrepancies arising from the first two readings. Quantification of any vertebral fractures detected will be performed.

Fractures will be recorded as AEs.

Section Changed:

7.3.1 Time to Stand Test (TTSTAND)
Paragraph 2

Original Text:

The TTSTAND measures the speed (m/s; velocity) with which the child can stand to an erect position from supine (floor), and is administered as part of the NSAA (see **7.3.4**). Complete instructions for administering and scoring the TTSTAND are given in the study manual to be supplied to the sites prior to study start.

Revised text:

The TTSTAND measures the speed (rise/second; velocity) with which the child can stand to an erect position from supine (floor), and is administered as part of the NSAA (see Section **7.3.4**). Complete instructions for administering and scoring the TTSTAND are given in the study manual to be supplied to the sites prior to study start.

Section Changed:

7.3.7 Pharmacodynamic Biomarker Panel

Paragraph 2, Table 12

Original Text:

The biomarker analysis includes osteocalcin and CTX1. Blood will also be collected at each of these time points and stored for exploratory efficacy and safety pharmacodynamic biomarkers using somaSCAN aptamer panels, steroid hormone profiling, and others.

Revised Text:

Blood will also be collected at each of these time points and stored for exploratory efficacy and safety pharmacodynamic biomarkers.

Original Text:

Table 12 Pharmacodynamic Biomarkers

**Adrenal Suppression**
Morning Cortisol
**Insulin Resistance**
Fasting Glucose
Fasting Insulin
**Bone Turnover**
Osteocalcin
CTX1
**Exploratory Biomarkers**
somaSCAN aptamer profiling, steroid hormone profiling, and others

Revised Text:

Table 12 Pharmacodynamic Biomarkers

Adrenal Suppression
Morning Cortisol
ACTH
17- hydroxyprogesterone
Testosterone
Corticosterone
11- deoxycortisol
**Insulin Resistance**
Fasting Glucose
Fasting Insulin
HbA1c
**Bone Turnover**
Osteocalcin
CTX1
P1NP
**Exploratory Biomarkers**
Other PD biomarkers

Section Changed:

7.4 Total Blood Volume Required

Original Text:

7.4 Total Blood Volume Required

The number and volume of blood samples and total volume of blood to be collected from each subject throughout the duration of the 24-month study are summarized in **Table 13**. A total of approximately 96-137 mL of blood will be collected from each subject over the course of the up to 24-month study.

Table 13 Blood Sample Number and Volume

Test	Total mL of Blood								
	**Day -1[a]**	**Month 6**	**Month 9**	**Month 12**	**Month 18**	**Month 21**	**Month 24**	**End of Dose-tapering Period[b]**	**Total Volume**
Clinical Safety Labs[c]	4.5	4.5		4.5	4.5		4.5	4.5	18-27
PD Biomarker Panel	15[d]	17[e]		17[e]	17[e]		17[e]	17[e]	68-100
PD Insulin/Glucose/ Cortisol			5[f]			5[f]			10
Total Volume by Visit (mL)	19.5	21.5	5	21.5	21.5	5	21.5	21.5	96-137
**Total Volume: approximately 96-137**									

[a] Day -1 blood draws for clinical labs and PD biomarkers will not be repeated for subjects whose Day -1 Visit is ≤ 28 days following VBP15-003 Week 24 Final Visit and who had blood drawn for clinical labs and PD biomarkers at that Visit.
[b] Only subjects who participate in the Dose-tapering Period will have bloods drawn.
[c] Hematology, Chemistry, Lipids.
[d] cortisol, P1NP, osteocalcin, 17- hydroxyprogesterone, testosterone, corticosterone, 11-deoxycortisol, CTX, ACTH, SomaScan, and proteomics testing.
[e] cortisol, P1NP, osteocalcin, 17- hydroxyprogesterone, testosterone, corticosterone, 11-deoxycortisol, CTX, ACTH, HbA1c, SomaScan, and proteomics testing.
[f] Subjects must have fasted ≥ 6 hours prior to blood draws. Blood will be drawn prior to administration of the dose of study medication.

Revised Text:

7.4 Total Blood Volume Required

The number and volume of blood samples and total volume of blood to be collected from each subject throughout the duration of the 24-month study are summarized in **Table 13**. A total of approximately 104.5-145.5 mL of blood will be collected from each subject over the course of the up to 24-month study.

Table 13 Blood Sample Number and Volume

Test	Total mL of Blood								
	Day -1[a]	Month 6	Month 9	Month 12	Month 18	Month 21	Month 24	End of Dose-tapering Period[b]	Total Volume
Clinical Safety Labs[c]	4.5	4.5		4.5	4.5		4.5	4.5	18-27
PD Biomarker Panel	15[d]	17[e]		17[e]	17[e]		17[e]	17[e]	68-100
PD Insulin/Glucose/ Cortisol			5[f]			5[f]			10
DNA Testing							8.5		8.5
Total Volume by Visit (mL)	19.5	21.5	5	21.5	21.5	5	30	21.5	104.5-145.5
**Total Volume: approximately 104.5-145.5 mL**									

[a] Day -1 blood draws for clinical labs and PD biomarkers will not be repeated for subjects whose Day -1 Visit is ≤ 28 days following VBP15-003 Week 24 Final Visit and who had blood drawn for clinical labs and PD biomarkers at that Visit.
[b] Only subjects who participate in the Dose-tapering Period will have bloods drawn.
[c] Hematology, Chemistry, Lipids.
[d] cortisol, P1NP, osteocalcin, 17- hydroxyprogesterone, testosterone, corticosterone, 11-deoxycortisol, CTX, ACTH, and exploratory PD biomarkers.
[e] cortisol, P1NP, osteocalcin, 17- hydroxyprogesterone, testosterone, corticosterone, 11-deoxycortisol, CTX, ACTH, HbA1c, and exploratory PD biomarkers.
[f] Subjects must have fasted ≥ 6 hours prior to blood draws. Blood will be drawn prior to administration of the dose of study medication.

Section Changed:

9 Statistical Methods and Planned Analyses
Paragraph 2

Original Text:

This is an open-label extension study with no placebo control. Historical control data are available for the same age range (4-10 years), at largely the same study sites, with the same outcome measures. Untreated natural history control population data is from the ongoing CINRG DNHS study of ~400 DMD boys.[43,44,45,46] Prednisone-treated historical control data is from a clinical trial of prednisone carried out by the CINRG group.[47] The vamorolone VBP15-LTE Phase IIa extension trial is carried out with daily dosing. The numbers of subjects needed per group to detect significant differences in the glucocorticoid effect on efficacy for three gross motor milestone outcomes (TTSTAND, TTRW, TTCLIMB) for observed changes and for conservative change estimates (100%, 80% or 60% of that observed with daily glucocorticoids), respectively, are shown in

Table 14. The numbers of subjects needed per group to detect significant differences in the glucocorticoid effect on safety for BMI z-score for observed changes and for conservative change estimates, respectively, are also shown in **Table 14.**

Vamorolone is not anticipated to show an increase in BMI.

Table 14 6-month and 12-month Changes in Outcomes in 4-<8 year-old DMD Boys

Outcome	Time (months)	Untreated – Natural history study		Treated – Prednisone trial		N needed per group to detect a significant difference – Using observed changes	N needed per group to detect a significant difference – Using conservative change estimates ^	N needed per group to detect a significant difference – Using conservative change estimates ^^
		N	Mean ± SD	N	Mean ± SD			
BMI (Z-score)	6	40	-0.14 ± 0.78	13	0.54 ± 0.66	12	15	
	12	26	-0.25 ± 0.83	13	0.63 ± 0.45	7	6	
Time to run/walk 10 m velocity (m/s)	6	39	0.02 ± 0.31	13	0.35 ± 0.32	12	19	28
	12	26	0.01 ± 0.25	12	0.42 ± 0.23	5	7	11
Time to climb 4 stairs velocity (climb/s)	6	40	0.01 ± 0.07	13	0.11 ± 0.08	7	11	17
	12	26	0.01 ± 0.06	12	0.16 ± 0.10	5	7	10
Time to stand from supine velocity (rise/s)	6	38	0.004 ± 0.07	12	0.10 ± 0.10	10	16	23
	12	26	-0.01 ± 0.06	11	0.11 ± 0.09	5	8	9

Notes: a) All patients are 4-<8 years of age at study start.
b) Data from untreated subjects in the CINRG DMD Natural history study[43,44,45,46] data from treated subjects in the CINRG Prednisone trial.[47]
c) Compares all only daily dose prednisone-treated patients to untreated patients.
d) All calculations assume a repeated measures ANOVA model with one pre- and one post-measurement. Correlation between repeated measures used as follows: 0.75 for height changes, 0.70 for 6 month BMI changes, and 0.65 for 12 month BMI changes, and 0.575 for 6 and 12 month velocity changes. Power = 0.80 and alpha = 0.025 for all calculations to account for multiple group comparisons (i.e. two vamorolone doses, each compared to prednisone for safety and placebo for efficacy).
^ Conservative change estimates were defined as follows: For BMI, the expected change in vamorolone was set to 0.0 rather than the observed decrease. For timed tests, the expected change in vamorolone was set to 80% of the observed change in the prednisone group.
^^ Conservative change estimates were defined as follows: For timed tests, the expected change in vamorolone was set to 60% of the observed change in the prednisone group (with SD at 80%).

Revised Text:

This is an open-label extension study with no placebo control. Historical control data are available for the same age range (4-10 years), at largely the same study sites, with the same outcome measures. Untreated natural history control population data is from the ongoing CINRG DNHS study of ~400 DMD boys.[44,45,46,47] Prednisone-treated and deflazacort-treated historical control data are from a clinical trial of daily dosing of prednisone and deflazacort.[48] The vamorolone VBP15-LTE Phase IIa extension trial is carried out with daily dosing.

Section Changed:

9.2 Analysis Populations

Original Text:

All analyses will be based on the actual treatment each subject received. Four populations will be defined for data analysis.

Revised Text:

Four populations will be defined for data analysis.

Section Changed:

9.2 Analysis Populations
9.2.2, 9.2.3, 9.2.4

Original Text:

9.2.2 Full Analysis Set (FAS)

All subjects who receive at least one dose of vamorolone study medication in the VBP15-LTE extension study and have at least one post-baseline assessment will be included in the FAS. The FAS is the primary analysis population for efficacy and PD assessments. This is the mITT population, with the additional requirement to have at least one post-baseline assessment. Subjects who receive at least one dose of vamorolone but never have post-baseline assessments will be excluded.

9.2.3 Control Population DNHS Study

The control population from the DNHS study will include all study subjects who were observed as part of the study in ages ≥ 4 years and <10 years of age at a start of an interval of observation and observed for at least one year. Further, the subjects need to have had at least two visits in a time interval of no more than 15 months (e.g., Month 24 and Month 36 of observation for a subject who entered at age 2 or 3). The subject should have been able to walk independently without assistive devices at the start of the interval and should have been able to complete the TTSTAND. The subject should not have had any history of disease or impairment or medications that would have made him ineligible to receive the vamorolone intervention as defined by the Phase IIa extension study

VBP15-LTE exclusion criteria. The subject should have been glucocorticoid-naïve for the entire interval considered in the control population for this study and should not have begun any investigational treatment for the interval considered for the control comparison. Finally, the control intervals to be considered should have the study outcomes of TTSTAND, TTCLIMB, TTRW, NSAA, 6MWT and QMT measured. It is acceptable if the participant had progressive disease and could not perform a measurement at a later point in the interval; this will be a velocity zero.

9.2.4 Control Population Prednisone Study

The control population from the prednisone study will include all subjects who were younger than 10 years old at entry and who were randomized to the daily prednisone arm.

Revised Text:

9.2.2 Full Analysis Set (FAS)

All subjects who receive at least one dose of vamorolone study medication in the VBP15-LTE extension study and have at least one post-baseline assessment will be included in the FAS. The FAS is the primary analysis population for efficacy and PD assessments. The FAS is the mITT population, with the additional requirement of having at least one post-baseline assessment. Subjects who receive at least one dose of vamorolone but never have post-baseline assessments will be excluded.

9.2.3 Control Population DNHS Study

The control population from the CINRG Duchenne Natural History Study will include all subjects who were observed as part of the study in ages ≥ 4 years and <7 years of age at a start of an interval of observation; observed for at least two years with TTSTAND, TTCLIMB, TTRW, NSAA, 6MWT and QMT measured; remained glucocorticoid-naive during the entire observation period; and were able to walk independently without assistive devices, able to complete the TTSTAND; and lacked any history of disease, impairment, or medications that would have made them ineligible to receive the vamorolone intervention as defined by the VBP15-LTE exclusion criteria at the start of the interval.

9.2.4 Control Population FOR DMD Study

The control population from the FOR DMD study will include all subjects who were 4-7 years old at entry and who were randomized to the daily prednisone or daily deflazacort arm.[48]

Section changed:

9.6 Statistical Analyses
9.6.1, 9.6.2, 9.6.3, 9.6.4

Original Text:

9.6.1 General Considerations

Statistical analyses will be performed using SAS®.

All measurements will be analyzed based upon the type of distribution, and descriptive statistics will be presented by treatment group and assessment time point, as appropriate. Descriptive statistics for continuous variables (number [N], mean, median, standard deviation [SD], minimum, and maximum), descriptive statistics for categorical variables (N and percentage), and individual subject profiles will be presented, as appropriate.

Baseline measurement is defined as the last non-missing value prior to the first dose of study drug in the VBP15-LTE extension study.

9.6.2 Subject Disposition, Demographics, and Baseline Characteristics

Subject disposition will be summarized by analysis population. The number of subjects enrolled, the number in each population, and the reason for discontinuation from the study will be summarized.

Subject demographics (e.g., age, race, and ethnicity) and baseline characteristics (e.g., height, weight, and months/years since DMD diagnosis) will be summarized descriptively by analysis population. Listings of individual subject data sorted by dose group will be reviewed for any differences among the dose groups.

9.6.3 Safety Analyses

All subjects who received at least one dose of vamorolone (Safety Population) will be included in the safety analyses. In general, descriptive statistics for each safety endpoint will be presented by dose level, while individual subject listings of safety endpoints,

sorted by dose group, will be reviewed for any evidence of dose-related differences or trends in the safety profile of vamorolone.

All safety data will be listed.

Baseline measurement is defined as the non-missing value of the core study VBP15-003 final visit or last non-missing value prior to first administration of study drug in the VBP15-LTE extension study.

All evaluations of clinical safety will be listed with descriptive statistics presented by treatment group. The primary safety variable will be BMI z-score and will be assessed using the same type of statistical models used for efficacy. Additional secondary safety data will include height z-score, blood pressure, and ECG results. As no change in blood pressure or cardiac results is expected, these data will be analyzed using an analysis of covariance (ANCOVA) approach which includes the baseline value for each measurement as a covariate. Changes in height z-scores, for which an observed change is expected, will be modelled using a linear mixed effects model. Continuous, quantitative laboratory values will be analyzed similar to BMI z-scores. Categorized laboratory values and presence or absence of AEs will be compared using an exact chi-square test.

Adverse events will be coded using the Medical Dictionary for Regulatory Activities (MedDRA). The incidence of AEs will be summarized overall and by dose level, SOC and preferred term; dose level, SOC, preferred term, and intensity (CTCAE grade); and dose level, SOC, preferred term, and relationship to study drug. Additional AE analyses will be at the subject level: the number of subjects who had any AE, the distribution of number of AEs per subject within a dose level, worst intensity in a subject within a dose level, highest level of relationship to study treatment for each subject within a dose level.

Additional hypotheses of safety will include similar linear modelling with the secondary and exploratory outcomes.

Physical examination results will be listed only.

9.6.4 Clinical Efficacy and Pharmacodynamic Analyses

The evaluations of clinical efficacy and PD will be performed using the FAS Population.

The primary efficacy outcome is the time to stand from supine velocity (TTSTAND). Secondary efficacy outcomes are the NSAA assessment, time to climb four stairs (TTCLIMB), time to run/walk 10 meters (TTRW), Quantitative Muscle Testing (QMT), and the distance walked in 6 minutes (6MWT). The primary outcome will be compared between vamorolone and historical untreated controls using a mixed-effects linear model with treatment group. Age at study entry will be included as a covariate. The initial model will combine vamorolone doses and a subsequent secondary model will look at each dose in comparison to untreated natural history controls. This will allow the testing of whether vamorolone in general and vamorolone at individual doses have significant effects on the slope of the velocity. Additional hypotheses of efficacy will include similar linear modelling with the secondary outcomes. They will also include differing comparison groups including untreated natural history controls.

Serum PD biomarkers of adrenal axis suppression, insulin resistance, and bone turnover will be assessed. Longitudinal analysis of PD biomarkers will be studied as a function of treatment, with comparison to prednisone-treated and untreated historical controls, as appropriate.

Individual clinical efficacy and PD data will be listed.

Revised Text:

9.6.1 General Considerations

Statistical analyses will be performed using SAS®.

All measurements will be analyzed based upon the type of distribution, and descriptive statistics will be presented by treatment group and assessment time point, as appropriate. Descriptive statistics for continuous variables (number [N], mean, median, standard deviation [SD], minimum, and maximum), descriptive statistics for categorical variables (N and percentage), and individual subject profiles will be presented, as appropriate.

All statistical tests will be performed at the 0.05 level. Paired t-tests will be utilized for select variables. When available, baseline from the VBP15-002 will be utilized when calculating change from baseline.

9.6.2 Subject Disposition, Demographics, and Baseline Characteristics

Subject disposition will be summarized by analysis population. The number of subjects enrolled, the number in each population, and the reason for discontinuation from the study will be summarized.

Subject demographics (e.g., age, race, and ethnicity) and baseline characteristics (e.g., height, weight, and months/years since DMD diagnosis) will be summarized descriptively by analysis population.

9.6.3 Safety Analyses

All subjects who received at least one dose of vamorolone (Safety Population) will be included in the safety analyses. In general, descriptive statistics for each safety endpoint will be presented by combined vamorolone dose level, individual dose levels, and other combinations of dose levels.

All safety data will be listed.

Safety data will include BMI (raw units and z-score), height (raw units and z-score), vital signs, clinical laboratory test results, spine x-ray results (spine fractures), hand x-ray results (bone age), and ECG results.

Changes in BMI z-score and height z-score will be analyzed using the same type of statistical models used for efficacy with vamorolone being compared with the daily prednisone and the daily deflazacort arms from the FOR DMD study. Hand x-ray and spine x-ray results will also be compared with the daily prednisone and the daily deflazacort arms from the FOR DMD study.

Adverse events will be summarized overall and by dose level by system organ class (SOC) and preferred term (using the Medical Dictionary for Regulatory Activities [MedDRA]); by relationship to study medication; and by intensity (CTCAE grade).

Physical examination results will be listed only.

9.6.4 Clinical Efficacy and Pharmacodynamic Analyses

The evaluations of clinical efficacy and PD will be performed using the FAS Population.

The primary efficacy outcome is TTSTAND (velocity). Secondary efficacy outcomes are the NSAA assessment, TTCLIMB, TTRW, QMT, and the 6MWT. TTSTAND, TTCLIMB, and TTRW will be analyzed using raw scores and velocity.

The primary outcome is TTSTAND (velocity) change from VBP15-002 study baseline to Month 24 and will be compared between vamorolone and historical untreated controls using a restricted maximum likelihood (REML)-based mixed model for repeated measures (MMRM). This model will include fixed effects for treatment, visit, baseline TTSTAND velocity (from the VBP15-002 study), age at study entry, and the treatment-by-visit interaction. The initial model will compare combined vamorolone doses to untreated natural history controls and subsequent secondary models may compare individual and other combinations of dose levels to untreated natural history controls. The secondary outcome measures will be compared using similar models. Full details will be provided in the Statistical Analysis Plan (SAP).

Serum PD biomarkers of adrenal axis suppression, insulin resistance, and bone turnover will be assessed, as well as exploratory biomarkers of safety and efficacy. Biomarkers will be summarized descriptively over time.

Individual clinical efficacy and PD data will be listed.

Section changed:

10.5 Study Monitoring
Paragraph 1

Original Text:

10.5 Study Monitoring

In accordance with applicable regulations, GCP, and the procedures of the Sponsor or its designees, the Study Monitor will periodically contact the site and conduct on-site visits. The extent, nature, and frequency of on-site visits will be based on enrollment rate and data quality at the site. Through frequent communications (e.g., letter, e-mail, and telephone), the Study Monitor will ensure that the investigation is conducted according to protocol and regulatory requirements.

Revised Text:

10.5 Data and Safety Monitoring Board (DSMB) and Other Study Monitoring

10.5.1 DSMB

An unblinded Data and Safety Monitoring Board (DSMB), operating autonomously from the Sponsor and the site investigators, will be responsible for providing independent recommendations to the Sponsor about risk-benefit of the study and for any modification affecting safety or data integrity required during the course of the study. The DSMB members must not be actively involved in study design, conduct or daily management of this study and must not have financial, proprietary, professional, or other interests that may affect impartial, independent decision-making.

Specialists may be invited to participate as non-voting members at any time if additional expertise is desired. The DSMB will formally interact with the Sponsor through the sharing of DSMB meeting minutes.

The DSMB will be responsible for:

- Examining accumulating safety and other relevant data at pre-specified points during the course of the study in order to make recommendations concerning continuation, termination, or modification of the study;
- Examining accumulating clinical efficacy data and comparing to corticosteroids and an untreated historical control group in order to make recommendations concerning continuation, termination, or modification of the study;
- Reviewing protocol violations;
- Providing expert advice to the Sponsor on an ad hoc basis regarding matters such as safety concerns or diagnostic evaluations in individual subjects;
- Based on the results of its deliberations, the DSMB can recommend continuation of the study unchanged, study interruption, study termination, modification of the studies, or alteration in the DSMB monitoring plan.

10.5.2 Study Monitoring

In accordance with applicable regulations, GCP, and the procedures of the Sponsor or its designees, the Study Monitor will periodically contact the site and conduct on-site visits.

The extent, nature, and frequency of on-site visits will be based on enrollment rate and data quality at the site. Through frequent communications (e.g., letter, e-mail, and telephone), the Study Monitor will ensure that the investigation is conducted according to protocol and regulatory requirements.

Section Changed:

13 References

Original Text:

29. Investigator's Brochure, Version 3, Vamorolone (17α,21-dihydroxy-16α-methyl-pregna-1,4,9(11)-triene-3,20-dione) 4% Oral Suspension, ReveraGen BioPharma, Inc., January 23, 2016.

Revised Text:

29. Investigator's Brochure, Version 8, Vamorolone (17α,21-dihydroxy-16α-methyl-pregna-1,4,9(11)-triene-3,20-dione) 1.33% and 4% Oral Suspension, ReveraGen BioPharma, Inc., September 24, 2018.

Original Text:

42. Hathout Y, Brody E, Clemens PR, et al. Large-scale serum protein biomarker discovery in Duchenne muscular dystrophy. *Proc Natl Acad Sci USA*. 2015; 112(23): 7153-8.
43. Bello L, Kesari A, Gordish-Dressman H, et al. Genetic modifiers of ambulation in the Cooperative International Neuromuscular Research Group Duchenne natural history study. *Ann Neurol*. 2015; 77(4):684-696.

Revised Text:

42. Hathout Y, Brody E, Clemens PR, et al. Large-scale serum protein biomarker discovery in Duchenne muscular dystrophy. *Proc Natl Acad Sci USA*. 2015; 112(23): 7153-8.
43. Conklin LS, Damsker JM, Hoffman EP, et al. Phase IIa trial in Duchenne muscular dystrophy shows vamorolone is a first-in-class dissociative steroidal anti-inflammatory drug. *Pharmacol. Res*. 2018 Sep 13; 136:140-50.
44. Bello L, Kesari A, Gordish-Dressman H, et al. Genetic modifiers of ambulation in the Cooperative International Neuromuscular Research Group Duchenne natural history study. *Ann Neurol*. 2015; 77(4):684-696.

Original Text:

47. Escolar DM, Hache LP, Clemens PR, et al. Randomized, blinded trial of weekend vs. daily prednisone in Duchenne muscular dystrophy. *Neurology.* 2011; 77(5):444-452.

Revised Text:

48. Guglieri M, Bushby K, McDermott MP, et al. Developing standardized corticosteroid treatment for Duchenne muscular dystrophy. *Contempt. Clin. Trials*. 2017 Jul; 58:34-39.
