# Supplementary material for: Efficacy and safety of vamorolone in Duchenne muscular dystrophy: An 18-month interim analysis of a non-randomized open-label extension study
Source: PLoS Med. 2020 Sep 21;17(9):e1003222. doi: 10.1371/journal.pmed.1003222 (PMC7505441; doi:10.1371/journal.pmed.1003222)
Supplement: S1 Table — (DOCX) [file pmed.1003222.s006.docx]

**S1 Table. Group-matching criteria for CINRG DNHS corticosteroid-treated and corticosteroid-naïve DMD participants.**

| **Corticosteroid-naïve group-matching criteria** |
| --- |
| Participants with 18 months of data defined as two study visits occurring between 16 and 20 months. |
| Participants between age 4 and <7 years old at the observation interval start (visit considered as Baseline for this iSAP). |
| Participants from all regions. |
| Participants who were not treated with corticosteroids (prednisone, deflazacort; any dose) at any time during 18-month period. |
| Participants not co-enrolled in other clinical trials. |
| Participants able to perform the time to run/walk 10m, time to stand from supine, and time to climb 4 stairs at the observation interval start. |
| Participants having both height and BMI assessments at both observation interval start and interval end (i.e. at 18 months). |
| **Corticosteroid-treated group matching criteria** |
| Participants with 18 months of data defined as two study visits occurring between 16 and 20 months. |
| Participants between age 4 and <7 years old at baseline visit. |
| Participants from all regions. |
| Participants who initiated corticosteroids (prednisone, deflazacort; any dose) prior to baseline visit, and were maintained on corticosteroids throughout the subsequent 18-month period (inclusive of those that start corticosteroids at the visit of the start of the 18-month period). |
| Participants not co-enrolled in other exon-skipping clinical trials. |
| Participants able to perform the time to run/walk 10m, time to stand from supine, and time to climb 4 stairs at the observation interval start. |
| Participants having both height and BMI assessments at both observation interval start and interval end (i.e. at 18 months). |
