# Supplementary material for: Efficacy and safety of vamorolone in Duchenne muscular dystrophy: An 18-month interim analysis of a non-randomized open-label extension study
Source: PLoS Med. 2020 Sep 21;17(9):e1003222. doi: 10.1371/journal.pmed.1003222 (PMC7505441; doi:10.1371/journal.pmed.1003222)
Supplement: S2 Table — CI, confidence interval; CINRG, Cooperative International Neuromuscular Research Group; DNHS, Duchenne Natural History Study; LS, least squares; SD, standard deviation; TTCLIMB, time to climb 4 stairs; TTRW, time to run/walk 10 meters; TTSTAND, time to stand from supine. (DOCX) [file pmed.1003222.s007.docx]

**S2 Table. Summary of comparisons between vamorolone-treated (2.0 and 6.0 mg/kg/day) and corticosteroid-naïve CINRG DNHS participants (efficacy endpoints reported in seconds).**

|  | N  Baseline/18-month | Baseline mean (SD) | 18-month mean (SD)^1^ | Change (SD)  [paired t-test p-value] | LS Mean Difference  (95% CI)  ANCOVA |
| --- | --- | --- | --- | --- | --- |
| **Efficacy** |  |  |  |  |  |
| **TTRW seconds** |  |  |  |  |  |
| Vamorolone | 23/22 | 5.99 (1.24) | 5.00 (0.976) | -1.06 (1.06) p=0.0001 | -0.84 (0.34)  (-1.54, -0.14)  p=0.02 |
| Corticosteroid-naïve DNHS | 19/18 | 6.78 (2.25) | 6.28 (1.95) | -0.51 (1.47) p=0.16 |  |
| **TTCLIMB seconds** |  |  |  |  |  |
| Vamorolone | 23/22 | 4.81 (2.61) | 4.37 (5.98) | -0.52 (4.84) p=0.62 | -0.34 (1.45)  (-3.28, 2.59)  p=0.81 |
| Corticosteroid-naïve DNHS | 19/18 | 5.77 (3.09) | 5.70 (5.18) | -0.05 (4.12) p=0.97 |  |
| **TTSTAND seconds** |  |  |  |  |  |
| Vamorolone | 23/22 | 5.54 (2.44) | 5.15 (4.66) | -0.48 (3.19) p=0.48 | -1.147 (0.85)  (-2.87, 0.57)  p=0.18 |
| Corticosteroid-naïve DNHS | 19/16 | 5.34 (1.63) | 5.19 (1.76) | 0.31 (1.29) p=0.36 |  |
