# Supplementary material for: Efficacy and safety of vamorolone in Duchenne muscular dystrophy: An 18-month interim analysis of a non-randomized open-label extension study
Source: PLoS Med. 2020 Sep 21;17(9):e1003222. doi: 10.1371/journal.pmed.1003222 (PMC7505441; doi:10.1371/journal.pmed.1003222)
Supplement: S3 Table — (DOCX) [file pmed.1003222.s008.docx]

**S3 Table: Mean (± SD change in Body Mass Index (BMI) for the VBP15-002/003 0.75 mg/kg/day (n=12), 2.0 mg/kg/day (n=11), and 6.0 mg/kg/day (n=11) dose group.**

Group B. 0.75 mg/kg/day dose group in VBP15-002/003

|  | N | Mean | Std Dev | Median | Minimum | Maximum |
| --- | --- | --- | --- | --- | --- | --- |
| BMI VBP15-002 Baseline | 12 | 16.53 | 1.49 | 16.75 | 14.10 | 18.60 |
| BMI VBP15-003 Week 24/VBP15-LTE Baseline | 12 | 16.33 | 1.60 | 16.60 | 13.60 | 18.50 |
| BMI VBP15-LTE Month 12 Visit (18 months treatment) | 12 | 17.03 | 2.35 | 16.70 | 14.60 | 23.30 |
| Change in BMI from VBP15-002 Baseline to VBP15-LTE Baseline (6 months treatment) | 12 | -0.20 | 0.632 | -0.2 | -1.00 | 1.30 |
| Change in BMI from VBP15-002 Baseline to VBP15-LTE Month 12 Visit (18 months treatment) | 12 | 0.5 | 2.02 | 0.05 | -1.60 | 6.20 |

Group C. 2.0 mg/kg/day dose group in VBP15-002/003

|  | N | Mean | Std Dev | Median | Minimum | Maximum |
| --- | --- | --- | --- | --- | --- | --- |
| BMI VBP15-002 Baseline | 12 | 17.25 | 0.84 | 17.30 | 15.90 | 18.60 |
| BMI VBP15-003 Week 24/VBP15-LTE Baseline | 11 | 17.51 | 0.97 | 17.50 | 16.30 | 19.40 |
| BMI VBP15-LTE Month 12 Visit (18 months treatment) | 11 | 18.45 | 2.01 | 17.80 | 16.30 | 22.80 |
| Change in BMI from VBP15-002 Baseline to VBP15-LTE Baseline (6 months treatment) | 11 | 0.309 | 0.85 | 0.10 | -0.40 | 2.60 |
| Change in BMI from VBP15-002 Baseline to VBP15-LTE Month 12 Visit (18 months treatment) | 11 | 1.11 | 1.98 | 0.90 | -0.80 | 5.10 |

Group D. 6.0 mg/kg/day dose group in VBP15-002/003

|  | N | Mean | Std Dev | Median | Minimum | Maximum |
| --- | --- | --- | --- | --- | --- | --- |
| BMI VBP15-002 Baseline | 11 | 16.69 | 0.815 | 16.90 | 15.30 | 17.90 |
| BMI VBP15-003 Week 24/VBP15-LTE Baseline | 11 | 17.85 | 1.48 | 17.90 | 15.90 | 21.50 |
| BMI VBP15-LTE Month 12 Visit | 11 | 19.24 | 2.50 | 18.90 | 16.10 | 24.90 |
| Change in BMI from VBP15-002 Baseline to VBP15-LTE Baseline (6 months treatment) | 11 | 1.16 | 1.35 | 1.00 | -0.70 | 4.10 |
| Change in BMI from VBP15-002 Baseline to VBP15-LTE Month 12 Visit (18 months treatment) | 11 | 2.55 | 2.10 | 2.20 | 0.10 | 7.50 |
