# Supplementary material for: Efficacy and safety of vamorolone in Duchenne muscular dystrophy: An 18-month interim analysis of a non-randomized open-label extension study
Source: PLoS Med. 2020 Sep 21;17(9):e1003222. doi: 10.1371/journal.pmed.1003222 (PMC7505441; doi:10.1371/journal.pmed.1003222)
Supplement: S4 Table — (DOCX) [file pmed.1003222.s009.docx]

S4 Table. Number of Treatment Emergent Adverse Events by System Organ Class and PT

|  | **Dose (mg/kg/day)** | | | | |
| --- | --- | --- | --- | --- | --- |
| **System Organ Class** | **0.25** | **0.75** | **2.0** | **4.0** | **6.0** |
| **Total Number of participant days on dose** | **968** | **1421** | **10462** | **255** | **18960** |
| **Blood and lymphatic system disorders** | 0 | 1 | 0 | 0 | 0 |
| Microcytic anaemia | 0 | 1 | 0 | 0 | 0 |
| **Cardiac disorders** | 0 | 1 | 0 | 0 | 2 |
| Palpitations | 0 | 0 | 0 | 0 | 1 |
| Tachycardia | 0 | 1 | 0 | 0 | 1 |
| **Ear and labyrinth disorders** | 0 | 0 | 5 | 0 | 3 |
| Ear pain | 0 | 0 | 5 | 0 | 2 |
| Motion sickness | 0 | 0 | 0 | 0 | 1 |
| **Endocrine disorders** | 0 | 0 | 0 | 0 | 1 |
| Cushingoid | 0 | 0 | 0 | 0 | 1 |
| **Gastrointestinal disorders** | 1 | 4 | 22 | 0 | 41 |
| Abdominal discomfort | 0 | 0 | 1 | 0 | 0 |
| Abdominal pain | 0 | 0 | 0 | 0 | 4 |
| Abdominal pain upper | 0 | 0 | 3 (1)* | 0 | 4 |
| Bowel movement irregularity | 0 | 0 | 0 | 0 | 1 |
| Constipation | 0 | 0 | 4 (3) | 0 | 5 |
| Dental caries | 0 | 1 | 0 | 0 | 1 |
| Diarrhoea | 0 | 1 | 2 (1) | 0 | 3 |
| Dyspepsia | 0 | 0 | 1 | 0 | 0 |
| Faeces discoloured | 0 | 0 | 1 | 0 | 0 |
| Frequent bowel movements | 0 | 0 | 1 | 0 | 0 |
| Gastrointestinal disorder | 0 | 0 | 0 | 0 | 2 (1) |
| Gastrointestinal pain | 0 | 0 | 0 | 0 | 2 |
| Gastrooesophageal reflux disease | 0 | 0 | 0 | 0 | 1 |
| Haematochezia | 0 | 0 | 1 | 0 | 0 |
| Nausea | 0 | 1 | 0 | 0 | 7 (1) |
| Oral pain | 0 | 0 | 0 | 0 | 1 |
| Toothache | 0 | 0 | 0 | 0 | 1 |
| Vomiting | 1 | 1 | 8 (5) | 0 | 9 (8) |
| **General disorders and administration site conditions** | 3 | 5 | 26 | 0 | 27 |
| Abasia | 0 | 0 | 0 | 0 | 1 |
| Chest pain | 0 | 2 | 2 (1) | 0 | 3 |
| Fatigue | 0 | 1 | 0 | 0 | 2 |
| Gait disturbance | 0 | 0 | 1 | 0 | 0 |
| Gait inability | 0 | 0 | 0 | 0 | 1 |
| Influenza like illness | 0 | 1 | 0 | 0 | 2 |
| Medical device site dermatitis | 0 | 0 | 0 | 0 | 1 |
| Medical device site pain | 1 | 0 | 0 | 0 | 0 |
| Medical device site rash | 1 | 0 | 0 | 0 | 0 |
| Pyrexia | 1 | 1 | 23 (9) | 0 | 17 (10) |
| **Immune system disorders** | 1 | 0 | 4 | 0 | 1 |
| Hypersensitivity | 1 | 0 | 2 (1) | 0 | 0 |
| Seasonal allergy | 0 | 0 | 2 | 0 | 1 |
| **Infections and infestations** | 5 | 12 | 53 | 0 | 51 |
| Body tinea | 1 | 0 | 0 | 0 | 0 |
| Bronchitis | 0 | 1 | 0 | 0 | 0 |
| Conjunctivitis | 0 | 0 | 1 | 0 | 2 (1) |
| Croup infectious | 0 | 0 | 0 | 0 | 1 |
| Ear infection | 0 | 1 | 2 | 0 | 2 |
| Enterobiasis | 0 | 0 | 0 | 0 | 1 |
| Gastroenteritis | 0 | 0 | 3 | 0 | 3 (2) |
| Gastroenteritis viral | 0 | 0 | 0 | 0 | 2 |
| Hand-foot-and-mouth disease | 1 | 0 | 0 | 0 | 0 |
| Helminthic infection | 0 | 0 | 1 | 0 | 0 |
| Influenza | 0 | 1 | 2 | 0 | 5 |
| Localised infection | 0 | 0 | 0 | 0 | 1 |
| Lung infection | 0 | 0 | 0 | 0 | 1 |
| Molluscum contagiosum | 0 | 0 | 0 | 0 | 1 |
| Nasopharyngitis | 0 | 1 | 25 (7) | 0 | 17 (11) |
| Otitis externa | 0 | 0 | 1 | 0 | 0 |
| Otitis media | 0 | 0 | 3 | 0 | 2 |
| Pharyngitis streptococcal | 2 (1) | 4 (2) | 3 (1) | 0 | 2 |
| Pneumonia | 0 | 1 | 0 | 0 | 0 |
| Postoperative wound infection | 0 | 0 | 1 | 0 | 0 |
| Respiratory syncytial virus infection | 0 | 0 | 1 | 0 | 0 |
| Rhinitis | 0 | 0 | 1 | 0 | 0 |
| Sinusitis | 1 | 0 | 1 | 0 | 1 |
| Tonsillitis | 0 | 1 | 2 (1) | 0 | 0 |
| Tooth abscess | 0 | 0 | 0 | 0 | 1 |
| Upper respiratory tract infection | 0 | 2 | 5 (4) | 0 | 7 (4) |
| Viral infection | 0 | 0 | 0 | 0 | 1 |
| Viral upper respiratory tract infection | 0 | 0 | 1 | 0 | 1 |
| **Injury, poisoning and procedural complications** | 1 | 2 | 23 | 0 | 20 |
| Arthropod bite | 1 | 1 | 0 | 0 | 1 |
| Back injury | 0 | 0 | 0 | 0 | 1 |
| Concussion | 0 | 0 | 1 | 0 | 0 |
| Contusion | 0 | 0 | 1 | 0 | 0 |
| Face injury | 0 | 0 | 0 | 0 | 1 |
| Fall | 0 | 1 | 5 (3) | 0 | 7 (4) |
| Foot fracture | 0 | 0 | 1 | 0 | 0 |
| Head injury | 0 | 0 | 4 (1) | 0 | 2 (1) |
| Humerus fracture | 0 | 0 | 0 | 0 | 1 |
| Joint injury | 0 | 0 | 2 | 0 | 0 |
| Laceration | 0 | 0 | 1 | 0 | 2 (1) |
| Ligament sprain | 0 | 0 | 1 | 0 | 0 |
| Limb injury | 0 | 0 | 4 (3) | 0 | 0 |
| Muscle strain | 0 | 0 | 1 | 0 | 0 |
| Nail injury | 0 | 0 | 0 | 0 | 1 |
| Procedural headache | 0 | 0 | 0 | 0 | 1 |
| Skin injury | 0 | 0 | 0 | 0 | 1 |
| Spinal fracture | 0 | 0 | 1 | 0 | 0 |
| Upper limb fracture | 0 | 0 | 0 | 0 | 2 |
| Wound | 0 | 0 | 1 | 0 | 0 |
| **Investigations** | 0 | 0 | 3 | 0 | 15 |
| Blood cholesterol increased | 0 | 0 | 0 | 0 | 1 |
| Blood triglycerides increased | 0 | 0 | 0 | 0 | 3 |
| Low density lipoprotein increased | 0 | 0 | 0 | 0 | 1 |
| Platelet count decreased | 0 | 0 | 0 | 0 | 2 |
| Vitamin D decreased | 0 | 0 | 0 | 0 | 1 |
| Weight increased | 0 | 0 | 3 | 0 | 7 |
| **Metabolism and nutrition disorders** | 0 | 1 | 0 | 1 | 5 |
| Dehydration | 0 | 1 | 0 | 0 | 0 |
| Hyperlipidaemia | 0 | 0 | 0 | 1 | 1 |
| Increased appetite | 0 | 0 | 0 | 0 | 2 |
| Polydipsia | 0 | 0 | 0 | 0 | 1 |
| Vitamin D deficiency | 0 | 0 | 0 | 0 | 1 |
| **Musculoskeletal and connective tissue disorders** | 0 | 3 | 17 | 0 | 14 |
| Arthralgia | 0 | 1 | 0 | 0 | 0 |
| Back pain | 0 | 0 | 2 | 0 | 1 |
| Groin pain | 0 | 0 | 0 | 0 | 1 |
| Muscle disorder | 0 | 0 | 1 | 0 | 0 |
| Muscle hypertrophy | 0 | 0 | 1 | 0 | 0 |
| Muscle spasms | 0 | 0 | 2 | 0 | 3 |
| Musculoskeletal pain | 0 | 0 | 0 | 0 | 1 |
| Myalgia | 0 | 0 | 1 | 0 | 0 |
| Pain in extremity | 0 | 2 | 9 (6) | 0 | 6 (4) |
| Pain in jaw | 0 | 0 | 0 | 0 | 1 |
| Plantar fasciitis | 0 | 0 | 1 | 0 | 1 |
| **Neoplasms benign, malignant and unspecified (incl cysts and polyps)** | 0 | 0 | 1 | 0 | 1 |
| Skin papilloma | 0 | 0 | 1 | 0 | 1 |
| **Nervous system disorders** | 0 | 1 | 7 | 0 | 14 |
| Dizziness | 0 | 0 | 0 | 0 | 1 |
| Headache | 0 | 0 | 7 (4) | 0 | 9 (6) |
| Middle insomnia | 0 | 1 | 0 | 0 | 0 |
| Migraine | 0 | 0 | 0 | 0 | 1 |
| Paraesthesia | 0 | 0 | 0 | 0 | 1 |
| Presyncope | 0 | 0 | 0 | 0 | 1 |
| Somnolence | 0 | 0 | 0 | 0 | 1 |
| **Psychiatric disorders** | 2 | 1 | 3 | 0 | 4 |
| Aggression | 1 | 0 | 0 | 0 | 0 |
| Agitation | 0 | 0 | 1 | 0 | 0 |
| Anxiety | 1 | 0 | 0 | 0 | 0 |
| Insomnia | 0 | 0 | 1 | 0 | 0 |
| Irritability | 0 | 1 | 0 | 0 | 2 |
| Personality change | 0 | 0 | 0 | 0 | 1 |
| Sleep disorder | 0 | 0 | 1 | 0 | 1 |
| **Renal and urinary disorders** | 0 | 0 | 0 | 0 | 4 |
| Haematuria | 0 | 0 | 0 | 0 | 1 |
| Myoglobinuria | 0 | 0 | 0 | 0 | 2 (1) |
| Polyuria | 0 | 0 | 0 | 0 | 1 |
| **Reproductive system and breast disorders** | 0 | 0 | 0 | 0 | 1 |
| Balanoposthitis | 0 | 0 | 0 | 0 | 1 |
| **Respiratory, thoracic and mediastinal disorders** | 1 | 6 | 16 | 0 | 26 |
| Acute respiratory failure | 0 | 1 | 0 | 0 | 0 |
| Cough | 1 | 1 | 11 (8) | 0 | 10 (8) |
| Epistaxis | 0 | 0 | 1 | 0 | 3 (2) |
| Hypoxia | 0 | 1 | 0 | 0 | 0 |
| Nasal congestion | 0 | 0 | 0 | 0 | 7 (3) |
| Oropharyngeal pain | 0 | 1 | 1 | 0 | 4 (2) |
| Pleural effusion | 0 | 1 | 0 | 0 | 0 |
| Rhinitis allergic | 0 | 0 | 1 | 0 | 1 |
| Sleep apnoea syndrome | 0 | 0 | 1 | 0 | 1 |
| Snoring | 0 | 0 | 1 | 0 | 0 |
| Tachypnoea | 0 | 1 | 0 | 0 | 0 |
| **Skin and subcutaneous tissue disorders** | 1 | 3 | 2 | 0 | 6 |
| Erythema | 0 | 0 | 1 | 0 | 0 |
| Pityriasis rosea | 0 | 1 | 0 | 0 | 0 |
| Pruritus | 0 | 0 | 1 | 0 | 0 |
| Rash | 0 | 2 | 0 | 0 | 2 (1) |
| Rash generalised | 0 | 0 | 0 | 0 | 1 |
| Rash macular | 1 | 0 | 0 | 0 | 0 |
| Rash papular | 0 | 0 | 0 | 0 | 1 |
| Urticaria | 0 | 0 | 0 | 0 | 2 (1) |
| **Surgical and medical procedures** | 0 | 0 | 1 | 0 | 5 |
| Adenoidectomy | 0 | 0 | 0 | 0 | 1 |
| Nasal polypectomy | 0 | 0 | 1 | 0 | 0 |
| Tonsillectomy | 0 | 0 | 0 | 0 | 2 (1) |
| Tooth extraction | 0 | 0 | 0 | 0 | 1 |
| Umbilical hernia repair | 0 | 0 | 0 | 0 | 1 |
| **Vascular disorders** | 0 | 1 | 1 | 0 | 0 |
| Haematoma | 0 | 0 | 1 | 0 | 0 |
| Pallor | 0 | 1 | 0 | 0 | 0 |
| **Total** | 15 | 41 | 184 | 1 | 241 |

* Shows the number of AE’s reported by the number of participants in parenthesis. For example, 2(1) shows that there were two adverse events reported by one participant.
