# Supplementary material for: Efficacy and safety of vamorolone in Duchenne muscular dystrophy: An 18-month interim analysis of a non-randomized open-label extension study
Source: PLoS Med. 2020 Sep 21;17(9):e1003222. doi: 10.1371/journal.pmed.1003222 (PMC7505441; doi:10.1371/journal.pmed.1003222)
Supplement: Attachment — Submitted filename: smith.pdf [file pmed.1003222.s010.pdf]

This paper reports interim results from a Phase 2 study of a therapeutic agent for Duchenne muscular dystrophy.

I was asked for a statistical report and I interpret that to include all aspects of the design and conduct of the study.

Points of detail

Page 2 Here the participants are referred to as participants (or as young boys) both of which seem preferable to me to subjects which is used repeatedly including in the title.

Pages 9 and 10 Rather than repeat the p–values here I would suggest it would be preferable to use the measure of effect size with its confidence interval. Arguably since they are all in the table it is unnecessary to repeat them at all in the text.

Page 9 I did not understand the parenthesis about Group A, specifically what the phrase older age refers to.

Page 10 In studies of child development in low and middle income countries with which I am familiar authors usually use height for age (HAZ) and weight for age (WAZ) rather than BMI. In a group of boys where height is changing rapidly at least some of the change in BMI must be accounted for by change in height so I think the authors need to convince us of the merits of BMI over WAZ.

Page 13 I think that the members of the safety monitoring board should be named here. Safety was an important issue in this study and they clearly had an important role in patient welfare.

Page 18, Figure 1 The dot plot in the left hand panels seems to me superior to the box plots in the right hand ones since we can actually see the data. The caption does not seem to describe the right hand panels unless I am missing something.

Page 19, Table 1 The race and ethnicity categories do not mean too much outside the United States as we do not have Hispanic as a category. I notice there is an international authorship. Were all these boys in the United States? It is rather hard to determine this as the list of contributors on page 14 has some initials which do not appear in the author list of the paper.

Page 23, Figure S1 This reveals that the early entrants to VBP15–LTE were on the lower doses. Is there an explanation for this?

Points of more substance

People who cannot do the task

It is always hard to know what to do with the analysis of tasks where some people cannot perform it at all. I did not find the authors' proposed solution of converting times to velocities and imputing zero very convincing. As a sensitivity analysis I would suggest repeating this using a ranking method with unable given the worst ranking. This has the disadvantage that the corresponding measures of effect size are unfamiliar so despite my misgivings the velocity analysis does have that advantage.

Adverse events reporting

It seems to me that one key feature of a Phase 2 study is the search for possible adverse events. I think these are rather under–reported and I would suggest that in the supplementary material we should have a complete list possibly with details like the dose. This would have advantages if in years to come researchers want to know about a specific event.

Interim reporting

The continuation phase was planned to last for 24 months but we have here a report on the first 12. I did not see a clear presentation of how and why this decision was made.

Summary

Mostly points to clarify and suggestions for enhancements.

Michael Dewey
