# Supplementary material for: Efficacy and safety of vamorolone in Duchenne muscular dystrophy: An 18-month interim analysis of a non-randomized open-label extension study
Source: PLoS Med. 2020 Sep 21;17(9):e1003222. doi: 10.1371/journal.pmed.1003222 (PMC7505441; doi:10.1371/journal.pmed.1003222)
Supplement: Attachment — Submitted filename: Response to reviews.docx [file pmed.1003222.s011.docx]

22 April 2020

Emma Veitch, PhD
PLOS Medicine

Dear Dr. Veitch,

Thank you for the highly constructive comments on our manuscript submission to PLOS Medicine.

We have revised the manuscript according to the suggestions of the three reviewers and Editor.

Please find below the point-by-point responses.

We look forward to your final decision.

Best regards,

Eddie Smith

Duke University

**Requests from the editors:**

*Please revise your title according to PLOS Medicine's style. Your title must be nondeclarative and not a question. It should begin with main concept if possible. "Effect of" should be used only if causality can be inferred, i.e., for an RCT. Please place the study design ("A randomized controlled trial," "A retrospective study," "A modelling study," etc.) in the subtitle (ie, after a colon). Although the title and abstract currently state the study as an open-label study, this doesn't fully explain the study design (eg, whether randomized or not), and we'd suggest including that per the journal's usual style.

- *We have modified the title to: “*Efficacy and safety of vamorolone in Duchenne muscular dystrophy: An 18-month non-randomized open-label extension study.”

*Please structure your abstract using the PLOS Medicine headings (Background, Methods and Findings, Conclusions). The Methods and Findings sections should be one single sub-section, “Methods and findings”.

- *We have modified the Abstract as suggested.*

*At this stage, we ask that you include a short, non-technical Author Summary of your research to make findings accessible to a wide audience that includes both scientists and non-scientists. The Author Summary should immediately follow the Abstract in your revised manuscript. This text is subject to editorial change and should be distinct from the scientific abstract. Please see our author guidelines for more information: [https://urldefense.com/v3/__https://journals.plos.org/plosmedicine/s/revising-your-manuscript*loc-author-summary__;Iw!!OToaGQ!_wNmEyPDrckkrGqeeIgnh6ij1lWpUvIhPHnH3VnELTAeJ98RIbAAm0QCIrlsNMh8Cgk$](https://urldefense.com/v3/__https:/journals.plos.org/plosmedicine/s/revising-your-manuscript*loc-author-summary__;Iw!!OToaGQ!_wNmEyPDrckkrGqeeIgnh6ij1lWpUvIhPHnH3VnELTAeJ98RIbAAm0QCIrlsNMh8Cgk$)

- *We have added an Author Summary.*

*The referencing style used in the paper doesn't match PLOS Medicine's usual format (the paper uses author name-year system). Please change to numbered referencing in square brackets and then a numbered reference list.

- *We have modified the referencing style.*

*One reviewer has said that part of the Results section reads like part of the Methods, however the editors would disagree with this point and would advise you to keep this information within the Results, however it could probably be presented more efficiently.

- *We have left the original as requested.*

Regarding the CONSORT checklist, review of the PLOS Medicine *Instructions for Authors* suggest using the “TREND” checklist for non-randomized studies. We have filled out the TREND checklist and submitted as an appendix. We have also submitted the full protocol as an appendix as required in *Instructions for Authors.*

-----------------------------------------------------------

**Comments from the reviewers:**

**Reviewer #1:**

This paper reports interim results from a Phase 2 study of a therapeutic agent for Duchenne muscular dystrophy.

I was asked for a statistical report and I interpret that to include all aspects of the design and conduct of the study.

**Points of detail**

**Page 2** Here the participants are referred to as participants (or as young boys) both of which seem preferable to me to subjects which is used repeatedly including in the title.

*Response: We have changed ‘subjects’ to ‘participants’ throughout the text.*

**Pages 9 and 10** Rather than repeat the *p*–values here I would suggest it would be preferable to use the measure of effect size with its confidence interval. Arguably since they are all in the table it is unnecessary to repeat them at all in the text.

*Response: We have added effect size with confidence intervals in the text as requested.*

**Page 9** I did not understand the parenthesis about Group A, specifically what the phrase older age refers to.

*Response: We have modified the cited text to be more clear and specific. It now reads, “*Groups B, C, and D each showed improvements from baseline after 18 months of treatment in comparison to corticosteroid-naïve participants from CINRG DNHS, whereas group A was similar to corticosteroid-naïve participants. Of note, group A was treated for only 3 to 9 months with high-dose vamorolone (**Supplemental Figure 1**), and was also 0.4 years older than other groups at study entry (Group A 5.2 ± 1.0 years; Groups B, C, D 4.8 ± 0.8 years).”

**Page 10** In studies of child development in low and middle income countries with which I am familiar authors usually use height for age (HAZ) and weight for age (WAZ) rather than BMI. In a group of boys where height is changing rapidly at least some of the change in BMI must be accounted for by change in height so I think the authors need to convince us of the merits of BMI over WAZ.

*Response: We had included BMI and height Z-scores in the text, and Z-scores accomplish the goal requested by the reviewer (Z-scores are age-specific values).*

**Page 13** I think that the members of the safety monitoring board should be named here. Safety was an important issue in this study and they clearly had an important role in patient welfare.

*Response: This is the first time that giving names of DSMB has been requested for any of our publications. The DSMB is run independently from the Sponsor (academic medical experts). We are asking the DSMB members if they are comfortable with providing their names, and will add to galleys if they are unanimous in their consent.*

**Page 18, Figure 1** The dot plot in the left hand panels seems to me supe- rior to the box plots in the right hand ones since we can actually see the data. The caption does not seem to describe the right hand panels unless I am missing something.

*Response: The Figure 1 legend did include a description of both Left and Right panels; we have now bolded these labels to make them more obvious. The left panels show patient-level data, and right panels show mean changes in each group relative to baseline.*

**Page 19, Table 1** The race and ethnicity categories do not mean too much outside the United States as we do not have Hispanic as a category. I notice there is an international authorship. Were all these boys in the United States? It is rather hard to determine this as the list of contributors on page 14 has some initials which do not appear in the author list of the paper.

*Response: We have added text to Methods (participants) as follows: “*Participants were recruited in the USA, Canada, United Kingdom, Sweden, Israel, and Australia.

**Page 23, Figure S1** This reveals that the early entrants to VBP15–LTE were on the lower doses. Is there an explanation for this?

*Response: We have revised Figure S1 to make this clearer. The antecedent VBP15-002 and VBP15-003 studies, from which participants enrolled into VBP15-LTE, were a dose-escalation PK/Tox study (VBP15-002) and dose-finding study (VBP15-003). This is now made clear in the revised Figure S1. This now answers the reviewer’s comment regarding why the early entrants to VBP15-LTE were on the lower doses.*

**Points of more substance**

**People who cannot do the task**

It is always hard to know what to do with the analysis of tasks where some people cannot perform it at all. I did not find the authors’ proposed solution of converting times to velocities and imputing zero very convincing. As a sensitivity analysis I would suggest repeating this using a ranking method with unable given the worst ranking. This has the disadvantage that the corresponding measures of effect size are unfamiliar so despite my misgivings the velocity analysis does have that advantage.

*Response: The use of velocities with TTSTAND in DMD is based on sensitivity analyses (comparative sample size calculations for power) in our previous reports (Hoffman et al. 2019). The ranking method suggested by the reviewer is used in the NorthStar Ambulatory Assessment outcome measure described in the manuscript. However, with NSAA, it is well-established that DMD patients improve without therapeutic interventions in the young age range studied (recruitment at 4 to <7 years) due to the neuro-developmental components of this measure that improve despite the DMD disease state (as acknowledged by Reviewer 3 below). To our knowledge, there is not a precedent for using a ranking method for TTSTAND in isolation (ranked assessment of TTSTAND is a component of NSAA). We feel that there would be a loss of power by a conversion to a ranking rather than the current continuous variable, particularly as there are very few participants that have lost the ability to do the test in the time frame of the reported LTE study.*

**Adverse events reporting**

It seems to me that one key feature of a Phase 2 study is the search for possible adverse events. I think these are rather under–reported and I would suggest that in the supplementary material we should have a complete list possibly with details like the dose. This would have advantages if in years to come researchers want to know about a specific event.

*Response: We have added a complete list of TEAEs in a new Supplemental Table 4, as requested.*

**Interim reporting**

The continuation phase was planned to last for 24 months but we have here a report on the first 12. I did not see a clear presentation of how and why this decision was made.

*Response: We have added text to the beginning of the Discussion explain that the interim analysis was done for dose-finding for the pivotal study, “*The main purpose of the VBP15-LTE was assessment of long-term safety and efficacy to support dose-selection in an ongoing Phase II/III study (VBP15-004).”

**Summary**

Mostly points to clarify and suggestions for enhancements.

*Response: We do not think these suggestions were transmitted with the review.*

**Reviewer #2**:

The abstract is well written.

The introduction is well written.

*Response: Thank you. No revisions requested.*

In the subjects and methods section, the obvious weakness of this trial, open label, was acknowledged.
The specific characteristics used to match the CINRG DNHS boys to the vamorolone trial boys should be spelled out in full, not relegated to the statement, "…and other characteristics (Supplemental Table 1)."  Otherwise, there is no means to know the composition of the comparator group.

*Response: Regarding comparators, there was text in both Methods – Participants, and Methods – Trial Design. We have noted that all matching criteria were in Supplemental Table 1, and have now added additional text to Methods – Trial Design as follows: “*Subjects were matched for age and treatment period (±1 month), and matching criteria were pre-specified in the statistical analysis plan, and matching carried out by two independent statisticians (Supplemental Table 1).”

Results:
 - The first five paragraphs in the Results section read like a Methods section.

*Response: The Editor suggested that we leave text as it was originally.*

 - It remains difficult to read results presented in velocities (however statistically appropriate).

*Response: We agree, and present both in the manuscript (raw data, and velocities).*

 - Opinion comments are best left to the Discussion section.  There are numerous comments throughout the Results section.

*Response: We studied the Results for offending statements. It seems that reaching an interpretation (conclusion) at the end of paragraphs was viewed by the reviewer as an opinion comment, but could alternatively be considered a summary of findings in that section. We did not find overtly subjective text that we felt should be left to Discussion.*

Discussion:
This is written well.
It is key that you have mentioned the significant limitations associated with this trial design.
Figure 1:  This is informative.

*Response: Thank you. No revisions requested.*

Table 1 is well laid out and comprehensively inclusive (mean, standard deviation, median, minimum, maximum).  I am not sure all of this information is necessary in the table.  Some could be placed in supplementary files.

Table 2 is well laid out and the numbers easy to see.  However, the use of velocity measurements is not easily understood by most readers.  Although statistically appropriate, it might be more reasonable to use raw data and discuss that velocity measurements confirmed (or reinforced) the results seen in the untransformed data.

*Response: We had included ‘seconds’ in Supplemental Table 2, and cross-reference this as an alternative to ‘velocity’ in the text. As noted in the text (and acknowledged by the reviewer), ‘velocity’ was our pre-specified primary and secondary outcomes in the trial, and we feel it best to keep the pre-specified variables in the text proper, and the non-pre-specified in the Supplemental materials.*

Table 3 is well laid out and easy to understand.

New agents for the treatment of dystrophinopathies are important.
Corticosteroid-like agents, such as vamorolone, with the potential for fewer side effects are needed.
However, overall, this article is a very difficult read with multiple comparator groups (many of which are historical) and obtuse measurements across these multiple comparator groups.

*Response: The concern that does not seem addressed above is “obtuse measurements”; perhaps the reviewer is referring to ‘velocity’ instead of ‘seconds’, but as spelled out in the manuscript, this was pre-specified and warranted by statistical considerations (retaining data from subjects unable to complete a test in a progressive disease), and sample size calculations from pre-existing data.*

-----------------------------------------------------------

**Reviewer #3**: The authors report on the 18-month safety and efficacy of an open-label trial of vamorolone in young boys with Duchenne muscular dystrophy with the aims of describing the safety and efficacy of this compound compared to external steroid naive and treated cohorts over a similar timeframe. Overall this study is useful to the field in providing an early view of signals of efficacy and safety of this compound as an alternative to traditional corticosteroid treatment options.

Major comments:
-There is inconsistent naming/labeling of groups/cohorts across text and figures, as well as differences in groups included in analysis that makes it challenging for the reader to easily follow and compare outcomes and safety across groups (specifically Supplemental Table 1). Additionally, the first paragraph states "Given the variable timing of dose escalations, we pre-specified that initial analyses of drug-related efficacy and safety would be limited to those subjects who had 18 months of treatment with 2.0 mg/kg/day vamorolone or more (dose group C + dose group D; n=23)." yet group A and B appear in Figure 1 and group B throughout the reported safety data.

*Response: There were primary analyses pre-specified in a Statistical Analysis Plan combining subjects that had been treated on high dose vamorolone for a full 18-months, and then a post-hoc analyses of dose-stratified subjects. We have now added text to better clarify this in the beginning of the Statistical Analysis, as follows: “*The statistical analyses was carried out in two sequential parts. First, we pre-specified groups and comparisons in a formal statistical analysis plan (SAP). This included only those vamorolone-treated subjects that had been on 2.0 and/or 6.0 mg/kg/day for the full 18-month treatment period (dose groups C + D; Table 1). This was to avoid the confounding variable of multiple dose-escalations in dose groups A and B (Supplemental Figure 1). The second analysis that was done post hoc was dose-stratified based on initial dose group in VBP15-002 (0.25, 0.75, 2.0, 6.0 mg/kg/day).”

-I would strongly recommend including steroid-treated functional data across tables and figures. Comparison of functional and safety data to a steroid-naive cohort has some utility, but the overall comparison to steroid-treated groups is more meaningful for clinicians in determining the best treatment options for their patients. While the variability in steroid treatment initiation is stated as the factor for not including this data, it would be preferential to include the data with the caveat that this variability in treatment duration may or may not impact findings.  Additionally, it is included as a comparator for safety data and one would expect the same concern to stand for that comparison, yet it is included.

*Response: The study team has extensively debated the important issues raised by the reviewer while designing the statistical analysis plan for this interim study. Key points of debate are the Reviewer’s rationale in the 2^nd^ half of the comment, namely, “*While the variability in steroid treatment initiation is stated as the factor for not including this data, it would be preferential to include the data with the caveat that this variability in treatment duration may or may not impact findings. Additionally, it is included as a comparator for safety data and one would expect the same concern to stand for that comparison, yet it is included*.” Efficacy of corticosteroids in DMD is acknowledged to have a strong acute component – with clear improvement within the first 6 months of treatment. As patient visits in the corticosteroid-treated comparator group had no baseline available (as is often the case with natural history studies), and given that the natural history visits were typically a year apart, much of the efficacy seen with corticosteroid-treated patients would be experienced prior to the ‘baseline’ utilized in this study. For this reason, the study team felt it was inappropriate to look at change from baseline in the corticosteroid-treated DMD comparator group, and this such analysis was purposefully not pre-specified in the statistical analysis plan. Regarding safety assessments, the reviewer correctly points out that safety analyses were done longitudinally (over time) in the corticosteroid-treated patients. This is because stunting of growth and weight gain are established to have a linear ‘rate’, and not an acute effect (as with efficacy). Thus, longitudinal comparative assessments of these safety findings were felt to be a valid comparison, and were pre-specified in the statistical analysis plan by consensus of the study team. For these reasons we ask the reviewer to reconsider her/his position, as we feel that inclusion of baseline data for corticosteroid-treated comparators will lead to an artificially elevated function at baseline, and obscure rather than clarify data comparisons.*

-Table 3 and the final paragraph of page 11 differs from the text in the previous paragraph on page 11 discussing AE's from the DSMB report (03Dec19). While this specific analysis is favorable for vamorolone, could be misleading to readers as weight gain appears to be quite common as evidenced by 10 AEs and similar increases in BMI. Additionally the authors comment on page 10 "This suggests that vamorolone may share the safety concern of weight gain with corticosteroids, although BMI does not distinguish between bone, muscle, and fat, yet adjusts for height. Each of these variables may be different for vamorolone-treatment and corticosteroid-treatment." The text "although BMI...." through the end of the next sentence should be removed as there is no evidence for this statement included in this study. Lastly, the vamorolone trial is open-label and being compared to randomized trials, so bias has the potential to significantly impact the difference in reporting.

*Response: Text removed as suggested.*

Additional comments:
- Improvements in the 6MWT and NSAA are generally expected in this <7 age range. There are other external cohorts to compare these improvements which could be considered. A graph of individual patient improvements should be considered as it may be helpful in interpreting these changes.

*Response: We did pursue multiple additional cohorts, including Italian network, British NorthStar network, and FOR-DMD clinical trial. None were available, due to challenges in data management and data queries (Italian, NorthStar), and challenges in synchronizing timelines (FOR-DMD; they wish to publish primary papers in late 2020 or early 2021 before considering comparator studies). There are cohorts from previous clinical trials, but all use steroid-treatment as a baseline (and placebo), and typically are an older age. Regarding graph of individual patient improvements, we tried this, but found that this ‘spaghetti plot’ was challenging to interpret with 46 subjects.*

- The graphs on the right of Figure 1 appear to indicate more variability in performance in the vamorolone-treated cohorts than the steroid-treated group which makes the reader question whether there is a more variable response rate to vamorolone treatment than traditional corticosteroids? Could the authors comment on this variability as standard deviations in performance at baseline seem similar to the steroid-naive group.

*Response: The smaller variability in steroid-treated is due to the much larger number of subjects studied (n=68), compared to vamorolone-treated groups (n=11-12/group). We have revised the manuscript to add the number of subjects shown to both the Figure legend, and Results section to make this more clear.*

-----------------------------------------------------------
