# Supplementary material for: Efficacy and safety of vamorolone in Duchenne muscular dystrophy: An 18-month interim analysis of a non-randomized open-label extension study
Source: PLoS Med. 2020 Sep 21;17(9):e1003222. doi: 10.1371/journal.pmed.1003222 (PMC7505441; doi:10.1371/journal.pmed.1003222)
Supplement: Attachment — Submitted filename: Response to critiques R3.docx [file pmed.1003222.s012.docx]

14 July 2020

RE: PMEDICINE-D-20-00478R3

Caitlin Moyer, Ph.D.
Associate Editor
PLOS Medicine
[plosmedicine.org](http://plosmedicine.org/)

Dear Dr. Moyer,

Thank you for the thoughtful review of our manuscript for PLOS Medicine entitled, "Efficacy and safety of vamorolone in Duchenne muscular dystrophy: An 18-month interim analysis of a non-randomized open-label extension study."

The two outstanding issues noted in your most recent communication are listed below.

1. Please make sure all references are cited in ascending numerical order in the text.

*I was unable to find references in the manuscript that were not in numerical order, therefore no changes to the manuscript were made. Please advise.*

2. S1 Protocol and iSAP: Page 2 of the S1 Protocol still says “confidential” at the bottom, and there is also a proprietary information statement at the bottom of the first page. Page 2 of the iSAP is still marked confidential. Please include versions of these two documents with this text removed (and any other text that would restrict sharing of these documents).

*Redacted versions of these documents have been uploaded.*

Please let me know if you require anything further from me. I look forward to your final decision and potential publication in PLOS Medicine.

Best regards,

Edward C. Smith, MD
